# Supplementary material for: MEF2C regulates cortical inhibitory and excitatory synapses and behaviors relevant to neurodevelopmental disorders
Source: eLife. 2016 Oct 25;5:e20059. doi: 10.7554/eLife.20059 (PMC5094851; doi:10.7554/eLife.20059)
Supplement: Supplementary file 1. — DOI: http://dx.doi.org/10.7554/eLife.20059.019 [file elife-20059-supp1.docx]

Table S1. Gene expression in SSC of *MEF2C cKO* mice.

| ID | log2FC | pval | padj | SYN | ID | ASD | ASD_sc | FMRP | SZdb | SZ_108 | SZ_full |
| --- | --- | --- | --- | --- | --- | --- | --- | --- | --- | --- | --- |
| 0610007P14Rik | 0.221747726 | 0.045381808 | 0.192125958 |  |  |  |  |  |  |  |  |
| 0610009B22Rik | 0.192759788 | 0.134902046 | 0.399534068 |  |  |  |  |  |  |  |  |
| 0610009O20Rik | -0.014974225 | 0.719812057 | 0.897994027 |  |  |  |  |  |  |  |  |
| 0610010B08Rik | 0.084794236 | 0.751059135 | 0.914432554 |  |  |  |  |  |  |  |  |
| 0610010F05Rik | -0.073731924 | 0.384892594 | 0.698108192 |  |  |  |  |  |  |  |  |
| 0610010K14Rik | 0.05336589 | 0.824966942 | 0.941950287 |  |  |  |  |  |  |  |  |
| 0610012G03Rik | 0.056516052 | 0.887817508 | 0.962995126 |  |  |  |  |  |  |  |  |
| 0610030E20Rik | -0.045922794 | 0.902178824 | 0.969220665 |  |  |  |  |  |  |  |  |
| 0610031J06Rik | -0.022942131 | 0.745491874 | 0.911928524 |  |  |  |  |  |  |  |  |
| 1110002L01Rik | 0.044434399 | 0.770895561 | 0.921167131 |  |  |  |  |  |  |  |  |
| 1110004E09Rik | -0.016035961 | 0.985580927 | 0.996071145 |  |  |  |  |  |  |  |  |
| 1110004F10Rik | 0.067673198 | 0.437140064 | 0.737692921 |  |  |  |  |  |  |  |  |
| 1110007C09Rik | -0.042292805 | 0.835657084 | 0.943188599 |  |  |  |  |  |  |  |  |
| 1110008F13Rik | 0.004799259 | 0.936573564 | 0.98530815 |  |  |  |  |  |  |  |  |
| 1110018G07Rik | 0.111309439 | 0.309007432 | 0.625861676 |  |  |  |  | FMRP |  |  |  |
| 1110032A03Rik | -0.110592944 | 0.287933 | 0.606211099 |  |  |  |  |  |  |  |  |
| 1110032F04Rik | 0.601979243 | 1.93164E-05 | 0.000261131 |  |  |  |  |  |  |  |  |
| 1110037F02Rik | -0.074739231 | 0.602003352 | 0.837095487 |  |  |  |  |  |  |  |  |
| 1110051M20Rik | 0.046141895 | 0.805934131 | 0.936945007 |  |  |  |  |  |  |  |  |
| 1110057K04Rik | 0.169174382 | 0.100661903 | 0.331897451 |  |  |  |  |  |  |  |  |
| 1190002N15Rik | -0.131261974 | 0.472555982 | 0.76369839 |  | ID | ASD | ASD_sc |  |  |  |  |
| 1200011I18Rik | -0.171527903 | 0.332720174 | 0.652193686 |  |  |  |  |  |  |  |  |
| 1200014J11Rik | 0.20971499 | 0.219396609 | 0.529180686 |  |  |  |  |  |  |  |  |
| 1300018J18Rik | -0.040044908 | 0.838722122 | 0.94473205 |  |  |  |  |  |  |  |  |
| 1500004A13Rik | 0.059160767 | 0.698578764 | 0.887098268 |  |  |  |  |  |  |  |  |
| 1500009C09Rik | 0.400868234 | 1.81605E-06 | 3.00514E-05 |  |  |  |  |  |  |  |  |
| 1500011B03Rik | -0.116700125 | 0.160145346 | 0.441814506 |  |  |  |  |  |  |  |  |
| 1500032L24Rik | 0.198586899 | 0.265397092 | 0.581700248 |  |  |  |  |  |  |  |  |
| 1600012H06Rik | -0.039469107 | 0.870089466 | 0.956485207 |  |  |  |  |  |  |  |  |
| 1600014C10Rik | -0.050811153 | 0.791097295 | 0.931851876 |  |  |  |  |  |  |  |  |
| 1700007B14Rik | 0.148237578 | 0.629567588 | 0.853694505 |  |  |  |  |  |  |  |  |
| 1700011I03Rik | -0.182486468 | 0.152849424 | 0.428817098 |  |  |  |  |  |  |  |  |
| 1700012B15Rik | -0.139909057 | 0.188824423 | 0.485468048 |  |  |  |  |  |  |  |  |
| 1700017B05Rik | -0.192267307 | 0.395445346 | 0.706399122 |  |  |  |  |  |  |  |  |
| 1700019G17Rik | -0.01301949 | 0.946760227 | 0.986189928 |  |  |  |  |  |  |  |  |
| 1700020I14Rik | -0.07077018 | 0.753581052 | 0.915220708 |  |  |  |  |  |  |  |  |
| 1700021F05Rik | -0.043737455 | 0.628183835 | 0.852766489 |  |  |  |  |  |  |  |  |
| 1700021K19Rik | -0.057545389 | 0.582498662 | 0.825996496 |  |  |  |  | FMRP |  |  |  |
| 1700025G04Rik | -0.361332459 | 0.000166207 | 0.001803631 |  |  |  |  |  |  |  |  |
| 1700086L19Rik | -0.420123463 | 0.003049362 | 0.022624849 |  |  |  |  |  |  |  |  |
| 1700109H08Rik | -0.155545657 | 0.175552542 | 0.465276844 |  |  |  |  |  |  |  |  |
| 1700111N16Rik | -0.207099318 | 0.265030396 | 0.58143683 |  |  |  |  |  |  |  |  |
| 1810013D10Rik | 0.045713558 | 0.561528817 | 0.81417085 |  |  |  |  |  |  |  |  |
| 1810013L24Rik | -0.017354275 | 0.940153546 | 0.985527867 |  |  |  |  |  |  |  |  |
| 1810014B01Rik | -0.114957739 | 0.469345485 | 0.762246238 |  |  |  |  |  |  |  |  |
| 1810026J23Rik | 0.113189163 | 0.137667654 | 0.405030324 |  |  |  |  |  |  |  |  |
| 1810037I17Rik | 0.254401107 | 0.010043669 | 0.059560077 |  |  |  |  |  |  |  |  |
| 1810055G02Rik | -0.24859026 | 0.049501234 | 0.204889384 |  |  |  |  |  |  |  |  |
| 1810058I24Rik | 0.020900332 | 0.989999529 | 0.998007614 |  |  |  |  |  |  |  |  |
| 2010012O05Rik | 0.081782463 | 0.691312738 | 0.884120561 |  |  |  |  |  |  | SZ_108 | SZ_full |
| 2010107E04Rik | 0.048093221 | 0.660970508 | 0.871388557 |  |  |  |  |  |  |  |  |
| 2010111I01Rik | 0.199945717 | 0.170989754 | 0.459498577 |  |  |  |  |  |  |  |  |
| 2010300C02Rik | 0.017293241 | 0.822724256 | 0.941605491 |  |  |  |  |  |  |  |  |
| 2210013O21Rik | 0.340439046 | 0.001095362 | 0.009643058 |  |  |  |  |  |  |  |  |
| 2210016F16Rik | -0.081310123 | 0.905830392 | 0.970739161 |  |  |  |  |  |  |  |  |
| 2210016L21Rik | 0.142787922 | 0.136419192 | 0.402843196 |  |  |  |  |  |  |  |  |
| 2210018M11Rik | -0.053178057 | 0.483638836 | 0.768733231 |  |  |  |  |  |  |  |  |
| 2210404J11Rik | -0.073375274 | 0.44698061 | 0.744686785 |  |  |  |  |  |  |  |  |
| 2300009A05Rik | 0.161475169 | 0.266096421 | 0.582912677 |  |  |  |  |  |  |  |  |
| 2310010M20Rik | 0.066410938 | 0.777673466 | 0.924797943 |  |  |  |  |  |  |  |  |
| 2310022A10Rik | -0.065827879 | 0.555445955 | 0.809875054 |  |  |  |  |  |  |  |  |
| 2310022B05Rik | 0.237921391 | 0.021808698 | 0.108988832 |  |  |  |  |  |  |  |  |
| 2310035C23Rik | 0.010003275 | 0.724255219 | 0.899896975 | SYN |  |  |  |  |  |  |  |
| 2310036O22Rik | 0.044414444 | 0.82087078 | 0.941239661 |  |  |  |  |  |  |  |  |
| 2310044G17Rik | 0.014726362 | 0.776591038 | 0.924077297 |  |  |  |  |  |  |  |  |
| 2310061I04Rik | -0.05766856 | 0.407005407 | 0.717408867 |  |  |  |  |  |  |  |  |
| 2310067B10Rik | -0.280188295 | 0.008357521 | 0.051399981 |  |  |  |  |  |  |  |  |
| 2410004B18Rik | 0.038615207 | 0.562942606 | 0.815331437 |  |  |  |  |  |  |  |  |
| 2410006H16Rik | -0.112377216 | 0.865023769 | 0.954203365 |  |  |  |  |  |  |  |  |
| 2410015M20Rik | 0.108615779 | 0.707524266 | 0.890939934 |  |  |  |  |  |  |  |  |
| 2410016O06Rik | -0.043249105 | 0.960708077 | 0.989984854 |  |  |  |  |  |  |  |  |
| 2410018M08Rik | 0.062301073 | 0.430139871 | 0.731710685 |  |  |  |  |  |  |  |  |
| 2410066E13Rik | -0.46414511 | 4.46053E-05 | 0.000554161 |  |  |  |  |  |  |  |  |
| 2410089E03Rik | 0.015450413 | 0.673931563 | 0.878026486 |  |  |  |  |  |  |  |  |
| 2510002D24Rik | 0.044126403 | 0.882729114 | 0.961049333 |  |  |  |  |  |  |  |  |
| 2510003E04Rik | 0.081241512 | 0.147996298 | 0.422465767 |  |  |  |  |  |  |  |  |
| 2510009E07Rik | -0.44845498 | 1.26039E-05 | 0.000177253 |  |  |  |  |  |  |  |  |
| 2610001J05Rik | 0.092859016 | 0.200903551 | 0.504464218 |  |  |  |  |  |  |  |  |
| 2610002J02Rik | 0.055731677 | 0.654983094 | 0.86959601 |  |  |  |  |  |  |  |  |
| 2610005L07Rik | -0.045674455 | 0.72521913 | 0.900427737 |  |  |  |  |  |  |  |  |
| 2610017I09Rik | 0.512048747 | 1.48509E-06 | 2.531E-05 |  |  |  |  |  |  |  |  |
| 2610044O15Rik8 | -0.33228463 | 0.000491772 | 0.004765949 |  |  |  |  |  |  |  |  |
| 2610100L16Rik | -0.282198744 | 0.142931247 | 0.413950482 |  |  |  |  |  |  |  |  |
| 2610301G19Rik | 0.020593557 | 0.989531834 | 0.997827478 |  |  |  |  |  |  |  |  |
| 2610507B11Rik | -0.106077672 | 0.31191557 | 0.629036306 |  |  |  |  | FMRP |  |  |  |
| 2700029M09Rik | -0.044619949 | 0.581547773 | 0.825342533 |  |  |  |  |  |  | SZ_108 | SZ_full |
| 2700049A03Rik | 0.044887348 | 0.945097593 | 0.985962641 |  |  |  |  |  |  |  |  |
| 2700050L05Rik | 0.134201529 | 0.290148534 | 0.607535998 |  |  |  |  |  |  |  |  |
| 2700060E02Rik | 0.009670026 | 0.857866864 | 0.951689326 |  |  |  |  |  |  |  |  |
| 2700062C07Rik | -0.081644074 | 0.594936942 | 0.833957302 |  |  |  |  |  |  |  |  |
| 2700081O15Rik | 0.209825549 | 0.03987575 | 0.173987408 |  |  |  |  |  |  |  |  |
| 2700089E24Rik | 0.033791984 | 0.701044273 | 0.887191673 |  |  |  |  |  |  |  |  |
| 2810001G20Rik | -0.004173182 | 0.929581976 | 0.981434219 |  |  |  |  |  |  |  |  |
| 2810004N23Rik | -0.029291447 | 0.67096519 | 0.87656975 |  |  |  |  |  |  |  |  |
| 2810006K23Rik | -0.054151943 | 0.807765505 | 0.93712146 |  |  |  |  |  |  | SZ_108 | SZ_full |
| 2810403A07Rik | 0.008653981 | 0.967694755 | 0.990673003 |  |  |  |  |  |  |  |  |
| 2810407C02Rik | 0.003472618 | 0.801674368 | 0.935393732 |  |  |  |  |  |  |  |  |
| 2810408M09Rik | -0.137886954 | 0.365684689 | 0.683032308 |  |  |  |  |  |  |  |  |
| 2810410L24Rik | 0.199152671 | 0.201580719 | 0.505123411 |  |  |  |  |  |  |  |  |
| 2810417H13Rik | -0.014386435 | 0.879181611 | 0.959555967 |  |  |  |  |  |  |  |  |
| 2810428I15Rik | 0.141380948 | 0.700775421 | 0.887191673 |  |  |  |  |  |  |  |  |
| 2810474O19Rik | 0.043186549 | 0.752296574 | 0.91454844 |  |  |  |  |  |  |  |  |
| 2900011O08Rik | -0.015754032 | 0.910189742 | 0.972262947 |  |  |  |  |  |  |  |  |
| 2900026A02Rik | -0.031249387 | 0.82859377 | 0.94210462 |  |  |  |  |  |  |  |  |
| 2900092D14Rik | 0.435189761 | 0.000439643 | 0.004334476 |  |  |  |  |  |  |  |  |
| 2900097C17Rik | -0.067516295 | 0.381543185 | 0.695268093 |  |  |  |  |  |  |  |  |
| 3000002C10Rik | 0.176458312 | 0.233619974 | 0.548048187 |  |  |  |  |  |  |  |  |
| 3110001D03Rik | 0.16650886 | 0.497119333 | 0.777150882 |  |  |  |  |  |  |  |  |
| 3110035E14Rik | 0.014516177 | 0.947465673 | 0.986441942 |  |  |  |  |  |  |  |  |
| 3110043O21Rik | 0.129108657 | 0.246003478 | 0.563224472 |  |  |  |  |  |  |  |  |
| 3110047P20Rik | 0.373506273 | 0.009947248 | 0.059076137 |  |  |  |  |  |  |  |  |
| 3110057O12Rik | 0.011703568 | 0.738603432 | 0.908748658 |  |  |  |  |  |  |  |  |
| 3632451O06Rik | -0.27407416 | 0.031630927 | 0.144909981 |  |  |  |  |  |  |  |  |
| 3830406C13Rik | -0.311905147 | 0.02688012 | 0.127237883 |  |  |  |  |  |  |  |  |
| 4632415K11Rik | 0.037199623 | 0.739405423 | 0.909125582 |  |  |  |  |  |  |  |  |
| 4632415L05Rik | 0.116210295 | 0.209178896 | 0.516216235 |  |  |  |  |  |  |  |  |
| 4732471J01Rik | 0.055411344 | 0.871512192 | 0.956962054 |  |  |  |  |  |  |  |  |
| 4833420G17Rik | -6.81678E-05 | 0.956840814 | 0.989355248 |  |  |  |  |  |  |  |  |
| 4833422C13Rik | 0.348776425 | 0.06104282 | 0.235615351 |  |  |  |  |  |  |  |  |
| 4833424O15Rik | -0.083392272 | 0.481089242 | 0.766666892 |  |  |  |  |  |  |  |  |
| 4833439L19Rik | 0.031441321 | 0.4772658 | 0.765665271 |  |  |  |  |  |  |  |  |
| 4930402H24Rik | 0.045999453 | 0.924978871 | 0.979179254 |  |  |  |  |  |  |  |  |
| 4930422G04Rik | -0.312982333 | 0.016994367 | 0.089505439 |  |  |  |  |  |  |  |  |
| 4930430F08Rik | 0.06157637 | 0.643751113 | 0.861470791 |  |  |  |  |  |  |  |  |
| 4930444A02Rik | 0.00975791 | 0.989077525 | 0.997735418 |  |  |  |  |  |  |  |  |
| 4930506M07Rik | 0.307728222 | 0.0026374 | 0.020246295 | SYN |  |  |  |  |  |  |  |
| 4930539E08Rik | -0.531314844 | 0.032255022 | 0.146925217 |  |  |  |  |  |  |  |  |
| 4930555G01Rik | -0.167404149 | 0.028194905 | 0.131973333 |  |  |  |  |  |  |  |  |
| 4930579G24Rik | 0.193904661 | 0.444094638 | 0.742734082 |  |  |  |  |  |  |  |  |
| 4930581F22Rik | -0.01968989 | 1 | 1 |  |  |  |  |  |  |  |  |
| 4931406C07Rik | -0.091695543 | 0.37481071 | 0.690094696 |  |  |  |  |  |  |  |  |
| 4931406P16Rik | -0.192680607 | 0.107840948 | 0.346970311 |  |  |  |  |  |  |  |  |
| 4931414P19Rik | 0.398968726 | 0.159450243 | 0.441129079 |  |  |  |  |  |  |  |  |
| 4931428F04Rik | 0.03302388 | 0.957544627 | 0.989355248 |  |  |  |  |  |  |  |  |
| 4932415G12Rik | -0.267292915 | 0.020030318 | 0.101731231 |  |  |  |  |  |  |  |  |
| 4932438A13Rik | -0.135744394 | 0.172819889 | 0.461934126 |  |  |  |  | FMRP |  |  |  |
| 4933407K13Rik | -0.095235963 | 0.454050049 | 0.75057061 |  |  |  |  |  |  |  |  |
| 4933411K20Rik | -0.12414037 | 0.36477848 | 0.681694742 |  |  |  |  |  |  |  |  |
| 4933426M11Rik | 0.749114828 | 2.22182E-14 | 1.15825E-12 |  |  |  |  |  |  |  |  |
| 4933431E20Rik | 0.312875719 | 0.000185103 | 0.001980088 |  |  |  |  |  |  |  |  |
| 4933434E20Rik | -0.21006287 | 0.063525759 | 0.242663533 |  |  |  |  |  |  |  |  |
| 4933439C10Rik | -0.863425466 | 2.1232E-17 | 1.52565E-15 |  |  |  |  |  |  |  |  |
| 5031434O11Rik | -0.439286094 | 0.022183554 | 0.110281768 |  |  |  |  |  |  |  |  |
| 5031439G07Rik | 0.144209213 | 0.071809489 | 0.262576126 |  |  |  |  |  |  |  |  |
| 5330417C22Rik | 0.050510423 | 0.723470856 | 0.899896975 |  |  |  |  |  |  |  |  |
| 5330434G04Rik | -0.304469404 | 0.034873218 | 0.15600044 |  |  |  |  |  |  |  |  |
| 5430417L22Rik | 0.122253854 | 0.13790615 | 0.405078683 |  |  |  |  |  |  |  |  |
| 5730409E04Rik | -0.03978647 | 0.716658625 | 0.897060451 |  |  |  |  |  |  |  |  |
| 5730455P16Rik | 0.001139959 | 0.971121865 | 0.99150896 |  |  |  |  |  |  |  |  |
| 5730507C01Rik | 0.044686509 | 0.678549737 | 0.878273493 |  |  |  |  |  |  |  |  |
| 5730522E02Rik | 0.097522772 | 0.766212582 | 0.920286747 |  |  |  |  |  |  |  |  |
| 5830417I10Rik | 0.009877587 | 0.908778003 | 0.971855926 |  |  |  |  |  |  |  |  |
| 6030419C18Rik | -0.008919424 | 0.802155957 | 0.935393732 |  |  |  |  |  |  |  |  |
| 6030458C11Rik | 0.048818542 | 0.425721133 | 0.73059413 |  |  |  |  |  |  |  |  |
| 6330403A02Rik | 0.974032763 | 6.98427E-13 | 2.97896E-11 |  |  |  |  |  |  |  |  |
| 6330403K07Rik | 0.443676451 | 7.04249E-08 | 1.52638E-06 |  |  |  |  |  |  |  |  |
| 6430548M08Rik | 0.186583647 | 0.01307188 | 0.07285906 | SYN |  |  |  |  |  |  |  |
| 6530402F18Rik | 0.959758022 | 4.53686E-12 | 1.76517E-10 |  |  |  |  |  |  |  |  |
| 6720401G13Rik | 0.117478569 | 0.360765974 | 0.677689451 |  |  |  |  |  |  |  |  |
| 6720456H20Rik | -0.156856827 | 0.262298375 | 0.579366335 |  |  |  |  |  |  |  |  |
| 6820431F20Rik | -0.053933695 | 0.945122069 | 0.985962641 |  |  |  |  |  |  |  |  |
| 8030462N17Rik | 0.051752072 | 0.517204695 | 0.789456636 |  |  |  |  |  |  |  |  |
| 8430410A17Rik | 0.035290622 | 0.812743983 | 0.938396933 |  |  |  |  |  |  |  |  |
| 8430419L09Rik | 0.607098339 | 0.006650864 | 0.0427456 |  |  |  |  |  |  |  |  |
| 8430427H17Rik | 0.167056465 | 0.176494227 | 0.466440673 |  |  |  |  |  |  |  |  |
| 9030624J02Rik | 0.065267025 | 0.49024578 | 0.773684278 |  |  |  |  |  |  |  |  |
| 9130011E15Rik | -0.166323717 | 0.145468915 | 0.418538798 |  |  |  |  |  |  |  |  |
| 9130024F11Rik | 0.073358072 | 0.478373806 | 0.765984483 |  |  |  |  |  |  |  |  |
| 9130401M01Rik | -0.139074854 | 0.342043032 | 0.662914472 |  |  |  |  |  |  |  |  |
| 9230114K14Rik | 0.031075081 | 0.676131457 | 0.878273493 |  |  |  |  |  |  |  |  |
| 9330151L19Rik | -0.107432338 | 0.514627392 | 0.787995408 |  |  |  |  |  |  |  |  |
| 9330182L06Rik | -0.390796107 | 0.002033964 | 0.016419935 |  |  |  |  |  |  |  |  |
| 9430016H08Rik | 0.003996104 | 0.880511301 | 0.960094948 |  |  |  |  |  |  | SZ_108 | SZ_full |
| 9430020K01Rik | -0.249446027 | 0.299156117 | 0.616077767 |  |  |  |  |  |  |  |  |
| 9530036O11Rik | -0.402580247 | 0.002526853 | 0.019529246 |  |  |  |  |  |  |  |  |
| 9530068E07Rik | 0.084682184 | 0.270083996 | 0.588724906 |  |  |  |  |  |  |  |  |
| 9930013L23Rik | -0.15040851 | 0.261106459 | 0.578754638 |  |  |  |  |  |  |  |  |
| 9930021J03Rik | -0.147498129 | 0.357907677 | 0.675972682 |  |  |  |  |  |  |  |  |
| 9930104L06Rik | 0.120105366 | 0.39755402 | 0.707892459 |  |  |  |  |  |  |  |  |
| A030009H04Rik | -0.210907136 | 0.007657002 | 0.047899802 |  |  |  |  |  |  |  |  |
| A130040M12Rik | 0.083723765 | 0.465427748 | 0.759462298 |  |  |  |  |  |  |  |  |
| A230046K03Rik | 0.072881961 | 0.654735899 | 0.86959601 | SYN |  |  |  |  |  |  |  |
| A230050P20Rik | 0.064727286 | 0.850123724 | 0.948352649 |  |  |  |  |  |  |  |  |
| A230057D06Rik | 0.299793526 | 0.259610837 | 0.576917826 |  |  |  |  |  |  |  |  |
| A230072C01Rik | 0.0625823 | 0.861534334 | 0.953064889 |  |  |  |  |  |  |  |  |
| A230073K19Rik | -0.003710708 | 0.639689528 | 0.860142008 |  |  |  |  |  |  |  |  |
| A330023F24Rik | -0.11407216 | 0.108727675 | 0.348562975 |  |  |  |  |  |  |  |  |
| A330050F15Rik | 0.050456369 | 0.536173029 | 0.799449638 |  |  |  |  |  |  |  |  |
| A330076H08Rik | 0.457784714 | 6.17986E-05 | 0.00074796 |  |  |  |  |  |  |  |  |
| A430005L14Rik | -0.009106959 | 0.980059055 | 0.994079139 |  |  |  |  |  |  |  |  |
| A430033K04Rik | 0.055069678 | 0.835898949 | 0.943188599 |  |  |  |  |  |  |  |  |
| A530054K11Rik | 0.015664385 | 0.826822351 | 0.941969116 |  |  |  |  |  |  |  |  |
| A730017C20Rik | -0.056522431 | 0.849270557 | 0.9478234 |  |  |  |  |  |  |  |  |
| A730017L22Rik | -0.073696594 | 0.357045001 | 0.675290951 |  |  |  |  |  |  |  |  |
| A830010M20Rik | 0.124125058 | 0.540853767 | 0.802680899 |  |  |  |  |  |  |  |  |
| A830018L16Rik | 0.369714649 | 7.2946E-06 | 0.000107944 |  |  |  |  |  |  |  |  |
| A930017M01Rik | -0.153585878 | 0.209297075 | 0.516221607 |  |  |  |  |  |  |  |  |
| AA465934 | 0.978723384 | 0.013224569 | 0.073504643 |  |  |  |  |  |  |  |  |
| AA474331 | -0.081447704 | 0.3435608 | 0.664139831 |  |  |  |  |  |  |  |  |
| Aaas | 0.013498753 | 0.967648112 | 0.990673003 |  |  |  |  |  |  |  |  |
| Aacs | 0.069417648 | 0.458217725 | 0.754249674 |  |  |  |  |  |  |  |  |
| Aagab | 0.094815173 | 0.284778596 | 0.602965919 |  |  |  |  |  |  |  |  |
| Aak1 | -0.064397472 | 0.485047786 | 0.770205285 | SYN |  |  |  | FMRP |  |  |  |
| Aamdc | 0.192278626 | 0.30159284 | 0.619496392 |  |  |  |  |  |  |  |  |
| Aamp | 0.09241655 | 0.260941053 | 0.578754638 |  |  |  |  |  |  |  |  |
| Aar2 | -0.01146475 | 0.967602781 | 0.990673003 |  |  |  |  |  |  |  |  |
| Aars | 0.049452992 | 0.584960747 | 0.827389061 | SYN |  |  |  |  |  |  |  |
| Aars2 | 0.149284567 | 0.261379698 | 0.578754638 |  |  |  |  |  |  |  |  |
| Aarsd1 | 0.122070739 | 0.403720652 | 0.712938716 |  |  |  |  |  |  |  |  |
| Aasdh | -0.080723322 | 0.420436781 | 0.727419472 |  |  |  |  |  |  |  |  |
| Aasdhppt | 0.046646149 | 0.717296143 | 0.897295176 | SYN |  |  |  |  |  |  |  |
| Aatf | -0.082165035 | 0.325443291 | 0.643145612 |  |  |  |  |  |  |  |  |
| Aatk | 0.008457253 | 0.85606351 | 0.951377789 |  |  |  |  | FMRP |  |  |  |
| Abat | -0.100699656 | 0.254223559 | 0.571324767 |  | ID | ASD | ASD_sc |  |  |  |  |
| Abca1 | 0.082607507 | 0.461584963 | 0.756884708 |  |  |  |  |  |  |  |  |
| Abca2 | 0.19621886 | 0.013504088 | 0.074487279 |  |  |  |  | FMRP |  |  |  |
| Abca3 | -0.146821199 | 0.056175403 | 0.22224951 |  |  |  |  | FMRP |  |  |  |
| Abca4 | -1.170116916 | 0.003251521 | 0.023814627 |  |  |  |  |  |  |  |  |
| Abca5 | -0.285082316 | 0.00563337 | 0.037164399 |  |  |  |  |  |  |  |  |
| Abca8a | 0.827011118 | 3.33595E-05 | 0.000428463 |  |  |  |  |  |  |  |  |
| Abca8b | 0.072541892 | 0.492060874 | 0.774709342 |  |  |  |  |  |  |  |  |
| Abcb1b | 0.101539265 | 0.678577047 | 0.878273493 | SYN |  |  |  |  |  |  |  |
| Abcb6 | 0.229335495 | 0.067761849 | 0.253028326 |  |  |  |  |  |  |  |  |
| Abcb8 | 0.086214387 | 0.496510652 | 0.77686598 | SYN |  |  |  |  |  |  |  |
| Abcb9 | -0.296238425 | 0.00041082 | 0.004060346 |  |  |  |  |  |  | SZ_108 | SZ_full |
| Abcc1 | 0.045071229 | 0.827130154 | 0.941969116 |  |  |  |  |  |  |  |  |
| Abcc4 | 0.15209244 | 0.243478965 | 0.55972495 |  |  |  |  |  |  |  |  |
| Abcc5 | -0.035337402 | 0.542069727 | 0.80318561 |  |  |  |  |  |  |  |  |
| Abcc8 | -0.400411203 | 1.83254E-05 | 0.000248577 |  | ID |  |  |  |  |  |  |
| Abcd2 | -0.331760021 | 0.00302972 | 0.02254202 |  |  |  |  |  |  |  |  |
| Abcd4 | 0.279507921 | 0.050286387 | 0.20651797 |  |  |  |  |  |  |  |  |
| Abce1 | 0.170405112 | 0.049635452 | 0.20521492 |  |  |  |  |  |  |  |  |
| Abcf1 | 0.126184024 | 0.162498283 | 0.445185011 |  |  |  |  |  |  |  |  |
| Abcf2 | 0.002612864 | 0.773663951 | 0.923211202 |  |  |  |  |  |  |  |  |
| Abcf3 | 0.041547208 | 0.68316532 | 0.88070577 | SYN |  |  |  |  |  |  |  |
| Abcg1 | -0.078529999 | 0.618781888 | 0.848007618 |  |  |  |  | FMRP |  |  |  |
| Abhd10 | -0.038854833 | 0.945787383 | 0.985962641 |  |  |  |  |  |  |  |  |
| Abhd12 | -0.077509916 | 0.383782012 | 0.697684359 |  |  |  |  |  |  |  |  |
| Abhd13 | -0.068677755 | 0.712977614 | 0.894981027 |  |  |  |  |  |  |  |  |
| Abhd16a | -0.280788513 | 0.003492862 | 0.025211827 |  |  |  |  |  |  |  |  |
| Abhd2 | -0.181367615 | 0.026555013 | 0.125773626 |  |  |  |  |  |  |  |  |
| Abhd3 | -0.060663089 | 0.355880913 | 0.674719677 |  |  |  |  |  |  |  |  |
| Abhd5 | -0.0329682 | 0.829012065 | 0.942131349 |  |  |  |  |  |  |  |  |
| Abi1 | 0.081228519 | 0.165400799 | 0.450445915 | SYN |  |  |  |  |  |  |  |
| Abi2 | -0.017168532 | 0.957036886 | 0.989355248 | SYN |  |  |  |  |  |  |  |
| Abl1 | -0.027349251 | 0.920728703 | 0.977398024 |  |  |  |  |  |  |  |  |
| Ablim1 | -0.141045973 | 0.05495917 | 0.21873969 | SYN |  |  |  |  |  |  |  |
| Ablim2 | -0.041906595 | 0.52549922 | 0.793545744 | SYN |  |  |  |  |  |  |  |
| Abr | -0.062878858 | 0.651069995 | 0.866499964 | SYN |  |  |  | FMRP |  |  |  |
| Abt1 | -0.060140635 | 0.746753569 | 0.912020725 |  |  |  |  |  |  |  |  |
| Abtb1 | -0.131486642 | 0.161563825 | 0.443925987 |  |  |  |  |  |  |  |  |
| Acaa1a | 0.08231628 | 0.496644656 | 0.77686598 | SYN |  |  |  |  |  |  |  |
| Acaa2 | -0.172969282 | 0.199963193 | 0.502696517 |  |  |  |  |  |  |  |  |
| Acaca | -0.102126214 | 0.268983355 | 0.587353112 | SYN |  |  |  |  |  |  |  |
| Acad11 | -0.192120753 | 0.089052856 | 0.304060607 |  |  |  |  |  |  |  |  |
| Acad8 | -0.012165563 | 0.896881109 | 0.966457471 |  |  |  |  |  |  |  |  |
| Acad9 | -0.03014638 | 0.671056638 | 0.87656975 |  |  |  |  |  |  |  |  |
| Acadm | -0.12019795 | 0.308354743 | 0.625257997 |  |  |  |  |  |  |  |  |
| Acadsb | -0.120406778 | 0.195236042 | 0.496398685 |  |  |  |  |  |  |  |  |
| Acadvl | 0.011534492 | 0.922559015 | 0.97811122 |  |  |  |  |  |  |  |  |
| Acap2 | 0.419630181 | 8.52063E-07 | 1.52378E-05 |  |  |  |  |  |  |  |  |
| Acap3 | 0.102739766 | 0.296851077 | 0.613072033 |  |  |  |  |  |  |  |  |
| Acat1 | 0.13970801 | 0.249422716 | 0.566779369 | SYN |  |  |  |  |  |  |  |
| Acat2 | 0.060258947 | 0.496675376 | 0.77686598 |  |  |  |  |  |  |  |  |
| Acat3 | 0.139283042 | 0.32333297 | 0.640880659 |  |  |  |  |  |  |  |  |
| Acbd3 | 0.001199577 | 0.76976783 | 0.921167131 |  |  |  |  |  |  |  |  |
| Acbd5 | -0.111006113 | 0.302593068 | 0.620198102 | SYN |  |  |  |  |  |  |  |
| Acbd6 | 0.006588683 | 0.753858225 | 0.915220708 |  |  |  |  |  |  |  |  |
| Ace | -0.212494993 | 0.832231052 | 0.943106438 |  |  |  |  |  |  |  |  |
| Acer3 | -0.19866296 | 0.063284978 | 0.242091598 |  |  |  |  |  |  |  |  |
| Ache | -0.079580312 | 0.498793783 | 0.779005132 |  |  |  |  |  |  |  |  |
| Acin1 | 0.164233086 | 0.094804871 | 0.318209315 |  |  |  |  |  |  |  |  |
| Acly | -0.146268838 | 0.344512044 | 0.664850729 | SYN |  |  |  | FMRP |  |  |  |
| Aco1 | -0.037554192 | 0.687814194 | 0.8825621 |  |  |  |  |  |  |  |  |
| Aco2 | 0.137443704 | 0.069652537 | 0.258394715 | SYN |  |  |  | FMRP |  |  |  |
| Acot11 | -0.324902042 | 0.002858451 | 0.021528803 |  |  |  |  |  |  |  |  |
| Acot2 | 0.20348558 | 0.071640991 | 0.26211401 |  |  |  |  |  |  |  |  |
| Acot7 | 0.101832706 | 0.248593624 | 0.565539859 | SYN |  |  |  |  |  |  |  |
| Acot8 | 0.147908367 | 0.221070948 | 0.53206454 | SYN |  |  |  |  |  |  |  |
| Acot9 | -0.150485077 | 0.462933729 | 0.757441468 |  |  |  |  |  |  |  |  |
| Acox1 | 0.17551988 | 0.020911914 | 0.105565457 |  | ID |  |  |  |  |  |  |
| Acox3 | -0.091965346 | 0.401958659 | 0.711185063 |  |  |  |  |  |  |  |  |
| Acp1 | 0.091750999 | 0.34013486 | 0.660719834 | SYN |  |  |  |  |  |  |  |
| Acp2 | 0.033408165 | 0.646823345 | 0.863491334 |  |  |  |  |  |  |  |  |
| Acp6 | 0.312880951 | 0.035958665 | 0.160048166 |  |  |  |  |  |  |  |  |
| Acpl2 | -0.537732853 | 7.98619E-05 | 0.000939496 |  |  |  |  |  |  |  |  |
| Acsbg1 | -0.20107752 | 0.059264436 | 0.230632301 | SYN |  |  |  |  |  |  |  |
| Acsl3 | 0.00474327 | 0.876751837 | 0.959227516 | SYN |  |  |  |  |  |  |  |
| Acsl4 | -0.078664517 | 0.61617405 | 0.846761583 | SYN | ID |  |  |  |  |  |  |
| Acsl5 | -0.024977358 | 0.7804648 | 0.926216702 |  |  |  |  |  |  |  |  |
| Acsl6 | -0.046933055 | 0.378581583 | 0.693356305 | SYN |  |  |  |  | SZdb |  | SZ_full |
| Acss1 | 0.135719358 | 0.285488558 | 0.603673579 |  |  |  |  |  |  |  |  |
| Acss2 | -0.143964845 | 0.064049205 | 0.243729228 |  |  |  |  |  |  |  |  |
| Acta1 | 0.484891069 | 0.024564886 | 0.119178546 | SYN |  |  |  |  |  |  |  |
| Actb | 0.288690455 | 8.64192E-05 | 0.001012158 | SYN |  |  |  | FMRP |  |  |  |
| Actg1 | 0.187625874 | 0.010754923 | 0.062580899 | SYN |  |  |  |  |  |  |  |
| Actl6b | 0.143963043 | 0.064710997 | 0.245427917 | SYN |  |  |  |  |  |  |  |
| Actn1 | -0.082449049 | 0.554349229 | 0.808759732 | SYN |  |  |  |  |  |  |  |
| Actn4 | -0.118736532 | 0.168776377 | 0.456431258 | SYN |  |  |  |  |  |  |  |
| Actr10 | -0.120064194 | 0.294941359 | 0.611588794 |  |  |  |  |  |  |  |  |
| Actr1a | -0.015677667 | 0.954708303 | 0.989057465 | SYN |  |  |  |  |  |  |  |
| Actr1b | -0.345349691 | 6.91684E-05 | 0.000822186 | SYN |  |  |  |  |  |  |  |
| Actr2 | 0.035920463 | 0.414745899 | 0.723063015 | SYN |  |  |  |  |  |  |  |
| Actr3 | -0.082889812 | 0.542348409 | 0.803401127 | SYN |  |  |  |  |  |  |  |
| Actr3b | 0.24721102 | 0.002946551 | 0.022064462 | SYN |  |  |  |  |  |  |  |
| Actr6 | 0.05754382 | 0.744274319 | 0.911738899 |  |  |  |  |  |  |  |  |
| Actr8 | -0.057661486 | 0.568839146 | 0.819376892 |  |  |  |  |  |  |  |  |
| Acvr1 | 0.217944771 | 0.01478579 | 0.080116481 |  |  |  |  |  |  |  |  |
| Acvr1b | -0.412697935 | 1.17596E-05 | 0.000166894 |  |  |  |  |  |  |  |  |
| Acvr1c | -0.342718538 | 0.004402315 | 0.030506401 |  |  |  |  |  |  |  |  |
| Adal | 0.026336602 | 0.975204219 | 0.99211875 |  |  |  |  |  |  |  |  |
| Adam10 | 0.184590863 | 0.020079247 | 0.101877911 |  |  |  |  |  |  |  |  |
| Adam11 | -0.377251021 | 6.61571E-05 | 0.000791107 | SYN |  |  |  |  |  |  |  |
| Adam15 | 0.163145862 | 0.053635739 | 0.215593817 |  |  |  |  |  |  |  |  |
| Adam17 | 0.189917469 | 0.362139167 | 0.67930903 |  |  |  |  |  |  |  |  |
| Adam19 | 0.421839936 | 6.43147E-05 | 0.000773717 |  |  |  |  |  |  |  |  |
| Adam22 | 0.024315288 | 0.719535184 | 0.897994027 | SYN |  |  |  |  |  |  |  |
| Adam23 | -0.288213215 | 0.000360371 | 0.003620052 | SYN |  |  |  |  |  |  |  |
| Adam9 | -0.239331231 | 0.011542854 | 0.066025139 |  |  |  |  |  |  |  |  |
| Adamts16 | 0.89469528 | 0.012760255 | 0.071321507 |  |  |  |  |  |  |  |  |
| Adamts2 | 0.215788919 | 0.218050747 | 0.527501596 |  |  |  |  |  |  |  |  |
| Adamts20 | -0.39481599 | 0.001149088 | 0.010049477 |  |  |  |  |  |  |  |  |
| Adamts3 | 1.022731909 | 4.14171E-23 | 3.98003E-21 |  |  |  |  |  |  |  |  |
| Adamts4 | 0.394961583 | 0.096416711 | 0.321362177 |  |  |  |  |  |  |  |  |
| Adamtsl1 | 0.456685295 | 0.005333072 | 0.035629216 |  |  |  |  |  |  |  |  |
| Adap1 | -0.175602671 | 0.020532421 | 0.103715383 |  |  |  |  |  |  |  |  |
| Adar | -0.1548891 | 0.08197486 | 0.288922441 | SYN |  |  |  |  |  |  |  |
| Adarb1 | -0.631740059 | 4.19164E-14 | 2.14267E-12 |  |  | ASD |  | FMRP |  |  |  |
| Adarb2 | 0.073426872 | 0.829404526 | 0.942131349 |  |  |  |  |  |  |  |  |
| Adc | -0.088295877 | 0.35487045 | 0.674076378 |  |  |  |  |  |  |  |  |
| Adck1 | -0.167070535 | 0.090060158 | 0.306712134 |  |  |  |  |  |  |  |  |
| Adck3 | 0.049639346 | 0.664474296 | 0.873548402 |  |  |  |  |  |  |  |  |
| Adck5 | 0.102701836 | 0.449932235 | 0.746704017 |  |  |  |  |  |  |  |  |
| Adcy1 | 0.209226205 | 0.004553132 | 0.031360775 |  |  |  |  | FMRP |  |  |  |
| Adcy2 | -0.436516047 | 2.32339E-07 | 4.55317E-06 |  |  |  |  |  |  |  |  |
| Adcy5 | 0.092617689 | 0.378484571 | 0.693356305 |  |  | ASD |  | FMRP |  |  |  |
| Adcy6 | -0.12065921 | 0.22912295 | 0.541892633 |  |  |  |  |  |  |  |  |
| Adcy8 | -0.528101409 | 0.000230754 | 0.002428089 |  |  |  |  |  |  |  |  |
| Adcy9 | 0.495009371 | 1.11439E-07 | 2.31468E-06 |  |  |  |  |  |  |  |  |
| Adcyap1 | -1.307055096 | 6.54353E-07 | 1.19158E-05 |  |  |  |  |  | SZdb |  | SZ_full |
| Adcyap1r1 | 0.321140761 | 4.19681E-05 | 0.000526614 |  |  |  |  |  |  |  |  |
| Add1 | -0.033712592 | 0.776557078 | 0.924077297 | SYN |  |  |  | FMRP |  |  |  |
| Add2 | 0.273365909 | 0.002278343 | 0.018009971 | SYN |  |  |  |  |  |  |  |
| Add3 | 0.006168096 | 0.793414906 | 0.932686409 | SYN |  |  |  |  |  |  |  |
| Adh5 | 0.060908693 | 0.368191029 | 0.684543508 |  |  |  |  |  |  |  |  |
| Adhfe1 | -0.440487804 | 0.192304546 | 0.491924651 |  |  |  |  |  |  |  |  |
| Adi1 | 0.964568761 | 0.000106754 | 0.001216381 |  |  |  |  |  |  |  |  |
| Adipor1 | 0.048342255 | 0.464578445 | 0.758852689 |  |  |  |  |  |  |  |  |
| Adipor2 | 0.122819896 | 0.192778102 | 0.492977923 |  |  |  |  |  |  |  |  |
| Adk | -0.244855426 | 0.013311205 | 0.073729288 |  |  | ASD | ASD_sc |  |  |  |  |
| Adnp | -0.030408371 | 0.718613221 | 0.897816268 |  |  | ASD | ASD_sc | FMRP |  |  |  |
| Adnp2 | -0.029475382 | 0.699140921 | 0.887191673 |  |  |  |  |  |  |  |  |
| Adora1 | 0.213648646 | 0.014414091 | 0.078512548 |  |  |  |  |  |  |  |  |
| Adprh | 0.073362595 | 0.538820006 | 0.801497271 |  |  |  |  |  |  |  |  |
| Adprhl2 | -0.000416496 | 0.972550931 | 0.991571804 |  |  |  |  |  |  |  |  |
| Adprm | -0.199821457 | 0.062630899 | 0.240473481 |  |  |  |  |  |  |  |  |
| Adra2c | 0.720468802 | 2.6911E-05 | 0.00035245 |  |  |  |  |  |  |  |  |
| Adrb1 | -0.485535521 | 1.02941E-05 | 0.000147938 |  |  |  |  |  |  |  |  |
| Adrbk1 | -0.258648769 | 0.006369799 | 0.041171408 | SYN |  |  |  | FMRP |  |  |  |
| Adrbk2 | -0.013014853 | 0.777736945 | 0.924797943 |  |  |  |  |  |  |  |  |
| Adrm1 | 0.244586852 | 0.055498818 | 0.220397674 |  |  |  |  |  |  |  |  |
| Adsl | 0.022601716 | 0.937846578 | 0.98530815 |  | ID | ASD | ASD_sc |  |  |  |  |
| Adss | 0.100560821 | 0.273396909 | 0.591112429 |  |  |  |  |  |  |  |  |
| Adssl1 | -0.429860511 | 0.020157979 | 0.102212358 |  |  |  |  |  |  |  |  |
| Aebp2 | -0.060318827 | 0.728649716 | 0.902465385 |  |  |  |  |  |  |  |  |
| Aes | 0.185423929 | 0.30274205 | 0.620259592 |  |  |  |  |  |  |  |  |
| Afap1 | -0.054985074 | 0.613967947 | 0.845371525 |  |  |  |  |  |  |  |  |
| Afap1l1 | -1.434515207 | 7.95008E-39 | 2.18655E-36 |  |  |  |  |  |  |  |  |
| Aff3 | 0.438093204 | 0.000130209 | 0.001456589 |  |  |  |  | FMRP |  |  |  |
| Aff4 | 0.059922087 | 0.23382748 | 0.548048187 |  |  | ASD |  | FMRP |  |  |  |
| Afg3l1 | 0.145733495 | 0.227410284 | 0.540150216 |  |  |  |  |  |  |  |  |
| Afg3l2 | 0.002980528 | 0.937347568 | 0.98530815 | SYN |  |  |  |  |  |  |  |
| Aftph | -0.01539996 | 0.994577664 | 1 |  |  |  |  |  |  |  |  |
| Agap1 | -0.152889023 | 0.129061203 | 0.389037096 | SYN |  | ASD | ASD_sc |  |  |  |  |
| Agap2 | 0.086875716 | 0.128256148 | 0.387782804 | SYN |  |  |  |  |  |  |  |
| Agap3 | 0.100868499 | 0.282156183 | 0.600607877 | SYN |  |  |  |  |  |  |  |
| Agbl4 | -0.127847444 | 0.274188473 | 0.591865227 |  |  | ASD | ASD_sc |  |  |  |  |
| Agfg1 | -0.115858056 | 0.303772281 | 0.621556507 |  |  |  |  |  |  |  |  |
| Agfg2 | 0.190676016 | 0.096571507 | 0.321743667 |  |  |  |  |  |  |  |  |
| Aggf1 | 0.075383417 | 0.519263375 | 0.790842978 |  |  |  |  |  |  |  |  |
| Agk | -0.085324565 | 0.408217293 | 0.7189095 | SYN |  |  |  |  |  |  |  |
| Agl | -0.134631697 | 0.067737182 | 0.253028326 | SYN |  |  |  |  |  |  |  |
| Ago1 | 0.151896253 | 0.104363333 | 0.33947877 |  |  |  |  |  |  |  |  |
| Ago2 | 0.039959254 | 0.694211734 | 0.885065682 |  |  |  |  |  |  |  |  |
| Agpat1 | 0.066879617 | 0.557023485 | 0.810374491 |  |  |  |  |  |  |  |  |
| Agpat3 | -0.016398805 | 0.802243774 | 0.935393732 |  |  |  |  | FMRP |  |  |  |
| Agpat4 | 0.478725497 | 1.74442E-06 | 2.91687E-05 |  |  |  |  |  |  |  |  |
| Agpat5 | -0.057266638 | 0.55018809 | 0.807563527 | SYN |  |  |  |  |  |  |  |
| Agpat6 | -0.057414779 | 0.545059632 | 0.805331215 |  |  |  |  |  |  |  |  |
| Agps | 0.078590922 | 0.338746158 | 0.659792809 |  |  |  |  |  |  |  |  |
| Agrn | 0.022380824 | 0.944253026 | 0.985962641 | SYN |  |  |  | FMRP |  |  |  |
| Agtpbp1 | -0.23879501 | 0.015849118 | 0.084840648 |  |  |  |  | FMRP |  |  |  |
| Ahcy | 0.050688981 | 0.524420785 | 0.7932511 | SYN |  |  |  |  |  |  |  |
| Ahcyl1 | -0.046319887 | 0.632654516 | 0.855343327 | SYN |  |  |  |  |  |  |  |
| Ahcyl2 | 0.120523692 | 0.178779079 | 0.470763266 |  |  |  |  |  |  |  |  |
| Ahdc1 | -0.123476535 | 0.270361935 | 0.588732633 |  |  |  |  | FMRP |  |  |  |
| Ahi1 | 0.135135288 | 0.135726042 | 0.401241999 |  |  | ASD | ASD_sc |  | SZdb |  | SZ_full |
| Ahnak | 0.089455956 | 0.552852896 | 0.808060922 | SYN |  |  |  |  |  |  |  |
| Ahsa1 | 0.089983141 | 0.187728884 | 0.483929674 | SYN |  |  |  |  |  |  |  |
| Ahsa2 | 0.186725025 | 0.104219973 | 0.339150757 |  |  |  |  |  |  |  |  |
| AI314180 | 0.003102698 | 0.866097406 | 0.954438815 |  |  |  |  |  |  |  |  |
| AI316807 | -0.148857902 | 0.518525021 | 0.790473159 |  |  |  |  |  |  |  |  |
| AI414108 | 0.127996279 | 0.25327029 | 0.570531033 |  |  |  |  |  |  |  |  |
| AI462493 | -0.108280312 | 0.408905079 | 0.719062719 |  |  |  |  |  |  |  |  |
| AI464131 | 0.08734735 | 0.411737598 | 0.720653738 |  |  |  |  |  |  |  |  |
| AI593442 | -0.928909773 | 6.62737E-19 | 4.94018E-17 |  |  |  |  |  |  | SZ_108 | SZ_full |
| AI597468 | -0.282540139 | 0.030558304 | 0.140967628 |  |  |  |  |  |  |  |  |
| AI836003 | -0.4957832 | 0.000232246 | 0.002440574 |  |  |  |  |  |  |  |  |
| AI837181 | 0.076171196 | 0.503684213 | 0.782353511 |  |  |  |  |  |  |  |  |
| AI846148 | 0.052917628 | 0.895606202 | 0.965840329 |  |  |  |  |  |  |  |  |
| AI854703 | -0.157153879 | 0.099146342 | 0.328538108 |  |  |  |  |  |  |  |  |
| Aifm1 | 0.031475209 | 0.93024397 | 0.981434219 | SYN |  |  |  |  |  |  |  |
| Aifm3 | -0.104791185 | 0.233698654 | 0.548048187 | SYN |  |  |  |  |  |  |  |
| Aig1 | -0.157263834 | 0.087024381 | 0.300869729 |  |  |  |  |  |  |  |  |
| Aimp1 | 0.083663078 | 0.342739044 | 0.663516169 |  |  |  |  |  |  |  |  |
| Aip | 0.086242847 | 0.350541449 | 0.669369249 | SYN |  |  |  |  |  |  |  |
| Ajap1 | -0.691041006 | 1.38563E-06 | 2.38184E-05 |  |  |  |  |  |  |  |  |
| Ak1 | -0.05315019 | 0.627917278 | 0.852766489 | SYN | ID |  |  |  |  |  |  |
| AK129341 | 0.146333401 | 0.229230911 | 0.541892633 |  |  |  |  |  |  |  |  |
| Ak3 | 0.012573959 | 0.727121131 | 0.901526215 | SYN |  |  |  |  |  |  |  |
| Ak5 | -0.042760453 | 0.69593195 | 0.885994131 | SYN |  |  |  |  |  |  |  |
| Akap1 | -0.158444205 | 0.053045008 | 0.214112844 |  |  |  |  |  |  |  |  |
| Akap10 | -0.084477421 | 0.362921932 | 0.679847949 |  |  |  |  |  |  |  |  |
| Akap11 | -0.018847347 | 0.818572933 | 0.941021502 |  |  |  |  |  |  |  |  |
| Akap13 | -0.199741497 | 0.123974403 | 0.380022998 |  |  |  |  |  |  |  |  |
| Akap2 | -0.103533472 | 0.23536865 | 0.549721919 | SYN |  |  |  |  |  |  |  |
| Akap5 | -0.262769658 | 0.015042056 | 0.081064487 | SYN |  |  |  |  |  |  |  |
| Akap6 | 0.009154824 | 0.779008342 | 0.925572849 |  |  |  |  | FMRP |  |  |  |
| Akap7 | -0.520686929 | 1.96979E-06 | 3.2129E-05 |  |  |  |  |  |  |  |  |
| Akap8 | 0.060038828 | 0.845335083 | 0.946081097 |  |  |  |  |  |  |  |  |
| Akap8l | -0.108623317 | 0.15454245 | 0.431810113 |  |  |  |  |  |  |  |  |
| Akap9 | 0.142866225 | 0.124472159 | 0.380472824 | SYN |  |  |  | FMRP |  |  |  |
| Akirin1 | -0.047378319 | 0.586644462 | 0.828155084 |  |  |  |  |  |  |  |  |
| Akirin2 | -5.82029E-05 | 0.945216807 | 0.985962641 |  |  |  |  |  |  |  |  |
| Akr1a1 | -0.010813238 | 0.909955757 | 0.972262947 | SYN |  |  |  |  |  |  |  |
| Akr1b10 | 0.095205394 | 0.447213158 | 0.744686785 |  |  |  |  |  |  |  |  |
| Akr1b3 | -0.307482932 | 0.010198587 | 0.060210165 |  |  |  |  |  |  |  |  |
| Akr1c14 | -0.18513217 | 0.270863766 | 0.589228634 | SYN |  |  |  |  |  |  |  |
| Akr1e1 | -0.160117891 | 0.284153258 | 0.602965919 |  |  |  |  |  |  |  |  |
| Akr7a5 | 0.079314922 | 0.538680596 | 0.801497271 | SYN |  |  |  |  |  |  |  |
| Akt1 | -0.09524236 | 0.284695397 | 0.602965919 | SYN |  |  |  |  | SZdb |  | SZ_full |
| Akt2 | -0.240921504 | 0.003469832 | 0.025068276 | SYN |  |  |  |  |  |  |  |
| Akt3 | 0.233132968 | 0.008365369 | 0.051399981 |  |  |  |  | FMRP |  | SZ_108 | SZ_full |
| Aktip | -0.284057685 | 0.055925024 | 0.221438997 |  |  |  |  |  |  |  |  |
| Alas1 | 0.0152804 | 0.854577709 | 0.950775814 |  |  |  |  |  |  |  |  |
| Alcam | 0.43759639 | 1.16631E-07 | 2.40996E-06 | SYN |  |  |  |  |  |  |  |
| Aldh18a1 | 0.009451578 | 0.858248335 | 0.951938356 |  | ID |  |  |  |  |  |  |
| Aldh1a1 | 0.19458306 | 0.241821875 | 0.557723052 | SYN |  |  |  |  |  |  |  |
| Aldh1a2 | 0.308006024 | 0.288047795 | 0.606211099 |  |  |  |  |  |  |  |  |
| Aldh1l1 | -0.402890336 | 0.000112987 | 0.001274665 | SYN |  |  |  |  |  |  |  |
| Aldh1l2 | 0.523918363 | 0.010554768 | 0.061764362 |  |  |  |  |  |  |  |  |
| Aldh2 | -0.099188008 | 0.341675176 | 0.66258235 | SYN |  |  |  |  |  |  |  |
| Aldh3a2 | 0.070651119 | 0.437750827 | 0.737692921 | SYN | ID |  |  |  |  |  |  |
| Aldh5a1 | -0.063397229 | 0.528908503 | 0.795401684 | SYN | ID | ASD | ASD_sc |  |  |  |  |
| Aldh7a1 | -0.022748372 | 0.723665973 | 0.899896975 | SYN |  |  |  |  |  |  |  |
| Aldh9a1 | -0.152069624 | 0.138851584 | 0.406713271 |  |  |  |  |  |  |  |  |
| Aldoa | 0.076207423 | 0.172010966 | 0.461007885 | SYN | ID |  |  | FMRP |  | SZ_108 | SZ_full |
| Aldoart1 | 0.241597579 | 0.054047509 | 0.216515785 | SYN | ID |  |  |  |  | SZ_108 | SZ_full |
| Aldoc | -0.215946478 | 0.01069744 | 0.062370457 | SYN |  |  |  | FMRP |  |  |  |
| Alg10b | 0.178384519 | 0.09447354 | 0.317561314 |  |  |  |  |  |  |  |  |
| Alg11 | -0.145017395 | 0.399851292 | 0.709660416 |  |  |  |  |  |  |  |  |
| Alg12 | -0.095335037 | 0.321881314 | 0.6391151 |  | ID |  |  |  |  |  |  |
| Alg13 | -0.200000551 | 0.166367528 | 0.451803679 |  |  |  |  |  |  |  |  |
| Alg6 | 0.078384093 | 0.997239467 | 1 |  | ID |  |  |  |  |  |  |
| Alg9 | -0.142456289 | 0.262986954 | 0.57968857 |  |  |  |  |  |  |  |  |
| Alkbh4 | 0.131070219 | 0.560938903 | 0.813907347 |  |  |  |  |  |  |  |  |
| Alkbh5 | 0.126966316 | 0.067234329 | 0.252002352 |  |  |  |  |  |  |  |  |
| Alkbh8 | -0.096363597 | 0.456411339 | 0.75237881 |  |  |  |  |  |  |  |  |
| Alms1 | -0.303966852 | 0.01168556 | 0.066582117 |  |  |  |  |  |  |  |  |
| Alpl | -0.146513522 | 0.578950433 | 0.824002257 | SYN |  |  |  |  |  |  |  |
| Als2 | -0.020151402 | 0.82551883 | 0.941950287 |  |  |  |  | FMRP |  |  |  |
| Ambra1 | -0.169585458 | 0.044682395 | 0.190174377 |  |  |  |  |  |  | SZ_108 | SZ_full |
| Amd1 | -0.048013571 | 0.990657471 | 0.998292355 |  |  |  |  |  |  |  |  |
| Amdhd2 | -0.208020105 | 0.063393561 | 0.242274576 |  |  |  |  |  |  |  |  |
| Amer1 | 0.158229524 | 0.68267436 | 0.880635221 |  |  |  |  |  |  |  |  |
| Amer3 | -0.15815743 | 0.181834046 | 0.47488813 |  |  |  |  |  |  |  |  |
| Amfr | -0.004623017 | 0.795773166 | 0.933669722 |  |  |  |  |  |  |  |  |
| Amigo1 | -0.234796685 | 0.022549075 | 0.111639615 |  |  |  |  |  |  |  |  |
| Ammecr1 | -0.672564875 | 0.000745066 | 0.006862179 |  |  |  |  |  |  |  |  |
| Amn | 0.066282126 | 0.865412184 | 0.954310463 |  |  |  |  |  |  |  |  |
| Amn1 | -0.085591251 | 0.432144075 | 0.733800692 |  |  |  |  |  |  |  |  |
| Ampd2 | 0.391014145 | 4.20717E-05 | 0.000526788 | SYN |  |  |  |  |  |  |  |
| Ampd3 | -0.031406603 | 0.918300041 | 0.976215315 |  |  |  |  |  |  |  |  |
| Amph | 0.087856414 | 0.24756826 | 0.564495266 | SYN |  |  |  | FMRP |  |  |  |
| Amy1 | 0.042168616 | 0.763738891 | 0.919979858 |  |  |  |  |  |  |  |  |
| Amz2 | 0.115756836 | 0.176033656 | 0.46584089 |  |  |  |  |  |  |  |  |
| Anapc1 | -0.046160775 | 0.809904215 | 0.93712146 |  |  |  |  | FMRP |  |  |  |
| Anapc11 | 0.129631331 | 0.363115288 | 0.679860924 |  |  |  |  |  |  |  |  |
| Anapc15 | -0.034475777 | 0.762912346 | 0.919979858 |  |  |  |  |  |  |  |  |
| Anapc2 | -0.000109441 | 0.866231208 | 0.954438815 |  |  |  |  |  |  |  |  |
| Anapc4 | -0.010414434 | 0.991265278 | 0.998526378 |  |  |  |  |  |  |  |  |
| Anapc5 | -0.125675875 | 0.126438755 | 0.384767459 |  |  |  |  |  |  |  |  |
| Anapc7 | -0.103999867 | 0.420076682 | 0.727091941 |  |  |  |  |  |  |  |  |
| Angel1 | -0.095883065 | 0.381265106 | 0.695268093 |  |  |  |  |  |  |  |  |
| Angel2 | -0.241717015 | 0.010616666 | 0.062080966 |  |  |  |  |  |  |  |  |
| Ank | -0.035233267 | 0.654684177 | 0.86959601 |  |  |  |  |  |  |  |  |
| Ank1 | -0.379856839 | 0.000716846 | 0.006663827 | SYN |  |  |  | FMRP |  |  |  |
| Ank2 | 0.0928054 | 0.317686533 | 0.634736419 | SYN |  | ASD | ASD_sc | FMRP |  |  |  |
| Ank3 | -0.123881093 | 0.037485941 | 0.16546091 | SYN |  | ASD |  | FMRP |  |  |  |
| Ankfy1 | -0.040789753 | 0.442876281 | 0.741819716 | SYN |  |  |  |  |  |  |  |
| Ankhd1 | 0.006908777 | 0.945216835 | 0.985962641 |  |  |  |  |  |  |  |  |
| Ankib1 | 0.050885662 | 0.399122142 | 0.709155315 |  |  |  |  |  |  |  |  |
| Ankle2 | 0.027626917 | 0.865209622 | 0.954219019 |  |  |  |  |  |  |  |  |
| Ankmy2 | 0.043944523 | 0.656958117 | 0.869611321 |  |  |  |  |  |  |  |  |
| Ankra2 | 0.03510111 | 0.650012482 | 0.865959505 |  |  |  |  |  |  |  |  |
| Ankrd10 | 0.201815938 | 0.027489534 | 0.129278612 |  |  |  |  |  |  |  |  |
| Ankrd11 | -0.022874118 | 0.700784241 | 0.887191673 |  |  | ASD | ASD_sc | FMRP |  |  |  |
| Ankrd12 | -0.021612187 | 0.52450791 | 0.7932511 |  |  |  |  |  |  |  |  |
| Ankrd13a | 0.193442795 | 0.07195006 | 0.262883042 |  |  |  |  |  |  |  |  |
| Ankrd13b | 0.292077282 | 0.003290251 | 0.024054116 |  |  |  |  |  |  |  |  |
| Ankrd13c | -0.164526643 | 0.262802319 | 0.579626142 |  |  |  |  |  |  |  |  |
| Ankrd17 | -0.027450832 | 0.923119756 | 0.978235671 |  |  |  |  | FMRP |  |  |  |
| Ankrd27 | -0.031779313 | 0.622680557 | 0.849966377 |  |  |  |  |  |  |  |  |
| Ankrd28 | -0.08688035 | 0.506712132 | 0.784399099 |  |  |  |  |  |  |  |  |
| Ankrd33b | 0.530688299 | 1.20377E-09 | 3.41681E-08 |  |  |  |  |  |  |  |  |
| Ankrd34a | -0.0124145 | 0.800428673 | 0.935166555 |  |  |  |  |  |  |  |  |
| Ankrd35 | 0.601035644 | 7.48377E-05 | 0.000885617 |  |  |  |  |  |  |  |  |
| Ankrd40 | -0.031639412 | 0.719428612 | 0.897994027 |  |  |  |  |  |  |  |  |
| Ankrd44 | 0.13682675 | 0.232375472 | 0.546733559 |  |  |  |  |  |  | SZ_108 | SZ_full |
| Ankrd45 | 0.395485449 | 1.65433E-05 | 0.000226212 |  |  |  |  |  |  |  |  |
| Ankrd46 | 0.050998716 | 0.474586955 | 0.76408458 |  |  |  |  |  |  |  |  |
| Ankrd50 | -0.575184931 | 8.35397E-08 | 1.78159E-06 |  |  |  |  |  |  |  |  |
| Ankrd52 | 0.063505038 | 0.669563879 | 0.875926013 |  |  |  |  | FMRP |  |  |  |
| Ankrd54 | 0.244044889 | 0.073020227 | 0.265455483 |  |  |  |  |  |  |  |  |
| Ankrd6 | -0.855353212 | 1.98583E-11 | 7.4014E-10 |  |  |  |  |  |  |  |  |
| Anks1b | 0.148482318 | 0.139103234 | 0.406864883 | SYN |  |  |  |  |  |  |  |
| Anks3 | 0.142698427 | 0.275648375 | 0.593281983 |  |  |  |  |  |  |  |  |
| Anks6 | -0.075525079 | 0.368849309 | 0.685281419 |  |  |  |  |  |  |  |  |
| Ankzf1 | -0.05963609 | 0.365938586 | 0.683062524 |  |  |  |  |  |  |  |  |
| Anln | 0.333661875 | 0.080436931 | 0.285266768 |  |  |  |  |  |  |  |  |
| Ano10 | -0.564705608 | 3.32773E-06 | 5.22479E-05 |  |  |  |  |  |  |  |  |
| Ano3 | -0.262346939 | 0.056237689 | 0.222275425 |  |  |  |  |  |  |  |  |
| Ano4 | 0.18626762 | 0.133977285 | 0.397988249 |  |  |  |  |  |  |  |  |
| Ano6 | -0.171449288 | 0.225626429 | 0.538695632 |  |  |  |  |  |  |  |  |
| Ano8 | 0.062487252 | 0.586982343 | 0.82825333 |  |  |  |  |  |  |  |  |
| Anp32a | 0.044461699 | 0.450114317 | 0.746784025 |  |  |  |  |  |  |  |  |
| Anp32b | 0.112643694 | 0.21853524 | 0.527873129 |  |  |  |  |  |  |  |  |
| Anp32e | -0.215129644 | 0.042935578 | 0.184686665 |  |  |  |  |  |  | SZ_108 | SZ_full |
| Anxa11 | 0.282343969 | 0.004528164 | 0.03121576 | SYN |  |  |  |  |  |  |  |
| Anxa5 | -0.422360416 | 0.001382195 | 0.011816061 | SYN |  |  |  |  |  |  |  |
| Anxa6 | -0.019839376 | 0.657987001 | 0.869611321 | SYN |  |  |  |  |  |  |  |
| Anxa7 | 0.153934259 | 0.043493938 | 0.186209152 | SYN |  |  |  |  |  |  |  |
| Ap1ar | -0.033008211 | 0.646860504 | 0.863491334 |  |  |  |  |  |  |  |  |
| Ap1b1 | 0.099803302 | 0.232669624 | 0.547102865 | SYN |  |  |  | FMRP |  |  |  |
| Ap1g1 | -0.014068712 | 0.855811386 | 0.951377789 | SYN |  |  |  |  |  |  |  |
| Ap1g2 | 0.557314705 | 0.003239814 | 0.023750698 |  |  |  |  |  |  |  |  |
| Ap1m1 | 0.175836272 | 0.085265658 | 0.296589134 | SYN |  |  |  |  |  |  |  |
| Ap1s1 | 0.001310956 | 0.968764931 | 0.991454046 | SYN |  |  |  |  |  |  |  |
| Ap1s3 | -0.47402512 | 0.014887395 | 0.080557573 |  |  |  |  |  |  |  |  |
| Ap2a1 | 0.140953404 | 0.066325092 | 0.249650275 | SYN |  |  |  | FMRP |  |  |  |
| Ap2a2 | 0.290998368 | 9.72464E-05 | 0.001124112 | SYN |  |  |  | FMRP |  |  |  |
| Ap2b1 | -0.048946796 | 0.700662043 | 0.887191673 | SYN |  |  |  | FMRP |  |  |  |
| Ap2m1 | -0.051643102 | 0.670643333 | 0.87656975 | SYN |  |  |  |  |  |  |  |
| Ap3b1 | 0.052356164 | 0.705500488 | 0.889759184 | SYN |  |  |  |  |  |  |  |
| Ap3b2 | 0.050582072 | 0.556059731 | 0.810114044 | SYN |  |  |  |  |  |  |  |
| Ap3d1 | 0.041126428 | 0.556114528 | 0.810114044 | SYN |  |  |  | FMRP |  |  |  |
| Ap3m1 | -0.048059835 | 0.762000309 | 0.919472687 |  |  |  |  |  |  |  |  |
| Ap3m2 | -0.302897587 | 0.000619804 | 0.005850363 | SYN |  |  |  |  |  |  |  |
| Ap3s2 | -0.083815923 | 0.445443494 | 0.743822819 | SYN |  |  |  |  |  |  |  |
| Ap4e1 | -0.086989766 | 0.384877625 | 0.698108192 |  |  |  |  |  |  |  |  |
| Ap4s1 | -0.220708409 | 0.102426488 | 0.334817079 |  |  |  |  |  |  |  |  |
| Ap5m1 | 0.029028128 | 0.741901322 | 0.910790356 |  |  |  |  |  |  |  |  |
| Apaf1 | 0.326984215 | 0.020241642 | 0.102571371 |  |  |  |  |  |  |  |  |
| Apba1 | 0.483479702 | 2.40347E-09 | 6.52044E-08 |  |  |  |  | FMRP |  |  |  |
| Apba2 | -0.00718884 | 0.71146246 | 0.893500957 |  |  | ASD | ASD_sc |  |  |  |  |
| Apba2 | -0.00718884 | 0.71146246 | 0.893500957 |  |  | ASD | ASD_sc |  |  |  |  |
| Apbb1 | 0.003567641 | 0.83321067 | 0.943106438 |  |  |  |  | FMRP |  |  |  |
| Apbb2 | 0.024332819 | 0.927437359 | 0.980676173 |  |  |  |  |  |  |  |  |
| Apbb3 | 0.106475898 | 0.610363131 | 0.842510679 |  |  |  |  |  |  |  |  |
| Apc | -0.09517178 | 0.165924592 | 0.451061536 | SYN |  | ASD |  | FMRP |  |  |  |
| Apc2 | 0.054678012 | 0.458836781 | 0.754418091 |  |  |  |  | FMRP |  |  |  |
| Apcdd1 | 0.983013659 | 0.026354504 | 0.125005428 |  |  |  |  |  |  |  |  |
| Aph1a | 0.104115118 | 0.326668283 | 0.643970891 |  |  | ASD | ASD_sc |  |  | SZ_108 | SZ_full |
| Aph1b | 0.254015346 | 0.004810365 | 0.032820759 |  |  |  |  |  |  |  |  |
| Apip | -0.024051883 | 0.697732361 | 0.886511738 |  |  |  |  |  |  |  |  |
| Aplp1 | 0.018835057 | 0.618250249 | 0.847941692 |  |  |  |  | FMRP |  |  |  |
| Aplp2 | -0.140608699 | 0.126610576 | 0.38477273 |  |  |  |  |  |  |  |  |
| Apmap | 0.005529077 | 0.885348613 | 0.962224578 |  |  |  |  |  |  |  |  |
| Apod | 0.38665924 | 6.20931E-05 | 0.000750386 | SYN |  |  |  |  |  |  |  |
| Apoe | 0.137443948 | 0.795942488 | 0.933720314 | SYN |  |  |  | FMRP | SZdb |  | SZ_full |
| Apol6 | 0.090775683 | 0.839889826 | 0.944846439 |  |  |  |  |  |  |  |  |
| Apol8 | -0.662170131 | 0.013236702 | 0.073520847 | SYN |  |  |  |  |  |  |  |
| Apool | 0.477729293 | 0.145829897 | 0.418821818 | SYN |  |  |  |  |  |  |  |
| Apopt1 | 0.024917323 | 0.833649786 | 0.943106438 |  |  |  |  |  |  | SZ_108 | SZ_full |
| App | -0.21205422 | 0.022011858 | 0.109797736 | SYN |  | ASD |  | FMRP |  |  |  |
| Appbp2 | -0.133559487 | 0.24205575 | 0.557749628 |  |  |  |  |  |  |  |  |
| Appl1 | 0.074086817 | 0.360145877 | 0.677462343 | SYN |  |  |  |  |  |  |  |
| Appl2 | 0.008680369 | 0.808129615 | 0.93712146 | SYN |  |  |  |  |  |  |  |
| Aprt | 0.228833968 | 0.286729705 | 0.604694904 |  |  |  |  |  |  |  |  |
| Aptx | -0.104121083 | 0.246580473 | 0.563531763 |  |  |  |  |  |  |  |  |
| Aqp11 | -0.992250757 | 1.89015E-10 | 5.93537E-09 |  |  |  |  |  |  |  |  |
| Aqp4 | 0.720670624 | 1.40784E-16 | 9.68012E-15 | SYN |  |  |  |  |  |  |  |
| Aqp7 | 0.106810389 | 0.792281943 | 0.932104771 |  |  |  |  |  |  |  |  |
| Ar | -0.137431402 | 0.555519862 | 0.809875054 |  |  | ASD | ASD_sc |  | SZdb |  | SZ_full |
| Araf | 0.116016959 | 0.190810124 | 0.48910346 |  |  |  |  |  |  |  |  |
| Arap1 | -0.039236596 | 0.944432675 | 0.985962641 |  |  |  |  |  |  |  |  |
| Arap2 | -0.177101959 | 0.04522976 | 0.191685742 |  |  |  |  |  |  |  |  |
| Arcn1 | 0.089384983 | 0.187731989 | 0.483929674 | SYN |  |  |  |  |  |  |  |
| Arf1 | 0.196208071 | 0.011195157 | 0.064517757 | SYN |  |  |  |  |  |  |  |
| Arf2 | -0.106933167 | 0.417133341 | 0.724559307 |  |  |  |  |  |  |  |  |
| Arf3 | -0.010130354 | 0.733137477 | 0.905793617 | SYN |  |  |  | FMRP |  |  |  |
| Arf4 | 0.135394347 | 0.107775646 | 0.346900142 |  |  |  |  |  |  |  |  |
| Arf5 | 0.158554247 | 0.076867268 | 0.275859153 | SYN |  |  |  |  |  |  |  |
| Arf6 | -0.056740898 | 0.803768507 | 0.936575254 |  |  |  |  |  |  |  |  |
| Arfgap1 | -0.085565535 | 0.35638971 | 0.675193426 |  |  |  |  |  |  |  |  |
| Arfgap2 | -0.038369363 | 0.757786426 | 0.916884789 | SYN |  |  |  |  |  |  |  |
| Arfgef1 | -0.141505205 | 0.160001583 | 0.441814506 |  |  |  |  | FMRP |  |  |  |
| Arfgef2 | -0.014192144 | 0.805275488 | 0.936945007 | SYN |  |  |  |  |  |  |  |
| Arfip1 | -0.108769964 | 0.294833199 | 0.611588794 |  |  |  |  |  |  |  |  |
| Arfip2 | 0.153943832 | 0.116886667 | 0.364627696 |  |  |  |  |  |  |  |  |
| Arfrp1 | -0.070154351 | 0.446798716 | 0.744686785 |  |  |  |  |  |  |  |  |
| Arglu1 | -0.058043421 | 0.567688572 | 0.818193721 |  |  |  |  |  |  |  |  |
| Arhgap1 | -0.111112202 | 0.388995815 | 0.700368085 | SYN |  |  |  |  |  | SZ_108 | SZ_full |
| Arhgap10 | -0.644584068 | 2.53161E-05 | 0.000334307 |  |  |  |  |  |  |  |  |
| Arhgap12 | -0.398047838 | 0.000678098 | 0.006352987 |  |  |  |  |  |  |  |  |
| Arhgap15 | -0.38572355 | 0.119180749 | 0.369016169 |  |  | ASD |  |  |  |  |  |
| Arhgap17 | 0.017879995 | 0.847857628 | 0.947466244 |  |  |  |  |  |  |  |  |
| Arhgap18 | -0.064105876 | 0.791805361 | 0.931893103 |  |  |  |  |  |  |  |  |
| Arhgap20 | -0.462593109 | 1.92764E-06 | 3.15773E-05 |  |  |  |  | FMRP |  |  |  |
| Arhgap21 | 0.053590716 | 0.540280746 | 0.802535845 | SYN |  |  |  | FMRP |  |  |  |
| Arhgap23 | 0.201666834 | 0.010452184 | 0.061344091 | SYN |  |  |  |  |  |  |  |
| Arhgap24 | -0.039527342 | 0.975716405 | 0.99211875 |  |  | ASD |  |  |  |  |  |
| Arhgap25 | 0.770004156 | 1.96303E-08 | 4.71286E-07 |  |  |  |  |  |  |  |  |
| Arhgap26 | -0.227158065 | 0.009333865 | 0.056059415 | SYN |  |  |  |  |  |  |  |
| Arhgap31 | 0.633639568 | 3.07469E-06 | 4.85618E-05 |  |  |  |  |  |  |  |  |
| Arhgap32 | -0.378230178 | 0.001689539 | 0.014066558 |  |  |  |  |  |  |  |  |
| Arhgap39 | 0.062304121 | 0.531142596 | 0.796763842 |  |  |  |  |  |  |  |  |
| Arhgap42 | -1.807204579 | 4.52568E-27 | 5.82207E-25 |  |  |  |  |  |  |  |  |
| Arhgap44 | 0.347106073 | 2.2198E-05 | 0.000294594 |  |  |  |  |  |  |  |  |
| Arhgap5 | -0.19034693 | 0.09483244 | 0.318209315 |  |  |  |  |  |  |  |  |
| Arhgdia | 0.236699643 | 0.010808013 | 0.062785662 | SYN |  |  |  |  |  |  |  |
| Arhgef10 | 0.255060032 | 0.129834861 | 0.389873367 |  |  |  |  |  | SZdb |  | SZ_full |
| Arhgef10l | -0.260962084 | 0.003623278 | 0.025965198 |  |  |  |  |  |  |  |  |
| Arhgef11 | -0.184944628 | 0.045257685 | 0.191702228 |  |  |  |  | FMRP |  |  |  |
| Arhgef12 | -0.179560945 | 0.052079073 | 0.211390682 |  |  |  |  | FMRP |  |  |  |
| Arhgef15 | -0.186301006 | 0.494036458 | 0.77668892 |  |  |  |  |  |  |  |  |
| Arhgef17 | -0.116521364 | 0.263439605 | 0.579755151 |  |  |  |  | FMRP |  |  |  |
| Arhgef18 | 0.113877713 | 0.52002171 | 0.790843055 |  |  |  |  |  |  |  |  |
| Arhgef19 | 0.204269841 | 0.097802131 | 0.325045671 |  |  |  |  |  |  |  |  |
| Arhgef2 | -0.031595511 | 0.675986329 | 0.878273493 | SYN |  |  |  | FMRP |  |  |  |
| Arhgef25 | 0.654334578 | 2.80691E-14 | 1.44438E-12 |  |  |  |  |  |  |  |  |
| Arhgef26 | 0.611331888 | 1.44446E-08 | 3.54492E-07 |  |  |  |  |  |  |  |  |
| Arhgef28 | 0.608943087 | 1.16913E-09 | 3.33036E-08 |  |  |  |  |  |  |  |  |
| Arhgef3 | 0.47711391 | 1.90826E-06 | 3.1382E-05 |  |  |  |  |  |  |  |  |
| Arhgef4 | 0.141810379 | 0.071344569 | 0.261629557 |  |  |  |  | FMRP |  |  |  |
| Arhgef40 | -0.014989995 | 0.907974005 | 0.97125661 |  |  |  |  |  |  |  |  |
| Arhgef7 | 0.125345137 | 0.123200856 | 0.378378908 | SYN | ID |  |  | FMRP |  |  |  |
| Arhgef9 | -0.041523878 | 0.687532758 | 0.882484918 | SYN | ID |  |  |  |  |  |  |
| Arid1a | -0.090557612 | 0.373124901 | 0.688992138 |  |  |  |  | FMRP |  |  |  |
| Arid2 | -0.154375143 | 0.169106115 | 0.457040044 |  |  |  |  | FMRP |  |  |  |
| Arid4a | 0.153580225 | 0.435715908 | 0.73675431 |  |  |  |  |  |  |  |  |
| Arid4b | -0.017315015 | 0.88836067 | 0.962995126 |  |  |  |  |  |  |  |  |
| Arid5b | 0.058800481 | 0.718429195 | 0.897786272 |  |  |  |  |  |  |  |  |
| Arih1 | 0.055692913 | 0.640952013 | 0.860142008 |  |  |  |  |  |  |  |  |
| Arih2 | -0.037936909 | 0.749575251 | 0.914065719 |  |  |  |  |  |  |  |  |
| Arl1 | -0.014824847 | 0.767429046 | 0.920286747 |  |  |  |  |  |  |  |  |
| Arl13b | -0.103074654 | 0.442025684 | 0.741213325 |  |  |  |  |  |  |  |  |
| Arl14ep | 0.106664005 | 0.259603924 | 0.576917826 |  |  |  |  |  |  |  |  |
| Arl15 | 0.44601051 | 1.33484E-06 | 2.30948E-05 |  |  |  |  |  |  |  |  |
| Arl2 | 0.297459778 | 0.081053216 | 0.286140772 |  |  |  |  |  |  |  |  |
| Arl2bp | 0.12205877 | 0.210754925 | 0.518399997 | SYN |  |  |  |  |  |  |  |
| Arl3 | 0.103859695 | 0.494413022 | 0.77668892 |  |  |  |  |  |  | SZ_108 | SZ_full |
| Arl4c | -0.369938958 | 0.000141733 | 0.001570086 |  |  |  |  |  |  |  |  |
| Arl5a | -0.075720357 | 0.68654484 | 0.882339565 |  |  |  |  |  |  |  |  |
| Arl6 | 0.043592233 | 0.509192821 | 0.785859508 |  |  |  |  |  |  |  |  |
| Arl6ip1 | 0.072958524 | 0.257971047 | 0.575546034 |  |  |  |  |  |  |  |  |
| Arl6ip5 | 0.206622701 | 0.022719062 | 0.112272143 |  |  |  |  |  |  |  |  |
| Arl8a | 0.084489993 | 0.257686545 | 0.575233104 |  |  |  |  |  |  |  |  |
| Arl8b | 0.125479569 | 0.051237328 | 0.208824185 |  |  |  |  |  |  |  |  |
| Armc1 | -0.054786947 | 0.958206273 | 0.989551899 |  |  |  |  |  |  |  |  |
| Armc10 | -0.050990381 | 0.733193987 | 0.905793617 | SYN |  |  |  |  |  |  |  |
| Armc2 | -0.75391283 | 3.04051E-08 | 7.04975E-07 |  |  |  |  |  |  |  |  |
| Armc5 | 0.034558046 | 0.919586729 | 0.976907798 |  |  |  |  |  |  |  |  |
| Armc8 | -0.026990287 | 0.538431651 | 0.801497271 |  |  |  |  |  |  |  |  |
| Armc9 | 0.151441805 | 0.157690936 | 0.437475792 |  |  |  |  |  |  |  |  |
| Armcx1 | 0.160527087 | 0.241127833 | 0.557136615 |  |  |  |  |  |  |  |  |
| Armcx2 | -0.06101306 | 0.543826319 | 0.804294219 |  |  |  |  |  |  |  |  |
| Armcx3 | 0.054195402 | 0.418348667 | 0.725853593 |  |  |  |  |  |  |  |  |
| Arnt | 0.115422204 | 0.4742055 | 0.76408458 | SYN |  |  |  |  |  |  |  |
| Arnt2 | -0.609097453 | 5.01313E-12 | 1.94101E-10 |  |  | ASD | ASD_sc | FMRP |  |  |  |
| Arntl | -0.473832697 | 1.55169E-06 | 2.6221E-05 |  |  |  |  |  |  |  |  |
| Arpc1a | 0.15211317 | 0.072838257 | 0.265156522 | SYN |  |  |  |  |  |  |  |
| Arpc2 | 0.300265142 | 0.036855804 | 0.163130905 | SYN |  |  |  |  |  |  |  |
| Arpc3 | 0.043718744 | 0.841942957 | 0.945289559 | SYN |  |  |  |  |  |  |  |
| Arpc4 | 0.246488235 | 0.011774204 | 0.066997433 | SYN |  |  |  |  |  |  |  |
| Arpc5 | 0.606358285 | 4.09137E-11 | 1.42501E-09 |  |  |  |  |  |  |  |  |
| Arpc5l | -0.087636971 | 0.403630326 | 0.712938716 | SYN |  |  |  |  |  |  |  |
| Arpp19 | -0.455271905 | 5.20346E-07 | 9.65181E-06 |  |  |  |  |  |  |  |  |
| Arpp21 | -0.317939298 | 0.000346791 | 0.003492434 |  |  |  |  | FMRP |  |  |  |
| Arrb1 | -0.040842405 | 0.758530175 | 0.917637037 | SYN |  |  |  | FMRP |  |  |  |
| Arrdc1 | -0.574673709 | 0.003841274 | 0.027161348 |  |  |  |  |  |  |  |  |
| Arrdc3 | 0.399177891 | 0.003771704 | 0.026883926 |  |  |  |  |  |  |  |  |
| Arsa | -0.074835544 | 0.58630217 | 0.827964962 |  | ID |  |  |  |  |  |  |
| Arsb | -0.207795042 | 0.041346842 | 0.179034969 |  |  |  |  |  |  |  |  |
| Arsg | 0.053323651 | 0.974361187 | 0.991895958 |  |  |  |  |  |  |  |  |
| Arsk | -0.01249499 | 0.813115267 | 0.938538051 |  |  |  |  |  |  |  |  |
| Arvcf | -0.032886138 | 0.749849616 | 0.914068732 | SYN |  |  |  | FMRP | SZdb |  | SZ_full |
| Arx | -0.114915916 | 0.469283445 | 0.762246238 |  | ID | ASD | ASD_sc |  |  |  |  |
| Arxes1 | -0.109237838 | 0.487530221 | 0.771851216 |  |  |  |  |  |  |  |  |
| Asah1 | 0.003771281 | 0.905135981 | 0.970378259 |  | ID |  |  |  |  |  |  |
| Asap1 | -0.28758192 | 0.000356013 | 0.003580783 |  |  |  |  |  |  |  |  |
| Asap2 | 0.635553202 | 4.31628E-06 | 6.68479E-05 |  |  |  |  |  |  |  |  |
| Asb13 | -0.096379918 | 0.276048177 | 0.593626385 |  |  |  |  |  |  |  |  |
| Asb8 | -0.038689221 | 0.526904307 | 0.794238803 |  |  |  |  |  |  |  |  |
| Ascc2 | 0.1611678 | 0.506655394 | 0.784399099 |  |  |  |  |  |  |  |  |
| Ascc3 | -0.107133323 | 0.4490686 | 0.746235921 |  |  |  |  |  |  |  |  |
| Ash1l | -0.08600249 | 0.376813083 | 0.692315926 |  |  | ASD | ASD_sc | FMRP |  |  |  |
| Ash2l | 0.107593984 | 0.307840125 | 0.624820652 |  |  |  |  |  |  |  |  |
| Asic1 | -0.025374991 | 0.692843892 | 0.884375762 |  |  |  |  |  |  |  |  |
| Asic2 | 0.037882309 | 0.808805223 | 0.93712146 |  |  |  |  |  |  |  |  |
| Asl | -0.099984686 | 0.51439374 | 0.787995408 |  | ID |  |  |  |  |  |  |
| Asna1 | 0.100712399 | 0.312687286 | 0.630022965 |  |  |  |  |  |  |  |  |
| Asns | -0.03906667 | 0.861291825 | 0.953060987 |  |  |  |  |  |  |  |  |
| Asnsd1 | 0.026191103 | 0.942448058 | 0.985900058 |  |  |  |  |  |  |  |  |
| Aspa | 0.202879918 | 0.320932924 | 0.637688241 |  | ID |  |  |  |  |  |  |
| Asph | -0.30145005 | 0.000949768 | 0.008521203 |  |  |  |  |  |  |  |  |
| Asphd2 | 0.12873572 | 0.186961376 | 0.482902829 |  |  |  |  |  |  |  |  |
| Aspscr1 | -0.031727331 | 0.557149619 | 0.810374491 |  |  |  |  |  |  |  |  |
| Asrgl1 | 0.001864596 | 0.89032692 | 0.963534262 |  |  |  |  |  |  |  |  |
| Ass1 | 0.623633522 | 1.3613E-07 | 2.79033E-06 |  | ID | ASD |  |  |  |  |  |
| Astn1 | -0.104128236 | 0.337959533 | 0.659329719 | SYN |  |  |  |  |  |  |  |
| Astn2 | 0.185422357 | 0.374719434 | 0.69008594 | SYN |  | ASD | ASD_sc |  |  |  |  |
| Asun | -0.211830669 | 0.02528986 | 0.121733206 |  |  |  |  |  |  |  |  |
| Asxl1 | -0.139758957 | 0.14079769 | 0.410304121 |  |  |  |  |  |  |  |  |
| Asxl2 | 0.122017069 | 0.264725583 | 0.58143683 |  |  |  |  |  |  |  |  |
| Atad1 | -0.099038754 | 0.455995617 | 0.752289793 | SYN |  |  |  |  |  |  |  |
| Atad2 | -0.257175936 | 0.009865671 | 0.058635314 |  |  |  |  |  |  |  |  |
| Atad2b | 0.066195117 | 0.59389818 | 0.833255969 |  |  |  |  |  |  |  |  |
| Atad3a | 0.126298742 | 0.388833325 | 0.700233597 | SYN |  |  |  |  |  |  |  |
| Atat1 | 0.141421104 | 0.130796584 | 0.391604187 |  |  |  |  |  |  |  |  |
| Atcay | 0.060210886 | 0.452445684 | 0.749471813 |  |  |  |  |  |  |  |  |
| Ate1 | -0.160351435 | 0.165090381 | 0.449867057 |  |  |  |  |  |  |  |  |
| Atf2 | -0.105250045 | 0.294250475 | 0.611182758 |  |  |  |  |  |  |  |  |
| Atf4 | -0.03127234 | 0.693622839 | 0.884890557 |  |  |  |  |  |  |  |  |
| Atf6 | -0.30433573 | 0.002521813 | 0.019509196 |  |  |  |  |  |  |  |  |
| Atf6b | 0.25357462 | 0.010226644 | 0.060331151 |  |  |  |  |  | SZdb |  | SZ_full |
| Atg12 | -0.011553832 | 0.800973838 | 0.935166555 |  |  |  |  |  |  |  |  |
| Atg13 | -0.119614278 | 0.21326704 | 0.520155963 |  |  |  |  |  |  | SZ_108 | SZ_full |
| Atg14 | -0.049562407 | 0.662642735 | 0.87215156 |  |  |  |  |  |  |  |  |
| Atg16l1 | 0.018378348 | 0.863201764 | 0.953809724 | SYN |  |  |  |  |  |  |  |
| Atg16l2 | -0.212825767 | 0.064200384 | 0.243886832 |  |  |  |  |  |  |  |  |
| Atg2a | -0.028386436 | 0.971769701 | 0.991571804 |  |  |  |  | FMRP |  |  |  |
| Atg2b | 0.054851629 | 0.550962843 | 0.807666914 |  |  |  |  | FMRP |  |  |  |
| Atg4b | 0.01398098 | 0.947901278 | 0.986605625 |  |  |  |  |  |  |  |  |
| Atg4c | -0.206960166 | 0.012870521 | 0.071837144 |  |  |  |  |  |  |  |  |
| Atg4d | 0.014309319 | 0.969616936 | 0.991454046 |  |  |  |  |  |  |  |  |
| Atg7 | -0.121662477 | 0.360409313 | 0.677657869 |  |  | ASD |  |  |  |  |  |
| Atg9a | 0.011002429 | 0.862035353 | 0.953354683 |  |  |  |  | FMRP |  |  |  |
| Atic | 0.214586787 | 0.038824765 | 0.170146335 | SYN |  |  |  |  |  |  |  |
| Atl1 | 0.060677864 | 0.37318116 | 0.688992138 | SYN |  |  |  |  |  |  |  |
| Atl2 | -0.090206498 | 0.439979485 | 0.739573524 | SYN |  |  |  |  |  |  |  |
| Atl3 | -0.09127449 | 0.32397879 | 0.64136382 |  |  |  |  |  |  |  |  |
| Atm | 0.118920083 | 0.367942456 | 0.684543508 |  |  |  |  |  |  |  |  |
| Atmin | -0.182379923 | 0.434020968 | 0.735135112 |  |  |  |  | FMRP |  |  |  |
| Atn1 | 0.031267442 | 0.759975619 | 0.918279887 |  | ID |  |  | FMRP | SZdb |  | SZ_full |
| Atp11a | 0.042757815 | 0.529826904 | 0.795987829 |  |  |  |  |  |  |  |  |
| Atp11b | -0.419468964 | 7.85608E-07 | 1.41765E-05 |  |  |  |  |  |  |  |  |
| Atp11c | 0.047523069 | 0.642496565 | 0.860835311 |  |  |  |  |  |  |  |  |
| Atp13a1 | 0.087392087 | 0.479161516 | 0.765984483 | SYN |  |  |  |  |  |  |  |
| Atp13a2 | 0.04794479 | 0.59986315 | 0.835576054 |  |  |  |  | FMRP |  |  |  |
| Atp13a3 | 0.005901938 | 0.625056355 | 0.850614729 |  |  |  |  |  |  |  |  |
| Atp13a5 | 0.036096911 | 0.844817244 | 0.945963967 |  |  |  |  |  |  |  |  |
| Atp1a1 | -1.050462152 | 3.70747E-28 | 4.84767E-26 | SYN |  |  |  | FMRP |  |  |  |
| Atp1a2 | -0.081340871 | 0.320088954 | 0.637688241 | SYN |  |  |  | FMRP |  |  |  |
| Atp1a3 | 0.45551879 | 3.13834E-09 | 8.3717E-08 | SYN |  |  |  | FMRP |  |  |  |
| Atp1a4 | -0.045837436 | 0.692984316 | 0.884375762 | SYN |  |  |  |  |  |  |  |
| Atp1b1 | 0.1745397 | 0.089602776 | 0.30554585 | SYN |  |  |  | FMRP |  |  |  |
| Atp1b2 | -0.104540528 | 0.202803498 | 0.506754605 | SYN |  |  |  |  |  |  |  |
| Atp1b3 | -0.120617214 | 0.127280563 | 0.385857003 |  |  |  |  |  |  |  |  |
| Atp2a2 | -0.093227493 | 0.411159416 | 0.720088075 | SYN | ID |  |  | FMRP |  | SZ_108 | SZ_full |
| Atp2b1 | 0.27631276 | 0.000166202 | 0.001803631 | SYN |  |  |  |  |  |  |  |
| Atp2b2 | -0.425195866 | 8.42901E-08 | 1.78803E-06 | SYN |  | ASD | ASD_sc | FMRP |  |  |  |
| Atp2b3 | 0.196171281 | 0.021695815 | 0.10859466 | SYN |  |  |  |  |  |  |  |
| Atp2b4 | -0.326171239 | 0.401665585 | 0.711185063 | SYN |  |  |  | FMRP |  |  |  |
| Atp2c1 | 0.113796406 | 0.095819511 | 0.320335566 |  |  |  |  |  |  |  |  |
| Atp5a1 | 0.121163169 | 0.101850306 | 0.333631962 | SYN |  |  |  | FMRP |  |  |  |
| Atp5b | 0.005647257 | 0.770747104 | 0.921167131 | SYN |  |  |  | FMRP |  |  |  |
| Atp5c1 | 0.093951579 | 0.269080776 | 0.587353112 | SYN |  |  |  |  |  |  |  |
| Atp5e | -0.18628776 | 0.525597189 | 0.793545744 |  |  |  |  |  |  |  |  |
| Atp5f1 | -0.035036057 | 0.778437983 | 0.925308696 | SYN |  |  |  |  |  |  |  |
| Atp5g2 | 0.099030626 | 0.65266226 | 0.868058719 |  |  |  |  |  |  |  |  |
| Atp5g3 | -0.077068665 | 0.380532583 | 0.695268093 |  |  |  |  |  |  |  |  |
| Atp5h | 0.22146662 | 0.255403742 | 0.572540822 | SYN |  |  |  |  |  |  |  |
| Atp5j | -0.00055065 | 0.659609239 | 0.87047969 | SYN |  |  |  |  |  |  |  |
| Atp5j2 | -0.003846991 | 0.724453169 | 0.899896975 | SYN |  |  |  |  |  |  |  |
| Atp5k | -0.041722449 | 0.61816195 | 0.847941692 | SYN |  |  |  |  |  |  |  |
| Atp5l | 0.089842213 | 0.320587171 | 0.637688241 | SYN |  |  |  |  |  |  |  |
| Atp5o | 0.245270562 | 0.544111164 | 0.804417173 | SYN |  |  |  |  |  |  |  |
| Atp5s | -0.009569175 | 0.957279076 | 0.989355248 |  |  |  |  |  |  |  |  |
| Atp5sl | -0.101060497 | 0.332132845 | 0.651683044 |  |  |  |  |  |  |  |  |
| Atp6ap1 | 0.054755221 | 0.387437579 | 0.699153451 |  |  |  |  |  |  |  |  |
| Atp6ap2 | -0.152272102 | 0.100981354 | 0.332134962 |  | ID |  |  |  |  |  |  |
| Atp6v0a1 | -0.021728098 | 0.893947166 | 0.965225747 | SYN |  |  |  | FMRP |  |  |  |
| Atp6v0a2 | 0.011592854 | 0.995105649 | 1 |  |  |  |  |  |  |  |  |
| Atp6v0b | 0.014705529 | 0.964182367 | 0.990416798 |  |  |  |  |  |  |  |  |
| Atp6v0c | -0.000497527 | 0.872790255 | 0.957631828 | SYN |  |  |  |  |  |  |  |
| Atp6v0d1 | 0.056925005 | 0.562729523 | 0.815184571 | SYN |  |  |  | FMRP |  |  |  |
| Atp6v0e | -0.093219573 | 0.188060792 | 0.484330925 |  |  |  |  |  |  |  |  |
| Atp6v0e2 | -0.038396878 | 0.659955639 | 0.870542186 |  |  |  |  |  |  |  |  |
| Atp6v1a | 0.033642835 | 0.428751459 | 0.73153195 | SYN |  |  |  |  |  |  |  |
| Atp6v1b2 | -0.062575733 | 0.745408958 | 0.911928524 | SYN |  |  |  | FMRP |  |  |  |
| Atp6v1c1 | -0.099280152 | 0.574243478 | 0.821203351 | SYN |  |  |  |  |  |  |  |
| Atp6v1d | 0.033977424 | 0.685161245 | 0.881864748 | SYN |  |  |  |  |  |  |  |
| Atp6v1e1 | 0.036244814 | 0.680240706 | 0.878917067 | SYN |  |  |  |  |  |  |  |
| Atp6v1f | -0.023258717 | 0.655820021 | 0.86959601 | SYN |  |  |  |  |  |  |  |
| Atp6v1g2 | 0.138688446 | 0.05335545 | 0.21503945 | SYN |  |  |  |  |  |  |  |
| Atp6v1h | -0.155920804 | 0.077668781 | 0.277671984 | SYN |  |  |  |  |  |  |  |
| Atp8a1 | -0.085919293 | 0.601478969 | 0.836949801 | SYN |  |  |  |  |  |  |  |
| Atp8a2 | -0.336084031 | 0.005862305 | 0.038452094 | SYN |  |  |  |  |  |  |  |
| Atp8b2 | -0.445029198 | 0.000146788 | 0.001612649 |  |  |  |  |  |  |  |  |
| Atp9a | -0.182039677 | 0.027115525 | 0.128048212 |  |  |  |  | FMRP |  |  |  |
| Atp9b | -0.237814875 | 0.003654188 | 0.026116314 |  |  |  |  |  |  |  |  |
| Atpaf2 | -0.047586671 | 0.689294354 | 0.883466458 |  |  |  |  |  |  | SZ_108 | SZ_full |
| Atpbd4 | 0.114311861 | 0.496114413 | 0.77686598 |  |  |  |  |  |  |  |  |
| Atpif1 | 0.062932789 | 0.969791353 | 0.991454046 | SYN |  |  |  |  |  |  |  |
| Atr | -0.086116445 | 0.429268169 | 0.73153195 |  | ID |  |  |  |  |  |  |
| Atraid | 0.199309865 | 0.101904057 | 0.333656306 |  |  |  |  |  |  |  |  |
| Atrn | -0.097401662 | 0.310187501 | 0.627296021 |  |  |  |  |  |  |  |  |
| Atrnl1 | -0.261606568 | 0.003210745 | 0.023573889 |  |  | ASD |  |  |  |  |  |
| Atrx | 0.090657416 | 0.185510467 | 0.48118097 | SYN | ID | ASD |  |  |  |  |  |
| Atxn10 | 0.250593054 | 0.00128508 | 0.011092858 | SYN |  |  |  |  |  |  |  |
| Atxn1l | -0.109295825 | 0.430400552 | 0.731800215 |  |  |  |  |  |  |  |  |
| Atxn2 | -0.042981835 | 0.367159649 | 0.684061051 |  |  |  |  |  |  |  |  |
| Atxn2l | 0.160342746 | 0.116289525 | 0.363735394 |  |  |  |  |  |  |  |  |
| Atxn3 | 0.034410454 | 0.833700858 | 0.943106438 |  |  |  |  |  | SZdb |  | SZ_full |
| Atxn7 | 0.035965633 | 0.93204908 | 0.98255663 |  |  | ASD |  |  |  | SZ_108 | SZ_full |
| Atxn7l1 | -0.45692483 | 0.000566383 | 0.005410143 |  |  |  |  |  |  |  |  |
| Atxn7l3 | -0.153475836 | 0.062702037 | 0.240553845 |  |  |  |  |  |  |  |  |
| Atxn7l3b | -0.025703165 | 1 | 1 |  |  |  |  |  |  |  |  |
| AU022252 | 0.174600252 | 0.242654853 | 0.558239142 |  |  |  |  |  |  |  |  |
| AU040320 | -0.221256749 | 0.019283997 | 0.099040024 |  |  |  |  |  |  |  |  |
| Auh | 0.099907332 | 0.452243183 | 0.749471134 | SYN |  |  |  |  |  |  |  |
| Auts2 | 0.002257575 | 0.809429724 | 0.93712146 |  |  | ASD | ASD_sc | FMRP |  |  |  |
| Avl9 | -0.08907381 | 0.513256914 | 0.787863192 |  |  |  |  |  |  |  |  |
| AW146154 | -0.131612348 | 0.494253285 | 0.77668892 |  |  |  |  |  |  |  |  |
| AW549877 | -0.115917508 | 0.290271718 | 0.607535998 |  |  |  |  |  |  |  |  |
| AW554918 | -0.271895988 | 0.012657324 | 0.070895235 |  |  |  |  |  |  |  |  |
| Axin1 | 0.093081326 | 0.517589591 | 0.789650837 |  |  |  |  |  |  |  |  |
| Axl | 0.003976593 | 0.711382627 | 0.893500957 |  |  |  |  |  |  |  |  |
| Azi1 | 0.106419974 | 0.503343516 | 0.782106171 |  |  |  |  |  |  |  |  |
| Azi2 | -0.009729967 | 0.800468267 | 0.935166555 | SYN |  |  |  |  |  |  |  |
| Azin1 | 0.017249658 | 0.879700645 | 0.959752724 |  |  |  |  |  |  |  |  |
| B230120H23Rik | -0.3548464 | 0.010313817 | 0.060667609 |  |  |  |  |  |  |  |  |
| B230217C12Rik | 0.040217349 | 0.631473621 | 0.854727738 |  |  |  |  |  |  |  |  |
| B230219D22Rik | 0.050468468 | 0.362772614 | 0.679847949 |  |  |  |  |  |  |  |  |
| B2m | -0.201317197 | 0.181729072 | 0.474769432 |  |  |  |  |  |  |  |  |
| B3galnt2 | 0.056194712 | 0.776352793 | 0.924077297 |  |  |  |  |  |  |  |  |
| B3galt1 | -0.12444019 | 0.316379261 | 0.633711949 |  |  |  |  |  |  |  |  |
| B3galt2 | 0.960283089 | 2.98298E-11 | 1.07172E-09 |  |  |  |  |  |  |  |  |
| B3galt5 | 0.007556172 | 0.677445232 | 0.878273493 |  |  |  |  |  |  |  |  |
| B3galt6 | -0.064518462 | 0.947483916 | 0.986441942 |  |  |  |  |  |  |  |  |
| B3gat1 | -0.311863661 | 0.000769631 | 0.007055835 |  |  |  |  | FMRP |  |  |  |
| B3gat3 | 0.111565786 | 0.426019338 | 0.73059413 | SYN |  |  |  |  |  |  |  |
| B3gnt1 | 0.291127671 | 0.010831379 | 0.062875601 |  |  |  |  |  |  |  |  |
| B4galnt1 | 0.508635303 | 1.50265E-07 | 3.05742E-06 |  |  |  |  |  |  |  |  |
| B4galnt2 | 0.018915677 | 0.891622487 | 0.964133804 |  |  |  |  |  |  |  |  |
| B4galnt4 | -0.073642751 | 0.369885552 | 0.685616352 |  |  |  |  |  |  |  |  |
| B4galt1 | -0.335700069 | 0.174606029 | 0.463949163 |  | ID |  |  |  |  |  |  |
| B4galt2 | -0.520487948 | 7.62198E-08 | 1.63862E-06 |  |  |  |  |  |  |  |  |
| B4galt3 | 0.004490152 | 1 | 1 |  |  |  |  |  |  |  |  |
| B4galt5 | -0.082301755 | 0.406548645 | 0.716762155 |  |  |  |  |  |  |  |  |
| B4galt6 | 0.044229422 | 0.374361273 | 0.689744863 |  |  |  |  |  |  |  |  |
| B4galt7 | -0.126226568 | 0.401526187 | 0.711185063 |  | ID |  |  |  |  |  |  |
| B630005N14Rik | 0.071546261 | 0.414220523 | 0.722937176 |  |  |  |  |  |  |  |  |
| Baalc | -0.584998255 | 4.60207E-07 | 8.59921E-06 |  |  |  |  |  |  |  |  |
| Babam1 | 0.055769173 | 0.440232103 | 0.739687453 |  |  |  |  |  |  |  |  |
| Bace1 | 0.533870196 | 1.65981E-10 | 5.38156E-09 |  |  |  |  |  |  |  |  |
| Bach2 | -0.571939209 | 9.97454E-06 | 0.000143864 |  |  |  |  |  |  |  |  |
| Bag1 | -0.064495226 | 0.468519143 | 0.76189972 |  |  |  |  |  |  |  |  |
| Bag4 | -0.001555807 | 0.748642043 | 0.913581539 |  |  |  |  |  |  |  |  |
| Bag5 | -0.105622874 | 0.458234056 | 0.754249674 | SYN |  |  |  |  |  | SZ_108 | SZ_full |
| Bag6 | -0.006039616 | 0.825727908 | 0.941950287 |  |  |  |  |  |  |  |  |
| Bai1 | -0.149250101 | 0.070367327 | 0.259837869 | SYN |  |  |  | FMRP |  |  |  |
| Bai2 | 0.102904186 | 0.134243493 | 0.398335604 |  |  |  |  | FMRP |  |  |  |
| Bai3 | 0.192814941 | 0.051013381 | 0.208249779 | SYN |  |  |  |  |  |  |  |
| Baiap2 | 0.226686352 | 0.01796195 | 0.093636938 | SYN |  | ASD |  |  |  |  |  |
| Banf1 | 0.166906545 | 0.775960727 | 0.924077297 | SYN |  |  |  |  |  |  |  |
| Banp | -0.037467101 | 0.491238585 | 0.773853814 |  |  |  |  |  |  |  |  |
| Bap1 | -0.192908082 | 0.057534702 | 0.225946225 |  |  |  |  | FMRP |  |  |  |
| Basp1 | 0.327688727 | 0.000106207 | 0.001211883 | SYN |  |  |  |  |  |  |  |
| Bax | 0.171335551 | 0.370739446 | 0.686720348 |  |  |  |  |  |  |  |  |
| Baz1b | 0.036142386 | 0.821965491 | 0.941239661 | SYN |  |  |  |  |  |  |  |
| Baz2a | 0.071161575 | 0.750767717 | 0.914217299 |  |  |  |  | FMRP |  |  |  |
| Baz2b | -0.119042693 | 0.201566304 | 0.505123411 |  |  |  |  |  |  |  |  |
| Bbs1 | 0.084146122 | 0.382138475 | 0.695875907 |  | ID |  |  |  |  |  |  |
| Bbs2 | -0.092986794 | 0.196154794 | 0.497784221 |  | ID |  |  |  |  |  |  |
| Bbs4 | -0.131371451 | 0.252867109 | 0.570531033 |  | ID | ASD |  |  |  |  |  |
| Bbs5 | 0.015562402 | 0.893691301 | 0.965225747 |  |  |  |  |  |  |  |  |
| Bbs9 | -0.368071707 | 0.005123035 | 0.034511257 |  |  |  |  |  |  |  |  |
| Bbx | 0.049459596 | 0.549969348 | 0.80739104 |  |  |  |  |  |  |  |  |
| BC003331 | 0.092462584 | 0.395372167 | 0.706399122 |  |  |  |  |  |  |  |  |
| BC004004 | -0.193814041 | 0.082123097 | 0.289317058 |  |  |  |  |  |  |  |  |
| BC005537 | 0.13424391 | 0.095027364 | 0.318461451 |  |  |  |  |  |  |  |  |
| BC005561 | -0.049855232 | 0.401946149 | 0.711185063 |  | ID |  |  |  |  |  |  |
| BC005624 | 0.005391527 | 0.880690099 | 0.960139999 |  |  |  |  |  |  |  |  |
| BC006965 | -1.183925839 | 2.43693E-06 | 3.92665E-05 |  |  |  |  |  |  |  |  |
| BC017158 | -0.411641413 | 0.001897454 | 0.015522151 |  |  |  |  |  |  |  |  |
| BC018242 | 0.462842203 | 0.003598255 | 0.025809063 |  |  |  |  |  |  |  |  |
| BC018507 | -0.209991777 | 0.018014837 | 0.093851299 |  |  |  |  | FMRP |  |  |  |
| BC023829 | 0.130984943 | 0.199397999 | 0.501861293 |  |  |  |  |  |  |  |  |
| BC026590 | -0.021305049 | 0.973109371 | 0.99178614 |  |  |  |  |  |  |  |  |
| BC029722 | 0.203704068 | 0.088624265 | 0.303116267 |  |  |  |  |  |  |  |  |
| BC030336 | -0.081887882 | 0.548281764 | 0.806991235 |  |  |  |  |  |  |  |  |
| BC031181 | 0.047289863 | 0.443282989 | 0.742141894 |  |  |  |  |  |  |  |  |
| BC037034 | 0.102936855 | 0.352983387 | 0.672092502 |  |  |  |  |  |  |  |  |
| BC048644 | 0.037362078 | 0.923244022 | 0.978235671 |  |  |  |  |  |  |  |  |
| BC056474 | 0.078853136 | 0.819240727 | 0.941021502 |  |  |  |  |  |  |  |  |
| BC068157 | 0.215444385 | 0.086844776 | 0.300378984 |  |  |  |  |  |  |  |  |
| BC068281 | 0.290270751 | 0.095089308 | 0.318535203 |  |  |  |  |  |  |  |  |
| Bcan | 0.107728924 | 0.281291804 | 0.599155737 | SYN |  |  |  | FMRP |  |  |  |
| Bcap31 | 0.05886854 | 0.580009586 | 0.82433294 |  |  |  |  |  |  |  |  |
| Bcar1 | 0.107073889 | 0.213501965 | 0.520155963 |  |  |  |  |  |  |  |  |
| Bcar3 | -0.423211442 | 0.00321274 | 0.023573889 |  |  |  |  |  |  |  |  |
| Bcas1 | 0.41282104 | 0.008298095 | 0.05122725 | SYN |  |  |  |  |  |  |  |
| Bcas2 | 0.064528383 | 0.624015225 | 0.85035801 |  |  |  |  |  |  |  |  |
| Bcas3 | -0.011382563 | 0.930297301 | 0.981434219 |  |  |  |  |  |  |  |  |
| Bcat1 | -0.085084332 | 0.69941932 | 0.887191673 |  |  |  |  |  |  |  |  |
| Bckdha | 0.034097888 | 0.993104358 | 0.999619397 |  | ID |  |  |  |  |  |  |
| Bckdhb | 0.085566101 | 0.344731541 | 0.665113394 |  | ID |  |  |  |  |  |  |
| Bckdk | -0.087973469 | 0.395677355 | 0.706474453 | SYN |  | ASD | ASD_sc |  |  |  |  |
| Bcl11a | 0.302063879 | 0.000447177 | 0.004397879 |  |  | ASD | ASD_sc |  |  |  |  |
| Bcl11b | 0.291044944 | 0.016885254 | 0.089189923 |  |  |  |  |  |  | SZ_108 | SZ_full |
| Bcl2 | -0.251128358 | 0.144567521 | 0.417023707 |  |  | ASD |  |  |  |  |  |
| Bcl2l1 | 0.115382776 | 0.137656583 | 0.405030324 |  |  |  |  |  |  |  |  |
| Bcl2l13 | -0.009598883 | 0.933073065 | 0.983116349 |  |  |  |  |  |  |  |  |
| Bcl2l2 | -0.080244861 | 0.426604758 | 0.730684295 |  |  |  |  |  |  |  |  |
| Bcl7a | -0.111960129 | 0.198558558 | 0.50069651 |  |  |  |  |  |  |  |  |
| Bcl7c | -0.436468803 | 0.002660339 | 0.020396476 |  |  |  |  |  |  |  |  |
| Bcl9 | 0.063931634 | 0.364368672 | 0.681248132 |  |  |  |  |  |  |  |  |
| Bcl9l | 0.156679936 | 0.079779094 | 0.283437884 |  |  |  |  | FMRP |  |  |  |
| Bclaf1 | 0.045713299 | 0.59972536 | 0.835530039 |  |  |  |  |  |  |  |  |
| Bcor | -0.328001289 | 0.00186258 | 0.015294918 |  | ID |  |  |  |  |  |  |
| Bcorl1 | -0.141917133 | 0.340398026 | 0.661070041 |  |  |  |  |  |  |  |  |
| Bcr | 0.118549802 | 0.139480636 | 0.407359045 | SYN |  |  |  | FMRP |  |  |  |
| Bcs1l | -0.119404827 | 0.527843655 | 0.794654775 |  | ID |  |  |  |  |  |  |
| Bdnf | -0.027335057 | 0.919936797 | 0.977078911 |  |  | ASD |  |  | SZdb |  | SZ_full |
| Bean1 | -0.005373446 | 0.758753292 | 0.917637037 |  |  |  |  |  |  |  |  |
| Becn1 | -0.004287133 | 0.783972556 | 0.928282627 |  |  |  |  |  |  |  |  |
| Begain | -0.280406429 | 0.003118258 | 0.022985318 | SYN |  |  |  |  |  |  |  |
| Bend6 | -0.166704775 | 0.019823883 | 0.101077505 |  |  |  |  |  |  |  |  |
| Bet1 | -0.009676812 | 0.847623496 | 0.947399805 |  |  |  |  |  |  |  |  |
| Bet1l | 0.092315158 | 0.565468837 | 0.81693133 |  |  |  |  |  |  |  |  |
| Beta-s | -0.192074765 | 0.145068861 | 0.41798885 |  |  |  |  |  |  |  |  |
| Bfar | 0.008432524 | 0.949684258 | 0.987315126 |  |  |  |  |  |  |  |  |
| Bhlhb9 | 0.024019362 | 0.424409683 | 0.729913577 |  |  |  |  |  |  |  |  |
| Bhlhe22 | -0.651732545 | 0.002941771 | 0.022052226 |  |  |  |  |  |  |  |  |
| Bhlhe40 | 0.216218544 | 0.161831552 | 0.444326492 |  |  |  |  |  |  |  |  |
| Bhlhe41 | 0.736799634 | 6.43434E-07 | 1.17761E-05 |  |  |  |  |  |  |  |  |
| Bicc1 | -0.272120174 | 0.152257592 | 0.428338929 |  |  |  |  |  |  |  |  |
| Bicd1 | 0.103567724 | 0.498943887 | 0.779027036 |  |  |  |  |  |  |  |  |
| Bicd2 | 0.027797506 | 0.742374254 | 0.910888856 |  |  |  |  |  |  |  |  |
| Bin1 | 0.110774104 | 0.385870058 | 0.698524644 | SYN |  | ASD |  |  |  |  |  |
| Bin3 | -0.043034251 | 0.516608733 | 0.789224137 |  |  |  |  |  |  |  |  |
| Birc2 | -0.003325284 | 0.846230274 | 0.946505773 |  |  |  |  |  |  |  |  |
| Birc6 | -0.063393201 | 0.454893926 | 0.751342711 | SYN |  |  |  | FMRP |  |  |  |
| Bivm | 0.027556721 | 0.763974761 | 0.919979858 |  |  |  |  |  |  |  |  |
| Blcap | 0.377765764 | 0.000150299 | 0.001646683 |  |  |  |  |  |  |  |  |
| Blmh | 0.131856797 | 0.110636381 | 0.35255125 |  |  |  |  |  |  |  |  |
| Bloc1s2a | 0.039926011 | 0.955552147 | 0.989355248 |  |  |  |  |  |  |  |  |
| Bloc1s4 | 0.120818327 | 0.402947036 | 0.71214393 |  |  |  |  |  |  |  |  |
| Bloc1s5 | -0.131885911 | 0.401876741 | 0.711185063 |  |  |  |  |  |  |  |  |
| Bloc1s6 | 0.075801726 | 0.727777023 | 0.90219611 |  |  |  |  |  |  |  |  |
| Bmi1 | 0.001378678 | 0.622611684 | 0.849966377 |  |  |  |  |  |  |  |  |
| Bmp1 | -0.756593235 | 4.10313E-06 | 6.36703E-05 |  |  |  |  |  |  |  |  |
| Bmper | -0.013230111 | 0.814903619 | 0.938851837 |  |  |  |  |  |  |  |  |
| Bmpr1a | 0.193060977 | 0.029376728 | 0.136702905 |  |  |  |  |  |  |  |  |
| Bmpr1b | 0.008273402 | 0.981192392 | 0.994407944 |  |  |  |  |  |  |  |  |
| Bms1 | -0.019563456 | 0.625481971 | 0.850757879 |  |  |  |  |  |  |  |  |
| Bmyc | 0.227843267 | 0.152183269 | 0.428304076 |  |  |  |  |  |  |  |  |
| Bnip2 | -0.024476887 | 0.614648153 | 0.845810503 |  |  |  |  |  |  |  |  |
| Bnip3 | -0.268519673 | 0.034017551 | 0.153290388 |  |  |  |  |  |  |  |  |
| Bnip3l | -0.026498137 | 0.909048117 | 0.971925976 |  |  |  |  |  |  |  |  |
| Boc | -1.560125484 | 6.76863E-38 | 1.7415E-35 |  |  |  |  |  |  |  |  |
| Bod1 | -0.201785958 | 0.070608192 | 0.260008491 |  |  |  |  |  |  |  |  |
| Bod1l | -0.00170624 | 0.764261146 | 0.919979858 |  |  |  |  |  |  |  |  |
| Bok | 1.269661874 | 2.10169E-33 | 3.99121E-31 |  |  |  |  |  |  |  |  |
| Bola1 | 0.137994185 | 0.838429362 | 0.944669105 |  |  |  |  |  |  |  |  |
| Bola2 | 0.002507865 | 0.726050096 | 0.900898502 |  |  |  |  |  |  |  |  |
| Bola3 | -0.051842115 | 0.594385497 | 0.83362383 |  |  |  |  |  |  |  |  |
| Bpgm | 0.12183344 | 0.222571037 | 0.534706804 |  |  |  |  |  |  |  |  |
| Bphl | 0.11517556 | 0.271231526 | 0.589493025 |  |  |  |  |  |  |  |  |
| Bpnt1 | -0.069452825 | 0.759690907 | 0.918279887 |  |  |  |  |  |  |  |  |
| Bptf | 0.017506916 | 0.823126426 | 0.941930614 |  |  |  |  | FMRP |  |  |  |
| Braf | 0.002205359 | 0.895123117 | 0.965827133 |  | ID | ASD |  |  |  |  |  |
| Brap | 0.055051699 | 0.502968618 | 0.782106171 |  |  |  |  |  |  |  |  |
| Brcc3 | 0.12085285 | 0.347775333 | 0.667779594 |  |  |  |  |  |  |  |  |
| Brd1 | -0.029826162 | 0.750398125 | 0.914185954 |  |  |  |  |  | SZdb |  | SZ_full |
| Brd2 | 0.18100766 | 0.037945251 | 0.166933991 |  |  |  |  |  |  |  |  |
| Brd3 | -0.037471278 | 0.722367412 | 0.899126479 |  |  |  |  |  |  |  |  |
| Brd4 | 0.077991588 | 0.517422062 | 0.789546273 |  |  |  |  | FMRP |  |  |  |
| Brd7 | 0.028265529 | 0.623579105 | 0.85035801 |  |  |  |  |  |  |  |  |
| Brd8 | -0.044862565 | 0.556598388 | 0.810114733 |  |  |  |  |  |  |  |  |
| Brd9 | 0.336701233 | 0.002319084 | 0.018259638 |  |  |  |  |  |  |  |  |
| Brdt | 0.009594624 | 0.729865177 | 0.903103421 |  |  |  |  |  |  |  |  |
| Bre | -0.074204281 | 0.350888333 | 0.669541948 |  |  |  |  |  |  |  |  |
| Bri3 | 0.198460203 | 0.071068184 | 0.261216516 |  |  |  |  |  |  |  |  |
| Bri3bp | -0.120300175 | 0.290690297 | 0.607744642 |  |  |  |  |  |  |  |  |
| Brk1 | 0.171612245 | 0.044511271 | 0.189749812 |  |  |  |  |  |  |  |  |
| Brms1l | 0.100517405 | 0.229512924 | 0.542237879 |  |  |  |  |  |  |  |  |
| Brpf1 | 0.10591513 | 0.31144253 | 0.628241179 |  |  |  |  |  |  |  |  |
| Brpf3 | -0.165068553 | 0.117256225 | 0.365183775 |  |  |  |  |  |  |  |  |
| Brsk1 | -0.151464166 | 0.086508812 | 0.299346763 | SYN |  |  |  | FMRP |  |  |  |
| Brsk2 | -0.248632052 | 0.003032736 | 0.022543433 | SYN |  |  |  | FMRP |  |  |  |
| Brwd1 | -0.15520298 | 0.070439657 | 0.25986434 |  |  |  |  |  |  |  |  |
| Bscl2 | 0.10932151 | 0.275059533 | 0.592509543 |  | ID |  |  |  |  |  |  |
| Bsdc1 | -0.039885406 | 0.777409713 | 0.924797943 |  |  |  |  |  |  |  |  |
| Bsg | 0.063125844 | 0.384605136 | 0.698108192 | SYN |  |  |  |  |  |  |  |
| Bsn | -0.394091205 | 1.77378E-06 | 2.95358E-05 | SYN |  |  |  | FMRP |  |  |  |
| Btaf1 | -0.021337789 | 0.827175487 | 0.941969116 |  |  | ASD |  |  |  |  |  |
| Btbd1 | -0.159945981 | 0.151424507 | 0.426634282 |  |  |  |  |  |  |  |  |
| Btbd10 | -0.040916605 | 0.863337412 | 0.953809724 |  |  |  |  |  |  |  |  |
| Btbd11 | -0.25972344 | 0.021760304 | 0.108815161 |  |  |  |  |  |  |  |  |
| Btbd17 | 0.062981578 | 0.980934477 | 0.994381525 | SYN |  |  |  |  |  |  |  |
| Btbd19 | 0.070671595 | 0.595673766 | 0.833964185 |  |  |  |  |  |  |  |  |
| Btbd2 | 0.146675181 | 0.173488157 | 0.462387242 |  |  |  |  |  |  |  |  |
| Btbd3 | -0.269559337 | 0.005374578 | 0.035723026 |  |  |  |  |  |  |  |  |
| Btbd6 | -0.056361146 | 0.704730303 | 0.889528231 |  |  |  |  |  |  |  |  |
| Btbd9 | 0.248161472 | 0.014133367 | 0.077263696 |  |  |  |  |  |  |  |  |
| Btd | 0.095465041 | 0.433266932 | 0.73436095 |  | ID |  |  |  |  |  |  |
| Btf3 | 0.151836865 | 0.225359377 | 0.538647406 |  |  |  |  |  |  |  |  |
| Btf3l4 | -0.038482875 | 0.95410596 | 0.988885952 |  |  |  |  |  |  |  |  |
| Btrc | -0.077966564 | 0.438148694 | 0.738207432 |  |  |  |  |  |  |  |  |
| Bub3 | 0.043363043 | 0.596915593 | 0.834737271 |  |  |  |  |  |  |  |  |
| Bud13 | 0.017161589 | 0.973444058 | 0.991836537 |  |  |  |  |  |  |  |  |
| Bud31 | 0.104224968 | 1 | 1 |  |  |  |  |  |  |  |  |
| Bzrap1 | -0.353186823 | 0.01612809 | 0.085986254 |  |  | ASD | ASD_sc | FMRP |  |  |  |
| Bzw1 | -0.031322822 | 0.92587366 | 0.979802085 |  |  |  |  |  |  |  |  |
| Bzw2 | 0.370432475 | 9.87031E-05 | 0.0011393 |  |  |  |  |  |  |  |  |
| C030023E24Rik | 0.127381735 | 0.613381381 | 0.844961985 |  |  |  |  |  |  |  |  |
| C030046E11Rik | 0.399144002 | 3.23626E-05 | 0.000416329 |  |  |  |  |  |  |  |  |
| C1d | 0.194389128 | 0.190608618 | 0.48910346 |  |  |  |  |  |  |  |  |
| C1qb | -0.184873393 | 0.308949585 | 0.625861676 |  |  |  |  |  |  |  |  |
| C1qc | -0.103273511 | 0.572167557 | 0.820827732 | SYN |  |  |  |  |  |  |  |
| C1ql3 | -0.151330633 | 0.674184257 | 0.878031572 |  |  |  |  |  |  |  |  |
| C1qtnf4 | -0.433668869 | 0.00850401 | 0.051975468 |  |  |  |  |  |  |  |  |
| C230081A13Rik | -0.571128757 | 0.209375238 | 0.516221607 |  |  |  |  |  |  |  |  |
| C230091D08Rik | -0.133854572 | 0.248340637 | 0.56544732 |  |  |  |  |  |  |  |  |
| C2cd2 | -0.41258949 | 0.001597906 | 0.013500948 |  |  |  |  |  |  |  |  |
| C2cd2l | 0.016534271 | 0.77091156 | 0.921167131 |  |  |  |  | FMRP |  |  |  |
| C2cd4c | -1.821874023 | 1.38223E-23 | 1.37808E-21 |  |  |  |  |  |  |  |  |
| C2cd5 | -0.099003402 | 0.176638397 | 0.466667061 |  |  |  |  |  |  |  |  |
| C530008M17Rik | 0.084629003 | 0.299086647 | 0.616077767 |  |  |  |  |  |  |  |  |
| C5ar2 | 0.108218426 | 0.361755293 | 0.678908287 |  |  |  |  |  |  |  |  |
| C77370 | 0.196023398 | 0.041826854 | 0.180721014 |  | ID | ASD |  |  |  |  |  |
| C78339 | 0.03211577 | 0.882317292 | 0.960863288 |  |  |  |  |  |  |  |  |
| Cab39 | -0.104480315 | 0.401694208 | 0.711185063 |  |  |  |  |  |  |  |  |
| Cab39l | 0.256538234 | 0.031980013 | 0.145922532 |  |  |  |  |  |  |  |  |
| Cabin1 | -0.073417239 | 0.329491985 | 0.647935916 |  |  |  |  | FMRP |  |  |  |
| Cables2 | -0.30205995 | 0.004944594 | 0.033478849 |  |  |  |  |  |  |  |  |
| Cabp1 | -0.458502071 | 0.000116917 | 0.001313427 |  |  |  |  |  |  |  |  |
| Cacfd1 | -0.104218955 | 0.427396301 | 0.731170745 |  |  |  |  |  |  |  |  |
| Cacna1a | -0.46013603 | 4.95043E-09 | 1.28197E-07 |  |  |  |  | FMRP |  |  |  |
| Cacna1b | -0.258599204 | 0.002506246 | 0.019407591 |  |  | ASD |  | FMRP |  |  |  |
| Cacna1c | -0.312223062 | 0.050308979 | 0.20651797 |  |  | ASD | ASD_sc |  |  | SZ_108 | SZ_full |
| Cacna1d | -0.281702683 | 0.00288673 | 0.021683831 |  |  | ASD |  |  |  |  |  |
| Cacna1e | 0.174546531 | 0.048338489 | 0.201132968 |  |  |  |  | FMRP |  |  |  |
| Cacna1g | 0.054262218 | 0.503066866 | 0.782106171 |  |  | ASD |  | FMRP |  |  |  |
| Cacna1h | 0.390614578 | 0.004275188 | 0.029780696 |  |  | ASD | ASD_sc |  |  |  |  |
| Cacna1i | -0.366660622 | 2.83577E-05 | 0.000369577 |  |  | ASD |  | FMRP |  | SZ_108 | SZ_full |
| Cacna2d1 | -0.276266008 | 0.001382032 | 0.011816061 | SYN |  |  |  |  |  |  |  |
| Cacna2d2 | -0.266656333 | 0.014915848 | 0.080602172 | SYN |  |  |  |  |  |  |  |
| Cacna2d3 | 0.044983542 | 0.703771339 | 0.888802931 | SYN |  | ASD | ASD_sc |  |  |  |  |
| Cacnb1 | 0.399616656 | 1.32235E-05 | 0.000185036 | SYN |  |  |  | FMRP |  |  |  |
| Cacnb2 | 0.175130736 | 0.165472233 | 0.450445915 |  |  | ASD | ASD_sc |  |  | SZ_108 | SZ_full |
| Cacnb3 | -0.26642295 | 0.015004219 | 0.080969994 | SYN |  |  |  | FMRP |  |  |  |
| Cacnb4 | -0.392653855 | 2.54862E-06 | 4.0901E-05 | SYN |  |  |  |  |  |  |  |
| Cacng2 | -0.428900566 | 3.02548E-06 | 4.78794E-05 | SYN |  |  |  |  |  |  |  |
| Cacng3 | -0.47939299 | 1.93201E-06 | 3.15773E-05 | SYN |  |  |  |  |  |  |  |
| Cacng5 | -0.374708933 | 0.152994834 | 0.42907412 |  |  |  |  |  |  |  |  |
| Cacng7 | -0.117967574 | 0.126759191 | 0.38477273 |  |  |  |  |  |  |  |  |
| Cacng8 | 0.333308963 | 0.010474538 | 0.061384975 |  |  |  |  |  |  |  |  |
| Cactin | 0.08471291 | 0.530197607 | 0.796094902 |  |  |  |  |  |  |  |  |
| Cacul1 | -0.138201044 | 0.221408326 | 0.532647393 |  |  |  |  |  |  |  |  |
| Cacybp | 0.083897552 | 0.426387136 | 0.73059413 | SYN |  |  |  |  |  |  |  |
| Cad | 0.164916914 | 0.259002348 | 0.576917826 | SYN |  |  |  |  |  |  |  |
| Cadm1 | 0.213621613 | 0.011000587 | 0.063672484 |  |  | ASD | ASD_sc |  |  |  |  |
| Cadm2 | -0.001723131 | 0.789793326 | 0.931748979 |  |  |  |  |  |  |  |  |
| Cadm3 | -0.336105987 | 0.002313062 | 0.018230218 |  |  |  |  |  |  |  |  |
| Cadm4 | 0.075915618 | 0.404142181 | 0.713459178 |  |  |  |  |  |  |  |  |
| Cadps | -0.085747007 | 0.1731082 | 0.462387242 | SYN |  |  |  | FMRP |  |  |  |
| Cadps2 | 0.603981693 | 3.46224E-07 | 6.62225E-06 |  |  | ASD | ASD_sc |  |  |  |  |
| Calb1 | -1.388112082 | 2.80563E-15 | 1.59841E-13 |  |  |  |  |  |  |  |  |
| Calcoco1 | 0.205598834 | 0.113811818 | 0.359083489 | SYN |  |  |  |  |  |  |  |
| Cald1 | -0.03050671 | 0.899284727 | 0.967861274 |  |  |  |  |  |  |  |  |
| Calm1 | -0.079861916 | 0.497860164 | 0.777851649 | SYN |  |  |  | FMRP |  |  |  |
| Calm2 | -0.128496183 | 0.560069478 | 0.813385681 |  |  |  |  |  |  |  |  |
| Calm3 | -0.009192012 | 0.594556689 | 0.833717326 | SYN |  |  |  | FMRP |  |  |  |
| Caln1 | 0.700544042 | 3.88918E-09 | 1.02716E-07 |  |  |  |  |  |  |  |  |
| Calr | 0.275684134 | 0.070267437 | 0.259805737 | SYN |  |  |  |  |  |  |  |
| Caly | 0.336048 | 0.008383335 | 0.051399981 |  |  |  |  |  |  |  |  |
| Camk1 | -0.265814054 | 0.00461746 | 0.03174902 |  |  |  |  |  |  |  |  |
| Camk1d | -0.719319254 | 0.146218514 | 0.419208794 |  |  |  |  |  |  |  |  |
| Camk1g | 0.040301741 | 0.511522359 | 0.786598895 |  |  |  |  |  |  |  |  |
| Camk2a | -0.012713049 | 0.632947106 | 0.855368709 | SYN |  |  |  | FMRP |  |  |  |
| Camk2b | 0.104498557 | 0.178301946 | 0.469661929 | SYN |  |  |  | FMRP |  |  |  |
| Camk2d | -0.546825714 | 0.014703509 | 0.079724806 |  |  |  |  |  |  |  |  |
| Camk2g | -0.349805498 | 7.04676E-06 | 0.00010486 | SYN |  |  |  |  |  |  |  |
| Camk2n1 | -0.851748884 | 1.9904E-05 | 0.000268166 |  |  |  |  | FMRP |  |  |  |
| Camk2n2 | -0.822804743 | 0.000361779 | 0.003629626 |  |  |  |  |  |  |  |  |
| Camk4 | -0.494592641 | 4.42076E-08 | 9.90449E-07 |  |  | ASD |  |  |  |  |  |
| Camkk1 | 0.449213594 | 1.4485E-05 | 0.000200576 | SYN |  |  |  |  |  |  |  |
| Camkk2 | -0.071996868 | 0.326587117 | 0.643970048 | SYN |  |  |  | FMRP |  |  |  |
| Camkmt | 0.055341224 | 0.608082791 | 0.841251463 |  |  |  |  |  |  |  |  |
| Camkv | 0.234965112 | 0.070829025 | 0.260457493 | SYN |  |  |  |  |  |  |  |
| Camsap1 | -0.074416408 | 0.477620863 | 0.765729447 |  |  |  |  | FMRP |  |  |  |
| Camsap2 | 0.020665511 | 0.549215595 | 0.807161534 |  |  | ASD |  |  |  |  |  |
| Camta1 | 0.133545756 | 0.075191058 | 0.270877995 |  |  | ASD |  | FMRP |  |  |  |
| Camta2 | -0.094400562 | 0.3084742 | 0.625257997 |  |  |  |  | FMRP |  |  |  |
| Cand1 | -0.056421251 | 0.936845212 | 0.98530815 | SYN |  |  |  | FMRP |  |  |  |
| Cant1 | 0.069458143 | 0.878960842 | 0.959555967 |  |  |  |  |  |  |  |  |
| Canx | 0.090683965 | 0.172578485 | 0.461777959 | SYN |  |  |  |  |  |  |  |
| Cap1 | 0.311671751 | 8.15305E-05 | 0.000956408 | SYN |  |  |  |  |  |  |  |
| Cap2 | -0.620402421 | 1.78268E-06 | 2.96223E-05 | SYN |  |  |  |  |  |  |  |
| Capn1 | 0.351825775 | 0.001246173 | 0.010803782 | SYN |  |  |  |  |  |  |  |
| Capn2 | -0.129646299 | 0.072485093 | 0.264232678 |  |  |  |  |  |  |  |  |
| Capn5 | 0.018549763 | 0.9454467 | 0.985962641 | SYN |  |  |  |  |  |  |  |
| Capn7 | 0.026567585 | 0.532625159 | 0.797338264 |  |  |  |  |  |  |  |  |
| Capns1 | 0.234792722 | 0.005398732 | 0.03576436 |  |  |  |  |  |  |  |  |
| Caprin1 | 0.06431684 | 0.30387769 | 0.621556507 |  |  | ASD |  |  |  |  |  |
| Caprin2 | -0.561840029 | 2.68603E-05 | 0.000352365 |  |  |  |  |  |  |  |  |
| Capza1 | -0.020442205 | 0.859570104 | 0.952644879 | SYN |  |  |  |  |  |  |  |
| Capza2 | -0.07813445 | 0.487967678 | 0.772271082 | SYN |  |  |  |  |  |  |  |
| Capzb | 0.152486256 | 0.054793838 | 0.218190538 | SYN |  |  |  |  |  |  |  |
| Car10 | -1.005657356 | 5.06839E-31 | 8.08509E-29 |  |  |  |  |  |  |  |  |
| Car11 | 0.20359872 | 0.33972719 | 0.660719834 |  |  |  |  |  |  |  |  |
| Car14 | 0.481287842 | 0.005393495 | 0.035759364 |  |  |  |  |  |  | SZ_108 | SZ_full |
| Car2 | 0.21141783 | 0.115733386 | 0.362992327 | SYN | ID |  |  |  |  |  |  |
| Car4 | -1.142632412 | 2.09749E-07 | 4.16158E-06 | SYN |  |  |  |  |  |  |  |
| Car5b | -0.010290501 | 0.770698913 | 0.921167131 |  |  |  |  |  |  |  |  |
| Car7 | 0.145659804 | 0.633486678 | 0.855539938 |  |  |  |  |  |  |  |  |
| Carkd | 0.099826209 | 0.599129883 | 0.835219314 |  |  |  |  |  |  |  |  |
| Carm1 | 0.115525564 | 0.184056269 | 0.478810439 |  |  |  |  |  |  |  |  |
| Cars | -0.085998101 | 0.375063851 | 0.690242104 |  |  |  |  |  |  |  |  |
| Cars2 | 0.338737968 | 0.259308275 | 0.576917826 |  |  |  |  |  |  |  |  |
| Casc3 | 0.093950462 | 0.39450883 | 0.705533207 |  |  |  |  |  |  |  |  |
| Casc4 | -0.206237043 | 0.022219509 | 0.110281768 |  |  | ASD | ASD_sc |  |  |  |  |
| Casd1 | 0.035731644 | 0.416152231 | 0.724350718 |  |  |  |  |  |  |  |  |
| Cask | 0.155540182 | 0.088321053 | 0.302697643 | SYN | ID |  |  |  |  |  |  |
| Caskin1 | -0.271342274 | 0.000940991 | 0.008462518 | SYN |  |  |  | FMRP |  |  |  |
| Caskin2 | 0.00869442 | 0.835270023 | 0.943188599 |  |  |  |  |  |  |  |  |
| Casp2 | 0.020218434 | 0.875164939 | 0.958702864 |  |  |  |  |  |  |  |  |
| Casp9 | -0.067156443 | 0.463815718 | 0.758236051 |  |  |  |  |  |  |  |  |
| Catsper2 | 0.071799407 | 0.710890572 | 0.893486165 |  |  |  |  |  |  |  |  |
| Cav2 | -0.965060764 | 3.69082E-05 | 0.000471007 |  |  |  |  |  |  |  |  |
| Cbfa2t2 | -0.003090738 | 0.732367498 | 0.905497313 |  |  |  |  |  |  |  |  |
| Cbfa2t3 | 0.370865964 | 0.021140737 | 0.106182945 |  |  |  |  |  |  |  |  |
| Cbl | 0.097154966 | 0.503843046 | 0.782447845 |  |  |  |  |  |  |  |  |
| Cbln2 | 1.352810641 | 2.62982E-43 | 9.9883E-41 |  |  |  |  |  |  |  |  |
| Cbln4 | -2.201098143 | 1.44045E-05 | 0.000199809 |  |  |  |  |  |  |  |  |
| Cbr1 | 0.058307427 | 0.889335631 | 0.963245653 | SYN |  |  |  |  |  |  |  |
| Cbs | 0.264359799 | 0.019596392 | 0.100321454 |  | ID | ASD |  |  |  |  |  |
| Cbx1 | 0.233791438 | 0.429798174 | 0.73153195 |  |  |  |  |  |  |  |  |
| Cbx3 | 0.098203151 | 0.191360802 | 0.490143423 |  |  |  |  |  |  |  |  |
| Cbx5 | 0.122092854 | 0.063955037 | 0.243603331 |  |  |  |  |  |  |  |  |
| Cbx7 | -0.072529058 | 0.479262234 | 0.765984483 |  |  |  |  |  |  |  |  |
| Cby1 | 0.171073289 | 0.20869503 | 0.515341041 |  |  |  |  |  |  |  |  |
| Cc2d1a | 0.088062102 | 0.512118171 | 0.787023994 | SYN |  | ASD | ASD_sc |  |  |  |  |
| Cc2d2a | -0.186083772 | 0.034176845 | 0.153811129 |  |  |  |  |  |  |  |  |
| Ccar1 | 0.119184898 | 0.22920278 | 0.541892633 |  |  |  |  |  |  |  |  |
| Ccbe1 | 1.916444271 | 2.85444E-05 | 0.000371403 |  |  |  |  |  |  |  |  |
| Ccbl1 | 0.172077258 | 0.235196518 | 0.549539852 |  |  |  |  |  |  |  |  |
| Ccdc104 | 0.3247872 | 0.001221968 | 0.01061701 |  |  |  |  |  |  |  |  |
| Ccdc107 | 0.025118371 | 0.897147833 | 0.966457471 |  |  |  |  |  |  |  |  |
| Ccdc108 | 0.452593606 | 0.002170634 | 0.017330304 |  |  |  |  |  |  |  |  |
| Ccdc112 | 0.107117396 | 0.473343043 | 0.76408458 |  |  |  |  |  |  |  |  |
| Ccdc115 | -0.022807886 | 0.860647756 | 0.952877083 |  |  |  |  |  |  |  |  |
| Ccdc12 | 0.162632816 | 0.408924999 | 0.719062719 |  |  |  |  |  |  |  |  |
| Ccdc120 | -0.153254316 | 0.290219422 | 0.607535998 |  |  |  |  |  |  |  |  |
| Ccdc124 | 0.106459839 | 0.680400374 | 0.878917067 | SYN |  |  |  |  |  |  |  |
| Ccdc127 | -0.0095752 | 1 | 1 | SYN |  |  |  |  |  |  |  |
| Ccdc132 | -0.195640491 | 0.065386665 | 0.247167793 |  |  |  |  |  |  |  |  |
| Ccdc136 | -0.88547792 | 1.76779E-23 | 1.74073E-21 |  |  |  |  |  |  |  |  |
| Ccdc141 | -0.102637116 | 0.387784121 | 0.699405224 |  |  |  |  |  |  |  |  |
| Ccdc142 | 0.169074575 | 0.389844083 | 0.701104037 |  |  |  |  |  |  |  |  |
| Ccdc148 | -0.146788806 | 0.358366307 | 0.676344578 |  |  |  |  |  |  |  |  |
| Ccdc164 | -0.067399328 | 0.757399154 | 0.916833457 |  |  |  |  |  |  |  |  |
| Ccdc177 | 0.09265714 | 0.54838002 | 0.806991235 |  |  |  |  |  |  |  |  |
| Ccdc25 | -0.023302707 | 0.864918768 | 0.954203365 |  |  |  |  |  |  |  |  |
| Ccdc28b | 0.356958934 | 0.181426443 | 0.474289516 |  |  |  |  |  |  |  |  |
| Ccdc3 | 1.86992301 | 1.7778E-14 | 9.45313E-13 |  |  |  |  |  |  |  |  |
| Ccdc30 | 0.161219962 | 0.229001491 | 0.541892633 |  |  |  |  |  |  |  |  |
| Ccdc32 | 0.072524715 | 0.289939965 | 0.607535998 |  |  |  |  |  |  |  |  |
| Ccdc39 | 0.123618238 | 0.496669228 | 0.77686598 |  |  |  |  |  |  | SZ_108 | SZ_full |
| Ccdc41 | -0.16403038 | 0.054687213 | 0.217874731 |  |  |  |  |  |  |  |  |
| Ccdc43 | -0.134235751 | 0.472404595 | 0.76369839 |  |  |  |  |  |  |  |  |
| Ccdc47 | 0.266213808 | 0.014487152 | 0.078819593 |  |  |  |  |  |  |  |  |
| Ccdc50 | -0.227462835 | 0.01620616 | 0.086180687 |  |  |  |  |  |  |  |  |
| Ccdc57 | -0.228229183 | 0.087623065 | 0.301965898 |  |  |  |  |  |  |  |  |
| Ccdc6 | 0.302227937 | 0.000740856 | 0.006832777 |  |  |  |  |  |  |  |  |
| Ccdc64 | -0.125509943 | 0.185960898 | 0.481595848 |  |  | ASD | ASD_sc |  |  |  |  |
| Ccdc66 | 0.130501059 | 0.758660364 | 0.917637037 |  |  |  |  |  |  |  |  |
| Ccdc71 | -0.00316873 | 0.857080815 | 0.95156968 |  |  |  |  |  |  |  |  |
| Ccdc71l | -0.038298988 | 0.950932365 | 0.988210704 |  |  |  |  |  |  |  |  |
| Ccdc84 | 0.193041169 | 0.341179829 | 0.6621047 |  |  |  |  |  |  |  |  |
| Ccdc85a | -0.645136218 | 2.27633E-10 | 7.09218E-09 |  |  |  |  |  |  |  |  |
| Ccdc86 | 0.260014939 | 0.215216667 | 0.521911869 |  |  |  |  |  |  |  |  |
| Ccdc88a | -0.035887186 | 0.601452225 | 0.836949801 |  |  |  |  |  |  |  |  |
| Ccdc90a | -0.146484484 | 0.167171836 | 0.453062373 |  |  |  |  |  |  |  |  |
| Ccdc90b | -0.088366938 | 0.571458921 | 0.820827732 |  |  |  |  |  |  |  |  |
| Ccdc91 | -0.180923377 | 0.066528587 | 0.250180109 |  |  |  |  |  |  |  |  |
| Ccdc92 | 0.193906428 | 0.125279082 | 0.381675309 |  |  |  |  |  |  |  |  |
| Ccdc93 | -0.107388864 | 0.322175296 | 0.639380483 | SYN |  |  |  |  |  |  |  |
| Ccdc94 | 0.268740439 | 0.100162865 | 0.330779152 |  |  |  |  |  |  |  |  |
| Ccdc97 | 0.119766519 | 0.214676178 | 0.520996547 |  |  |  |  |  |  |  |  |
| Cck | 0.81569689 | 3.92779E-07 | 7.45906E-06 |  |  |  |  |  |  |  |  |
| Cckbr | -1.544765841 | 3.57211E-34 | 7.12279E-32 |  |  |  |  |  |  |  |  |
| Ccl21c | -1.445331934 | 0.009185108 | 0.055416354 |  |  |  |  |  |  |  |  |
| Ccl25 | 0.118425506 | 0.5891916 | 0.829543792 |  |  |  |  |  |  |  |  |
| Ccl27a | -0.946792746 | 1.50752E-05 | 0.000207667 |  |  |  |  |  |  |  |  |
| Ccl27b | -1.575779456 | 1.98184E-05 | 0.000267465 |  |  |  |  |  |  |  |  |
| Ccm2 | 0.041345855 | 0.69225923 | 0.884375762 |  |  |  |  |  |  |  |  |
| Ccnc | 0.026989415 | 0.789813797 | 0.931748979 |  |  |  |  |  |  |  |  |
| Ccnd1 | -0.369677583 | 0.124671189 | 0.380550097 |  |  |  |  |  |  |  |  |
| Ccnd2 | 0.463807518 | 4.45198E-06 | 6.86828E-05 |  |  |  |  |  |  |  |  |
| Ccnd3 | 0.311549916 | 0.016515299 | 0.087378758 |  |  |  |  |  |  |  |  |
| Ccndbp1 | 0.021141313 | 0.62931579 | 0.853643323 |  |  |  |  |  |  |  |  |
| Ccne1 | -0.77929306 | 1.62226E-07 | 3.2924E-06 |  |  |  |  |  |  |  |  |
| Ccne2 | -0.154800097 | 0.464235201 | 0.75844735 |  |  |  |  |  |  |  |  |
| Ccng1 | -0.032157356 | 0.972532827 | 0.991571804 |  |  |  |  |  |  |  |  |
| Ccnh | 0.082626695 | 0.357528992 | 0.675586647 |  |  |  |  |  |  |  |  |
| Ccni | -0.258934353 | 0.012347698 | 0.069567974 |  |  |  |  |  |  |  |  |
| Ccnk | -0.019901326 | 0.547133521 | 0.806637752 |  |  |  |  |  |  |  |  |
| Ccnl1 | 0.037981977 | 0.701475799 | 0.88745677 |  |  |  |  |  |  |  |  |
| Ccnl2 | 0.233360193 | 0.023122683 | 0.11391385 |  |  |  |  |  |  |  |  |
| Ccny | -0.056458355 | 0.657867295 | 0.869611321 | SYN |  |  |  |  |  |  |  |
| Ccnyl1 | -0.108005021 | 0.305005614 | 0.622657992 |  |  |  |  |  |  |  |  |
| Ccp110 | 0.093783082 | 0.419075381 | 0.726086902 |  |  |  |  |  |  |  |  |
| Ccpg1 | 0.130837491 | 0.158226348 | 0.438503597 |  |  |  |  |  |  |  |  |
| Ccrn4l | 0.163716179 | 0.054496236 | 0.217548538 |  |  |  |  |  |  |  |  |
| Ccs | 0.128354278 | 0.451130275 | 0.74791417 |  |  |  |  |  |  |  |  |
| Ccsap | 0.304818833 | 0.000678629 | 0.006352987 |  |  |  |  |  |  |  |  |
| Ccser2 | -0.076239308 | 0.478524793 | 0.765984483 |  |  |  |  |  |  |  |  |
| Cct2 | 0.138945446 | 0.129273311 | 0.389359415 | SYN |  |  |  |  |  |  |  |
| Cct3 | 0.129937748 | 0.118451757 | 0.367329398 | SYN |  |  |  |  |  |  |  |
| Cct4 | 0.08199906 | 0.252411709 | 0.570483568 | SYN |  |  |  |  |  |  |  |
| Cct5 | 0.119047674 | 0.159573308 | 0.441275196 | SYN |  |  |  |  |  |  |  |
| Cct6a | 0.108107668 | 0.158503236 | 0.439118378 | SYN |  |  |  |  |  |  |  |
| Cct7 | 0.137885463 | 0.08508277 | 0.296082099 | SYN |  |  |  |  |  |  |  |
| Ccz1 | 0.181810601 | 0.100701082 | 0.331897451 |  |  |  |  |  |  |  |  |
| Cd151 | -0.205423509 | 0.171894067 | 0.460849437 |  |  |  |  |  |  |  |  |
| Cd164 | -0.118149288 | 0.343958717 | 0.664586901 |  |  |  |  |  |  |  |  |
| Cd200 | 0.19810512 | 0.030370743 | 0.140345915 |  |  |  |  |  |  |  |  |
| Cd200r3 | -0.476115103 | 0.310116162 | 0.627296021 |  |  |  |  |  |  |  |  |
| Cd209c | 0.333321648 | 0.141721377 | 0.411342688 |  | ID |  |  |  |  |  |  |
| Cd276 | -0.195194115 | 0.143987609 | 0.416254139 |  |  |  |  |  |  |  |  |
| Cd2ap | -0.087372591 | 0.316703625 | 0.633884093 |  |  |  |  |  |  |  |  |
| Cd2bp2 | 0.181938532 | 0.028972673 | 0.134980166 |  |  |  |  |  |  |  |  |
| Cd302 | -1.230540356 | 9.1113E-09 | 2.29974E-07 |  |  |  |  |  |  |  |  |
| Cd320 | -0.027280529 | 0.560869125 | 0.813907347 |  |  |  |  |  |  |  |  |
| Cd34 | 0.550126455 | 0.000751763 | 0.006915748 |  |  |  |  |  |  |  |  |
| Cd46 | -0.138169972 | 0.119325216 | 0.369320109 |  |  |  |  |  |  | SZ_108 | SZ_full |
| Cd47 | 0.088341892 | 0.245714071 | 0.563224472 | SYN |  |  |  |  |  |  |  |
| Cd81 | 0.128330453 | 0.097966497 | 0.32534294 | SYN |  |  |  |  |  |  |  |
| Cd82 | 0.426555648 | 0.002737752 | 0.020895988 |  |  |  |  |  |  |  |  |
| Cd83 | -0.384256431 | 0.027360012 | 0.128942798 |  |  |  |  |  |  |  |  |
| Cd99l2 | -0.246021872 | 0.006836237 | 0.04383105 |  |  | ASD |  |  |  |  |  |
| Cdadc1 | -0.013281937 | 0.771982936 | 0.921894879 |  |  |  |  |  |  |  |  |
| Cdan1 | -0.044112635 | 0.520813584 | 0.791490992 |  |  |  |  |  |  |  |  |
| Cdc123 | 0.01608963 | 0.973912462 | 0.991836537 |  |  |  |  |  |  |  |  |
| Cdc16 | -0.002468455 | 0.802245805 | 0.935393732 |  |  |  |  |  |  |  |  |
| Cdc23 | 0.063918 | 0.490671058 | 0.773853814 |  |  |  |  |  |  |  |  |
| Cdc25b | -0.130679431 | 0.105208684 | 0.341804287 |  |  |  |  |  |  |  |  |
| Cdc27 | 0.055103677 | 0.756976368 | 0.916833457 |  |  |  |  |  |  |  |  |
| Cdc37 | 0.145134429 | 0.12521439 | 0.38164556 |  |  |  |  |  |  |  |  |
| Cdc37l1 | 0.052439521 | 0.397470623 | 0.707892459 |  |  |  |  |  |  |  |  |
| Cdc40 | 0.267231091 | 0.012679883 | 0.070971751 |  |  |  |  |  |  |  |  |
| Cdc42 | -0.019912667 | 0.962268716 | 0.990072781 | SYN |  |  |  |  |  |  |  |
| Cdc42bpa | -0.161330111 | 0.071512161 | 0.261882918 | SYN |  |  |  | FMRP |  |  |  |
| Cdc42bpb | -0.193259431 | 0.030107134 | 0.139444138 | SYN |  | ASD | ASD_sc | FMRP |  |  |  |
| Cdc42ep4 | -0.046596906 | 0.962391458 | 0.990072781 | SYN |  |  |  |  |  |  |  |
| Cdc42se1 | 0.232895266 | 0.014857639 | 0.080451141 |  |  |  |  |  |  |  |  |
| Cdc42se2 | -0.03921814 | 0.929055084 | 0.98139961 |  |  |  |  |  | SZdb |  | SZ_full |
| Cdc5l | 0.080899014 | 0.459144287 | 0.754768103 |  |  |  |  |  |  |  |  |
| Cdc7 | 0.245377641 | 0.07393034 | 0.267727747 |  |  |  |  |  |  |  |  |
| Cdc73 | 0.202250416 | 0.100084034 | 0.330683618 |  |  |  |  |  |  |  |  |
| Cdh10 | 0.160227773 | 0.161128257 | 0.443464105 | SYN |  | ASD | ASD_sc |  |  |  |  |
| Cdh11 | -0.130712049 | 0.169242205 | 0.457120158 |  |  | ASD |  |  |  |  |  |
| Cdh12 | -0.260947493 | 0.013394207 | 0.074034787 |  |  |  |  |  |  |  |  |
| Cdh13 | 0.131283856 | 0.347595509 | 0.667731642 | SYN |  |  |  |  |  |  |  |
| Cdh18 | -0.008594219 | 0.926567998 | 0.980146731 |  |  |  |  |  |  |  |  |
| Cdh19 | 0.067119847 | 0.75985542 | 0.918279887 |  |  |  |  |  |  |  |  |
| Cdh2 | -0.030209995 | 0.928864983 | 0.98139961 | SYN |  |  |  |  |  |  |  |
| Cdh20 | 0.082266743 | 0.459493236 | 0.754875159 |  |  |  |  |  |  |  |  |
| Cdh22 | 0.148441631 | 0.342185162 | 0.662914472 |  |  | ASD | ASD_sc |  |  |  |  |
| Cdh24 | 0.924255168 | 7.64694E-11 | 2.5735E-09 |  |  |  |  |  |  |  |  |
| Cdh4 | 0.494764627 | 0.000270929 | 0.00281005 | SYN |  |  |  |  |  |  |  |
| Cdh6 | -2.179814834 | 2.1933E-50 | 1.02904E-47 |  |  |  |  |  |  |  |  |
| Cdh8 | -0.537493834 | 6.60416E-08 | 1.44711E-06 |  |  | ASD | ASD_sc |  |  |  |  |
| Cdh9 | 1.673840424 | 4.4758E-53 | 2.37993E-50 |  |  | ASD | ASD_sc |  |  |  |  |
| Cdip1 | 0.088281269 | 0.312159243 | 0.629368585 |  |  |  |  |  |  |  |  |
| Cdipt | -0.015718814 | 0.766443342 | 0.920286747 | SYN |  |  |  |  |  |  |  |
| Cdk10 | -0.189150939 | 0.046759084 | 0.196911538 |  |  |  |  |  |  |  |  |
| Cdk12 | 0.000861275 | 0.988581687 | 0.99759678 |  |  |  |  |  |  |  |  |
| Cdk13 | -0.06538188 | 0.723777516 | 0.899896975 |  |  |  |  |  |  |  |  |
| Cdk14 | 0.268377895 | 0.000752617 | 0.006915748 |  |  |  |  |  |  |  |  |
| Cdk16 | -0.208992529 | 0.008341191 | 0.051399981 |  |  |  |  |  |  |  |  |
| Cdk17 | 0.194322291 | 0.028965153 | 0.134980166 |  |  |  |  |  |  |  |  |
| Cdk18 | 0.808647108 | 1.6388E-05 | 0.000225139 |  |  |  |  |  |  |  |  |
| Cdk19 | -0.37181336 | 0.000320574 | 0.00325305 |  |  |  |  |  |  |  |  |
| Cdk2ap1 | 0.004896503 | 0.770114982 | 0.921167131 |  |  |  |  |  |  | SZ_108 | SZ_full |
| Cdk4 | 0.171438127 | 0.150050242 | 0.425756219 |  |  |  |  |  |  |  |  |
| Cdk5 | 0.035295565 | 0.734130985 | 0.905992378 | SYN |  |  |  |  |  |  |  |
| Cdk5r1 | -0.22484681 | 0.013432492 | 0.074143636 | SYN |  |  |  | FMRP |  |  |  |
| Cdk5r2 | -0.018659356 | 0.571068811 | 0.820692764 |  |  |  |  | FMRP |  |  |  |
| Cdk5rap2 | 0.175711658 | 0.154661854 | 0.431926802 |  |  |  |  |  |  |  |  |
| Cdk7 | -0.025708907 | 0.768487465 | 0.920494741 |  |  |  |  |  |  |  |  |
| Cdk8 | -0.437904305 | 0.266663196 | 0.583833559 |  | ID |  |  |  |  |  |  |
| Cdk9 | -0.175034068 | 0.048576174 | 0.202003942 |  |  |  |  |  |  |  |  |
| Cdkl1 | 0.11021668 | 0.676993446 | 0.878273493 |  |  |  |  |  |  |  |  |
| Cdkl2 | 0.08408504 | 0.465878321 | 0.760042031 | SYN |  |  |  |  |  |  |  |
| Cdkl4 | 0.563905509 | 1.18386E-07 | 2.43992E-06 |  |  |  |  |  |  |  |  |
| Cdkl5 | -0.351460696 | 0.0015628 | 0.013218337 | SYN | ID | ASD | ASD_sc | FMRP |  |  |  |
| Cdkn1b | -0.113446052 | 0.479489555 | 0.765984483 |  |  | ASD |  |  |  |  |  |
| Cdkn2aipnl | -0.073099374 | 0.863883756 | 0.953943907 |  |  |  |  |  |  |  |  |
| Cdkn2d | -0.03518158 | 0.658752551 | 0.870046422 |  |  |  |  |  |  |  |  |
| Cds1 | -0.688499033 | 2.21235E-15 | 1.27867E-13 |  |  |  |  |  |  |  |  |
| Cds2 | -0.073172606 | 0.537958058 | 0.801453443 |  |  |  |  |  |  |  |  |
| Cdv3 | -0.128199825 | 0.305606604 | 0.62282745 |  |  |  |  |  |  |  |  |
| Cebpg | -0.163298118 | 0.184669496 | 0.479936102 |  |  |  |  |  |  |  |  |
| Cebpz | 0.055255784 | 0.560684785 | 0.813834731 |  |  |  |  |  |  |  |  |
| Cecr5 | -0.182724828 | 0.024023132 | 0.11712011 |  |  |  |  |  |  |  |  |
| Cecr6 | 0.029800569 | 0.914279128 | 0.974441679 |  |  |  |  |  |  |  |  |
| Celf1 | -0.139058382 | 0.155034075 | 0.432814764 |  | ID |  |  |  |  |  |  |
| Celf2 | 0.051860213 | 0.866124994 | 0.954438815 |  |  |  |  |  |  |  |  |
| Celf3 | 0.220457714 | 0.011341635 | 0.06507977 |  |  |  |  |  |  |  |  |
| Celf4 | -0.009042236 | 0.882621692 | 0.961049333 |  |  | ASD |  |  |  |  |  |
| Celf5 | 0.416920913 | 0.000710077 | 0.006608602 |  |  |  |  |  |  |  |  |
| Celsr2 | 0.332531151 | 1.35326E-05 | 0.00018903 |  |  |  |  | FMRP |  |  |  |
| Celsr3 | 0.029419963 | 0.788252402 | 0.930732962 |  |  |  |  | FMRP |  |  |  |
| Cend1 | 0.025256615 | 0.848395676 | 0.947466244 | SYN |  |  |  |  |  |  |  |
| Cenpp | -0.081014368 | 0.483022143 | 0.768365499 |  |  |  |  |  |  |  |  |
| Cenpv | 0.113232842 | 0.688713638 | 0.882941855 |  |  |  |  |  |  |  |  |
| Cep112 | -0.020902997 | 0.849309659 | 0.9478234 |  |  |  |  |  |  |  |  |
| Cep152 | 0.040829309 | 0.986970731 | 0.996717973 |  |  |  |  |  |  |  |  |
| Cep170 | 0.078970141 | 0.276643221 | 0.59394256 |  |  |  |  |  |  |  |  |
| Cep170b | -0.269372172 | 0.00383035 | 0.02710814 |  |  |  |  |  |  |  |  |
| Cep19 | 0.029370435 | 0.861431539 | 0.953064889 |  |  |  |  |  |  |  |  |
| Cep250 | 0.038014113 | 0.877706829 | 0.959510646 |  |  |  |  |  |  |  |  |
| Cep290 | 0.045656004 | 0.743935816 | 0.911502699 |  |  | ASD |  |  |  |  |  |
| Cep350 | 0.034932752 | 0.904254992 | 0.97025806 |  |  |  |  |  |  |  |  |
| Cep57 | -0.049041491 | 0.668367823 | 0.875497086 |  |  |  |  |  |  |  |  |
| Cep63 | -0.316432771 | 0.007985154 | 0.049641141 |  |  |  |  |  |  |  |  |
| Cep70 | -0.173193397 | 0.084515841 | 0.294881167 |  |  |  |  |  |  |  |  |
| Cep76 | 0.270258128 | 0.070399904 | 0.259837869 |  |  |  |  |  |  |  |  |
| Cep78 | 0.033480166 | 0.873362043 | 0.957780236 |  |  |  |  |  |  |  |  |
| Cep95 | -0.087873133 | 0.426343608 | 0.73059413 |  |  |  |  |  |  |  |  |
| Cept1 | -0.059148113 | 0.50873919 | 0.785601869 |  |  |  |  |  |  |  |  |
| Cerk | -0.235785325 | 0.023710906 | 0.115966855 |  |  |  |  |  |  |  |  |
| Cers1 | 0.253200433 | 0.00958117 | 0.057285915 |  |  |  |  |  |  |  |  |
| Cers2 | 0.37081849 | 0.007355476 | 0.046304085 |  |  |  |  |  |  |  |  |
| Cers4 | -0.055612399 | 0.606784159 | 0.840665356 |  |  |  |  |  |  |  |  |
| Cers5 | -0.278605803 | 0.001877612 | 0.015391401 |  |  |  |  |  |  |  |  |
| Cers6 | 0.329817567 | 0.000685582 | 0.006410552 |  |  |  |  |  |  |  |  |
| Cetn3 | 0.250393293 | 0.013701771 | 0.075421204 |  |  |  |  |  |  |  |  |
| Cfl1 | 0.123031833 | 0.065104011 | 0.246216022 | SYN |  |  |  |  |  |  |  |
| Cfl2 | 0.001879342 | 0.789304031 | 0.931423132 | SYN |  |  |  |  |  |  |  |
| Cflar | 0.120056546 | 0.239552527 | 0.554531538 |  |  |  |  |  |  |  |  |
| Cfp | 0.237462142 | 0.503426916 | 0.782106171 |  |  |  |  |  |  |  |  |
| Cggbp1 | 0.004099304 | 0.591047951 | 0.830403111 |  |  |  |  |  |  |  |  |
| Cgnl1 | 0.07149553 | 0.377508168 | 0.693078766 |  |  |  |  |  |  |  |  |
| Cgrrf1 | -0.260443383 | 0.064523424 | 0.244949468 |  |  |  |  |  |  |  |  |
| Chchd1 | -0.036922588 | 0.708949176 | 0.892243729 |  |  |  |  |  |  |  |  |
| Chchd2 | 0.088090743 | 0.412123728 | 0.720855011 |  |  |  |  |  |  |  |  |
| Chchd3 | 0.178955378 | 0.151105815 | 0.426634282 | SYN |  |  |  |  |  |  |  |
| Chchd4 | -0.015600023 | 0.764662973 | 0.919979858 |  |  |  |  |  |  |  |  |
| Chchd6 | -0.006760319 | 0.784780561 | 0.928282627 | SYN |  |  |  |  |  |  |  |
| Chchd7 | -0.132556493 | 0.155326541 | 0.432873687 |  |  |  |  |  |  |  |  |
| Chd1 | -0.016680716 | 0.795202538 | 0.933274786 |  |  |  |  |  |  |  |  |
| Chd2 | -0.039413176 | 0.688385607 | 0.882869207 |  |  | ASD | ASD_sc |  |  |  |  |
| Chd3 | 0.22673498 | 0.003325507 | 0.024200952 |  |  |  |  | FMRP |  |  |  |
| Chd4 | -0.024872256 | 0.790160943 | 0.931851876 |  |  |  |  | FMRP |  |  |  |
| Chd5 | -0.102951312 | 0.320776524 | 0.637688241 |  |  |  |  | FMRP |  |  |  |
| Chd6 | -0.022418051 | 0.71057345 | 0.893228344 |  |  |  |  | FMRP |  |  |  |
| Chd8 | -0.044940807 | 0.514190267 | 0.787995408 |  |  | ASD | ASD_sc | FMRP |  |  |  |
| Chd9 | 0.118857748 | 0.207193535 | 0.513427381 |  |  |  |  |  |  |  |  |
| Chfr | 0.054797465 | 0.918512735 | 0.97628699 |  |  |  |  |  |  |  |  |
| Chga | 0.025262772 | 0.527528802 | 0.79458303 |  |  |  |  |  | SZdb |  | SZ_full |
| Chgb | 0.642656631 | 6.98002E-08 | 1.52111E-06 |  |  |  |  |  | SZdb |  | SZ_full |
| Chic1 | 0.133288136 | 0.272118297 | 0.590268027 |  |  |  |  |  |  |  |  |
| Chid1 | 0.104974388 | 0.273733084 | 0.591359138 |  |  |  |  |  |  |  |  |
| Chka | -0.006085321 | 0.688360411 | 0.882869207 |  |  |  |  |  |  |  |  |
| Chl1 | -0.104893197 | 1 | 1 | SYN |  |  |  |  | SZdb |  | SZ_full |
| Chm | 0.129486378 | 0.114195492 | 0.359724819 |  |  |  |  |  |  |  |  |
| Chmp1a | 0.088911326 | 0.250089479 | 0.567486111 | SYN |  |  |  |  |  |  |  |
| Chmp2a | 0.025624946 | 0.924671408 | 0.979179254 |  |  |  |  |  |  |  |  |
| Chmp2b | -0.050043699 | 0.815063439 | 0.938900345 |  |  |  |  |  |  |  |  |
| Chmp3 | -0.068782201 | 0.470956974 | 0.763331199 |  |  |  |  |  |  |  |  |
| Chmp4b | 0.00631346 | 0.732884167 | 0.905793617 | SYN |  |  |  |  |  |  |  |
| Chmp5 | 0.020506257 | 0.782255081 | 0.927358282 |  |  |  |  |  |  |  |  |
| Chmp6 | -0.345385979 | 0.016138195 | 0.085986254 |  |  |  |  |  |  |  |  |
| Chmp7 | -0.028364383 | 0.814108117 | 0.938538051 |  |  |  |  |  |  |  |  |
| Chn1 | 0.124648714 | 0.078794666 | 0.280408713 |  |  |  |  | FMRP |  |  |  |
| Chn2 | -0.953919997 | 2.02729E-17 | 1.46997E-15 |  |  |  |  | FMRP | SZdb |  | SZ_full |
| Chordc1 | 0.289826138 | 0.061012545 | 0.235615351 |  |  |  |  |  |  |  |  |
| Chp1 | -0.022144707 | 0.828055663 | 0.94210462 |  |  |  |  |  |  |  |  |
| Chpf | 0.183613208 | 0.060427962 | 0.23374075 |  |  |  |  |  |  |  |  |
| Chpf2 | 0.038008865 | 0.870911398 | 0.956962054 |  |  |  |  |  |  |  |  |
| Chpt1 | 0.178850772 | 0.121862148 | 0.375745837 |  |  |  |  |  |  |  |  |
| Chrd | 0.527364857 | 1.19051E-06 | 2.08235E-05 |  |  |  |  |  |  |  |  |
| Chrm1 | 0.250638467 | 0.003073197 | 0.022759349 |  |  |  |  |  |  |  |  |
| Chrm3 | -0.778351815 | 2.74367E-11 | 9.90205E-10 |  |  | ASD |  |  |  |  |  |
| Chrm4 | 0.268861785 | 0.00814809 | 0.050457425 |  |  |  |  |  |  | SZ_108 | SZ_full |
| Chrna4 | -0.141659314 | 0.197463952 | 0.499157953 |  |  |  |  |  |  |  |  |
| Chrna7 | -0.137619332 | 0.429709717 | 0.73153195 |  |  | ASD | ASD_sc |  | SZdb |  | SZ_full |
| Chrnb2 | -0.105305591 | 0.483468623 | 0.76869627 |  |  |  |  |  |  |  |  |
| Chst1 | -0.117945917 | 0.289896688 | 0.607535998 |  |  |  |  |  |  |  |  |
| Chst10 | 0.014320328 | 0.78400475 | 0.928282627 |  |  |  |  |  |  |  |  |
| Chst15 | -0.355593135 | 0.052791752 | 0.213306491 |  |  |  |  |  |  |  |  |
| Chst3 | 0.375083424 | 0.173117272 | 0.462387242 |  |  |  |  |  |  |  |  |
| Chsy1 | -0.214356537 | 0.172309134 | 0.461651883 |  |  |  |  |  |  |  |  |
| Chtf8 | 0.108026021 | 0.179148774 | 0.471152926 |  |  |  |  |  |  |  |  |
| Chtop | -0.008660517 | 0.975288574 | 0.99211875 |  |  |  |  |  |  |  |  |
| Churc1 | 0.007975512 | 0.721104234 | 0.898289262 |  |  |  |  |  |  |  |  |
| Ciapin1 | 0.078956162 | 0.382850958 | 0.696696153 |  |  |  |  |  |  |  |  |
| Cic | 0.054273102 | 0.365935032 | 0.683062524 |  |  |  |  | FMRP |  |  |  |
| Cir1 | 0.07540048 | 0.642196962 | 0.860723067 |  |  |  |  |  |  |  |  |
| Cirbp | -0.336750252 | 0.026393572 | 0.125083263 |  |  |  |  |  |  |  |  |
| Cirh1a | 0.207684639 | 0.058376593 | 0.227825652 |  |  |  |  |  |  |  |  |
| Cisd1 | 0.224172387 | 0.346620244 | 0.666442487 | SYN |  |  |  |  |  |  |  |
| Cisd2 | -0.066264761 | 0.625330001 | 0.850696245 |  |  |  |  |  |  |  |  |
| Cish | 0.103180128 | 0.585758483 | 0.827452483 |  |  |  |  |  |  |  |  |
| Cit | -0.387586794 | 0.000458703 | 0.004489095 | SYN |  |  |  | FMRP |  |  |  |
| Cited2 | -0.574723646 | 0.013955493 | 0.076659097 |  |  |  |  |  |  |  |  |
| Ciz1 | -0.088143695 | 0.238548851 | 0.553100475 |  |  |  |  |  |  |  |  |
| Ckap5 | -0.162848041 | 0.116931868 | 0.364627696 | SYN |  |  |  | FMRP |  | SZ_108 | SZ_full |
| Ckb | 0.046384065 | 0.640425629 | 0.860142008 | SYN |  |  |  | FMRP |  | SZ_108 | SZ_full |
| Ckmt1 | 0.207783094 | 0.014385588 | 0.078427512 | SYN |  |  |  |  |  |  |  |
| Clasp1 | -0.319779504 | 0.000455899 | 0.004472637 | SYN |  |  |  | FMRP |  |  |  |
| Clasp2 | -0.142228945 | 0.103166107 | 0.336682843 | SYN |  |  |  | FMRP |  |  |  |
| Clasrp | 0.100996879 | 0.558899893 | 0.812291307 |  |  |  |  |  |  |  |  |
| Clcn2 | 0.152770385 | 0.211210961 | 0.518502158 |  |  |  |  |  |  |  |  |
| Clcn3 | -0.270016326 | 0.002606832 | 0.020050235 | SYN |  |  |  | FMRP |  | SZ_108 | SZ_full |
| Clcn4-2 | -0.039703726 | 0.965708086 | 0.990416798 |  | ID |  |  |  |  |  |  |
| Clcn6 | 0.102383876 | 0.260193771 | 0.577744154 |  |  |  |  |  |  |  |  |
| Clcn7 | 0.054862948 | 0.577839656 | 0.823155759 |  |  |  |  |  |  |  |  |
| Cldn11 | 0.764157287 | 0.0002518 | 0.002632183 |  |  |  |  |  |  |  |  |
| Cldn12 | -0.034105632 | 0.718925796 | 0.897994027 |  |  |  |  |  |  |  |  |
| Cldn25 | -0.188048766 | 0.078593171 | 0.279847825 |  |  |  |  |  |  |  |  |
| Cldn5 | 0.049919497 | 0.775543234 | 0.924077297 |  |  |  |  |  | SZdb |  | SZ_full |
| Clec16a | -0.21080393 | 0.054405977 | 0.217405849 |  |  |  |  | FMRP |  |  |  |
| Clec2l | -0.03675356 | 0.477396836 | 0.765678094 |  |  |  |  |  |  |  |  |
| Clint1 | -0.257872482 | 0.011078345 | 0.064076056 |  |  |  |  |  | SZdb |  | SZ_full |
| Clip1 | -0.000583178 | 0.884795775 | 0.962081792 | SYN |  |  |  |  |  |  |  |
| Clip2 | -0.028591123 | 0.682997406 | 0.880635221 | SYN |  |  |  |  |  |  |  |
| Clip3 | 0.055819512 | 0.474789281 | 0.76408458 | SYN |  |  |  | FMRP |  |  |  |
| Clip4 | 0.175262945 | 0.153677959 | 0.430204164 |  |  |  |  |  |  |  |  |
| Clk1 | 0.115678972 | 0.35108683 | 0.669600324 |  |  |  |  |  |  |  |  |
| Clk4 | 0.069297263 | 0.52120827 | 0.791490992 |  |  |  |  |  |  |  |  |
| Clmn | 0.428630569 | 7.77958E-05 | 0.000917899 | SYN |  |  |  |  |  |  |  |
| Cln5 | 0.084778812 | 0.569854316 | 0.81993979 |  | ID |  |  |  |  |  |  |
| Clns1a | 0.108145609 | 0.214474422 | 0.520956779 |  |  |  |  |  |  |  |  |
| Clock | -0.039612076 | 0.854719208 | 0.950800614 |  |  |  |  |  | SZdb |  | SZ_full |
| Clpb | 0.013459587 | 0.890119887 | 0.963498449 |  |  |  |  |  |  |  |  |
| Clpp | 0.114158805 | 0.423138877 | 0.729913577 |  |  |  |  |  |  |  |  |
| Clptm1 | 0.047048042 | 0.428470773 | 0.73153195 |  |  |  |  |  |  |  |  |
| Clptm1l | -0.007406957 | 0.82475549 | 0.941950287 |  |  |  |  |  |  |  |  |
| Clstn1 | -0.336238731 | 0.000180736 | 0.001942795 |  |  |  |  | FMRP |  |  |  |
| Clstn2 | 0.449027803 | 2.26507E-07 | 4.46079E-06 |  |  |  |  |  |  |  |  |
| Clstn3 | -0.103474828 | 0.177985792 | 0.469160463 |  |  | ASD |  |  |  |  |  |
| Clta | 0.121878834 | 0.211229024 | 0.518502158 | SYN |  |  |  |  |  |  |  |
| Cltb | 0.079948716 | 0.525376714 | 0.793545744 | SYN |  |  |  |  |  |  |  |
| Cltc | 0.062280145 | 0.29520269 | 0.611588794 | SYN |  |  |  | FMRP |  |  |  |
| Clu | 0.166724939 | 0.064807493 | 0.245560365 | SYN |  |  |  |  |  | SZ_108 | SZ_full |
| Cluap1 | 0.002132591 | 0.746703698 | 0.912020725 |  |  |  |  |  |  |  |  |
| Cluh | 0.05495677 | 0.374212614 | 0.68963027 |  |  |  |  |  |  |  |  |
| Clvs1 | -0.399298241 | 0.000996827 | 0.008903352 |  |  |  |  |  |  |  |  |
| Clybl | 0.071370154 | 0.741326674 | 0.910365135 |  |  |  |  |  |  |  |  |
| Cmah | 0.109741593 | 0.627103546 | 0.852276259 |  |  |  |  |  |  |  |  |
| Cmas | -0.052288894 | 0.611941604 | 0.843999002 |  |  |  |  |  |  |  |  |
| Cmc1 | -0.296569693 | 0.008390182 | 0.051399981 | SYN |  |  |  |  |  |  |  |
| Cmip | 0.153881848 | 0.060669212 | 0.234560174 |  |  | ASD |  |  |  |  |  |
| Cmpk1 | -0.03559897 | 0.509034533 | 0.78576726 | SYN |  |  |  |  |  |  |  |
| Cmss1 | -0.612856329 | 0.185242106 | 0.480954114 |  |  |  |  |  |  |  |  |
| Cmtm5 | 0.431188972 | 0.001139516 | 0.009987669 |  |  |  |  |  |  |  |  |
| Cmtm6 | 0.033904857 | 0.71212912 | 0.894056644 |  |  |  |  |  |  |  |  |
| Cnbd2 | -0.316688852 | 0.113400362 | 0.358173379 |  |  |  |  |  |  |  |  |
| Cnbp | -0.014376346 | 0.850140602 | 0.948352649 |  |  |  |  |  |  |  |  |
| Cndp2 | -0.088210772 | 0.385287093 | 0.698492545 | SYN |  |  |  |  |  |  |  |
| Cnep1r1 | -0.040787109 | 0.619111819 | 0.848210083 |  |  |  |  |  |  |  |  |
| Cnih | -0.147128981 | 0.231262444 | 0.545281816 |  |  |  |  |  |  |  |  |
| Cnih2 | 0.905665649 | 2.8101E-06 | 4.47372E-05 |  |  |  |  |  |  |  |  |
| Cnksr2 | -0.058053925 | 0.422963085 | 0.729913577 | SYN |  |  |  |  |  | SZ_108 | SZ_full |
| Cnn3 | 0.170888378 | 0.038259174 | 0.168222257 |  |  |  |  |  |  |  |  |
| Cnnm1 | -0.229900275 | 0.009535602 | 0.057099068 |  |  |  |  |  |  |  |  |
| Cnnm3 | 0.116313797 | 0.267917164 | 0.585532517 |  |  |  |  |  |  |  |  |
| Cnot1 | -0.063405738 | 0.68985961 | 0.883708164 |  |  |  |  |  |  | SZ_108 | SZ_full |
| Cnot10 | -0.016355087 | 0.785558382 | 0.928789453 |  |  |  |  |  |  |  |  |
| Cnot2 | -0.033237758 | 0.767665637 | 0.920286747 |  |  |  |  |  |  |  |  |
| Cnot3 | 0.256866309 | 0.058189726 | 0.227622 |  |  |  |  |  |  |  |  |
| Cnot6 | -0.200346329 | 0.052764131 | 0.213306491 |  |  |  |  |  |  |  |  |
| Cnot6l | -0.050117526 | 0.840628675 | 0.94499151 |  |  |  |  |  |  |  |  |
| Cnot7 | -0.07334337 | 0.499286003 | 0.77924576 |  |  |  |  |  |  |  |  |
| Cnot8 | 0.061192711 | 0.416184312 | 0.724350718 |  |  |  |  |  |  |  |  |
| Cnp | 0.500475945 | 0.03424573 | 0.15393587 | SYN |  |  |  | FMRP | SZdb |  | SZ_full |
| Cnpy2 | 0.024700992 | 0.648993414 | 0.865179921 |  |  |  |  |  |  |  |  |
| Cnpy3 | 0.060265102 | 0.688774351 | 0.882941855 |  |  |  |  |  |  |  |  |
| Cnpy4 | 0.256981414 | 0.143285755 | 0.414826563 |  |  |  |  |  |  |  |  |
| Cnr1 | -0.067562775 | 0.64192462 | 0.860502651 |  |  | ASD |  |  | SZdb |  | SZ_full |
| Cnst | -0.108306384 | 0.427223488 | 0.731167599 |  |  |  |  |  |  |  |  |
| Cntfr | -0.054973473 | 0.617834035 | 0.847780211 | SYN |  |  |  |  |  |  |  |
| Cntln | -0.16660733 | 0.073575876 | 0.266903337 |  |  |  |  |  |  |  |  |
| Cntn1 | -0.048286825 | 0.700955482 | 0.887191673 | SYN |  |  |  |  |  |  |  |
| Cntn2 | 0.184620513 | 0.115309709 | 0.362073707 | SYN |  |  |  |  |  |  |  |
| Cntn4 | -0.388619822 | 1.23192E-05 | 0.000173908 |  |  | ASD | ASD_sc |  |  | SZ_108 | SZ_full |
| Cntn6 | -1.178262791 | 5.38432E-24 | 5.43611E-22 |  |  | ASD |  |  |  |  |  |
| Cntnap1 | 0.15525433 | 0.056911571 | 0.22416133 | SYN |  |  |  |  |  |  |  |
| Cntnap2 | -0.058879943 | 0.223686198 | 0.536094086 | SYN | ID | ASD | ASD_sc |  |  |  |  |
| Cntnap5c | 0.823138204 | 4.57987E-07 | 8.59506E-06 |  |  | ASD | ASD_sc |  |  |  |  |
| Coa3 | -0.188987873 | 0.466673065 | 0.760560762 |  |  |  |  |  |  |  |  |
| Coa5 | -0.095164012 | 0.410583814 | 0.719965338 |  |  |  |  |  |  |  |  |
| Coasy | 0.10847541 | 0.344349418 | 0.66472929 | SYN |  |  |  |  |  |  |  |
| Cobl | -0.159597776 | 0.114059689 | 0.359438987 |  |  |  |  | FMRP |  |  |  |
| Cobll1 | 0.06623319 | 0.31479884 | 0.631815689 |  |  |  |  |  |  |  |  |
| Coch | -2.567843361 | 4.93601E-23 | 4.68686E-21 |  |  |  |  |  |  |  |  |
| Cog2 | -0.169084167 | 0.078509362 | 0.27967426 |  |  |  |  |  |  |  |  |
| Cog3 | 0.006598891 | 0.941246364 | 0.985779516 | SYN |  |  |  |  |  |  |  |
| Cog4 | 0.020984807 | 0.875580196 | 0.95886148 |  |  |  |  |  |  |  |  |
| Cog5 | 0.004507249 | 0.775934819 | 0.924077297 |  |  |  |  |  |  |  |  |
| Cog7 | 0.007051257 | 0.966707642 | 0.990416798 |  |  |  |  |  |  |  |  |
| Cog8 | 0.053480002 | 0.598432749 | 0.835219314 |  |  |  |  |  |  |  |  |
| Coil | -0.004458736 | 0.821466811 | 0.941239661 |  |  |  |  |  |  |  |  |
| Col11a2 | 0.601982985 | 3.24528E-08 | 7.43804E-07 |  |  |  |  |  |  |  |  |
| Col23a1 | -0.323899713 | 0.252946625 | 0.570531033 |  |  |  |  |  |  |  |  |
| Col26a1 | -2.035266203 | 9.21511E-15 | 4.9662E-13 |  |  |  |  |  |  |  |  |
| Col4a1 | 0.281628291 | 0.0030429 | 0.022597923 |  |  |  |  |  |  |  |  |
| Col4a2 | 0.292687808 | 0.001962986 | 0.015976298 |  |  |  |  |  |  |  |  |
| Col4a3bp | -0.234814277 | 0.0181925 | 0.094468346 |  |  |  |  |  |  |  |  |
| Col6a1 | -0.182738053 | 0.858814675 | 0.952434072 |  |  |  |  |  |  |  |  |
| Col6a2 | -0.102457675 | 0.964541753 | 0.990416798 |  |  |  |  |  |  |  |  |
| Commd10 | -0.426268008 | 0.003763926 | 0.02685248 |  |  |  |  |  |  |  |  |
| Commd4 | 0.082514935 | 0.53689853 | 0.800280822 |  |  |  |  |  |  |  |  |
| Commd9 | -0.030195969 | 0.620112855 | 0.848665088 |  |  |  |  |  |  |  |  |
| Comt | 0.06202924 | 0.673491113 | 0.877823808 |  |  |  |  |  | SZdb |  | SZ_full |
| Comtd1 | 0.108733853 | 0.753909263 | 0.915220708 | SYN |  |  |  |  |  |  |  |
| Copa | -0.190292428 | 0.426171794 | 0.73059413 | SYN |  |  |  |  |  |  |  |
| Copb1 | -0.015164577 | 0.887382786 | 0.962995126 |  |  |  |  |  |  |  |  |
| Copb2 | -0.019061706 | 0.751407795 | 0.91454844 | SYN |  |  |  |  |  |  |  |
| Cope | 0.054448087 | 0.811348168 | 0.93732807 |  |  |  |  |  |  |  |  |
| Copg1 | 0.089738391 | 0.211465432 | 0.518502158 |  |  |  |  |  |  |  |  |
| Copg2 | -0.005259188 | 0.84489873 | 0.945963967 |  |  |  |  |  |  |  |  |
| Coprs | -0.142270093 | 0.377895221 | 0.693078766 |  |  |  |  |  |  |  |  |
| Cops2 | -0.02365862 | 0.994787947 | 1 |  |  |  |  |  |  |  |  |
| Cops3 | -0.011698506 | 0.987265426 | 0.996889358 | SYN |  |  |  |  |  |  |  |
| Cops4 | -0.083557648 | 0.619142315 | 0.848210083 |  |  |  |  |  |  |  |  |
| Cops5 | -0.141020271 | 0.112839394 | 0.35736989 |  |  |  |  |  |  |  |  |
| Cops6 | 0.070165484 | 0.372255474 | 0.687930875 |  |  |  |  |  |  |  |  |
| Cops7a | 0.057666103 | 0.269815376 | 0.588473459 |  |  |  |  |  |  |  |  |
| Cops7b | 0.088123446 | 0.647733042 | 0.864077395 |  |  |  |  |  |  |  |  |
| Cops8 | 0.053382637 | 0.288680239 | 0.607042864 |  |  |  |  |  |  |  |  |
| Copz1 | 0.047141174 | 0.555741431 | 0.81005001 |  |  |  |  |  |  |  |  |
| Coq10b | 0.01170773 | 0.815221133 | 0.938946391 | SYN |  |  |  |  |  | SZ_108 | SZ_full |
| Coq2 | -0.078547977 | 0.161630023 | 0.443925987 |  |  |  |  |  |  |  |  |
| Coq3 | -0.090662681 | 0.44715057 | 0.744686785 |  |  |  |  |  |  |  |  |
| Coq5 | 0.051370289 | 0.819854366 | 0.941021502 |  |  |  |  |  |  |  |  |
| Coq6 | 0.025677754 | 0.799456318 | 0.934965337 |  |  |  |  |  |  |  |  |
| Coq9 | -0.060633455 | 0.36424419 | 0.681175067 |  |  |  |  |  |  |  |  |
| Coro1a | 0.050912401 | 0.663559457 | 0.872925982 | SYN |  |  |  |  |  |  |  |
| Coro1b | 0.002231532 | 0.93586392 | 0.985275954 | SYN |  |  |  |  |  |  |  |
| Coro1c | 0.106578149 | 0.134567101 | 0.398998958 | SYN |  |  |  |  |  |  |  |
| Coro2b | -0.265694501 | 0.003809353 | 0.027007464 | SYN |  |  |  |  |  |  |  |
| Coro6 | -0.720102192 | 6.36993E-06 | 9.58614E-05 |  |  |  |  |  |  |  |  |
| Coro7 | 0.005620582 | 0.826563276 | 0.941950287 |  |  |  |  |  |  |  |  |
| Cotl1 | 0.158470704 | 0.170604629 | 0.45924486 |  |  |  |  |  |  |  |  |
| Cox10 | -0.06896991 | 0.479816762 | 0.766016512 |  | ID |  |  |  |  |  |  |
| Cox14 | -0.034909075 | 0.725894284 | 0.90084531 |  |  |  |  |  |  |  |  |
| Cox17 | 0.079044955 | 0.740483746 | 0.909890365 |  |  |  |  |  |  |  |  |
| Cox4i1 | 0.114462583 | 0.666897791 | 0.875092018 | SYN |  |  |  |  |  |  |  |
| Cox5a | -0.021051757 | 0.938496206 | 0.98530815 | SYN |  |  |  |  |  |  |  |
| Cox6a1 | 0.076163577 | 0.781332667 | 0.92656842 |  |  |  |  |  |  |  |  |
| Cox6c | -0.122202145 | 0.095257514 | 0.318964707 | SYN |  |  |  |  |  |  |  |
| Cox7a2 | -0.046425506 | 0.501857796 | 0.781827313 |  |  |  |  |  |  |  |  |
| Cox7a2l | 0.083807347 | 0.240622957 | 0.556292378 | SYN |  |  |  |  |  |  |  |
| Cox7b | 0.083609577 | 0.755134473 | 0.915759853 |  |  |  |  |  |  |  |  |
| Cox7c | 0.187879121 | 0.234594218 | 0.548716564 |  |  |  |  |  |  |  |  |
| Cox8a | 0.177409592 | 0.599125939 | 0.835219314 |  |  |  |  |  |  |  |  |
| Cpd | 0.079159914 | 0.339938862 | 0.660719834 |  |  |  |  |  |  |  |  |
| Cpe | 0.366633077 | 1.28486E-06 | 2.23268E-05 |  |  |  |  | FMRP |  |  |  |
| Cpeb1 | -0.068274708 | 0.454314279 | 0.750696227 |  |  |  |  |  |  |  |  |
| Cpeb3 | -0.058869395 | 0.591938953 | 0.83106937 | SYN |  |  |  |  |  |  |  |
| Cpeb4 | -0.355843334 | 0.000393496 | 0.003925653 |  |  |  |  |  |  |  |  |
| Cped1 | 0.14491071 | 0.348919083 | 0.668361715 |  |  |  |  |  |  |  |  |
| Cplx1 | -0.269259888 | 0.016332673 | 0.086635433 |  |  |  |  | FMRP |  |  |  |
| Cplx2 | 0.714497971 | 2.09558E-11 | 7.77412E-10 | SYN |  |  |  | FMRP | SZdb |  | SZ_full |
| Cplx3 | 0.242867195 | 0.070347411 | 0.259837869 |  |  |  |  |  |  |  |  |
| Cpne1 | 0.087928816 | 0.535112736 | 0.798215669 |  |  |  |  |  |  |  |  |
| Cpne3 | 0.044784852 | 0.674895212 | 0.878134455 |  |  |  |  |  |  |  |  |
| Cpne5 | -1.457681653 | 3.56085E-16 | 2.34722E-14 | SYN |  |  |  |  |  |  |  |
| Cpne6 | 0.610828942 | 0.000631387 | 0.005952653 | SYN |  |  |  |  |  |  |  |
| Cpne7 | 2.109162998 | 4.21764E-14 | 2.14267E-12 |  |  |  |  |  |  |  |  |
| Cpne8 | -0.08176538 | 0.371410212 | 0.687011124 |  |  |  |  |  |  |  |  |
| Cpne9 | -0.796394113 | 8.71141E-16 | 5.38622E-14 |  |  |  |  |  |  |  |  |
| Cpox | 0.118148815 | 0.237062147 | 0.551738456 |  |  |  |  |  |  |  |  |
| Cpped1 | -0.152004574 | 0.14495341 | 0.417887999 |  |  |  |  |  |  |  |  |
| Cpsf1 | -0.011911928 | 0.786297908 | 0.929112906 |  |  |  |  |  |  |  |  |
| Cpsf2 | 0.073834941 | 0.304362825 | 0.622062588 |  |  |  |  |  |  |  |  |
| Cpsf3 | -0.111981842 | 0.250690493 | 0.568337129 |  |  |  |  |  |  |  |  |
| Cpsf6 | 0.159739042 | 0.043197557 | 0.185311366 |  |  |  |  |  |  |  |  |
| Cpsf7 | -0.063509616 | 0.409026618 | 0.719062719 |  |  |  |  |  |  |  |  |
| Cpt1a | -0.026306742 | 0.883745106 | 0.961761627 | SYN |  |  |  |  |  |  |  |
| Cpt1c | 0.216515499 | 0.043214536 | 0.185311366 |  |  |  |  | FMRP |  |  |  |
| Cramp1l | -0.266859399 | 0.007304921 | 0.046058535 |  |  |  |  |  |  |  |  |
| Crat | 0.043507828 | 0.749337432 | 0.914010607 | SYN |  |  |  |  |  |  |  |
| Crbn | -0.051346037 | 0.971023236 | 0.99150896 |  |  |  |  |  |  |  |  |
| Creb1 | 0.017867244 | 0.646860672 | 0.863491334 |  |  |  |  |  |  |  |  |
| Creb3 | 0.061669026 | 0.530085444 | 0.796076351 |  |  |  |  |  |  |  |  |
| Crebbp | -0.08788467 | 0.510801638 | 0.786364382 |  | ID | ASD |  | FMRP |  |  |  |
| Crebl2 | -0.112023003 | 0.448251477 | 0.745627232 |  |  |  |  |  |  |  |  |
| Crebrf | -0.098943038 | 0.472222921 | 0.76369839 |  |  |  |  |  |  |  |  |
| Crebzf | 0.017031195 | 0.770438712 | 0.921167131 |  |  |  |  |  |  |  |  |
| Creg1 | -0.091987417 | 0.556248226 | 0.810114044 |  |  |  |  |  |  |  |  |
| Creg2 | -0.391605246 | 0.006643558 | 0.042733076 |  |  |  |  |  |  |  |  |
| Creld1 | 0.105424718 | 0.549272939 | 0.807161534 |  |  |  |  |  |  |  |  |
| Crim1 | 0.900130456 | 1.35859E-22 | 1.27483E-20 |  |  |  |  |  |  |  |  |
| Crip2 | 0.232036317 | 0.094023384 | 0.316827422 | SYN |  |  |  |  |  |  |  |
| Crk | -0.060126187 | 0.587025586 | 0.82825333 |  |  |  |  |  |  |  |  |
| Crkl | 0.333649464 | 0.000274128 | 0.00283586 | SYN |  |  |  |  |  |  |  |
| Crlf2 | 0.120743758 | 0.74809134 | 0.913109955 |  |  |  |  |  |  |  |  |
| Crlf3 | 0.005170106 | 0.972169638 | 0.991571804 |  |  |  |  |  |  |  |  |
| Crls1 | 0.126678195 | 0.164289639 | 0.448451117 |  |  |  |  |  |  |  |  |
| Crmp1 | 0.089795513 | 0.253831704 | 0.571210811 | SYN |  |  |  | FMRP |  |  |  |
| Crnkl1 | 0.053990215 | 0.470413569 | 0.762867484 |  |  |  |  |  |  |  |  |
| Crocc | 0.227628637 | 0.065571005 | 0.247629894 |  |  |  |  |  |  |  |  |
| Crot | -0.23746241 | 0.031399544 | 0.144264263 |  |  |  |  |  |  |  |  |
| Crtac1 | 0.195147811 | 0.018915156 | 0.097522483 | SYN |  |  |  |  |  |  |  |
| Crtc1 | 0.1252967 | 0.170669395 | 0.459264201 |  |  |  |  | FMRP |  |  |  |
| Cry1 | -0.014194985 | 0.703424813 | 0.888798685 |  |  |  |  |  |  |  |  |
| Cry2 | -0.003042841 | 0.826623616 | 0.941950287 |  |  |  |  |  |  |  |  |
| Cryab | 0.098000574 | 0.539821967 | 0.802239613 | SYN |  |  |  |  |  |  |  |
| Cryl1 | -0.195210642 | 0.07394737 | 0.267727747 |  |  |  |  |  |  |  |  |
| Crym | 0.171855193 | 0.418089453 | 0.725719582 | SYN |  |  |  |  |  |  |  |
| Cryzl1 | -0.10541826 | 0.210027994 | 0.517351229 |  |  |  |  |  |  |  |  |
| Cs | 0.007357472 | 0.754300247 | 0.915220708 | SYN |  |  |  |  |  |  |  |
| Csad | -0.126652822 | 0.259658751 | 0.576917826 |  |  |  |  |  |  |  |  |
| Csda | 0.147961476 | 0.260621913 | 0.578386304 |  |  |  |  |  |  |  |  |
| Csdc2 | -0.202357971 | 0.023256139 | 0.114359413 |  |  |  |  |  |  |  |  |
| Csde1 | 0.055614088 | 0.417148488 | 0.724559307 |  |  |  |  |  |  |  |  |
| Cse1l | -0.150941123 | 0.138832264 | 0.406713271 | SYN |  |  |  |  |  |  |  |
| Csf1r | -0.188135707 | 0.039923245 | 0.174099402 |  |  |  |  |  |  |  |  |
| Csgalnact1 | -0.043678994 | 0.524996428 | 0.793545744 |  |  |  |  |  |  |  |  |
| Csgalnact2 | 0.14443288 | 0.291874644 | 0.608837802 |  |  |  |  |  |  |  |  |
| Csk | 0.060067831 | 0.628050764 | 0.852766489 |  |  |  |  |  |  |  |  |
| Csmd1 | -0.233278474 | 0.222113844 | 0.534091052 |  |  | ASD |  |  |  | SZ_108 | SZ_full |
| Csmd3 | 0.381994474 | 0.000393746 | 0.003925653 |  |  |  |  |  |  |  |  |
| Csnk1a1 | 0.038889671 | 0.575356901 | 0.821820674 |  |  |  |  |  |  |  |  |
| Csnk1d | 0.165334908 | 0.033943581 | 0.15313009 | SYN |  | ASD |  |  |  |  |  |
| Csnk1e | -0.076621644 | 0.307868481 | 0.624820652 | SYN |  |  |  |  |  |  |  |
| Csnk1g1 | -0.018136807 | 0.661990307 | 0.871599054 |  |  |  |  |  |  |  |  |
| Csnk1g2 | -0.018031156 | 0.860401567 | 0.952877083 |  |  |  |  |  |  |  |  |
| Csnk1g3 | -0.031567423 | 0.961643213 | 0.989984854 |  |  |  |  |  |  |  |  |
| Csnk2a1 | -0.135833496 | 0.11301667 | 0.357601697 | SYN |  |  |  |  |  |  |  |
| Csnk2b | 0.037828245 | 0.625160598 | 0.850614729 | SYN |  |  |  |  |  |  |  |
| Cspg5 | -0.166710254 | 0.045061976 | 0.191438681 | SYN |  |  |  |  |  |  |  |
| Cspp1 | -0.095251715 | 0.150994517 | 0.426634282 |  |  |  |  |  |  |  |  |
| Csrnp2 | -0.2549489 | 0.005230246 | 0.035114851 |  |  |  |  |  |  |  |  |
| Csrnp3 | -0.124782403 | 0.291884028 | 0.608837802 |  |  |  |  |  |  |  |  |
| Csrp1 | 0.066583366 | 0.705272792 | 0.889759184 | SYN |  |  |  |  |  |  |  |
| Csrp2bp | -0.04381962 | 0.97229424 | 0.991571804 |  |  |  |  |  |  |  |  |
| Cst3 | 0.270596264 | 0.08500867 | 0.295953362 |  |  |  |  |  |  |  |  |
| Cstf1 | 0.033451173 | 0.663962852 | 0.873312617 |  |  |  |  |  |  |  |  |
| Cstf2 | 0.090173657 | 0.380626017 | 0.695268093 |  |  |  |  |  |  |  |  |
| Cstf2t | 0.201876789 | 0.008703706 | 0.053033429 |  |  |  |  |  |  |  |  |
| Cstf3 | 0.024666109 | 0.845376125 | 0.946081097 |  |  |  |  |  |  |  |  |
| Ctage5 | 0.182315684 | 0.080265589 | 0.284912477 |  |  |  |  |  |  |  |  |
| Ctbp1 | -0.00465061 | 0.901698681 | 0.969134709 | SYN |  |  |  | FMRP |  |  |  |
| Ctc1 | -0.106513862 | 0.270278086 | 0.588732633 |  |  |  |  |  |  |  |  |
| Ctcf | -0.013170316 | 0.920433681 | 0.97728688 |  |  | ASD | ASD_sc |  |  |  |  |
| Ctcfl | -0.064455304 | 0.844069212 | 0.945963967 |  |  |  |  |  |  |  |  |
| Ctdsp1 | 0.029545837 | 0.473736432 | 0.76408458 |  |  |  |  |  |  |  |  |
| Ctdsp2 | 0.134207861 | 0.151209799 | 0.426634282 |  |  |  |  |  |  |  |  |
| Ctdspl | -0.134847441 | 0.077723212 | 0.277742088 |  |  |  |  |  |  |  |  |
| Ctdspl2 | -0.029492546 | 0.881768565 | 0.960863288 |  |  |  |  |  |  |  |  |
| Ctgf | 0.80469895 | 0.048927741 | 0.203042489 |  |  |  |  |  |  |  |  |
| Ctif | -0.034467673 | 0.65976646 | 0.870520642 |  |  |  |  |  |  |  |  |
| Ctla2a | -0.086870809 | 0.350344798 | 0.669369249 |  |  |  |  |  |  |  |  |
| Ctnna1 | -0.006896107 | 0.757664835 | 0.916884789 | SYN |  |  |  |  |  | SZ_108 | SZ_full |
| Ctnna2 | -0.335886911 | 0.000140762 | 0.001561497 | SYN |  |  |  |  |  |  |  |
| Ctnnal1 | -0.243358679 | 0.010029907 | 0.059522721 |  |  |  |  |  |  |  |  |
| Ctnnb1 | -0.107095862 | 0.501361617 | 0.781638049 | SYN |  | ASD | ASD_sc | FMRP |  |  |  |
| Ctnnbip1 | -0.196860875 | 0.059047109 | 0.230073152 |  |  |  |  |  |  |  |  |
| Ctnnd1 | 0.197310152 | 0.025621383 | 0.122810187 | SYN |  |  |  |  |  | SZ_108 | SZ_full |
| Ctnnd2 | -0.339805692 | 5.76274E-05 | 0.000702808 | SYN |  |  |  | FMRP |  |  |  |
| Ctns | 0.170591502 | 0.399500231 | 0.709407276 |  |  |  |  |  |  |  |  |
| Ctps | -0.067192468 | 0.532325906 | 0.797338264 |  |  |  |  |  |  |  |  |
| Ctps2 | -0.42640791 | 6.49507E-05 | 0.000779017 |  |  |  |  |  |  |  |  |
| Ctr9 | 0.149233654 | 0.124641986 | 0.380550097 |  |  |  |  |  |  |  |  |
| Ctsa | 0.074952915 | 0.615089197 | 0.845924204 |  | ID |  |  |  |  |  |  |
| Ctsb | 0.01979264 | 0.680514699 | 0.878917067 |  |  |  |  |  |  |  |  |
| Ctsd | 0.060246214 | 0.277087609 | 0.59394256 |  |  |  |  |  |  |  |  |
| Ctse | 0.291594923 | 0.004197499 | 0.029342028 |  |  |  |  |  |  |  |  |
| Ctsl | 0.257040948 | 0.005324753 | 0.035629216 |  |  |  |  |  |  |  |  |
| Ctss | 0.068989722 | 0.631581997 | 0.854727738 |  |  |  |  |  |  |  |  |
| Ctsz | 0.248421608 | 0.040023474 | 0.174441108 |  |  |  |  |  |  |  |  |
| Cttn | -0.070374687 | 0.544054366 | 0.804417173 | SYN |  |  |  |  |  |  |  |
| Cttnbp2 | -0.014152445 | 0.844775784 | 0.945963967 | SYN |  | ASD | ASD_sc |  |  |  |  |
| Cttnbp2nl | -0.027464786 | 0.660001078 | 0.870542186 |  |  |  |  |  |  |  |  |
| Ctxn1 | 0.340825228 | 0.057697173 | 0.226250076 |  |  |  |  |  |  |  |  |
| Cuedc1 | 0.167503511 | 0.121368533 | 0.374481787 |  |  |  |  |  |  |  |  |
| Cuedc2 | 0.044397597 | 0.697783436 | 0.886511738 |  |  |  |  |  |  |  |  |
| Cul1 | -0.132506762 | 0.133558527 | 0.397189712 |  |  |  |  |  |  |  |  |
| Cul2 | 0.01176328 | 0.902969791 | 0.969220665 |  |  |  |  |  |  |  |  |
| Cul3 | 0.013227776 | 0.656126089 | 0.869611321 |  |  | ASD | ASD_sc |  |  | SZ_108 | SZ_full |
| Cul4a | 0.062347827 | 0.59925473 | 0.835219314 |  |  |  |  |  |  |  |  |
| Cul4b | 0.160071078 | 0.04706531 | 0.197575218 |  | ID |  |  |  |  |  |  |
| Cul7 | -0.155194996 | 0.293562833 | 0.6107087 |  |  |  |  |  |  |  |  |
| Cul9 | -0.183191009 | 0.037627519 | 0.165902209 |  |  |  |  |  |  |  |  |
| Cuta | 0.090101628 | 0.696850338 | 0.886206371 |  |  |  |  |  |  |  |  |
| Cux1 | -0.723573109 | 5.11027E-16 | 3.26076E-14 |  |  |  |  | FMRP |  |  |  |
| Cux2 | -1.750079874 | 1.32605E-67 | 2.11532E-64 |  |  |  |  | FMRP |  |  |  |
| Cwc15 | 0.089104298 | 0.472437932 | 0.76369839 |  |  |  |  |  |  |  |  |
| Cwc22 | 0.008079682 | 0.996457012 | 1 |  |  |  |  |  |  |  |  |
| Cwc27 | -0.186389923 | 0.102413596 | 0.334817079 |  |  |  |  |  |  |  |  |
| Cwf19l2 | -0.13246859 | 0.183897727 | 0.478554085 |  |  |  |  |  |  |  |  |
| Cx3cl1 | -0.190112063 | 0.050061453 | 0.206144108 |  |  |  |  |  |  |  |  |
| Cx3cr1 | -0.243551938 | 0.023881495 | 0.116572097 |  |  | ASD |  |  |  |  |  |
| Cxcl12 | 0.096834797 | 0.34412684 | 0.66472929 |  |  |  |  |  |  |  |  |
| Cxcl14 | 0.511352292 | 0.000293051 | 0.002996639 |  |  |  |  |  |  |  |  |
| Cxcr2 | -0.095409822 | 0.40925733 | 0.719062719 |  |  |  |  |  |  |  |  |
| Cxcr7 | 0.287753543 | 0.026001543 | 0.123813916 |  |  |  |  |  |  |  |  |
| Cxx1a | -0.150165179 | 0.065697138 | 0.247637225 |  |  |  |  |  |  |  |  |
| Cxx1b | -0.087708099 | 0.271356792 | 0.589578255 |  |  |  |  |  |  |  |  |
| Cxx1c | -0.122344674 | 0.224257814 | 0.536818826 |  |  |  |  |  |  |  |  |
| Cxxc5 | -0.088543356 | 0.368177316 | 0.684543508 |  |  |  |  |  |  |  |  |
| Cyb561 | -0.191692586 | 0.063649215 | 0.243018736 |  |  |  |  |  |  |  |  |
| Cyb561d1 | -0.129484807 | 0.176969222 | 0.467231551 |  |  |  |  |  |  |  |  |
| Cyb5b | 0.061156296 | 0.385764752 | 0.698492545 | SYN |  |  |  |  |  |  |  |
| Cyb5d1 | 0.3278225 | 0.023251287 | 0.114359413 |  |  |  |  |  |  |  |  |
| Cyb5d2 | 0.063883138 | 0.504445456 | 0.782850979 |  |  |  |  |  |  |  |  |
| Cyb5r1 | 0.35503302 | 0.012796383 | 0.071473357 | SYN |  |  |  |  |  |  |  |
| Cyb5r3 | 0.159917782 | 0.096301519 | 0.321112424 |  | ID |  |  |  |  |  |  |
| Cyb5r4 | -0.065157714 | 0.706022786 | 0.890016189 |  |  |  |  |  |  |  |  |
| Cybasc3 | -0.076822017 | 0.700037169 | 0.887191673 |  |  |  |  |  |  |  |  |
| Cyc1 | 0.118690353 | 0.166571093 | 0.451925141 | SYN |  |  |  |  |  |  |  |
| Cycs | 0.05905076 | 0.583945062 | 0.826685448 | SYN |  |  |  |  |  |  |  |
| Cyfip1 | 0.206359281 | 0.048719308 | 0.202493593 | SYN |  | ASD |  |  |  |  |  |
| Cyfip2 | -0.228376913 | 0.010625076 | 0.062080966 | SYN |  |  |  | FMRP |  |  |  |
| Cygb | 0.189859634 | 0.074699985 | 0.269840165 |  |  |  |  |  |  |  |  |
| Cyhr1 | -0.064461365 | 0.368847526 | 0.685281419 |  |  |  |  |  |  |  |  |
| Cyld | 0.079931218 | 0.194694706 | 0.495712359 | SYN |  |  |  |  |  |  |  |
| Cyp20a1 | 0.083946446 | 0.709929842 | 0.893043852 |  |  |  |  |  |  |  |  |
| Cyp2j6 | 0.263721754 | 0.025283519 | 0.121733206 |  |  |  |  |  |  |  |  |
| Cyp46a1 | -0.322563524 | 0.000582479 | 0.005537436 |  |  |  |  |  |  |  |  |
| Cyp4f13 | 0.016653139 | 0.766570265 | 0.920286747 |  |  |  |  |  |  |  |  |
| Cyp4f15 | -0.058269333 | 0.655801474 | 0.86959601 |  |  |  |  |  |  |  |  |
| Cyp51 | 0.100059563 | 0.254068883 | 0.571324767 |  |  |  |  |  |  |  |  |
| Cys1 | 0.998903535 | 7.18375E-13 | 3.04774E-11 |  |  |  |  |  |  |  |  |
| Cystm1 | -0.030356765 | 0.580416265 | 0.824617053 |  |  |  |  |  |  |  |  |
| Cyth1 | -0.312016598 | 0.000544585 | 0.00521442 |  |  |  |  |  |  |  |  |
| Cyth2 | 0.001917022 | 0.985972822 | 0.996214749 | SYN |  |  |  |  |  |  |  |
| Cyth3 | -0.270993648 | 0.021140557 | 0.106182945 | SYN |  |  |  |  |  |  |  |
| D10Bwg1379e | -0.373430424 | 0.001053897 | 0.009339865 |  |  |  |  | FMRP |  |  |  |
| D10Jhu81e | 0.191783009 | 0.107153271 | 0.346052663 |  |  |  |  |  |  |  |  |
| D10Wsu52e | 0.016211109 | 0.836946008 | 0.943363721 |  |  |  |  |  |  |  |  |
| D130017N08Rik | -0.16899724 | 0.210759416 | 0.518399997 |  |  |  |  |  |  |  |  |
| D130043K22Rik | -0.030582984 | 0.424228821 | 0.729913577 |  |  |  |  |  |  |  |  |
| D14Abb1e | 0.008607552 | 0.608988626 | 0.841605715 |  |  |  |  |  |  |  |  |
| D15Ertd621e | -0.154578082 | 0.116342573 | 0.363758669 |  |  |  |  | FMRP |  |  |  |
| D17Wsu92e | -0.193081577 | 0.034523021 | 0.154868174 |  |  |  |  |  |  |  |  |
| D19Bwg1357e | -0.068491239 | 0.746753914 | 0.912020725 |  |  |  |  |  |  |  |  |
| D1Ertd622e | 0.236079027 | 0.019439083 | 0.099708119 |  |  |  |  |  |  |  |  |
| D2hgdh | -0.078340156 | 0.572665406 | 0.820827732 |  |  |  |  |  |  |  |  |
| D2Wsu81e | 0.150973872 | 0.274538077 | 0.592033284 |  |  |  |  |  |  |  |  |
| D330041H03Rik | 1.345629823 | 7.75986E-06 | 0.000114193 |  |  |  |  |  |  |  |  |
| D3Bwg0562e | 0.030891356 | 0.360220386 | 0.677462343 |  |  |  |  | FMRP |  |  |  |
| D3Ertd254e | 0.054226759 | 0.727906425 | 0.90219611 |  |  |  |  |  |  |  |  |
| D430019H16Rik | 0.343283321 | 0.01987482 | 0.101162453 |  |  |  |  |  |  |  |  |
| D430041D05Rik | -0.298614143 | 0.002072878 | 0.016616357 |  |  |  |  | FMRP |  |  |  |
| D4Wsu53e | -0.013820882 | 0.687483676 | 0.882484918 |  |  |  |  |  |  |  |  |
| D5Ertd579e | -0.05420331 | 0.755390711 | 0.915931333 |  |  |  |  |  |  |  |  |
| D630037F22Rik | -0.029488353 | 0.758912156 | 0.917690018 |  |  |  |  |  |  |  |  |
| D630045J12Rik | 0.194978717 | 0.030887273 | 0.142238389 | SYN |  |  |  |  |  |  |  |
| D6Wsu163e | -0.019898945 | 0.900871181 | 0.968898131 |  |  |  |  |  |  |  |  |
| D7Ertd715e | 0.02429314 | 0.894232111 | 0.965280668 |  |  |  |  |  |  |  |  |
| D8Ertd82e | -0.055744927 | 0.985175431 | 0.995925857 |  |  |  |  |  |  |  |  |
| D930015E06Rik | -0.122558321 | 0.238204031 | 0.553100475 |  |  |  |  |  |  |  |  |
| D930016D06Rik | -0.144337971 | 0.223908518 | 0.536465706 |  |  |  |  |  |  |  |  |
| Daam1 | 0.049513598 | 0.728000772 | 0.90219611 | SYN |  |  |  |  | SZdb |  | SZ_full |
| Daam2 | -0.142858883 | 0.162446938 | 0.445185011 | SYN |  |  |  |  | SZdb |  | SZ_full |
| Dab1 | -0.00191823 | 0.629971746 | 0.853812312 |  |  | ASD |  |  |  |  |  |
| Dab2ip | -0.106514302 | 0.246951731 | 0.563895507 | SYN |  |  |  | FMRP |  |  |  |
| Dact2 | -1.280879549 | 1.04435E-21 | 9.05407E-20 |  |  |  |  |  |  |  |  |
| Dact3 | -0.185096156 | 0.028968523 | 0.134980166 |  |  |  |  |  |  |  |  |
| Dad1 | 0.283923351 | 0.12490088 | 0.380977866 | SYN |  |  |  |  |  |  |  |
| Dag1 | -0.059827747 | 0.818860923 | 0.941021502 |  |  |  |  |  |  |  |  |
| Dagla | 0.060513841 | 0.532500211 | 0.797338264 |  |  | ASD |  | FMRP |  |  |  |
| Daglb | 0.008346877 | 0.732280313 | 0.905497313 |  |  |  |  |  |  |  |  |
| Dak | -0.109280092 | 0.396692085 | 0.707042696 |  |  |  |  |  |  |  |  |
| Dalrd3 | 0.42706985 | 0.011968597 | 0.067944151 |  |  |  |  |  |  |  |  |
| Dand5 | 0.154660144 | 0.585292967 | 0.827439595 |  |  |  |  |  |  |  |  |
| Dap | 0.286397118 | 0.150167831 | 0.425938343 |  |  |  |  |  |  |  |  |
| Dap3 | 0.095912962 | 0.316472973 | 0.633740504 |  |  |  |  |  |  |  |  |
| Dapk1 | 0.053479781 | 0.357987539 | 0.675972682 |  |  | ASD |  | FMRP |  |  |  |
| Dapk3 | 0.132335769 | 0.453456123 | 0.750292953 |  |  |  |  |  |  |  |  |
| Dazap2 | -0.156594039 | 0.205116326 | 0.51053825 |  |  |  |  |  |  |  |  |
| Dbc1 | -0.252648018 | 0.00473692 | 0.032458486 |  |  |  |  | FMRP |  |  |  |
| Dbi | 0.257225247 | 0.502894673 | 0.782106171 |  |  |  |  |  |  |  |  |
| Dbn1 | -0.034997899 | 0.829577566 | 0.942131349 | SYN |  |  |  |  |  |  |  |
| Dbndd2 | -0.052055518 | 0.671798752 | 0.876747282 |  |  |  |  |  |  |  |  |
| Dbnl | 0.131278251 | 0.134739223 | 0.399212498 | SYN |  |  |  |  |  |  |  |
| Dbp | 0.201911345 | 0.057869979 | 0.226628753 |  |  |  |  |  |  |  |  |
| Dbpht2 | -0.756404433 | 2.99662E-09 | 8.04749E-08 |  |  |  |  |  |  |  |  |
| Dbr1 | 0.125290329 | 0.389365624 | 0.700594 |  |  |  |  |  |  |  |  |
| Dbt | 0.138842838 | 0.484626467 | 0.769689507 | SYN | ID |  |  |  |  |  |  |
| Dbx2 | 0.133560571 | 0.489766863 | 0.77362416 |  |  |  |  |  |  |  |  |
| Dcaf10 | 0.028923232 | 0.93852922 | 0.98530815 |  |  |  |  |  |  |  |  |
| Dcaf11 | -0.076158959 | 0.290588035 | 0.607744642 |  |  |  |  |  |  |  |  |
| Dcaf12 | 0.093367987 | 0.349494807 | 0.668802922 |  |  |  |  |  |  |  |  |
| Dcaf13 | 0.045664787 | 0.746791293 | 0.912020725 |  |  |  |  |  |  |  |  |
| Dcaf15 | -0.062527366 | 0.64113472 | 0.860142008 |  |  |  |  |  |  |  |  |
| Dcaf5 | -0.18412594 | 0.103557294 | 0.337634538 |  |  |  |  |  |  |  |  |
| Dcaf6 | -0.04671324 | 0.874253012 | 0.958362015 |  |  |  |  |  |  |  |  |
| Dcaf7 | 0.081825308 | 0.222791872 | 0.534754129 |  |  |  |  |  |  |  |  |
| Dcaf8 | -0.054576483 | 0.469767488 | 0.762409271 |  |  |  |  |  |  |  |  |
| Dcbld1 | 1.131607632 | 1.61024E-20 | 1.25914E-18 |  |  |  |  |  |  |  |  |
| Dcbld2 | -0.962438762 | 3.49678E-19 | 2.65622E-17 |  |  |  |  |  |  |  |  |
| Dcc | 0.313047663 | 0.115947832 | 0.363140842 |  |  |  |  |  |  |  |  |
| Dclk1 | 0.179126222 | 0.012113393 | 0.068522283 | SYN |  |  |  | FMRP |  |  |  |
| Dclk3 | 0.008849433 | 0.683652846 | 0.88111717 |  |  |  |  |  |  |  |  |
| Dclre1a | -0.462419504 | 0.000499943 | 0.004827532 |  |  |  |  |  |  |  |  |
| Dclre1c | 0.067673077 | 0.913608446 | 0.974320225 |  |  |  |  |  |  |  |  |
| Dcn | 2.186633604 | 0.008726522 | 0.053131859 |  |  |  |  |  |  |  |  |
| Dcp2 | -0.178946087 | 0.036162694 | 0.160687269 |  |  |  |  |  |  |  |  |
| Dctn1 | 0.139907282 | 0.082268327 | 0.289700739 | SYN |  |  |  | FMRP |  |  |  |
| Dctn2 | -0.013982452 | 0.847271708 | 0.947139334 | SYN |  |  |  |  |  |  |  |
| Dctn3 | -0.082522691 | 0.358786482 | 0.676840346 | SYN |  |  |  |  |  |  |  |
| Dctn4 | 0.073463586 | 0.26455261 | 0.58143683 | SYN |  |  |  |  |  |  |  |
| Dctn6 | 0.066490634 | 0.780387253 | 0.926216702 |  |  |  |  |  |  |  |  |
| Dcun1d1 | -0.023676315 | 0.922373655 | 0.978044705 |  |  | ASD |  |  |  |  |  |
| Dcun1d2 | -0.033506173 | 0.422119176 | 0.729539014 |  |  |  |  |  |  |  |  |
| Dcun1d3 | -0.035564767 | 0.835409332 | 0.943188599 |  |  |  |  |  |  |  |  |
| Dcun1d4 | 0.196712439 | 0.023938961 | 0.116781133 |  |  |  |  |  |  |  |  |
| Dda1 | 0.138884675 | 0.373757626 | 0.689428961 |  |  |  |  |  |  |  |  |
| Ddb1 | 0.004390798 | 0.833764582 | 0.943106438 |  |  |  |  |  |  |  |  |
| Ddhd1 | 0.06567205 | 0.334766456 | 0.655399423 |  |  |  |  |  |  |  |  |
| Ddit3 | -0.150317353 | 0.379179653 | 0.693655256 |  |  |  |  |  |  |  |  |
| Ddit4l | -1.076811699 | 1.78344E-21 | 1.51326E-19 |  |  |  |  |  |  |  |  |
| Ddn | 0.320658776 | 3.36628E-05 | 0.00043097 |  |  |  |  | FMRP |  |  |  |
| Ddost | 0.1187066 | 0.207582122 | 0.513865613 | SYN |  |  |  |  |  |  |  |
| Ddr1 | 0.3376654 | 0.025554447 | 0.122563002 | SYN |  |  |  |  | SZdb |  | SZ_full |
| Ddr2 | 0.009815869 | 0.596725717 | 0.834708918 |  |  |  |  |  |  |  |  |
| Ddt | 0.246687067 | 0.183889362 | 0.478554085 |  |  |  |  |  |  |  |  |
| Ddx1 | -0.091188588 | 0.40151803 | 0.711185063 | SYN |  |  |  |  |  |  |  |
| Ddx17 | -0.037685147 | 0.539085438 | 0.801686963 | SYN |  |  |  |  |  |  |  |
| Ddx19b | 0.125135626 | 0.538808956 | 0.801497271 |  |  |  |  |  |  |  |  |
| Ddx23 | -0.049369101 | 0.718290051 | 0.897786272 |  |  |  |  |  |  |  |  |
| Ddx24 | 0.031397209 | 0.612643802 | 0.84438344 |  |  |  |  | FMRP |  |  |  |
| Ddx26b | -0.072230882 | 0.398295035 | 0.708790986 |  |  |  |  |  |  |  |  |
| Ddx27 | 0.011519914 | 0.942884358 | 0.985900058 |  |  |  |  |  |  |  |  |
| Ddx39 | 0.134151824 | 0.510019484 | 0.786093307 |  |  |  |  |  |  |  |  |
| Ddx39b | 0.122640022 | 0.088135491 | 0.302697643 |  |  |  |  |  |  |  |  |
| Ddx3x | -0.141680918 | 0.249878555 | 0.567244294 | SYN |  |  |  |  |  |  |  |
| Ddx3y | 0.010113975 | 0.977772188 | 0.993086842 |  |  |  |  |  |  |  |  |
| Ddx41 | 0.056980155 | 0.742869132 | 0.910996956 |  |  |  |  |  |  |  |  |
| Ddx42 | 0.030796966 | 0.592072889 | 0.831111116 |  |  |  |  |  |  |  |  |
| Ddx46 | 0.104672359 | 0.319024537 | 0.636771698 |  |  |  |  |  |  |  |  |
| Ddx47 | 0.046504567 | 0.342169384 | 0.662914472 |  |  |  |  |  |  |  |  |
| Ddx5 | 0.047300454 | 0.336626306 | 0.657587905 | SYN |  |  |  |  |  |  |  |
| Ddx50 | 0.123700356 | 0.152571577 | 0.428338929 |  |  |  |  |  |  |  |  |
| Ddx52 | 0.069973527 | 0.607856593 | 0.841251463 |  |  |  |  |  |  |  |  |
| Ddx54 | 0.109762582 | 0.33560883 | 0.656404126 |  |  |  |  |  |  |  |  |
| Ddx6 | -0.012607232 | 0.752415605 | 0.91454844 | SYN |  |  |  |  |  |  |  |
| Deaf1 | 0.038371082 | 0.981643285 | 0.994569342 |  |  | ASD | ASD_sc |  |  |  |  |
| Deb1 | -0.028561444 | 0.876846517 | 0.959227516 |  |  |  |  |  |  |  |  |
| Decr2 | -0.093646663 | 0.309382342 | 0.626461934 | SYN |  |  |  |  |  |  |  |
| Dedd | 0.017186618 | 0.911118401 | 0.972729693 |  |  |  |  |  |  |  |  |
| Dedd2 | 0.197061619 | 0.401524312 | 0.711185063 |  |  |  |  |  |  |  |  |
| Def8 | 0.11677721 | 0.290777806 | 0.607768287 |  |  |  |  |  |  |  |  |
| Degs1 | 0.071147694 | 0.519940565 | 0.790843055 |  |  |  |  |  |  |  |  |
| Dek | -0.13172814 | 0.239488991 | 0.554531538 |  |  |  |  |  |  |  |  |
| Dennd1a | 0.024628007 | 0.894361101 | 0.965280668 |  |  |  |  |  |  |  |  |
| Dennd2a | 0.477587996 | 1.27598E-05 | 0.000178862 |  |  |  |  |  |  |  |  |
| Dennd3 | -0.560801011 | 0.069638853 | 0.258394715 |  |  |  |  |  |  |  |  |
| Dennd4a | -0.171151751 | 0.061205112 | 0.235945854 |  |  |  |  |  |  |  |  |
| Dennd4c | -0.157242596 | 0.149084657 | 0.423921291 |  |  |  |  |  |  |  |  |
| Dennd5a | -0.167621879 | 0.211665375 | 0.518502158 |  |  |  |  | FMRP |  |  |  |
| Dennd5b | -0.224536498 | 0.010527267 | 0.061648667 |  |  |  |  |  |  |  |  |
| Dennd6b | -0.110734283 | 0.190557745 | 0.48910346 |  |  |  |  |  |  |  |  |
| Denr | -0.158243079 | 0.151291885 | 0.426634282 |  |  |  |  |  |  |  |  |
| Depdc5 | 0.009956336 | 0.852521873 | 0.9494683 |  |  | ASD |  |  |  |  |  |
| Deptor | 1.04216567 | 1.79145E-12 | 7.2531E-11 |  |  |  |  |  |  |  |  |
| Desi1 | 0.00132063 | 0.973929759 | 0.991836537 |  |  |  |  |  |  |  |  |
| Desi2 | 0.052010469 | 0.906356647 | 0.970739161 |  |  |  |  |  |  |  |  |
| Dexi | -0.086081961 | 0.18615346 | 0.481595848 |  |  |  |  |  |  |  |  |
| Dffa | 0.037959425 | 0.834173377 | 0.943106438 |  |  |  |  |  |  |  |  |
| Dgat1 | -0.138169283 | 0.093430383 | 0.315228737 |  |  |  |  |  |  |  |  |
| Dgcr2 | 0.092780069 | 0.30714147 | 0.624472496 |  |  |  |  | FMRP | SZdb |  | SZ_full |
| Dgcr6 | -0.05468491 | 0.520056648 | 0.790843055 |  |  |  |  |  | SZdb |  | SZ_full |
| Dgcr8 | -0.048246564 | 0.428510776 | 0.73153195 |  |  |  |  |  |  |  |  |
| Dgkb | -0.075759004 | 0.891683204 | 0.964133804 | SYN |  |  |  |  |  |  |  |
| Dgkd | -0.161287147 | 0.075265827 | 0.270902635 |  |  |  |  |  |  |  |  |
| Dgke | -0.458584788 | 0.03677977 | 0.162919242 |  |  |  |  |  |  |  |  |
| Dgkg | 0.673374765 | 4.26141E-10 | 1.29236E-08 |  |  |  |  |  |  |  |  |
| Dgkh | -0.608909393 | 0.000126211 | 0.001415838 |  |  |  |  |  |  |  |  |
| Dgki | -0.12571428 | 0.420156313 | 0.727091941 |  |  |  |  |  |  | SZ_108 | SZ_full |
| Dgkq | -0.036012173 | 0.574981008 | 0.821430865 |  |  |  |  |  |  |  |  |
| Dgkz | 0.039620931 | 0.848649882 | 0.947617452 | SYN |  |  |  | FMRP |  | SZ_108 | SZ_full |
| Dguok | 0.051260796 | 0.965896597 | 0.990416798 |  |  |  |  |  |  |  |  |
| Dhcr24 | -0.245215541 | 0.025030018 | 0.121020878 |  |  |  |  |  |  |  |  |
| Dhdds | 0.02589614 | 0.959546059 | 0.989888316 |  |  |  |  |  |  |  |  |
| Dhps | 0.045233234 | 0.810891494 | 0.93712146 |  |  |  |  |  |  |  |  |
| Dhrs1 | 0.112928965 | 0.433287692 | 0.73436095 |  |  |  |  |  |  |  |  |
| Dhrs3 | -0.342592342 | 0.007919351 | 0.049308934 |  |  |  |  |  |  |  |  |
| Dhrs4 | 0.321432257 | 0.111238462 | 0.353936314 | SYN |  |  |  |  |  |  |  |
| Dhrs7 | 0.027068386 | 0.828300667 | 0.94210462 |  |  |  |  |  |  |  |  |
| Dhtkd1 | 0.063688644 | 0.89428839 | 0.965280668 |  |  |  |  |  |  |  |  |
| Dhx15 | 0.093589439 | 0.273799771 | 0.591359138 |  |  |  |  |  |  |  |  |
| Dhx30 | -0.083682977 | 0.378014789 | 0.693078766 | SYN |  |  |  | FMRP |  |  |  |
| Dhx32 | -0.101933821 | 0.437734557 | 0.737692921 |  |  |  |  |  |  |  |  |
| Dhx33 | -0.022287198 | 0.990802037 | 0.998311906 |  |  |  |  |  |  |  |  |
| Dhx34 | 0.049233957 | 0.907072791 | 0.971115782 |  |  |  |  |  |  |  |  |
| Dhx36 | 0.049308962 | 0.310179749 | 0.627296021 |  |  |  |  |  |  |  |  |
| Dhx37 | 0.105602444 | 0.416950223 | 0.724559307 |  |  |  |  |  |  |  |  |
| Dhx57 | 0.070450308 | 0.674577351 | 0.878031572 |  |  |  |  |  |  |  |  |
| Dhx8 | 0.035645734 | 0.723784531 | 0.899896975 |  |  |  |  |  |  |  |  |
| Dhx9 | 0.009423985 | 0.714122934 | 0.896126574 |  |  |  |  |  |  |  |  |
| Diap1 | -0.285643313 | 0.005063829 | 0.03419907 |  |  |  |  |  |  |  |  |
| Diap2 | -0.728749312 | 1.02403E-10 | 3.37353E-09 |  |  |  |  |  |  |  |  |
| Dido1 | -0.063102508 | 0.518820467 | 0.79062133 |  |  |  |  | FMRP |  |  |  |
| Diexf | -0.170126344 | 0.16209286 | 0.444890796 |  |  |  |  |  |  |  |  |
| Dip2a | 0.103187942 | 0.220966943 | 0.531974747 |  |  | ASD | ASD_sc | FMRP |  |  |  |
| Dip2b | -0.056373227 | 0.45226974 | 0.749471134 | SYN |  |  |  | FMRP |  |  |  |
| Dip2c | 0.121335255 | 0.098150205 | 0.325778625 |  |  |  |  | FMRP |  |  |  |
| Diras2 | 0.457199101 | 3.0399E-05 | 0.000393607 | SYN |  |  |  | FMRP |  |  |  |
| Dirc2 | 0.042786513 | 0.671220744 | 0.87656975 |  |  |  |  |  |  |  |  |
| Dis3l | 0.004767003 | 0.751845355 | 0.91454844 |  |  |  |  |  |  |  |  |
| Dis3l2 | -0.29435894 | 0.002307649 | 0.018205551 |  |  |  |  |  |  |  |  |
| Disp2 | 0.246840607 | 0.001046628 | 0.009285769 |  |  |  |  | FMRP |  |  |  |
| Dixdc1 | 0.215904613 | 0.018230212 | 0.094602585 |  |  |  |  |  |  |  |  |
| Dkc1 | -0.078310666 | 0.777972967 | 0.924893782 |  | ID |  |  |  |  |  |  |
| Dkk3 | 1.068956297 | 2.52291E-30 | 3.79674E-28 |  |  |  |  |  |  |  |  |
| Dlat | 0.013549703 | 0.895124486 | 0.965827133 | SYN |  |  |  |  |  |  |  |
| Dlc1 | 0.180808991 | 0.220891864 | 0.531974747 |  |  |  |  | FMRP |  |  |  |
| Dld | 0.074748314 | 0.53176478 | 0.797247347 | SYN | ID |  |  |  |  |  |  |
| Dlg1 | -0.218813695 | 0.026336037 | 0.125005428 | SYN |  |  |  |  |  |  |  |
| Dlg2 | -0.165107619 | 0.016480305 | 0.087282146 | SYN |  |  |  | FMRP |  |  |  |
| Dlg3 | 0.147299661 | 0.088483047 | 0.302893038 | SYN | ID |  |  |  |  |  |  |
| Dlg4 | -0.221696788 | 0.0157477 | 0.084354369 | SYN |  | ASD |  | FMRP |  |  |  |
| Dlgap1 | -0.1984004 | 0.00697121 | 0.044481897 | SYN |  |  |  | FMRP |  |  |  |
| Dlgap2 | -0.613689578 | 0.000105505 | 0.001205593 | SYN |  | ASD | ASD_sc | FMRP |  |  |  |
| Dlgap3 | -0.185008537 | 0.042961868 | 0.184686665 | SYN |  | ASD |  | FMRP |  |  |  |
| Dlgap4 | 0.04462752 | 0.856660475 | 0.95150034 | SYN |  |  |  | FMRP |  |  |  |
| Dlk2 | -0.163119433 | 0.113239766 | 0.357844839 |  |  |  |  |  |  |  |  |
| Dll4 | 0.077041415 | 0.661667252 | 0.871583757 |  |  |  |  |  |  |  |  |
| Dlst | 0.012703931 | 0.953794428 | 0.988881367 | SYN |  |  |  |  |  |  |  |
| Dlx2 | 0.249249349 | 0.174315099 | 0.463600276 |  |  | ASD | ASD_sc |  |  |  |  |
| Dmap1 | 0.150374249 | 0.308687448 | 0.625531271 |  |  |  |  |  |  |  |  |
| Dmd | 0.227292451 | 0.096224174 | 0.321028172 | SYN | ID | ASD | ASD_sc |  |  |  |  |
| Dmpk | 0.133226103 | 0.504593391 | 0.782850979 |  | ID | ASD | ASD_sc |  |  |  |  |
| Dmrtc1a | 0.218048576 | 0.440942207 | 0.740099967 |  |  |  |  |  |  |  |  |
| Dmtf1 | -0.069719048 | 0.657362158 | 0.869611321 |  |  |  |  |  |  |  |  |
| Dmwd | -0.023520824 | 0.919079204 | 0.976628795 | SYN |  |  |  | FMRP |  |  |  |
| Dmxl1 | 0.039535928 | 0.416458057 | 0.724350718 |  |  |  |  |  |  |  |  |
| Dmxl2 | -0.120184642 | 0.235538995 | 0.549804675 | SYN |  |  |  | FMRP |  |  |  |
| Dnahc1 | -0.426979128 | 0.19606137 | 0.497784221 |  |  |  |  |  |  |  |  |
| Dnaja1 | 0.086340806 | 0.394285352 | 0.705533207 | SYN |  |  |  |  |  |  |  |
| Dnaja2 | 0.054073256 | 0.323914495 | 0.64136382 | SYN |  |  |  |  |  |  |  |
| Dnaja3 | 0.039876738 | 0.620339827 | 0.84868447 | SYN |  |  |  |  |  |  |  |
| Dnaja4 | 0.014866116 | 0.877577926 | 0.959501239 |  |  |  |  |  |  |  |  |
| Dnajb1 | -0.127708291 | 0.202207766 | 0.505741342 | SYN |  |  |  |  |  |  |  |
| Dnajb11 | 0.158692125 | 0.208376176 | 0.515220745 |  |  |  |  |  |  |  |  |
| Dnajb12 | 0.074329297 | 0.433176125 | 0.73436095 |  |  |  |  |  |  |  |  |
| Dnajb14 | -0.034661897 | 0.729290645 | 0.902532534 |  |  |  |  |  |  |  |  |
| Dnajb2 | -0.187533575 | 0.051226608 | 0.208824185 | SYN |  |  |  |  |  |  |  |
| Dnajb4 | 0.122431129 | 0.391573728 | 0.703263241 | SYN |  |  |  |  |  |  |  |
| Dnajb5 | 0.095018275 | 0.227401553 | 0.540150216 |  |  |  |  |  |  |  |  |
| Dnajb6 | -0.076574089 | 0.395091003 | 0.70608242 |  |  |  |  |  |  |  |  |
| Dnajc10 | -0.167060892 | 0.088388015 | 0.302697643 |  |  |  |  |  |  |  |  |
| Dnajc11 | 0.075494593 | 0.406423302 | 0.716699592 | SYN |  |  |  |  |  |  |  |
| Dnajc13 | -0.308907161 | 0.000579573 | 0.005522906 | SYN |  |  |  |  |  |  |  |
| Dnajc15 | 0.082920242 | 0.570948775 | 0.820668125 |  |  |  |  |  |  |  |  |
| Dnajc18 | -0.011695703 | 0.941426365 | 0.985797123 |  |  |  |  |  |  |  |  |
| Dnajc19 | 0.077265446 | 0.864938189 | 0.954203365 | SYN |  | ASD |  |  |  | SZ_108 | SZ_full |
| Dnajc2 | -0.065908224 | 0.464877566 | 0.759024242 |  |  |  |  |  |  |  |  |
| Dnajc21 | -1.328893304 | 1.89125E-14 | 9.98982E-13 |  |  |  |  |  |  |  |  |
| Dnajc27 | -0.12810081 | 0.179092345 | 0.471152926 |  |  |  |  |  |  |  |  |
| Dnajc5 | -0.055778833 | 0.689412664 | 0.883476126 | SYN |  |  |  |  |  |  |  |
| Dnajc6 | -0.058100461 | 0.66502264 | 0.873985925 | SYN |  |  |  | FMRP |  |  |  |
| Dnajc7 | -0.016350726 | 0.707233351 | 0.890730001 |  |  |  |  |  |  |  |  |
| Dnajc8 | -0.079316261 | 0.379058197 | 0.693655256 |  |  |  |  |  |  |  |  |
| Dnajc9 | 0.201327636 | 0.066658838 | 0.250488431 |  |  |  |  |  |  |  |  |
| Dnalc1 | 0.070123859 | 0.264748384 | 0.58143683 |  |  |  |  |  |  |  |  |
| Dnalc4 | 0.116992942 | 0.39276367 | 0.704131946 |  |  |  |  |  |  |  |  |
| Dner | 0.257896527 | 0.019312102 | 0.099120542 |  |  | ASD |  |  |  |  |  |
| Dnlz | 0.178927376 | 0.259264575 | 0.576917826 |  |  |  |  |  |  |  |  |
| Dnm1 | 0.246199746 | 0.002787112 | 0.021147366 | SYN |  |  |  | FMRP |  |  |  |
| Dnm1l | 0.010315152 | 0.819797565 | 0.941021502 | SYN |  | ASD |  |  |  |  |  |
| Dnm2 | 0.031036017 | 0.926248288 | 0.979938499 | SYN |  |  |  |  |  |  |  |
| Dnm3 | -0.003316482 | 0.82822831 | 0.94210462 | SYN |  |  |  |  |  |  |  |
| Dnmbp | -0.415053761 | 0.001803892 | 0.014878843 |  |  |  |  |  |  |  |  |
| Dnmt1 | 0.083520564 | 0.352056203 | 0.670647307 |  |  |  |  |  |  |  |  |
| Dnmt3a | -0.246704095 | 0.026350129 | 0.125005428 |  |  |  |  |  |  |  |  |
| Dnpep | 0.068145425 | 0.504879099 | 0.783141909 |  |  |  |  |  |  |  |  |
| Doc2a | -1.469882121 | 2.44772E-41 | 8.48825E-39 | SYN |  |  |  |  |  | SZ_108 | SZ_full |
| Dock10 | 0.322255346 | 0.116098155 | 0.36327928 | SYN |  | ASD |  |  |  |  |  |
| Dock3 | -0.106139511 | 0.196155442 | 0.497784221 | SYN |  |  |  | FMRP |  |  |  |
| Dock4 | -0.307179245 | 0.001544055 | 0.013073653 | SYN |  | ASD |  | FMRP |  |  |  |
| Dock6 | 0.013863063 | 0.662113675 | 0.871599054 |  |  |  |  |  |  |  |  |
| Dock7 | -0.370801353 | 0.000114984 | 0.001293529 | SYN |  |  |  |  |  |  |  |
| Dock9 | 0.6765998 | 6.94161E-16 | 4.39415E-14 | SYN |  |  |  | FMRP |  |  |  |
| Dohh | 0.06357023 | 0.652785381 | 0.868058719 |  |  |  |  |  |  |  |  |
| Dok3 | 0.494720903 | 0.165431322 | 0.450445915 |  |  |  |  |  |  |  |  |
| Dok4 | 0.427729649 | 0.000282759 | 0.002906292 |  |  |  |  |  |  |  |  |
| Dok6 | 0.107025236 | 0.9670571 | 0.990416798 |  |  |  |  |  |  |  |  |
| Dolpp1 | -0.092109885 | 0.584831075 | 0.827352368 |  |  |  |  |  |  |  |  |
| Donson | 0.127917917 | 0.35157707 | 0.670296337 |  |  |  |  |  |  |  |  |
| Dopey1 | -0.026645228 | 0.825371357 | 0.941950287 |  |  |  |  | FMRP |  |  |  |
| Dopey2 | -0.022031637 | 0.677888041 | 0.878273493 |  |  |  |  | FMRP |  |  |  |
| Dos | 0.246367507 | 0.002224403 | 0.017671157 |  |  |  |  | FMRP |  |  |  |
| Dot1l | 0.101255059 | 0.570499623 | 0.82017033 |  |  |  |  | FMRP |  |  |  |
| Dpf1 | 0.474567899 | 4.81465E-06 | 7.39916E-05 |  |  |  |  |  |  |  |  |
| Dpf2 | 0.224624572 | 0.036498299 | 0.161998016 |  |  |  |  |  |  |  |  |
| Dpf3 | 0.090693737 | 1 | 1 |  |  |  |  |  |  |  |  |
| Dph5 | 0.040453051 | 0.705843113 | 0.889946992 |  |  |  |  |  |  |  |  |
| Dpm2 | 0.041442182 | 0.889259707 | 0.963245653 |  |  |  |  |  |  |  |  |
| Dpp10 | -0.903702103 | 2.10234E-23 | 2.04492E-21 | SYN |  | ASD | ASD_sc |  |  |  |  |
| Dpp6 | -0.091969092 | 0.467180601 | 0.760951676 | SYN |  | ASD | ASD_sc |  |  |  |  |
| Dpp7 | 0.10946779 | 0.434489698 | 0.735304441 |  |  |  |  |  |  |  |  |
| Dpp8 | -0.320594743 | 0.006991443 | 0.044558876 |  |  |  |  | FMRP |  |  |  |
| Dpp9 | 0.166407123 | 0.088895394 | 0.303783059 |  |  |  |  |  |  |  |  |
| Dpy19l1 | 0.388346798 | 1.52383E-06 | 2.58048E-05 |  |  |  |  |  |  |  |  |
| Dpy19l3 | -0.507221196 | 0.001370375 | 0.011740181 |  |  |  |  |  |  |  |  |
| Dpy19l4 | -0.2318976 | 0.03963492 | 0.173126025 |  |  |  |  |  |  |  |  |
| Dpy30 | 0.061703716 | 0.933853518 | 0.983678771 |  |  |  |  |  |  |  |  |
| Dpysl2 | 0.040262259 | 0.605850389 | 0.840393513 | SYN |  |  |  | FMRP | SZdb |  | SZ_full |
| Dpysl3 | 0.11797786 | 0.362907385 | 0.679847949 | SYN |  |  |  |  |  |  |  |
| Dpysl4 | -0.01808471 | 0.703633421 | 0.888802931 | SYN |  |  |  |  |  |  |  |
| Dpysl5 | 0.437019902 | 0.05935548 | 0.23082365 | SYN |  |  |  |  |  |  |  |
| Dr1 | -0.055210404 | 0.885376903 | 0.962224578 |  |  |  |  |  |  |  |  |
| Dram2 | 0.158906619 | 0.486904197 | 0.771519173 |  |  |  |  |  |  |  |  |
| Drap1 | 0.147933193 | 0.15247495 | 0.428338929 |  |  |  |  |  |  |  |  |
| Drd1a | 1.719857694 | 1.05408E-09 | 3.02422E-08 |  |  | ASD |  |  |  |  |  |
| Drg1 | -0.020080519 | 0.875815375 | 0.958877817 |  |  |  |  |  |  |  |  |
| Drg2 | 0.070196752 | 0.552286125 | 0.807970335 |  |  |  |  |  |  | SZ_108 | SZ_full |
| Drosha | -0.037092473 | 0.784162223 | 0.928282627 |  |  |  |  |  |  |  |  |
| Drp2 | -0.537734778 | 3.08466E-10 | 9.4628E-09 |  |  |  |  |  |  |  |  |
| Dscam | -0.142338799 | 0.072430206 | 0.264232227 |  |  | ASD | ASD_sc | FMRP |  |  |  |
| Dscaml1 | -0.058754572 | 0.783131428 | 0.927983401 |  |  |  |  | FMRP |  |  |  |
| Dscr3 | 0.090448461 | 0.553015288 | 0.808060922 |  |  |  |  |  |  |  |  |
| Dst | -0.048102572 | 0.480672684 | 0.766539151 | SYN |  | ASD |  | FMRP |  |  |  |
| Dstn | 0.05828179 | 0.381511138 | 0.695268093 | SYN |  |  |  |  |  |  |  |
| Dstyk | -0.036199046 | 0.944170733 | 0.985962641 |  |  |  |  |  |  |  |  |
| Dtd1 | 0.069042387 | 0.606207915 | 0.840653884 | SYN |  |  |  |  |  |  |  |
| Dtna | -0.370081881 | 1.70541E-05 | 0.000232122 | SYN |  |  |  | FMRP |  |  |  |
| Dtnb | 0.294475894 | 0.010632222 | 0.062080966 |  |  |  |  |  |  |  |  |
| Dtnbp1 | 0.049047524 | 0.506803338 | 0.784399099 |  |  |  |  |  | SZdb |  | SZ_full |
| Dtwd1 | -0.160014136 | 0.416988 | 0.724559307 |  |  |  |  |  |  |  |  |
| Dtx1 | 0.112806027 | 0.219408291 | 0.529180686 |  |  |  |  | FMRP |  |  |  |
| Dtx2 | 0.013396387 | 0.741788171 | 0.910790356 |  |  |  |  |  |  |  |  |
| Dtx3 | 0.164651793 | 0.132600334 | 0.395224313 |  |  |  |  |  |  |  |  |
| Dtx4 | 0.557427462 | 1.52797E-08 | 3.72693E-07 |  |  |  |  |  |  |  |  |
| Dtymk | 0.11680413 | 0.430206519 | 0.731710685 |  |  |  |  |  |  |  |  |
| Dus2l | -0.037537491 | 0.524254811 | 0.7932511 |  |  |  |  |  |  |  |  |
| Dus3l | 0.146963313 | 0.149075024 | 0.423921291 |  |  |  |  |  |  |  |  |
| Dusp1 | -0.341436724 | 0.132957654 | 0.395788702 |  |  |  |  |  |  |  |  |
| Dusp11 | -0.028678875 | 0.416001313 | 0.724350718 |  |  |  |  |  |  |  |  |
| Dusp12 | -0.31920837 | 0.326528864 | 0.643970048 |  |  |  |  |  |  |  |  |
| Dusp14 | -0.54986006 | 8.42199E-08 | 1.78803E-06 |  |  |  |  |  |  |  |  |
| Dusp15 | 0.093630091 | 0.646679877 | 0.863491334 |  |  |  |  |  |  |  |  |
| Dusp16 | -0.355076267 | 0.010086028 | 0.059766834 |  |  |  |  |  |  |  |  |
| Dusp22 | -0.09994877 | 0.194739834 | 0.495712359 |  |  | ASD |  |  |  |  |  |
| Dusp3 | -0.318266601 | 0.000340036 | 0.003428735 | SYN |  |  |  |  |  |  |  |
| Dusp4 | 0.008361314 | 0.938370802 | 0.98530815 | SYN |  |  |  |  |  |  |  |
| Dusp6 | -0.204654725 | 0.50645825 | 0.784371068 |  |  |  |  |  |  |  |  |
| Dusp7 | -0.103624919 | 0.305410164 | 0.62282745 |  |  |  |  |  |  |  |  |
| Dusp8 | -0.14275161 | 0.185449709 | 0.48118097 |  |  |  |  | FMRP |  |  |  |
| Dvl1 | 0.025695122 | 0.913975482 | 0.974441679 |  |  |  |  |  |  |  |  |
| Dvl3 | -0.066218252 | 0.369875924 | 0.685616352 |  |  |  |  |  |  |  |  |
| Dym | 0.070260953 | 0.475254311 | 0.764239594 |  |  |  |  |  |  |  |  |
| Dync1h1 | 0.101148828 | 0.17396994 | 0.462991072 | SYN |  |  |  | FMRP |  |  |  |
| Dync1i1 | 0.443206289 | 7.483E-08 | 1.61309E-06 | SYN |  |  |  |  |  |  |  |
| Dync1i2 | 0.038777808 | 0.816546267 | 0.939945551 | SYN |  |  |  |  |  |  |  |
| Dync1li1 | 0.107357774 | 0.162358006 | 0.445185011 | SYN |  |  |  |  |  |  |  |
| Dync1li2 | -0.038750096 | 0.763572158 | 0.919979858 | SYN |  |  |  |  |  |  |  |
| Dync2h1 | 0.042982982 | 0.656614645 | 0.869611321 |  |  |  |  |  |  |  |  |
| Dync2li1 | -0.168629182 | 0.137938027 | 0.405078683 |  |  |  |  |  |  |  |  |
| Dynll1 | 0.457588812 | 0.000481334 | 0.004687572 | SYN |  |  |  |  |  |  |  |
| Dynll2 | -0.21433645 | 0.013579247 | 0.074850086 | SYN |  |  |  |  |  |  |  |
| Dynlt1b | 0.656419551 | 5.72188E-06 | 8.65991E-05 |  |  |  |  |  |  |  |  |
| Dynlt1f | 0.493163862 | 8.15393E-05 | 0.000956408 |  |  |  |  |  |  |  |  |
| Dynlt3 | -0.081255256 | 0.402304097 | 0.711638386 |  |  |  |  |  |  |  |  |
| Dyrk1a | -0.146092525 | 0.225776122 | 0.538695632 |  | ID | ASD | ASD_sc |  |  |  |  |
| Dyrk2 | -0.019630868 | 0.808774286 | 0.93712146 |  |  |  |  |  |  |  |  |
| Dzank1 | 0.071793284 | 0.324496164 | 0.642069315 |  |  |  |  |  |  |  |  |
| Dzip1 | 0.445519478 | 8.90424E-07 | 1.58174E-05 |  |  |  |  |  |  |  |  |
| Dzip3 | -0.11204317 | 0.494481405 | 0.77668892 |  |  |  |  |  |  |  |  |
| E130012A19Rik | 0.14293953 | 0.19678745 | 0.498445165 |  |  |  |  |  |  |  |  |
| E130114P18Rik | 0.122815327 | 0.346080796 | 0.666324661 |  |  |  |  |  |  |  |  |
| E130308A19Rik | 0.243250286 | 0.033191287 | 0.150416876 |  |  |  |  |  |  |  |  |
| E130309D02Rik | 0.167567754 | 0.310572699 | 0.627438665 |  |  |  |  |  |  |  |  |
| E130309D14Rik | -0.053875641 | 0.734700814 | 0.90627493 |  |  |  |  |  |  |  |  |
| E130311K13Rik | -0.073109065 | 0.561065372 | 0.81394278 |  |  |  |  |  |  |  |  |
| E2f3 | -0.085834549 | 0.456464526 | 0.75237881 |  |  |  |  |  |  |  |  |
| E2f6 | -0.081641855 | 0.602089583 | 0.837095487 |  |  |  |  |  |  |  |  |
| E330009J07Rik | 0.12445286 | 0.130197229 | 0.390249192 |  |  |  |  |  |  |  |  |
| E430025E21Rik | -0.069174585 | 0.744574347 | 0.91183125 | SYN |  |  |  |  |  |  |  |
| Eaf1 | -0.106559678 | 0.483853946 | 0.768921911 |  |  |  |  |  |  |  |  |
| Ebf4 | 0.08731478 | 0.493701345 | 0.776678881 |  |  |  |  |  |  |  |  |
| Ebna1bp2 | -0.034555634 | 0.916164531 | 0.97483035 |  |  |  |  |  |  |  |  |
| Ecd | 0.059198585 | 0.534512432 | 0.798060053 |  |  |  |  |  |  |  |  |
| Ece1 | -0.349265224 | 0.000184824 | 0.001980088 |  |  |  |  |  |  |  |  |
| Ech1 | 0.222639232 | 0.016942557 | 0.089401884 |  |  |  |  |  |  |  |  |
| Echs1 | 0.115960045 | 0.201807932 | 0.505495724 |  |  |  |  |  |  |  |  |
| Eci1 | -0.092556802 | 0.562738998 | 0.815184571 |  |  |  |  |  |  |  |  |
| Ecm1 | -0.259845134 | 0.29980634 | 0.616620776 |  |  |  |  |  |  |  |  |
| Ecsit | 0.168147571 | 0.168031644 | 0.454620216 |  |  |  |  |  |  |  |  |
| Edc3 | 0.175417537 | 0.073585812 | 0.266903337 |  |  |  |  |  |  |  |  |
| Edc4 | 0.010456699 | 0.9521672 | 0.988280766 |  |  |  |  |  |  | SZ_108 | SZ_full |
| Edem2 | 0.163376189 | 0.179674201 | 0.471408363 |  |  |  |  |  |  |  |  |
| Edil3 | -0.091176352 | 0.567053153 | 0.818016992 |  |  |  |  |  |  |  |  |
| Ednrb | 0.381439481 | 0.002855352 | 0.021525792 |  |  |  |  |  |  |  |  |
| Eef1a1 | 0.2203526 | 0.002384194 | 0.018625199 | SYN |  |  |  |  |  |  |  |
| Eef1a2 | -0.030209045 | 0.587614968 | 0.828352242 | SYN |  | ASD |  | FMRP |  |  |  |
| Eef1b2 | 0.149161152 | 0.116780267 | 0.364555542 | SYN |  |  |  |  |  |  |  |
| Eef1d | 0.191856461 | 0.090330083 | 0.307368918 | SYN |  |  |  |  |  |  |  |
| Eef1g | -0.010685768 | 0.690720821 | 0.884120561 | SYN |  |  |  |  |  |  |  |
| Eef2 | 0.045915958 | 0.479783448 | 0.766016512 | SYN |  |  |  | FMRP |  |  |  |
| Eef2k | -0.059694483 | 0.395010428 | 0.70608242 |  |  |  |  |  |  |  |  |
| Eepd1 | -0.036738891 | 0.667773637 | 0.875092018 |  |  |  |  |  |  |  |  |
| Efcab14 | 0.053395818 | 0.430913042 | 0.732047375 |  |  |  |  |  |  |  |  |
| Efcab5 | -0.02278264 | 0.733197714 | 0.905793617 |  |  |  |  |  |  |  |  |
| Efha1 | -0.105073449 | 0.272940994 | 0.590927625 |  |  |  |  |  |  |  |  |
| Efha2 | -0.221158031 | 0.021487925 | 0.107723249 |  |  |  |  |  |  |  |  |
| Efhd2 | -0.540107763 | 1.39456E-09 | 3.9028E-08 | SYN |  |  |  |  |  |  |  |
| Efna3 | 0.458943762 | 9.41336E-05 | 0.001092882 |  |  |  |  |  |  |  |  |
| Efna5 | -2.144517106 | 2.01455E-40 | 6.69502E-38 |  |  |  |  |  |  |  |  |
| Efnb2 | 0.756917331 | 1.82254E-15 | 1.06887E-13 |  |  |  |  |  |  |  |  |
| Efnb3 | 0.452636897 | 0.003154099 | 0.023207652 |  |  |  |  |  |  |  |  |
| Efr3a | 0.398074425 | 1.25762E-06 | 2.19012E-05 | SYN |  | ASD |  |  |  |  |  |
| Efr3b | -0.390793336 | 1.73241E-05 | 0.000235395 | SYN |  |  |  |  |  |  |  |
| Eftud2 | -0.00969254 | 0.938497793 | 0.98530815 |  |  |  |  |  |  |  |  |
| Egfem1 | 0.780853817 | 0.000224756 | 0.002374379 |  |  |  |  |  |  |  |  |
| Egln1 | -0.284900139 | 0.004904646 | 0.033293156 |  |  |  |  |  |  |  |  |
| Egln3 | -0.277039418 | 0.147213641 | 0.421153516 |  |  |  |  |  |  |  |  |
| Egr1 | -0.312240849 | 0.51722172 | 0.789456636 |  |  |  |  | FMRP |  | SZ_108 | SZ_full |
| Egr3 | -0.230113666 | 0.395895538 | 0.706474453 |  |  |  |  |  | SZdb |  | SZ_full |
| Ehbp1 | -0.194027889 | 0.021313676 | 0.106916905 |  |  |  |  |  |  |  |  |
| Ehd1 | 0.070209094 | 0.42784712 | 0.73153195 | SYN |  |  |  |  |  |  |  |
| Ehd3 | 0.45786477 | 6.12379E-08 | 1.34926E-06 | SYN |  |  |  |  |  |  |  |
| Ehmt1 | -0.026215682 | 0.50230905 | 0.782106171 |  | ID | ASD | ASD_sc | FMRP |  |  |  |
| Ehmt2 | -0.104618919 | 0.25471352 | 0.57184254 | SYN |  |  |  | FMRP |  |  |  |
| Ei24 | -0.068787518 | 0.543007683 | 0.803828746 |  |  |  |  |  |  |  |  |
| Eid1 | 0.309249399 | 6.60524E-05 | 0.000791042 |  |  |  |  |  |  |  |  |
| Eid2b | -0.002891716 | 0.707245875 | 0.890730001 |  |  |  |  |  |  |  |  |
| Eif1 | -0.013502083 | 0.927999687 | 0.98101067 |  |  |  |  |  |  |  |  |
| Eif1a | 0.083995956 | 0.257680063 | 0.575233104 |  |  |  |  |  |  |  |  |
| Eif1ad | -0.030592764 | 0.864644301 | 0.954203365 |  |  |  |  |  |  |  |  |
| Eif1b | 0.808447691 | 1.42082E-08 | 3.49767E-07 |  |  |  |  |  |  |  |  |
| Eif2a | 0.04117161 | 0.726503758 | 0.901240272 |  |  |  |  |  |  |  |  |
| Eif2ak1 | 0.161104774 | 0.143460475 | 0.415181693 |  |  |  |  |  |  |  |  |
| Eif2ak2 | -0.042712915 | 0.881189737 | 0.960480474 | SYN |  |  |  |  |  |  |  |
| Eif2ak3 | -0.106583789 | 0.440603697 | 0.739687453 |  | ID |  |  |  |  |  |  |
| Eif2ak4 | -0.196922899 | 0.052661447 | 0.213212032 |  |  |  |  |  |  |  |  |
| Eif2b1 | 0.193258272 | 0.126775393 | 0.38477273 |  |  |  |  |  |  |  |  |
| Eif2b2 | 0.172535858 | 0.148406959 | 0.423078052 |  |  |  |  |  |  |  |  |
| Eif2b3 | 0.014816471 | 0.868256366 | 0.955465339 |  |  |  |  |  |  |  |  |
| Eif2b4 | 0.044654753 | 0.550536341 | 0.807666914 |  |  |  |  |  |  |  |  |
| Eif2b5 | -0.086091186 | 0.424135951 | 0.729913577 |  |  |  |  |  |  |  |  |
| Eif2d | -0.067934455 | 0.373615739 | 0.689326656 |  |  |  |  |  |  |  |  |
| Eif2s2 | 0.104827504 | 0.346673756 | 0.666442487 |  |  |  |  |  |  |  |  |
| Eif2s3x | -0.006268862 | 0.829371934 | 0.942131349 |  | ID |  |  |  |  |  |  |
| Eif3a | 0.164399908 | 0.049054975 | 0.203426471 |  |  |  |  |  |  |  |  |
| Eif3b | 0.149906925 | 0.160764155 | 0.442917039 |  |  |  |  |  |  |  |  |
| Eif3c | 0.061520949 | 0.384042922 | 0.697909852 | SYN |  |  |  |  |  |  |  |
| Eif3d | 0.084811811 | 0.328833715 | 0.646960462 |  |  |  |  |  |  |  |  |
| Eif3f | 0.100771974 | 0.345836509 | 0.666324661 |  |  |  |  |  |  |  |  |
| Eif3g | 0.185620994 | 0.204897442 | 0.510236534 |  |  |  |  |  |  |  |  |
| Eif3h | 0.024889273 | 0.715688381 | 0.896549478 |  |  |  |  |  |  |  |  |
| Eif3i | 0.176393589 | 0.044417527 | 0.189508635 |  |  |  |  |  |  |  |  |
| Eif3j1 | -0.018913189 | 0.817279608 | 0.940502402 |  |  |  |  |  |  |  |  |
| Eif3k | 0.179585297 | 0.059763823 | 0.231733717 |  |  |  |  |  |  |  |  |
| Eif3l | 0.156201572 | 0.141434448 | 0.411045522 |  |  |  |  |  |  |  |  |
| Eif3m | 0.049881674 | 0.443130434 | 0.742055079 |  |  |  |  |  |  |  |  |
| Eif4a1 | 0.218776928 | 0.005255696 | 0.03525604 |  |  |  |  |  |  |  |  |
| Eif4a2 | 0.031348732 | 0.473518023 | 0.76408458 | SYN |  |  |  |  |  |  |  |
| Eif4b | 0.142276758 | 0.069711409 | 0.258479285 | SYN |  |  |  |  |  |  |  |
| Eif4e | -0.183441265 | 0.085327022 | 0.296673202 | SYN |  | ASD | ASD_sc |  |  |  |  |
| Eif4e2 | 0.060383977 | 0.526784646 | 0.794238803 |  |  |  |  |  |  |  |  |
| Eif4enif1 | -0.076183048 | 0.659626284 | 0.87047969 |  |  |  |  |  |  |  |  |
| Eif4g1 | 0.129344826 | 0.156154906 | 0.434574854 | SYN |  |  |  | FMRP |  |  |  |
| Eif4g2 | -0.188335577 | 0.07847504 | 0.27967426 |  |  |  |  | FMRP |  |  |  |
| Eif4g3 | -0.202581744 | 0.008387273 | 0.051399981 |  |  |  |  | FMRP |  |  |  |
| Eif4h | -0.046944346 | 0.733288539 | 0.905793617 |  |  |  |  |  |  |  |  |
| Eif5 | -0.045441495 | 0.599571183 | 0.835461173 |  |  |  |  |  |  |  |  |
| Eif5a | 0.157276792 | 0.043041729 | 0.184769017 | SYN |  |  |  |  |  |  |  |
| Eif5a2 | 0.06334206 | 0.576737296 | 0.822172774 |  |  |  |  |  |  |  |  |
| Eif5b | 0.035025162 | 0.992658913 | 0.999551507 |  |  |  |  |  |  |  |  |
| Eif6 | 0.018234991 | 0.989217076 | 0.997735418 |  |  |  |  |  |  |  |  |
| Elac1 | -0.102569145 | 0.385663134 | 0.698492545 |  |  |  |  |  |  |  |  |
| Elavl1 | 0.046348246 | 0.399761928 | 0.709659723 |  |  |  |  |  |  |  |  |
| Elavl2 | -0.4071421 | 6.48114E-05 | 0.000778517 |  |  |  |  |  |  |  |  |
| Elavl3 | 0.038442492 | 0.824157383 | 0.941950287 |  |  |  |  |  |  |  |  |
| Elavl4 | -0.226687182 | 0.020015841 | 0.101731231 |  |  |  |  |  |  |  |  |
| Elf2 | -0.121077204 | 0.314653408 | 0.631791222 |  |  |  |  |  |  |  |  |
| Elfn2 | 0.353192771 | 6.65325E-05 | 0.000794406 | SYN |  |  |  | FMRP |  |  |  |
| Elk1 | -0.110561757 | 0.237926693 | 0.55290613 |  |  |  |  |  |  |  |  |
| Elk4 | 0.057569166 | 0.578573474 | 0.823895893 |  |  |  |  |  |  |  |  |
| Ell | -0.028661071 | 0.595648557 | 0.833964185 |  |  |  |  |  |  |  |  |
| Ell2 | -0.526608947 | 5.46549E-05 | 0.0006686 |  |  |  |  |  |  |  |  |
| Elmo1 | -0.699459532 | 1.25988E-13 | 5.84232E-12 |  |  |  |  |  |  |  |  |
| Elmo2 | -0.154993096 | 0.082479475 | 0.290060095 | SYN |  |  |  | FMRP |  |  |  |
| Elmod1 | -0.292719303 | 0.000596637 | 0.005645047 |  |  |  |  |  |  |  |  |
| Elmod2 | 0.06910892 | 0.571841522 | 0.820827732 |  |  |  |  |  |  |  |  |
| Elmsan1 | 0.089178301 | 0.357237113 | 0.675290951 |  |  |  |  |  |  |  |  |
| Elof1 | 0.153737926 | 0.395737372 | 0.706474453 |  |  |  |  |  |  |  |  |
| Elovl4 | 0.226874316 | 0.05652235 | 0.223179339 |  |  |  |  |  |  |  |  |
| Elovl5 | 0.03189169 | 0.496253075 | 0.77686598 |  |  |  |  |  |  |  |  |
| Elovl6 | -0.012443686 | 0.94668512 | 0.986189928 |  |  |  |  |  |  |  |  |
| Elp2 | -0.11739159 | 0.123946037 | 0.380022998 |  |  |  |  |  |  |  |  |
| Elp3 | -0.090546975 | 0.388136777 | 0.699457876 |  |  |  |  |  |  |  |  |
| Elp4 | -0.120440636 | 0.325849627 | 0.643489032 |  |  |  |  |  |  |  |  |
| Elp5 | -0.114819078 | 0.23834476 | 0.553100475 |  |  |  |  |  |  |  |  |
| Elp6 | -0.00391978 | 0.860616568 | 0.952877083 |  |  |  |  |  |  |  |  |
| Emb | -0.984564925 | 2.07907E-15 | 1.21041E-13 |  |  |  |  |  |  |  |  |
| Emc10 | 0.408982878 | 0.018258945 | 0.094690081 |  |  |  |  |  |  |  |  |
| Emc2 | 0.281268685 | 0.014915839 | 0.080602172 |  |  |  |  |  |  |  |  |
| Emc3 | 0.143711708 | 0.117943489 | 0.36618033 |  |  |  |  |  |  |  |  |
| Emc4 | -0.090341336 | 0.314673037 | 0.631791222 |  |  |  |  |  |  |  |  |
| Emc6 | 0.050006397 | 0.835981868 | 0.943188599 |  |  |  |  |  |  |  |  |
| Emc8 | 0.146291085 | 0.170794464 | 0.45944575 |  |  |  |  |  |  |  |  |
| Emc9 | 0.080973546 | 0.625050789 | 0.850614729 |  |  |  |  |  |  |  |  |
| Emd | 0.27862571 | 0.02269531 | 0.1122243 |  |  |  |  |  |  |  |  |
| Eml1 | 0.279981509 | 0.000838197 | 0.007614418 |  |  | ASD |  |  |  |  |  |
| Eml2 | 0.094238258 | 0.298299449 | 0.615266719 |  |  |  |  | FMRP |  |  |  |
| Eml4 | -0.110211162 | 0.214018199 | 0.52090606 |  |  |  |  |  |  |  |  |
| Eml5 | -0.309069419 | 5.8507E-05 | 0.000712445 |  |  |  |  |  |  |  |  |
| Eml6 | 0.158477722 | 0.252482937 | 0.570483568 |  |  |  |  |  |  |  |  |
| Emp2 | 0.331523056 | 0.271833126 | 0.589970344 |  |  |  |  |  |  |  |  |
| Enah | -0.011971179 | 0.838709744 | 0.94473205 | SYN |  |  |  |  |  |  |  |
| Enc1 | 0.375242577 | 9.40424E-05 | 0.001092882 |  |  |  |  | FMRP |  |  |  |
| Endod1 | 0.362747288 | 4.3527E-05 | 0.000541608 |  |  |  |  |  |  |  |  |
| Endov | -0.011760385 | 0.839806123 | 0.944846439 |  |  |  |  |  |  |  |  |
| Eno1 | 0.054019935 | 0.345165728 | 0.665727902 | SYN |  |  |  |  |  |  |  |
| Eno2 | 0.335799307 | 1.42083E-05 | 0.000197775 | SYN |  |  |  |  |  |  |  |
| Eno3 | -0.391714999 | 0.008194483 | 0.050705352 | SYN |  |  |  |  |  |  |  |
| Eno4 | 0.007687691 | 0.796701816 | 0.933720314 |  |  |  |  |  |  |  |  |
| Enoph1 | 0.075287073 | 0.379176041 | 0.693655256 |  |  |  |  |  |  |  |  |
| Enox1 | 0.302258885 | 0.047951212 | 0.200215655 |  |  |  |  |  |  |  |  |
| Enpp1 | 0.106826963 | 0.604317292 | 0.839393297 |  |  |  |  |  |  |  |  |
| Enpp2 | -0.245261347 | 0.185462865 | 0.48118097 |  |  |  |  |  |  |  |  |
| Enpp4 | 0.169250825 | 0.206511396 | 0.51216881 |  |  |  |  |  |  |  |  |
| Enpp5 | -0.258168266 | 0.004118932 | 0.028868718 |  |  |  |  |  |  |  |  |
| Ensa | 0.013610728 | 0.67710235 | 0.878273493 |  |  |  |  |  |  |  |  |
| Enthd2 | 0.220799824 | 0.153626453 | 0.430204164 |  |  |  |  |  |  |  |  |
| Entpd4 | -0.319173101 | 0.00048825 | 0.004737568 |  |  |  |  |  |  |  |  |
| Entpd5 | 0.182026465 | 0.055880807 | 0.221438997 |  |  |  |  |  |  |  |  |
| Entpd6 | -0.079527302 | 0.413266423 | 0.721903852 |  |  |  |  |  |  |  |  |
| Entpd7 | -0.047054717 | 0.609622852 | 0.842085516 |  |  |  |  |  |  |  |  |
| Eny2 | 0.064103749 | 0.464095512 | 0.758374472 |  |  |  |  |  |  |  |  |
| Eogt | -0.357410948 | 0.002022406 | 0.016376354 |  |  |  |  |  |  |  |  |
| Ep300 | -0.003067692 | 0.714671128 | 0.896386611 |  | ID | ASD |  | FMRP |  | SZ_108 | SZ_full |
| Ep400 | -0.164337584 | 0.091608556 | 0.310395007 |  |  | ASD |  | FMRP |  |  |  |
| Epas1 | -0.122836713 | 0.191453544 | 0.490219412 |  |  |  |  |  |  |  |  |
| Epb4.1 | 0.680941062 | 4.69266E-14 | 2.33929E-12 | SYN |  |  |  |  |  |  |  |
| Epb4.1l1 | -0.134461694 | 0.074784065 | 0.270021593 | SYN |  |  |  | FMRP |  |  |  |
| Epb4.1l2 | -0.486095445 | 1.00375E-07 | 2.10682E-06 | SYN |  |  |  |  |  |  |  |
| Epb4.1l3 | -0.110781181 | 0.129854564 | 0.389873367 | SYN |  |  |  |  |  |  |  |
| Epb4.9 | -0.002711144 | 0.84431895 | 0.945963967 |  |  |  |  | FMRP |  |  |  |
| Epc1 | 0.046045865 | 0.853778906 | 0.950119195 |  |  |  |  |  |  |  |  |
| Epc2 | -0.116985736 | 0.488750457 | 0.773006869 |  |  | ASD | ASD_sc |  |  | SZ_108 | SZ_full |
| Epdr1 | 0.070408779 | 0.36119864 | 0.678182758 |  |  |  |  |  |  |  |  |
| Epha10 | -0.442726985 | 0.002953731 | 0.022079624 |  |  |  |  |  |  |  |  |
| Epha4 | 0.079809342 | 0.211627606 | 0.518502158 | SYN |  |  |  | FMRP |  |  |  |
| Epha5 | 0.530068679 | 4.22707E-11 | 1.45953E-09 |  |  |  |  |  |  |  |  |
| Epha6 | 1.060626996 | 1.56791E-15 | 9.40274E-14 |  |  | ASD |  |  |  |  |  |
| Epha7 | 0.84077451 | 4.16797E-22 | 3.82112E-20 |  |  |  |  |  |  |  |  |
| Ephb1 | 0.088728267 | 0.464968962 | 0.759024242 |  |  |  |  |  |  |  |  |
| Ephb2 | -0.071412704 | 0.277088799 | 0.59394256 |  |  | ASD |  |  |  |  |  |
| Ephb3 | 0.401948514 | 0.000477812 | 0.004660208 |  |  |  |  |  |  |  |  |
| Ephb6 | -0.216550317 | 0.007705265 | 0.048163946 |  |  | ASD |  |  |  |  |  |
| Ephx4 | -0.028031727 | 0.604392641 | 0.839393297 |  |  |  |  |  |  |  |  |
| Epm2aip1 | -0.058641506 | 0.9324465 | 0.982845683 |  |  |  |  |  |  |  |  |
| Epn1 | 0.141358872 | 0.073830473 | 0.267602616 |  |  |  |  | FMRP |  |  |  |
| Epn2 | -0.0048579 | 0.776390591 | 0.924077297 | SYN |  |  |  |  |  |  |  |
| Eprs | -0.027454917 | 0.921458145 | 0.977593797 | SYN |  |  |  |  |  |  |  |
| Eps15 | 0.194160198 | 0.02536416 | 0.121943664 | SYN |  |  |  |  |  |  |  |
| Eps15l1 | -0.034137589 | 0.436779937 | 0.737692921 | SYN |  |  |  |  |  |  |  |
| Ept1 | -0.056266401 | 0.610408016 | 0.842510679 |  |  |  |  |  |  |  |  |
| Erbb2ip | 0.069475019 | 0.534456039 | 0.798060053 | SYN |  |  |  |  |  |  |  |
| Erbb3 | 0.366099763 | 0.037535447 | 0.165587793 |  |  |  |  |  | SZdb |  | SZ_full |
| Erbb4 | -0.11949513 | 0.205902789 | 0.511441309 |  |  | ASD |  |  | SZdb |  | SZ_full |
| Erc1 | 0.189391342 | 0.12777579 | 0.386821144 | SYN |  |  |  |  |  |  |  |
| Erc2 | -0.25656396 | 0.006476557 | 0.041793707 | SYN |  |  |  |  |  |  |  |
| Ercc2 | -0.121329226 | 0.16956082 | 0.457670762 |  | ID |  |  |  |  |  |  |
| Ercc4 | -0.056144929 | 0.402938238 | 0.71214393 |  |  |  |  |  |  | SZ_108 | SZ_full |
| Ercc5 | 0.088480275 | 0.39462684 | 0.705569082 |  | ID |  |  |  |  |  |  |
| Ercc6 | 0.051078421 | 0.660181679 | 0.870636421 |  | ID |  |  |  |  |  |  |
| Ercc6l2 | 0.030002967 | 0.863190527 | 0.953809724 |  |  |  |  |  |  |  |  |
| Ercc8 | -0.054650001 | 0.780161961 | 0.926216702 |  | ID |  |  |  |  |  |  |
| Erf | 0.003388124 | 0.876568475 | 0.959227516 |  |  |  |  |  |  |  |  |
| Ergic1 | -0.21035761 | 0.036544206 | 0.16202145 |  |  |  |  |  |  |  |  |
| Ergic2 | -0.17148937 | 0.177809246 | 0.468983646 |  |  |  |  |  |  |  |  |
| Ergic3 | -0.022515578 | 0.627285274 | 0.852338559 |  |  |  |  |  |  |  |  |
| Erh | 0.118347338 | 0.106927269 | 0.345613686 |  |  |  |  |  |  |  |  |
| Eri3 | 0.008069847 | 1 | 1 |  |  |  |  |  |  |  |  |
| Erlec1 | -0.275794353 | 0.005347063 | 0.035629216 |  |  |  |  |  |  |  |  |
| Erlin1 | -0.319717419 | 0.007156638 | 0.045256516 | SYN |  |  |  |  |  |  |  |
| Erlin2 | -0.145502971 | 0.170388023 | 0.459126647 | SYN |  |  |  |  |  |  |  |
| Ermn | 0.413655715 | 0.051018063 | 0.208249779 |  |  |  |  |  |  |  |  |
| Ermp1 | 0.281907071 | 0.001079496 | 0.009545518 |  |  |  |  |  |  |  |  |
| Ero1l | -0.001204123 | 0.871370421 | 0.956962054 |  |  |  |  |  |  |  |  |
| Ero1lb | -0.145182978 | 0.178895233 | 0.470913657 |  |  |  |  |  |  |  |  |
| Erp29 | 0.122717695 | 0.424628312 | 0.729941697 |  |  |  |  |  |  |  |  |
| Erp44 | 0.017063016 | 0.961432684 | 0.989984854 |  |  |  |  |  |  |  |  |
| Esam | -0.216083359 | 0.24591635 | 0.563224472 |  |  |  |  |  |  | SZ_108 | SZ_full |
| Esco1 | -0.128085516 | 0.25473377 | 0.57184254 |  |  |  |  |  |  |  |  |
| Esd | -0.223948589 | 0.137788928 | 0.405078683 |  |  |  |  |  | SZdb |  | SZ_full |
| Esrra | 0.327814341 | 0.001664457 | 0.01389755 |  |  |  |  |  |  |  |  |
| Esrrg | -1.008469044 | 8.87719E-10 | 2.57471E-08 |  |  |  |  |  |  |  |  |
| Esyt1 | 0.061772065 | 0.970728182 | 0.991487768 |  |  |  |  |  |  |  |  |
| Esyt2 | 0.064837168 | 0.597353763 | 0.834737271 |  |  |  |  |  |  |  |  |
| Etf1 | -0.050050908 | 0.810231102 | 0.93712146 |  |  |  |  |  |  | SZ_108 | SZ_full |
| Etfb | 0.285855522 | 0.069867499 | 0.258710852 | SYN |  | ASD | ASD_sc |  |  |  |  |
| Etfdh | 0.221790241 | 0.112865441 | 0.35736989 | SYN |  |  |  |  |  |  |  |
| Ethe1 | 0.101603582 | 0.737395821 | 0.908090941 |  |  |  |  |  |  |  |  |
| Etl4 | 0.031046708 | 0.823485008 | 0.941950287 | SYN |  |  |  |  |  |  |  |
| Etnk1 | -0.196820784 | 0.014588555 | 0.079155314 |  |  |  |  |  |  |  |  |
| Etnk2 | -3.488017831 | 1.38684E-51 | 6.91341E-49 |  |  |  |  |  |  |  |  |
| Etohi1 | 0.000812059 | 0.915662859 | 0.97483035 |  |  |  |  |  |  |  |  |
| Ets2 | 0.365452303 | 2.17938E-05 | 0.000289712 |  |  |  |  |  |  |  |  |
| Etv1 | -0.443584828 | 0.006516551 | 0.041983856 |  |  |  |  |  |  |  |  |
| Etv3 | 0.239877861 | 0.019806343 | 0.101077505 |  |  |  |  |  |  |  |  |
| Etv5 | 0.287490587 | 0.024495319 | 0.118913369 |  |  |  |  |  |  |  |  |
| Etv6 | -0.206219186 | 0.072897222 | 0.265250112 |  |  |  |  |  |  |  |  |
| Evi5 | -0.257644703 | 0.002079269 | 0.016650854 |  |  |  |  |  |  |  |  |
| Evi5l | -0.068274066 | 0.428454439 | 0.73153195 |  |  |  |  |  |  |  |  |
| Evl | -0.075007204 | 0.386058887 | 0.698615651 | SYN |  |  |  |  |  |  |  |
| Ewsr1 | 0.111540955 | 0.20191943 | 0.505495724 |  |  |  |  |  |  |  |  |
| Exd2 | -0.393803299 | 9.39432E-06 | 0.000135987 |  |  |  |  |  |  |  |  |
| Exoc1 | -0.105815867 | 0.307369523 | 0.624472496 | SYN |  |  |  |  |  |  |  |
| Exoc2 | -0.13946966 | 0.179559811 | 0.471297716 | SYN |  |  |  |  |  |  |  |
| Exoc3 | -0.005289231 | 0.985863757 | 0.996214749 | SYN |  |  |  |  |  |  |  |
| Exoc4 | 0.029609213 | 1 | 1 | SYN |  |  |  |  |  |  |  |
| Exoc5 | -0.081177143 | 0.541049622 | 0.802680899 | SYN |  |  |  |  |  |  |  |
| Exoc6 | 0.066399328 | 0.298674138 | 0.615445356 | SYN |  |  |  |  |  |  |  |
| Exoc6b | 0.084190084 | 0.302332355 | 0.620057306 | SYN |  | ASD |  |  |  |  |  |
| Exoc7 | 0.033880764 | 0.583654995 | 0.826572391 | SYN |  |  |  |  |  |  |  |
| Exoc8 | 0.052212041 | 0.654241982 | 0.869560748 | SYN |  |  |  |  |  |  |  |
| Exog | -0.01260421 | 0.800001325 | 0.935166555 |  |  |  |  |  |  |  |  |
| Exosc1 | 0.108271822 | 0.475024605 | 0.76408458 |  |  |  |  |  |  |  |  |
| Exosc10 | 0.102157566 | 0.226494896 | 0.53915805 |  |  |  |  |  |  |  |  |
| Exosc2 | -0.0801505 | 0.47461436 | 0.76408458 |  |  |  |  |  |  |  |  |
| Exosc4 | 0.034669044 | 0.625163433 | 0.850614729 |  |  |  |  |  |  |  |  |
| Exosc5 | 0.377933334 | 0.050801632 | 0.207578801 |  |  |  |  |  |  |  |  |
| Exosc6 | 0.272176808 | 0.36775681 | 0.684534029 |  |  |  |  |  |  |  |  |
| Exosc7 | 0.045344346 | 0.867658043 | 0.955251846 |  |  |  |  |  |  |  |  |
| Exosc8 | -0.201965458 | 0.123609539 | 0.379341933 |  |  |  |  |  |  |  |  |
| Exosc9 | -0.040902503 | 0.644727447 | 0.862375669 |  |  |  |  |  |  |  |  |
| Exph5 | -2.226099207 | 2.87314E-46 | 1.14581E-43 |  |  |  |  |  |  |  |  |
| Ext1 | -0.528419092 | 7.16631E-05 | 0.000850572 |  |  | ASD | ASD_sc |  |  |  |  |
| Ext2 | -0.091638786 | 0.323565259 | 0.641181741 | SYN |  |  |  |  |  |  |  |
| Extl1 | 0.4928556 | 1.76471E-06 | 2.94463E-05 |  |  |  |  |  |  |  |  |
| Extl2 | -0.360603862 | 0.000289054 | 0.002959558 |  |  |  |  |  |  |  |  |
| Extl3 | -0.626531273 | 1.50514E-12 | 6.18814E-11 |  |  |  |  | FMRP |  |  |  |
| Eya1 | 0.184580073 | 0.26766534 | 0.585384905 |  |  |  |  |  |  |  |  |
| Eya3 | -0.11428227 | 0.236724185 | 0.551595704 |  |  |  |  |  |  |  |  |
| Ezh1 | -0.068416495 | 0.472511264 | 0.76369839 |  |  |  |  |  |  |  |  |
| Ezh2 | -0.097656679 | 0.593914266 | 0.833255969 |  |  |  |  |  |  |  |  |
| Ezr | -0.08775791 | 0.698099353 | 0.886630643 | SYN |  |  |  |  |  |  |  |
| F11r | -0.176118634 | 0.243904854 | 0.560127769 |  |  |  |  |  |  |  |  |
| F3 | -0.020787052 | 0.911573243 | 0.97303544 |  |  |  |  |  |  |  |  |
| F730043M19Rik | -0.719558547 | 2.20154E-09 | 6.03419E-08 |  |  |  |  |  |  |  |  |
| Faah | -0.237021541 | 0.002031821 | 0.016419257 | SYN |  |  |  |  |  |  |  |
| Fabp3 | -0.00410593 | 0.745572455 | 0.911928524 | SYN |  | ASD |  |  |  |  |  |
| Fabp5 | 0.308997964 | 0.001266494 | 0.010944261 | SYN |  | ASD | ASD_sc |  |  |  |  |
| Fads1 | 0.012915416 | 0.9654451 | 0.990416798 |  |  |  |  |  |  |  |  |
| Fads2 | 0.146714738 | 0.063174353 | 0.241784376 |  |  |  |  |  |  |  |  |
| Faf1 | -0.074308016 | 0.414632348 | 0.723036433 |  |  |  |  |  |  |  |  |
| Faf2 | -0.031474877 | 0.76507352 | 0.919979858 |  |  |  |  |  |  |  |  |
| Faim | 0.044591748 | 0.814057013 | 0.938538051 |  |  |  |  |  |  |  |  |
| Faim2 | -0.114958682 | 0.152544214 | 0.428338929 |  |  |  |  |  |  |  |  |
| Fam102a | 0.08591479 | 0.348929317 | 0.668361715 |  |  |  |  |  |  |  |  |
| Fam102b | -0.6000349 | 2.17073E-06 | 3.51905E-05 |  |  |  |  |  |  |  |  |
| Fam103a1 | -0.066237632 | 0.609892628 | 0.842192452 |  |  |  |  |  |  |  |  |
| Fam105b | 0.110243474 | 0.446305101 | 0.744401816 |  |  |  |  |  |  |  |  |
| Fam107a | -0.261433205 | 0.003200874 | 0.023530114 | SYN |  |  |  |  |  |  |  |
| Fam108a | 0.00296707 | 0.888717633 | 0.962995126 |  |  |  |  |  |  |  |  |
| Fam108c | 0.139656737 | 0.077420256 | 0.277031835 |  |  |  |  |  |  |  |  |
| Fam110b | 0.030497845 | 0.46029413 | 0.755723751 |  |  |  |  |  |  |  |  |
| Fam115a | 0.034689323 | 0.552590153 | 0.808060922 |  |  |  |  |  |  |  |  |
| Fam117b | -0.147270305 | 0.266378249 | 0.583369829 |  |  |  |  |  |  |  |  |
| Fam118a | 0.366115694 | 0.000837078 | 0.007612923 |  |  |  |  |  |  |  |  |
| Fam120a | -0.014148915 | 0.957361022 | 0.989355248 |  |  |  |  |  |  |  |  |
| Fam120aos | -0.070815531 | 1 | 1 |  |  |  |  |  |  |  |  |
| Fam120c | 0.021843605 | 0.426629484 | 0.730684295 |  |  |  |  |  |  |  |  |
| Fam126a | 0.136462053 | 0.194873992 | 0.495712359 |  |  |  |  |  |  |  |  |
| Fam126b | -0.180043879 | 0.286157167 | 0.604177883 |  |  |  |  |  |  |  |  |
| Fam129b | -0.40619672 | 0.004052878 | 0.02845577 |  |  |  |  |  |  |  |  |
| Fam131a | 0.009080237 | 0.834716585 | 0.943188599 |  |  |  |  |  |  |  |  |
| Fam131b | -0.155909923 | 0.12630581 | 0.384509595 |  |  |  |  |  |  |  |  |
| Fam133b | -0.027381519 | 0.759864256 | 0.918279887 |  |  |  |  |  |  |  |  |
| Fam134a | -0.217754666 | 0.010764919 | 0.062580899 |  |  |  |  |  |  |  |  |
| Fam134b | -0.312156654 | 0.004829212 | 0.03284224 |  |  |  |  |  |  |  |  |
| Fam134c | 0.006374584 | 0.897075835 | 0.966457471 |  |  |  |  |  |  |  |  |
| Fam135a | -0.040736718 | 0.809727453 | 0.93712146 |  |  |  |  |  |  |  |  |
| Fam13a | 0.411360432 | 0.002228664 | 0.017683902 |  |  |  |  |  |  |  |  |
| Fam13b | -0.122400456 | 0.281506489 | 0.599384879 |  |  |  |  |  |  |  |  |
| Fam149b | -0.150200149 | 0.087736059 | 0.302021066 |  |  |  |  |  |  |  |  |
| Fam155a | -0.49760576 | 0.000276887 | 0.002856992 |  |  |  |  |  |  |  |  |
| Fam160a2 | -0.27620234 | 0.010952357 | 0.063485466 |  |  |  |  |  |  |  |  |
| Fam160b1 | -0.085332757 | 0.283825022 | 0.602914815 |  |  |  |  |  |  |  |  |
| Fam160b2 | -0.115907746 | 0.172656367 | 0.461777959 |  |  |  |  |  |  |  |  |
| Fam161a | 0.265396384 | 0.228690145 | 0.541738223 |  |  |  |  |  |  |  |  |
| Fam163b | -0.442450575 | 5.09832E-06 | 7.76034E-05 |  |  |  |  |  |  |  |  |
| Fam168a | -0.029723978 | 0.808169646 | 0.93712146 |  |  |  |  |  |  |  |  |
| Fam168b | -0.127610854 | 0.150464383 | 0.42632466 |  |  |  |  |  |  |  |  |
| Fam169a | -0.333247108 | 0.008083124 | 0.050132966 |  |  |  |  |  |  |  |  |
| Fam171a1 | 0.01328972 | 0.737105523 | 0.907977398 | SYN |  |  |  |  |  |  |  |
| Fam171a2 | 0.104340391 | 0.295701725 | 0.612283738 |  |  |  |  |  |  |  |  |
| Fam171b | -0.123199492 | 0.360678928 | 0.677685543 |  |  |  |  |  |  |  |  |
| Fam173a | 0.146549914 | 0.357287696 | 0.675290951 |  |  |  |  |  |  |  |  |
| Fam174a | -0.027541964 | 0.578849017 | 0.824002257 |  |  |  |  |  |  |  |  |
| Fam174b | 0.142354109 | 0.192043963 | 0.491573379 |  |  |  |  |  |  |  |  |
| Fam175a | -0.203064409 | 0.17054861 | 0.45924486 |  |  |  |  |  |  |  |  |
| Fam175b | -0.06465637 | 0.63677462 | 0.858214662 |  |  |  |  |  |  |  |  |
| Fam178a | -0.061277871 | 0.427095177 | 0.731167599 |  |  |  |  |  |  |  |  |
| Fam179b | -0.072021708 | 0.575833384 | 0.822118754 |  |  |  |  |  |  |  |  |
| Fam181b | 0.258156883 | 0.1295353 | 0.389729745 |  |  |  |  |  |  |  |  |
| Fam185a | -0.216106662 | 0.114273626 | 0.35979585 |  |  |  |  |  |  |  |  |
| Fam188a | 0.01138541 | 0.849719565 | 0.948148188 |  |  |  |  |  |  |  |  |
| Fam189a1 | -0.308109175 | 0.001142866 | 0.010006041 |  |  |  |  |  |  |  |  |
| Fam189b | 0.353768 | 0.004145487 | 0.029029328 |  |  |  |  |  |  |  |  |
| Fam192a | 0.040940073 | 0.581872964 | 0.825657136 |  |  |  |  |  |  |  |  |
| Fam193a | 0.007839657 | 0.939170582 | 0.98530815 |  |  |  |  |  |  |  |  |
| Fam193b | 0.056624361 | 0.892707669 | 0.965063211 |  |  |  |  |  |  |  |  |
| Fam195b | 0.038630646 | 0.669040565 | 0.875803996 |  |  |  |  |  |  |  |  |
| Fam19a1 | -2.403784567 | 3.70606E-98 | 1.47798E-94 |  |  |  |  |  |  |  |  |
| Fam19a2 | -1.373713532 | 1.69662E-42 | 6.151E-40 |  |  |  |  |  |  |  |  |
| Fam19a5 | 0.284533623 | 0.001261928 | 0.01091664 |  |  |  |  |  |  |  |  |
| Fam203a | 0.02671772 | 1 | 1 |  |  |  |  |  |  |  |  |
| Fam207a | -0.005502092 | 0.875331408 | 0.958753545 |  |  |  |  |  |  |  |  |
| Fam20b | 0.033325878 | 0.511355842 | 0.786598895 |  |  |  |  |  |  |  |  |
| Fam20c | -0.172199931 | 0.201228116 | 0.504695914 |  |  |  |  |  |  |  |  |
| Fam21 | 0.143038999 | 0.124427333 | 0.380472824 |  |  |  |  |  |  |  |  |
| Fam210a | -0.087366888 | 0.581019036 | 0.825093019 |  |  |  |  |  |  |  |  |
| Fam211b | 0.105408201 | 0.57364736 | 0.820827732 |  |  |  |  |  |  |  |  |
| Fam212b | 0.309983405 | 0.000273856 | 0.00283586 |  |  |  |  |  |  |  |  |
| Fam213b | -0.668749119 | 2.06421E-08 | 4.92939E-07 |  |  |  |  |  |  |  |  |
| Fam214a | 0.207547488 | 0.025975061 | 0.123761699 |  |  |  |  |  |  |  |  |
| Fam216a | 0.124212621 | 0.28251571 | 0.601121212 |  |  |  |  |  |  |  |  |
| Fam219a | -0.100396473 | 0.152749741 | 0.428688224 |  |  |  |  |  |  |  |  |
| Fam219b | 0.004856907 | 0.937164885 | 0.98530815 |  |  |  |  |  |  |  |  |
| Fam222b | 0.018608785 | 0.95683449 | 0.989355248 |  |  |  |  |  |  |  |  |
| Fam32a | -0.101077679 | 0.463831787 | 0.758236051 |  |  |  |  |  |  |  |  |
| Fam3c | -0.506528041 | 9.46163E-08 | 1.99645E-06 |  |  |  |  |  |  |  |  |
| Fam43b | 0.473506182 | 0.000455429 | 0.004472637 |  |  |  |  |  |  |  |  |
| Fam46a | 0.792369361 | 0.000397276 | 0.003949223 |  |  |  |  |  |  |  |  |
| Fam49a | 0.113264686 | 0.111859587 | 0.355313448 | SYN |  |  |  |  |  |  |  |
| Fam49b | -0.070120598 | 0.604813944 | 0.839508853 | SYN |  |  |  |  |  |  |  |
| Fam50a | 0.230330122 | 0.279648645 | 0.597182756 |  |  |  |  |  |  |  |  |
| Fam53a | 0.030411865 | 1 | 1 |  |  |  |  |  |  |  |  |
| Fam53b | 0.290967377 | 0.001175227 | 0.010251298 |  |  |  |  |  |  |  |  |
| Fam53c | -0.004977106 | 0.983058239 | 0.994693692 |  |  |  |  |  |  | SZ_108 | SZ_full |
| Fam57a | 0.109212225 | 0.622166105 | 0.849836043 |  |  |  |  |  |  |  |  |
| Fam57b | 0.093837998 | 0.473705413 | 0.76408458 |  |  |  |  |  |  | SZ_108 | SZ_full |
| Fam5b | -0.065976083 | 0.489586568 | 0.77362416 |  |  |  |  |  |  |  |  |
| Fam5c | -1.373512845 | 5.76014E-33 | 1.06844E-30 |  |  |  |  |  |  |  |  |
| Fam63b | 0.190694787 | 0.022991934 | 0.113495842 |  |  |  |  |  |  |  |  |
| Fam65a | 0.271762839 | 0.002367867 | 0.01853396 |  |  |  |  |  |  |  |  |
| Fam65b | 0.066906379 | 0.338741875 | 0.659792809 |  |  |  |  |  |  |  |  |
| Fam69a | -0.124438673 | 0.179267662 | 0.471271876 |  |  |  |  |  |  |  |  |
| Fam71e1 | -0.168035307 | 0.258055633 | 0.575573749 |  |  |  |  |  |  |  |  |
| Fam73a | -0.00812642 | 0.810155889 | 0.93712146 |  |  |  |  |  |  |  |  |
| Fam73b | -0.075868863 | 0.305713323 | 0.62282745 |  |  |  |  |  |  |  |  |
| Fam76a | 0.187487011 | 0.034257293 | 0.15393587 |  |  |  |  |  |  |  |  |
| Fam81a | -0.041622628 | 0.659150397 | 0.870176186 | SYN |  |  |  |  |  |  |  |
| Fam92a | 0.026385856 | 0.948802017 | 0.986912479 |  |  |  |  |  |  |  |  |
| Fam98c | 0.408034335 | 0.000944675 | 0.00848505 |  |  |  |  |  |  |  |  |
| Fancd2 | 1.778393028 | 3.04267E-29 | 4.33363E-27 |  |  |  |  |  |  |  |  |
| Fancg | -0.380930805 | 0.003426717 | 0.024785886 |  |  |  |  |  |  |  |  |
| Fancm | -0.327529926 | 0.004641344 | 0.031885757 |  |  |  |  |  |  |  |  |
| Fap | 0.455826456 | 0.026361476 | 0.125005428 |  |  |  |  |  |  |  |  |
| Far1 | 0.085607557 | 0.270152101 | 0.588724906 |  |  |  |  |  |  |  |  |
| Farp1 | -0.440877991 | 1.83788E-07 | 3.66474E-06 | SYN |  |  |  |  |  |  |  |
| Farsa | 0.076013765 | 0.629839429 | 0.853812312 | SYN |  |  |  |  |  |  |  |
| Farsb | -0.059231121 | 0.756254877 | 0.916421893 | SYN |  |  |  |  |  |  |  |
| Fasn | -0.10368737 | 0.43240026 | 0.733800692 | SYN |  |  |  | FMRP |  |  |  |
| Fastk | 0.09870779 | 0.295187257 | 0.611588794 |  |  |  |  |  |  |  |  |
| Fastkd2 | 0.00838939 | 0.802285421 | 0.935393732 |  |  |  |  |  |  |  |  |
| Fat1 | -0.33175025 | 0.000856289 | 0.007769926 |  |  | ASD |  | FMRP |  |  |  |
| Fat3 | -0.509973161 | 6.10315E-08 | 1.34844E-06 |  |  |  |  | FMRP |  |  |  |
| Fat4 | -0.395706226 | 0.071384523 | 0.26165577 |  |  |  |  | FMRP |  |  |  |
| Faxc | -0.205436999 | 0.035573927 | 0.158689956 |  |  |  |  |  |  |  |  |
| Fbf1 | -0.038375756 | 0.4971163 | 0.777150882 |  |  |  |  |  |  |  |  |
| Fbl | -0.003502162 | 0.883381929 | 0.961506354 |  |  |  |  |  |  |  |  |
| Fbll1 | 0.17418503 | 0.590594922 | 0.830351683 |  |  |  |  |  |  |  |  |
| Fbn1 | 0.160562344 | 0.173505671 | 0.462387242 |  | ID |  |  |  |  |  |  |
| Fbn2 | 1.470177662 | 1.51854E-22 | 1.40836E-20 |  |  |  |  |  |  |  |  |
| Fbxl12 | 0.0560941 | 0.95956457 | 0.989888316 |  |  |  |  |  |  |  |  |
| Fbxl14 | 0.17229744 | 0.112619996 | 0.356948835 |  |  |  |  |  |  |  |  |
| Fbxl15 | 0.360363735 | 0.303222017 | 0.621045999 |  |  |  |  |  |  |  |  |
| Fbxl16 | -0.239767249 | 0.01177663 | 0.066997433 |  |  |  |  | FMRP |  |  |  |
| Fbxl17 | -0.182306149 | 0.023522607 | 0.115456192 |  |  |  |  |  |  |  |  |
| Fbxl18 | -0.037866536 | 0.589411682 | 0.82956548 |  |  |  |  |  |  |  |  |
| Fbxl19 | 0.03838292 | 0.845966521 | 0.94647622 |  |  |  |  | FMRP |  |  |  |
| Fbxl2 | -0.24929941 | 0.001762361 | 0.014566415 |  |  |  |  |  |  |  |  |
| Fbxl20 | -0.086487421 | 0.263098373 | 0.57968857 |  |  |  |  |  |  |  |  |
| Fbxl3 | 0.031844473 | 0.429425405 | 0.73153195 |  |  |  |  |  |  |  |  |
| Fbxl4 | -0.111061303 | 0.443368371 | 0.742141894 |  |  |  |  |  |  |  |  |
| Fbxl5 | -0.121630081 | 0.384939798 | 0.698108192 |  |  |  |  |  |  |  |  |
| Fbxl6 | 0.163836585 | 0.226045387 | 0.538833834 |  |  |  |  |  |  |  |  |
| Fbxo10 | -0.192100541 | 0.101633559 | 0.333592291 |  |  |  |  |  |  |  |  |
| Fbxo11 | -0.061040218 | 0.755918706 | 0.916153715 |  |  |  |  |  |  |  |  |
| Fbxo18 | -0.479663992 | 3.13322E-06 | 4.92911E-05 |  |  |  |  |  |  |  |  |
| Fbxo2 | 0.644249038 | 3.40751E-06 | 5.33921E-05 | SYN |  |  |  |  |  |  |  |
| Fbxo21 | 0.23047814 | 0.00423557 | 0.029556348 |  |  |  |  |  |  |  |  |
| Fbxo22 | -0.091125973 | 0.31405414 | 0.63143328 |  |  |  |  |  |  |  |  |
| Fbxo25 | -0.00454193 | 0.608261934 | 0.841251463 |  |  |  |  |  |  |  |  |
| Fbxo27 | -0.276255409 | 0.032868606 | 0.149293851 |  |  |  |  |  |  |  |  |
| Fbxo28 | 0.010727239 | 0.88211235 | 0.960863288 |  |  |  |  |  |  |  |  |
| Fbxo3 | -0.054840256 | 0.588867713 | 0.829437611 |  |  |  |  |  |  |  |  |
| Fbxo31 | 0.273556239 | 0.00190806 | 0.015592914 | SYN |  |  |  |  |  |  |  |
| Fbxo32 | -0.024494733 | 0.938975034 | 0.98530815 |  |  |  |  |  |  |  |  |
| Fbxo33 | -0.296471289 | 0.002433105 | 0.018951606 |  |  | ASD | ASD_sc |  |  |  |  |
| Fbxo34 | -0.659736858 | 2.59691E-11 | 9.45796E-10 |  |  |  |  |  |  |  |  |
| Fbxo36 | 0.085268832 | 0.767677868 | 0.920286747 |  |  |  |  |  |  |  |  |
| Fbxo38 | -0.09439293 | 0.438577401 | 0.738351249 |  |  |  |  |  |  |  |  |
| Fbxo41 | -0.074974784 | 0.359886293 | 0.677462343 | SYN |  |  |  | FMRP |  |  |  |
| Fbxo42 | -0.078588593 | 0.468545402 | 0.76189972 |  |  |  |  |  |  |  |  |
| Fbxo44 | 0.048246593 | 0.580365645 | 0.824617053 |  |  |  |  |  |  |  |  |
| Fbxo45 | -0.246072417 | 0.024418692 | 0.118673258 |  |  |  |  |  |  |  |  |
| Fbxo6 | -0.160952699 | 0.318620288 | 0.636124009 |  |  |  |  |  |  |  |  |
| Fbxo7 | 0.050182361 | 0.674536256 | 0.878031572 |  |  |  |  |  |  |  |  |
| Fbxo9 | -0.004761945 | 0.982828032 | 0.994693692 |  |  |  |  |  |  |  |  |
| Fbxw11 | -0.232745281 | 0.031688411 | 0.145089991 |  |  |  |  |  |  |  |  |
| Fbxw2 | -0.093318828 | 0.541159035 | 0.802680899 |  |  |  |  |  |  |  |  |
| Fbxw4 | -0.009294225 | 0.714967953 | 0.896386611 |  |  |  |  |  |  |  |  |
| Fbxw5 | -0.01628356 | 0.884062686 | 0.961975987 |  |  |  |  |  |  |  |  |
| Fbxw7 | -0.015099998 | 0.828564313 | 0.94210462 |  |  |  |  |  |  |  |  |
| Fbxw8 | 0.121417777 | 0.216868148 | 0.525437529 |  |  |  |  |  |  |  |  |
| Fbxw9 | 0.024082316 | 0.901664477 | 0.969134709 |  |  |  |  |  |  |  |  |
| Fcgr2b | 0.161531887 | 0.683000185 | 0.880635221 |  |  |  |  |  |  |  |  |
| Fcho1 | -0.643004168 | 1.87906E-11 | 7.03632E-10 |  |  |  |  | FMRP |  |  |  |
| Fcho2 | -0.153620521 | 0.127894344 | 0.38698228 |  |  |  |  |  |  |  |  |
| Fchsd2 | -0.200372814 | 0.105996699 | 0.343530951 |  |  |  |  |  |  |  |  |
| Fdft1 | 0.032475169 | 0.595926728 | 0.834171917 |  |  |  |  |  |  |  |  |
| Fdps | 0.233677465 | 0.005931076 | 0.038871208 | SYN |  |  |  |  |  |  |  |
| Fech | 0.149178687 | 0.090832956 | 0.308548609 |  |  |  |  |  |  |  |  |
| Fem1a | 0.061699046 | 0.514119108 | 0.787995408 |  |  |  |  |  |  |  |  |
| Fem1b | -0.028450254 | 0.863494307 | 0.953809724 |  |  |  |  |  |  |  |  |
| Fem1c | -0.322562626 | 0.013001899 | 0.072519685 |  |  |  |  |  |  |  |  |
| Fen1 | -0.067092243 | 0.716509913 | 0.897015079 |  |  |  |  |  |  |  |  |
| Fermt2 | -0.094302125 | 0.231500593 | 0.545479684 |  |  |  |  |  |  |  |  |
| Fert2 | -0.082484214 | 0.561300317 | 0.814107604 |  |  |  |  |  |  |  |  |
| Fez1 | 0.38443518 | 0.00134293 | 0.0115547 |  |  |  |  |  |  |  |  |
| Fez2 | -0.479810415 | 1.63999E-05 | 0.000225139 |  |  |  |  |  |  |  |  |
| Fgd1 | 0.182294677 | 0.179808771 | 0.471606301 |  | ID | ASD |  |  |  |  |  |
| Fgd5 | 0.139576864 | 0.397612615 | 0.707892459 |  |  |  |  |  |  |  |  |
| Fgd6 | -0.147357845 | 0.235221797 | 0.549539852 |  |  |  |  |  |  |  |  |
| Fgf1 | 0.142218371 | 0.231430763 | 0.545476289 |  |  |  |  |  | SZdb |  | SZ_full |
| Fgf12 | -0.632742265 | 2.60531E-14 | 1.34935E-12 |  |  |  |  |  |  |  |  |
| Fgf13 | 0.004167503 | 0.927271768 | 0.980631082 |  |  |  |  |  |  |  |  |
| Fgf14 | -0.190008641 | 0.245870528 | 0.563224472 |  |  |  |  |  | SZdb |  | SZ_full |
| Fgf5 | -0.253475336 | 0.210778003 | 0.518399997 |  |  |  |  |  |  |  |  |
| Fgf9 | -0.460389703 | 9.19304E-06 | 0.000133316 |  |  |  |  |  |  |  |  |
| Fgfbp3 | 0.67299501 | 6.3242E-05 | 0.000761961 |  |  | ASD |  |  |  |  |  |
| Fgfr1 | 0.331629312 | 0.000167684 | 0.001817188 |  | ID |  |  |  | SZdb |  | SZ_full |
| Fgfr1op | 0.016943725 | 0.997128656 | 1 |  |  |  |  |  |  |  |  |
| Fgfr1op2 | -0.272689096 | 0.003791889 | 0.026931532 |  |  |  |  |  |  |  |  |
| Fgfr2 | 0.117210121 | 0.591013432 | 0.830403111 |  | ID |  |  |  |  |  |  |
| Fgfr3 | -0.064925824 | 0.490703421 | 0.773853814 |  | ID |  |  |  |  |  |  |
| Fggy | -0.195442254 | 0.10085724 | 0.332000554 |  |  |  |  |  |  |  |  |
| Fhl1 | 0.033247839 | 0.622768745 | 0.849966377 |  |  |  |  |  |  |  |  |
| Fhl2 | 0.062335572 | 0.617871912 | 0.847780211 |  |  |  |  |  |  |  |  |
| Fhod3 | -1.503044817 | 3.7062E-55 | 2.46339E-52 |  |  |  |  |  |  |  |  |
| Fibcd1 | 2.279443957 | 9.45572E-16 | 5.80145E-14 |  |  |  |  |  |  |  |  |
| Fig4 | 0.140625447 | 0.13817724 | 0.405333454 |  |  |  |  |  |  |  |  |
| Fip1l1 | 0.154718706 | 0.117321164 | 0.365243404 |  |  |  |  |  |  |  |  |
| Fis1 | 0.071884647 | 0.699763507 | 0.887191673 | SYN |  |  |  |  |  |  |  |
| Fitm2 | -0.088838534 | 0.525460944 | 0.793545744 |  |  |  |  |  |  |  |  |
| Fjx1 | 0.082196461 | 0.462922205 | 0.757441468 |  |  |  |  |  |  |  |  |
| Fkbp15 | 0.055363409 | 0.532621808 | 0.797338264 | SYN |  |  |  |  |  |  |  |
| Fkbp1a | -0.141742711 | 0.116068811 | 0.36327928 |  |  |  |  |  |  |  |  |
| Fkbp1b | -0.017604092 | 0.487132718 | 0.771519173 | SYN |  |  |  |  |  |  |  |
| Fkbp3 | -0.055275435 | 0.339427612 | 0.660457575 |  |  |  |  |  |  |  |  |
| Fkbp4 | 0.163716533 | 0.04337664 | 0.18587121 | SYN |  |  |  |  |  |  |  |
| Fkbp5 | 0.573259219 | 2.05361E-06 | 3.33596E-05 |  |  |  |  |  |  |  |  |
| Fkbp8 | 0.031071879 | 0.899689837 | 0.968019175 | SYN |  |  |  | FMRP |  |  |  |
| Fkbp9 | 0.456057579 | 8.53574E-06 | 0.000124463 |  |  |  |  |  |  |  |  |
| Fkrp | 0.108205282 | 0.338013944 | 0.659329719 |  | ID |  |  |  |  |  |  |
| Fktn | -0.089604596 | 0.25093698 | 0.568599324 |  | ID |  |  |  |  |  |  |
| Flad1 | 0.054694225 | 0.617531143 | 0.847604267 |  |  |  |  |  |  |  |  |
| Flcn | 0.018788082 | 0.907455256 | 0.97125661 |  |  |  |  |  |  |  |  |
| Flii | -0.042648082 | 0.771516742 | 0.921476121 | SYN |  |  |  |  |  |  |  |
| Flna | 0.219223824 | 0.05974956 | 0.231733717 | SYN | ID |  |  |  |  |  |  |
| Flnb | -0.140421515 | 0.040523008 | 0.176137064 |  |  |  |  |  | SZdb |  | SZ_full |
| Flot1 | -0.199489643 | 0.035694812 | 0.159051295 | SYN |  |  |  |  |  |  |  |
| Flot2 | -0.24269327 | 0.003305295 | 0.024075827 | SYN |  |  |  |  |  |  |  |
| Flrt1 | -0.690203395 | 1.80302E-10 | 5.70671E-09 |  |  |  |  |  |  |  |  |
| Flrt2 | -0.173002949 | 0.097361852 | 0.323835753 |  |  |  |  |  |  |  |  |
| Flrt3 | 0.094930485 | 0.309472889 | 0.626486234 |  |  |  |  |  |  |  |  |
| Flt1 | -0.092089611 | 0.54967611 | 0.80739104 |  |  | ASD |  |  |  |  |  |
| Flt3 | -0.318406914 | 0.002418582 | 0.018857276 |  |  |  |  |  |  |  |  |
| Flywch1 | -0.202600363 | 0.013299288 | 0.07371447 |  |  |  |  |  |  |  |  |
| Flywch2 | -0.269234237 | 0.116545709 | 0.364108332 |  |  |  |  |  |  |  |  |
| Fmn1 | -0.158496063 | 0.134536339 | 0.398998958 |  |  |  |  |  |  |  |  |
| Fmn2 | 0.007300917 | 0.73529151 | 0.906582946 | SYN |  |  |  |  |  |  |  |
| Fmnl1 | -0.131405863 | 0.306594546 | 0.624043555 | SYN |  |  |  |  |  |  |  |
| Fmnl2 | -0.063105969 | 0.514347231 | 0.787995408 | SYN |  |  |  |  |  |  |  |
| Fmo5 | -0.023338331 | 0.906477592 | 0.970739161 |  |  |  |  |  |  |  |  |
| Fmr1 | -0.879215968 | 0.145978517 | 0.418821818 |  | ID | ASD | ASD_sc |  |  |  |  |
| Fn1 | 0.270484611 | 0.151126885 | 0.426634282 |  |  |  |  |  |  |  |  |
| Fn3k | -0.122338609 | 0.186067302 | 0.481595848 | SYN |  |  |  |  |  |  |  |
| Fn3krp | -0.388458877 | 0.000276325 | 0.002854886 |  |  |  |  |  |  |  |  |
| Fnbp1 | 0.163125937 | 0.10611855 | 0.34378617 |  |  |  |  |  |  |  |  |
| Fnbp1l | -0.609770992 | 2.41665E-10 | 7.50009E-09 |  |  |  |  |  |  |  |  |
| Fndc3a | -0.026217334 | 0.836986474 | 0.943363721 |  |  |  |  |  |  |  |  |
| Fndc3b | -0.199648782 | 0.11472968 | 0.360553164 |  |  |  |  |  |  |  |  |
| Fndc4 | -0.042333582 | 0.60255672 | 0.837572743 |  |  |  |  |  |  |  |  |
| Fndc5 | -0.299365981 | 0.000216259 | 0.002290685 |  |  |  |  |  |  |  |  |
| Fnip1 | 0.00481869 | 0.957274907 | 0.989355248 |  |  |  |  |  |  |  |  |
| Fnip2 | -0.190693376 | 0.086257766 | 0.298737274 |  |  |  |  |  |  |  |  |
| Fnta | -0.042535789 | 0.742770692 | 0.910996956 |  |  |  |  |  |  |  |  |
| Fntb | -0.036040869 | 0.766493699 | 0.920286747 |  |  |  |  |  |  |  |  |
| Focad | 0.045506861 | 0.943496727 | 0.985962641 |  |  |  |  |  |  |  |  |
| Fopnl | -0.141415642 | 0.186289835 | 0.481792388 |  |  |  |  |  |  |  |  |
| Fosl2 | 1.088356828 | 7.28028E-09 | 1.84928E-07 |  |  |  |  |  |  |  |  |
| Foxg1 | 0.610431121 | 1.04177E-08 | 2.60474E-07 |  |  | ASD |  |  |  |  |  |
| Foxj2 | -0.037836712 | 0.703373768 | 0.888798685 |  |  |  |  |  |  |  |  |
| Foxj3 | -0.090188749 | 0.518205334 | 0.790287905 |  |  |  |  |  |  |  |  |
| Foxk1 | -0.254893302 | 0.023687034 | 0.115966855 |  |  |  |  |  |  |  |  |
| Foxk2 | -0.263377569 | 0.007768819 | 0.048447306 |  |  |  |  | FMRP |  |  |  |
| Foxn2 | -0.246453051 | 0.077173638 | 0.276397369 |  |  |  |  |  |  |  |  |
| Foxo1 | 0.492804994 | 0.001453016 | 0.012368465 |  |  |  |  |  |  |  |  |
| Foxo3 | -0.699010677 | 6.88318E-11 | 2.33618E-09 |  |  |  |  |  |  |  |  |
| Foxp2 | 0.151971396 | 0.163321564 | 0.446572777 |  | ID | ASD | ASD_sc |  | SZdb |  | SZ_full |
| Foxp4 | 0.314142676 | 0.002179625 | 0.017384688 |  |  |  |  |  |  |  |  |
| Fras1 | -0.365284248 | 0.000992671 | 0.008886129 |  | ID |  |  |  |  |  |  |
| Frg1 | 0.057183928 | 0.616727019 | 0.847083641 |  |  |  |  |  |  |  |  |
| Frmd4a | -0.077345231 | 0.519988229 | 0.790843055 |  |  |  |  |  |  |  |  |
| Frmd4b | -0.025892243 | 0.778711766 | 0.925395592 |  |  |  |  |  |  |  |  |
| Frmd5 | -0.635297478 | 3.70376E-06 | 5.76977E-05 |  |  |  |  |  |  |  |  |
| Frmd6 | 0.096541883 | 0.249223923 | 0.566489032 |  |  |  |  |  |  |  |  |
| Frmd8 | 0.213128179 | 0.571595934 | 0.820827732 |  |  |  |  |  |  |  |  |
| Frmpd1 | -0.264666754 | 0.04096473 | 0.177476744 |  |  |  |  |  |  |  |  |
| Frmpd4 | -0.018606944 | 0.743870627 | 0.911502699 |  |  | ASD |  | FMRP |  |  |  |
| Frrs1l | 0.004969267 | 0.856148789 | 0.951377789 |  |  |  |  |  |  |  |  |
| Frs2 | -0.203130371 | 0.027471062 | 0.129267959 |  |  |  |  |  |  |  |  |
| Frs3 | 0.00422237 | 0.965771793 | 0.990416798 |  |  |  |  |  |  |  |  |
| Fry | -0.03905586 | 0.817808737 | 0.94093963 |  |  |  |  | FMRP |  |  |  |
| Fryl | 0.292207409 | 0.001345088 | 0.011560804 | SYN |  |  |  |  |  |  |  |
| Frzb | -1.493104702 | 6.60394E-14 | 3.27162E-12 |  |  |  |  |  |  |  |  |
| Fscn1 | -0.208428739 | 0.008924533 | 0.054048654 | SYN |  |  |  | FMRP |  |  |  |
| Fsd1l | -0.211860539 | 0.04274689 | 0.184296861 |  |  |  |  |  |  |  |  |
| Fstl1 | 0.40548699 | 0.000128097 | 0.00143497 |  |  |  |  |  |  |  |  |
| Fstl4 | -0.132565185 | 0.283413599 | 0.602319976 |  |  |  |  |  |  |  |  |
| Fstl5 | 0.113738759 | 0.263710091 | 0.579755151 |  |  |  |  |  |  |  |  |
| Fth1 | 0.387669962 | 0.017034761 | 0.089505439 | SYN |  |  |  |  |  |  |  |
| Ftl1 | 0.242556359 | 0.217340312 | 0.526102072 |  |  |  |  |  |  |  |  |
| Fto | -0.018509579 | 0.884883655 | 0.962081792 |  |  |  |  |  |  |  |  |
| Ftsjd2 | -0.100386693 | 0.194366199 | 0.495687842 |  |  |  |  |  |  |  |  |
| Ftx | 0.192192685 | 0.066444694 | 0.249982492 |  |  |  |  |  |  |  |  |
| Fubp1 | 0.031566639 | 0.88338999 | 0.961506354 |  |  |  |  |  |  |  |  |
| Fubp3 | -0.075243983 | 0.238429033 | 0.553100475 |  |  |  |  |  |  |  |  |
| Fuk | 0.115279839 | 0.418634749 | 0.726034085 |  |  |  |  |  |  |  |  |
| Fundc2 | 0.047090686 | 0.531516354 | 0.79708594 |  |  |  |  |  |  |  |  |
| Fuom | 0.343328986 | 0.00249643 | 0.019350368 |  |  |  |  |  |  |  |  |
| Furin | 0.194614191 | 0.042322091 | 0.182761776 |  |  |  |  |  |  | SZ_108 | SZ_full |
| Fus | 0.036327049 | 0.745928508 | 0.912020725 | SYN |  |  |  |  |  |  |  |
| Fut11 | -0.010854094 | 0.765887561 | 0.920286747 |  |  |  |  |  |  |  |  |
| Fut8 | 0.037264673 | 0.48020668 | 0.766025695 |  |  |  |  |  |  |  |  |
| Fut9 | 0.482455243 | 1.90748E-06 | 3.1382E-05 |  |  |  |  |  |  | SZ_108 | SZ_full |
| Fuz | -0.00422819 | 0.767366328 | 0.920286747 |  |  |  |  |  |  |  |  |
| Fxn | -0.08822537 | 0.487535911 | 0.771851216 |  |  |  |  |  |  |  |  |
| Fxr1 | 0.097544153 | 0.199675894 | 0.50224375 |  |  |  |  |  |  | SZ_108 | SZ_full |
| Fxr2 | 0.076955476 | 0.422961177 | 0.729913577 | SYN |  |  |  |  |  |  |  |
| Fxyd6 | 0.115122251 | 0.507164764 | 0.784399099 | SYN |  |  |  |  | SZdb |  | SZ_full |
| Fxyd7 | -0.212236648 | 0.02426834 | 0.118099011 | SYN |  |  |  |  |  |  |  |
| Fyco1 | 0.137561925 | 0.241461444 | 0.557723052 |  |  |  |  |  |  |  |  |
| Fyn | -0.129026803 | 0.127758323 | 0.386821144 | SYN |  |  |  | FMRP |  |  |  |
| Fyttd1 | 0.071607957 | 0.317092666 | 0.634185332 |  |  |  |  |  |  |  |  |
| Fzd2 | 0.000273435 | 0.639948016 | 0.860142008 |  |  |  |  |  |  |  |  |
| Fzd3 | -0.111639372 | 0.356955292 | 0.675290951 |  |  |  |  |  | SZdb |  | SZ_full |
| G2e3 | 0.322667738 | 0.003799955 | 0.026964809 |  |  |  |  |  |  |  |  |
| G3bp2 | -0.099531736 | 0.462967873 | 0.757441468 | SYN |  |  |  |  |  |  |  |
| G530011O06Rik | -0.245574164 | 0.264977311 | 0.58143683 |  |  |  |  |  |  |  |  |
| G6pc3 | -0.170646651 | 0.07692831 | 0.275859153 |  |  |  |  |  |  |  |  |
| Gaa | -0.028481182 | 0.878942506 | 0.959555967 |  |  |  |  |  |  |  |  |
| Gab1 | 0.280833505 | 0.057078065 | 0.224595286 |  |  |  |  |  |  |  |  |
| Gabarap | 0.219597174 | 0.00923176 | 0.055601128 |  |  |  |  |  |  |  |  |
| Gabarapl1 | -0.012234497 | 0.890553228 | 0.963648426 |  |  |  |  |  |  |  |  |
| Gabarapl2 | 0.033285492 | 0.567630924 | 0.818193721 | SYN |  |  |  |  |  |  |  |
| Gabbr1 | -0.111964065 | 0.261731191 | 0.578754638 | SYN |  |  |  | FMRP | SZdb |  | SZ_full |
| Gabbr2 | -0.215345127 | 0.010970549 | 0.063544735 | SYN |  |  |  | FMRP |  |  |  |
| Gabpa | 0.267929567 | 0.021602289 | 0.108228554 |  |  |  |  |  |  |  |  |
| Gabpb2 | 0.215504984 | 0.102821724 | 0.335952568 |  |  |  |  |  |  |  |  |
| Gabra1 | -0.389560085 | 0.025229325 | 0.121662092 | SYN |  | ASD |  |  | SZdb |  | SZ_full |
| Gabra2 | 0.029350849 | 0.479399378 | 0.765984483 |  |  |  |  |  |  |  |  |
| Gabra3 | 0.047884282 | 0.334980442 | 0.655657425 |  |  | ASD |  |  |  |  |  |
| Gabra4 | 0.007367742 | 0.581061621 | 0.825093019 | SYN |  | ASD | ASD_sc |  |  |  |  |
| Gabra5 | 1.230860674 | 1.03636E-39 | 3.30639E-37 |  |  |  |  |  |  |  |  |
| Gabrb1 | 0.474968767 | 0.000898876 | 0.008137838 |  |  | ASD |  |  |  |  |  |
| Gabrb2 | -0.307662216 | 0.000226521 | 0.002389854 |  |  |  |  |  | SZdb |  | SZ_full |
| Gabrb3 | 0.077759892 | 0.323793054 | 0.641314477 |  |  | ASD | ASD_sc |  |  |  |  |
| Gabrd | -1.277301925 | 5.29167E-35 | 1.14071E-32 |  |  |  |  |  |  |  |  |
| Gabrg1 | -0.233111007 | 0.148416799 | 0.423078052 |  |  |  |  |  |  |  |  |
| Gabrg2 | -0.281270124 | 0.004177902 | 0.029230653 |  |  |  |  |  | SZdb |  | SZ_full |
| Gabrg3 | -0.205032884 | 0.127044204 | 0.385286909 |  |  |  |  |  |  |  |  |
| Gad1 | -0.269999171 | 0.113946459 | 0.359224095 | SYN |  | ASD |  |  | SZdb |  | SZ_full |
| Gad2 | 0.15317276 | 0.034336 | 0.154202668 |  |  |  |  |  |  |  |  |
| Gak | -0.259503618 | 0.006769512 | 0.043438155 | SYN |  |  |  |  |  |  |  |
| Gal3st3 | 0.185774871 | 0.044430936 | 0.189508635 |  |  |  |  |  |  |  |  |
| Galc | 0.147584691 | 0.189726431 | 0.48736168 |  |  |  |  |  |  |  |  |
| Galnt1 | -0.228742614 | 0.026033543 | 0.123892328 |  |  |  |  |  |  |  |  |
| Galnt11 | 0.251191875 | 0.000602211 | 0.005691039 |  |  |  |  |  |  |  |  |
| Galnt13 | 0.042867182 | 0.834253274 | 0.943106438 |  |  | ASD | ASD_sc |  |  |  |  |
| Galnt14 | 1.377005311 | 1.12717E-39 | 3.45781E-37 |  |  | ASD |  |  |  |  |  |
| Galnt16 | 1.021845407 | 5.69544E-27 | 7.2106E-25 |  |  |  |  |  |  |  |  |
| Galnt18 | 0.217549747 | 0.140154199 | 0.409176386 |  |  |  |  |  |  |  |  |
| Galnt7 | -0.182026085 | 0.20886041 | 0.515589796 |  |  |  |  |  |  |  |  |
| Galntl6 | -0.018664881 | 0.569928059 | 0.81993979 |  |  |  |  |  |  |  |  |
| Galt | -0.307464957 | 0.002037354 | 0.016422737 |  | ID |  |  |  |  |  |  |
| Ganab | 0.145416096 | 0.094339573 | 0.31749048 |  |  |  |  |  |  |  |  |
| Gap43 | -0.253757208 | 0.433561428 | 0.734512734 | SYN |  | ASD |  |  |  |  |  |
| Gapdh | 0.14330844 | 0.081010237 | 0.286140772 | SYN |  |  |  |  |  |  |  |
| Gapdhs | -0.139387378 | 0.427463539 | 0.731170745 |  |  |  |  |  |  |  |  |
| Gapvd1 | 0.040735675 | 0.678985862 | 0.878273493 | SYN |  |  |  |  |  |  |  |
| Garnl3 | -0.231396934 | 0.002799786 | 0.021186997 |  |  |  |  | FMRP |  |  |  |
| Gars | -0.219138431 | 0.033854895 | 0.152902969 |  |  |  |  |  |  |  |  |
| Gart | -0.003088045 | 0.804918415 | 0.936945007 |  |  |  |  |  |  |  |  |
| Gas2l1 | 0.237924476 | 0.028017185 | 0.131295574 |  |  |  |  |  |  |  |  |
| Gas2l3 | -0.260009675 | 0.090436633 | 0.30760025 |  |  |  |  |  |  |  |  |
| Gas5 | 0.144226103 | 0.241524164 | 0.557723052 |  |  |  |  |  |  |  |  |
| Gas6 | -0.31036375 | 0.00048511 | 0.00471858 |  |  |  |  |  |  |  |  |
| Gas7 | 0.007421446 | 0.830080627 | 0.942131349 | SYN |  |  |  | FMRP |  |  |  |
| Gas8 | 0.289788682 | 0.082880156 | 0.290956041 |  |  |  |  |  |  |  |  |
| Gatad1 | -0.00113347 | 0.900819861 | 0.968898131 |  |  |  |  |  |  |  |  |
| Gatad2a | 0.076247758 | 0.621669676 | 0.849483867 |  |  |  |  |  |  | SZ_108 | SZ_full |
| Gatm | -0.124747548 | 0.590188384 | 0.830204807 |  | ID | ASD |  |  |  |  |  |
| Gatsl2 | -0.059521911 | 0.667260037 | 0.875092018 |  |  |  |  |  |  |  |  |
| Gba | 0.023492847 | 0.928593531 | 0.98139961 |  |  |  |  |  |  |  |  |
| Gba2 | -0.321042194 | 0.000718846 | 0.006666879 |  |  |  |  |  |  |  |  |
| Gbas | 0.224917422 | 0.015952768 | 0.085223897 | SYN |  |  |  |  |  |  |  |
| Gbe1 | 0.053720136 | 0.719500248 | 0.897994027 |  |  |  |  |  |  |  |  |
| Gbf1 | -0.041030314 | 0.923753269 | 0.978466943 |  |  |  |  | FMRP |  |  |  |
| Gca | -0.366645389 | 0.000651264 | 0.006125568 |  |  |  |  |  |  |  |  |
| Gcat | 0.235589103 | 0.162534949 | 0.445185011 |  |  |  |  |  |  |  |  |
| Gcc2 | -0.058728071 | 0.526018185 | 0.793773756 |  |  |  |  |  |  |  |  |
| Gcdh | 0.045774318 | 0.751836024 | 0.91454844 |  | ID |  |  |  |  |  |  |
| Gclc | 0.072423728 | 0.332617572 | 0.652193686 |  |  |  |  |  |  |  |  |
| Gcn1l1 | -0.011921771 | 0.997841997 | 1 |  |  |  |  | FMRP |  |  |  |
| Gcsh | 0.128947615 | 0.201879267 | 0.505495724 |  | ID |  |  |  |  |  |  |
| Gda | -0.672842257 | 4.61054E-13 | 1.99857E-11 |  |  |  |  |  |  |  |  |
| Gdap1 | -0.16226275 | 0.122776157 | 0.377715609 | SYN |  |  |  |  |  |  |  |
| Gdap1l1 | 0.028058019 | 0.854950419 | 0.95092519 | SYN |  |  |  |  |  |  |  |
| Gdap2 | -0.016749641 | 0.921060143 | 0.977431572 |  |  |  |  |  |  |  |  |
| Gde1 | -0.068585286 | 0.334508123 | 0.655054454 |  |  |  |  |  |  |  |  |
| Gdi1 | 0.040550605 | 0.505212539 | 0.783483666 | SYN | ID |  |  |  |  |  |  |
| Gdpd1 | 0.140601612 | 0.190766687 | 0.48910346 |  |  |  |  |  |  |  |  |
| Gdpd5 | -0.189767295 | 0.202184674 | 0.505741342 |  |  |  |  |  |  |  |  |
| Gemin5 | -0.052959863 | 0.692997557 | 0.884375762 |  |  |  |  |  |  |  |  |
| Gemin7 | 0.142732368 | 0.606734357 | 0.840665356 |  |  |  |  |  |  |  |  |
| Get4 | 0.149877818 | 0.117542586 | 0.36578996 |  |  |  |  |  |  |  |  |
| Gfap | 1.367766459 | 1.41095E-07 | 2.87819E-06 | SYN | ID |  |  |  |  |  |  |
| Gfm1 | 0.089177549 | 0.290285819 | 0.607535998 |  |  |  |  |  |  |  |  |
| Gfm2 | 0.02225313 | 0.789258493 | 0.931423132 |  |  |  |  |  |  |  |  |
| Gfod1 | 0.013292528 | 0.582510114 | 0.825996496 |  |  |  |  |  |  |  |  |
| Gfod2 | 0.003591847 | 0.797338147 | 0.933720314 |  |  |  |  |  |  | SZ_108 | SZ_full |
| Gfpt1 | -0.100512293 | 0.291487333 | 0.60871534 |  |  |  |  |  |  |  |  |
| Gfpt2 | -0.868287632 | 1.02419E-09 | 2.94909E-08 |  |  |  |  |  |  |  |  |
| Gfra4 | 0.142920163 | 0.392481534 | 0.703942594 |  |  |  |  |  |  |  |  |
| Gga1 | -0.005260393 | 0.946301381 | 0.986111813 |  |  |  |  |  |  |  |  |
| Gga2 | 0.311967619 | 0.005553538 | 0.036698439 |  |  |  |  |  |  |  |  |
| Gga3 | 0.007189698 | 0.847199155 | 0.947139334 | SYN |  |  |  |  |  |  |  |
| Ggct | 0.12329742 | 0.436406366 | 0.737296585 |  |  |  |  |  |  |  |  |
| Ggcx | 0.009378584 | 0.823993512 | 0.941950287 |  |  |  |  |  |  |  |  |
| Ggnbp2 | 0.004882756 | 0.944468487 | 0.985962641 |  |  |  |  |  |  |  |  |
| Ggps1 | 0.00451068 | 0.986705041 | 0.996717973 |  |  |  |  |  |  |  |  |
| Ggt7 | -0.116942761 | 0.121994677 | 0.375977411 |  |  |  |  |  |  |  |  |
| Ghitm | 0.048189577 | 0.450167702 | 0.746784025 |  |  |  |  |  |  |  |  |
| Gid4 | -0.099676457 | 0.197323335 | 0.499001559 |  |  |  |  |  |  | SZ_108 | SZ_full |
| Gid8 | 0.067317766 | 0.445513988 | 0.743822819 |  |  |  |  |  |  |  |  |
| Gigyf1 | -0.012098286 | 0.856161003 | 0.951377789 |  |  | ASD | ASD_sc |  |  |  |  |
| Gigyf2 | 0.016480501 | 0.979418173 | 0.993945956 |  |  |  |  |  |  | SZ_108 | SZ_full |
| Gipc1 | -0.266844788 | 0.00607211 | 0.039632691 | SYN |  |  |  |  |  |  |  |
| Git1 | -0.120747653 | 0.202342218 | 0.505918975 | SYN |  |  |  | FMRP |  |  |  |
| Git2 | 0.029387224 | 0.767509828 | 0.920286747 | SYN |  |  |  |  |  |  |  |
| Gja1 | 0.251787113 | 0.00933148 | 0.056059415 | SYN |  |  |  |  |  |  |  |
| Gjc2 | 0.235144974 | 0.405223062 | 0.714899169 |  |  |  |  |  |  |  |  |
| Gjc3 | 0.223334249 | 0.170099452 | 0.458736914 |  |  |  |  |  |  |  |  |
| Gje1 | -0.143024291 | 0.404553747 | 0.713876257 |  |  |  |  |  |  |  |  |
| Gla | 0.183467724 | 0.42392457 | 0.729913577 |  |  |  |  |  |  |  |  |
| Glb1 | 0.021730346 | 0.748871213 | 0.913721401 |  | ID |  |  |  |  |  |  |
| Glcci1 | 0.199871259 | 0.010139417 | 0.059994056 |  |  |  |  |  |  |  |  |
| Glce | -0.178509846 | 0.083422322 | 0.29211416 |  |  |  |  |  |  |  |  |
| Gle1 | -0.014983221 | 0.995882831 | 1 |  |  |  |  |  |  |  |  |
| Glg1 | -0.15004151 | 0.173395672 | 0.462387242 | SYN |  |  |  |  |  |  |  |
| Gli3 | -0.004141899 | 0.937977722 | 0.98530815 |  | ID |  |  |  |  |  |  |
| Glmn | -0.197477089 | 0.03852051 | 0.169042407 |  |  |  |  |  |  |  |  |
| Glo1 | -0.116527477 | 0.207211978 | 0.513427381 |  |  | ASD | ASD_sc |  |  |  |  |
| Glrb | -0.205951683 | 0.034162241 | 0.153811129 |  |  |  |  |  |  |  |  |
| Glrx | -0.042812287 | 0.666839055 | 0.875092018 |  |  |  |  |  |  |  |  |
| Glrx2 | 0.014005386 | 0.930035205 | 0.981434219 |  |  |  |  |  |  |  |  |
| Glrx3 | -0.068667655 | 0.743872193 | 0.911502699 |  |  |  |  |  |  |  |  |
| Glrx5 | 0.114338707 | 0.744663949 | 0.91183125 |  |  |  |  |  |  |  |  |
| Gls | -0.274593906 | 0.00382451 | 0.027090843 | SYN |  |  |  |  |  |  |  |
| Gls2 | 0.059441178 | 0.80603113 | 0.936945007 | SYN |  |  |  |  |  |  |  |
| Glt25d1 | 0.074337979 | 0.437616817 | 0.737692921 |  |  |  |  |  |  |  |  |
| Glt25d2 | 0.161564065 | 0.359257599 | 0.677301536 |  |  |  |  |  |  |  |  |
| Glt8d2 | -1.037701515 | 2.17632E-13 | 9.80698E-12 |  |  |  |  |  |  |  |  |
| Gltscr1 | 0.273563358 | 0.078502335 | 0.27967426 |  |  |  |  |  |  |  |  |
| Gltscr1l | -0.123043261 | 0.155222938 | 0.432873687 |  |  |  |  |  |  |  |  |
| Gltscr2 | 0.157356863 | 0.20082327 | 0.504464218 |  |  |  |  |  |  |  |  |
| Glud1 | 0.097830458 | 0.223083958 | 0.535135253 | SYN |  |  |  |  |  |  |  |
| Glul | -0.029554849 | 0.579165263 | 0.824099372 | SYN |  |  |  | FMRP |  |  |  |
| Glyr1 | -0.197999116 | 0.030877261 | 0.142238389 |  |  |  |  |  |  |  |  |
| Gm10125 | -0.153687007 | 0.123071747 | 0.3782737 |  |  |  |  |  |  |  |  |
| Gm10389 | -0.321961555 | 0.160818423 | 0.442917039 |  |  |  |  |  |  |  |  |
| Gm10406 | -0.097090489 | 0.276951147 | 0.59394256 |  |  |  |  |  |  |  |  |
| Gm10649 | 0.963764891 | 2.4062E-11 | 8.84417E-10 |  |  |  |  |  |  |  |  |
| Gm10845 | 0.14462691 | 0.336376673 | 0.65726123 |  |  |  |  |  |  |  |  |
| Gm10863 | 0.416355551 | 0.10295187 | 0.336121211 |  |  |  |  |  |  |  |  |
| Gm12070 | 0.15034876 | 0.050502247 | 0.207098161 |  |  |  |  |  |  |  |  |
| Gm12657 | 0.272511437 | 0.0068731 | 0.044032003 |  |  |  |  |  |  |  |  |
| Gm129 | 0.115886247 | 0.491230936 | 0.773853814 |  |  |  |  |  |  |  |  |
| Gm13152 | 0.227334522 | 0.008655843 | 0.05278211 |  |  |  |  |  |  |  |  |
| Gm13238 | 0.548204857 | 6.13465E-05 | 0.000744749 |  |  |  |  |  |  |  |  |
| Gm13251 | 0.22851944 | 0.007744538 | 0.048333671 |  |  |  |  |  |  |  |  |
| Gm13298 | -0.737686149 | 0.007062086 | 0.044844001 |  |  |  |  |  |  |  |  |
| Gm13305 | -1.256815624 | 0.000262105 | 0.002725617 |  |  |  |  |  |  |  |  |
| Gm13375 | 0.033245593 | 0.958354225 | 0.989551899 |  |  |  |  |  |  |  |  |
| Gm13498 | 0.145718368 | 0.750071004 | 0.914068732 |  |  |  |  |  |  |  |  |
| Gm13695 | -0.056786058 | 0.904325278 | 0.97025806 |  |  |  |  |  |  |  |  |
| Gm13698 | -0.054991745 | 0.892980477 | 0.96508915 |  |  |  |  |  |  |  |  |
| Gm13889 | 0.155799173 | 0.272312213 | 0.590528061 |  |  |  |  |  |  |  |  |
| Gm14288 | -0.075881972 | 0.320913767 | 0.637688241 |  |  |  |  |  |  |  |  |
| Gm14305 | 0.126318312 | 0.564987989 | 0.816810621 |  |  |  |  |  |  |  |  |
| Gm14308 | 0.025797348 | 1 | 1 |  |  |  |  |  |  |  |  |
| Gm14326 | 0.014669754 | 0.828384687 | 0.94210462 |  |  |  |  |  |  |  |  |
| Gm14393 | 0.130158527 | 0.51282879 | 0.787509131 |  |  |  |  |  |  |  |  |
| Gm14403 | -0.079517833 | 0.429118259 | 0.73153195 |  |  |  |  |  |  |  |  |
| Gm14420 | -0.025781308 | 0.584678421 | 0.827352368 |  |  |  |  |  |  |  |  |
| Gm14430 | 0.051961146 | 0.49495469 | 0.77668892 |  |  |  |  |  |  |  |  |
| Gm14461 | 0.210358638 | 0.129483654 | 0.389721368 |  |  |  |  |  |  |  |  |
| Gm16432 | 0.017689672 | 0.975140231 | 0.99211875 |  |  |  |  |  |  |  |  |
| Gm16515 | 0.19634017 | 0.285858786 | 0.603976073 |  |  |  |  |  |  |  |  |
| Gm17296 | 0.098070499 | 0.745206551 | 0.911928524 |  |  |  |  |  |  |  |  |
| Gm17821 | -0.132764542 | 0.179572939 | 0.471297716 |  |  |  |  |  |  |  |  |
| Gm1821 | 0.078668033 | 0.21322256 | 0.520155963 |  |  |  |  |  |  |  |  |
| Gm20594 | -0.056036295 | 0.957128781 | 0.989355248 |  |  |  |  |  |  |  |  |
| Gm2897 | -0.171827308 | 0.152327302 | 0.428338929 |  |  |  |  |  |  |  |  |
| Gm3696 | -0.065715521 | 0.510566794 | 0.786200163 |  |  |  |  |  |  |  |  |
| Gm3893 | -0.809972557 | 0.000140059 | 0.001555862 |  |  |  |  |  |  |  |  |
| Gm5069 | 0.001601118 | 0.929980663 | 0.981434219 |  |  |  |  |  |  |  |  |
| Gm5113 | -0.02038703 | 0.680436682 | 0.878917067 |  |  |  |  |  |  |  |  |
| Gm514 | -0.028043359 | 0.546808784 | 0.806312971 |  |  |  |  |  |  |  |  |
| Gm5506 | 0.138978137 | 0.204738474 | 0.510215761 |  |  |  |  |  |  |  |  |
| Gm5523 | 0.232457264 | 0.056974981 | 0.224300322 |  |  |  |  |  |  |  |  |
| Gm5577 | 0.095718733 | 0.912808485 | 0.973857742 |  |  |  |  |  |  |  |  |
| Gm5643 | -0.037389225 | 0.95171544 | 0.988251608 |  |  |  |  |  |  |  |  |
| Gm5796 | -0.141241211 | 0.106361039 | 0.344292065 |  |  |  |  |  |  |  |  |
| Gm5801 | 0.079390201 | 0.541809949 | 0.802949862 |  |  |  |  |  |  |  |  |
| Gm5803 | -0.23958391 | 0.28943059 | 0.607535998 |  |  |  |  |  |  |  |  |
| Gm6034 | -0.720149287 | 1.92817E-06 | 3.15773E-05 |  |  |  |  |  |  |  |  |
| Gm608 | -0.117001413 | 0.285148308 | 0.60327398 |  |  |  |  | FMRP |  |  |  |
| Gm6300 | -0.165632033 | 0.314267102 | 0.63154306 |  |  |  |  |  |  |  |  |
| Gm6548 | 0.315364176 | 0.024226808 | 0.117968878 |  |  |  |  |  |  |  |  |
| Gm6623 | -0.130899732 | 0.364056003 | 0.681142548 |  |  |  |  |  |  |  |  |
| Gm6682 | 0.223985777 | 0.004341675 | 0.030191105 |  |  |  |  |  |  |  |  |
| Gm6788 | 0.449736775 | 0.008853898 | 0.053784228 |  |  |  |  |  |  |  |  |
| Gm6900 | -0.258631306 | 0.093271482 | 0.314959077 |  |  |  |  |  |  |  |  |
| Gm6981 | 0.002753672 | 0.693760581 | 0.884924739 |  |  |  |  |  |  |  |  |
| Gm7102 | -0.192160968 | 0.26021623 | 0.577744154 |  |  |  |  |  |  |  |  |
| Gm7120 | -0.016529126 | 0.988329444 | 0.99759678 |  |  |  |  |  |  |  |  |
| Gm8801 | -0.152676781 | 0.386813191 | 0.699121235 |  |  |  |  |  |  |  |  |
| Gm889 | -0.363736271 | 0.193019373 | 0.493278603 |  |  |  |  |  |  |  |  |
| Gm996 | -0.025121453 | 0.939084013 | 0.98530815 |  |  |  |  | FMRP |  |  |  |
| Gmcl1 | -0.059006691 | 0.358303614 | 0.676344578 |  |  |  |  |  |  |  |  |
| Gmeb1 | -0.07856293 | 0.423879462 | 0.729913577 |  |  |  |  |  |  |  |  |
| Gmeb2 | -0.186400135 | 0.165877144 | 0.451061536 |  |  |  |  |  |  |  |  |
| Gmfb | 0.033798428 | 0.399092093 | 0.709155315 |  |  |  |  |  |  |  |  |
| Gmnn | 0.287986923 | 0.211366363 | 0.518502158 |  |  |  |  |  |  |  |  |
| Gmppa | 0.021266292 | 0.95339851 | 0.988856504 |  |  |  |  |  |  |  |  |
| Gmps | 0.025988944 | 0.469111538 | 0.762246238 |  |  |  |  |  |  |  |  |
| Gna11 | 0.161919463 | 0.069760983 | 0.258479285 | SYN |  |  |  |  |  |  |  |
| Gna12 | 0.228707149 | 0.016142347 | 0.085986254 | SYN |  |  |  |  |  |  |  |
| Gna13 | 0.106871492 | 0.170934602 | 0.459498577 | SYN |  |  |  |  |  |  |  |
| Gnai1 | 0.026516887 | 0.481454163 | 0.767012795 | SYN |  |  |  |  |  |  |  |
| Gnai2 | 0.201815235 | 0.013861907 | 0.0761975 | SYN |  |  |  |  |  |  |  |
| Gnai3 | 0.088042623 | 0.486772539 | 0.771519173 | SYN |  |  |  |  |  |  |  |
| Gnal | 0.418757634 | 0.002631229 | 0.020218381 | SYN |  |  |  | FMRP | SZdb |  | SZ_full |
| Gnao1 | 0.08608654 | 0.286363979 | 0.604403042 | SYN |  |  |  | FMRP | SZdb |  | SZ_full |
| Gnaq | -0.031189348 | 0.836387881 | 0.943188599 | SYN |  |  |  |  |  |  |  |
| Gnas | 0.185499945 | 0.014263468 | 0.077921519 | SYN | ID | ASD |  | FMRP | SZdb |  | SZ_full |
| Gnaz | -0.055756026 | 0.539443426 | 0.801975911 | SYN |  |  |  | FMRP |  |  |  |
| Gnb1 | -0.096549437 | 0.351619844 | 0.670296337 | SYN |  |  |  | FMRP |  |  |  |
| Gnb2l1 | 0.115568412 | 0.320592301 | 0.637688241 | SYN |  |  |  |  |  |  |  |
| Gnb4 | -0.201146614 | 0.628600511 | 0.853108334 | SYN |  |  |  |  |  |  |  |
| Gnb5 | 0.048178913 | 0.301735283 | 0.619554862 | SYN |  |  |  |  |  |  |  |
| Gne | 0.089850826 | 0.273525193 | 0.591229524 |  |  |  |  |  |  |  |  |
| Gng12 | -0.069823849 | 0.535096935 | 0.798215669 | SYN |  |  |  |  |  |  |  |
| Gng2 | 0.087496831 | 0.301486189 | 0.619485554 | SYN |  |  |  |  |  |  |  |
| Gng3 | -0.11941747 | 0.059277359 | 0.230632301 | SYN |  |  |  |  |  |  |  |
| Gng7 | -0.257601873 | 0.626235433 | 0.851347165 | SYN |  |  |  |  |  |  |  |
| Gnl1 | -0.176268821 | 0.036534256 | 0.16202145 | SYN |  |  |  |  |  |  |  |
| Gnl2 | -0.190965451 | 0.044086476 | 0.188442517 |  |  |  |  |  |  |  |  |
| Gnl3 | 0.059751792 | 0.725178941 | 0.900427737 |  |  |  |  |  |  | SZ_108 | SZ_full |
| Gnl3l | 0.025601125 | 0.67083723 | 0.87656975 |  |  |  |  |  |  |  |  |
| Gnpat | -0.017208542 | 0.674595972 | 0.878031572 | SYN | ID |  |  |  | SZdb |  | SZ_full |
| Gnpda1 | 0.08963727 | 0.359672254 | 0.677389822 |  | ID |  |  |  |  |  |  |
| Gnpda2 | -0.139644662 | 0.344296594 | 0.66472929 |  |  |  |  |  |  |  |  |
| Gnpnat1 | -0.179568358 | 0.290256773 | 0.607535998 |  |  |  |  |  |  |  |  |
| Gnptab | 0.334086259 | 7.53169E-05 | 0.000889967 |  |  |  |  |  |  |  |  |
| Gnptg | -0.199166602 | 0.015737142 | 0.084354369 |  |  |  |  |  |  |  |  |
| Gns | -0.037321223 | 0.590453166 | 0.83029874 |  | ID |  |  |  |  |  |  |
| Golga1 | 0.002467427 | 0.906240607 | 0.970739161 |  |  |  |  |  |  |  |  |
| Golga2 | 0.081159209 | 0.428341539 | 0.73153195 | SYN |  |  |  |  |  | SZ_108 | SZ_full |
| Golga3 | 0.044443485 | 0.715100356 | 0.896386611 |  |  |  |  |  |  |  |  |
| Golga4 | -0.197631929 | 0.01256267 | 0.070613006 |  |  |  |  |  |  |  |  |
| Golga5 | 0.016822037 | 0.952041208 | 0.988280766 |  |  |  |  |  |  |  |  |
| Golga7 | 0.071829021 | 0.41901946 | 0.726086902 |  |  |  |  |  |  |  |  |
| Golga7b | -0.562679881 | 2.4452E-11 | 8.9463E-10 | SYN |  |  |  |  |  |  |  |
| Golgb1 | -0.069662889 | 0.325592121 | 0.643280346 | SYN |  |  |  |  |  |  |  |
| Golm1 | 0.836349045 | 6.68043E-15 | 3.6747E-13 |  |  |  |  |  |  |  |  |
| Golph3 | -0.071010324 | 0.939191355 | 0.98530815 | SYN |  |  |  |  |  |  |  |
| Gon4l | 0.021614546 | 0.890173028 | 0.963498449 |  |  |  |  |  |  |  |  |
| Gopc | 0.094623818 | 0.207381982 | 0.513529553 |  |  |  |  |  |  |  |  |
| Gorasp1 | 0.017306737 | 0.800988876 | 0.935166555 |  |  |  |  |  |  |  |  |
| Gorasp2 | 0.092666853 | 0.211851615 | 0.518774484 |  |  |  |  |  |  |  |  |
| Gosr1 | 0.044015812 | 0.624286674 | 0.850480636 | SYN |  |  |  |  |  |  |  |
| Gosr2 | 0.01627882 | 0.740928419 | 0.910061682 |  |  |  |  |  |  |  |  |
| Got1 | 0.004593681 | 0.622247053 | 0.849836043 | SYN |  |  |  |  |  |  |  |
| Got2 | 0.052724984 | 0.349962943 | 0.669217079 | SYN |  |  |  |  |  |  |  |
| Gpaa1 | 0.116601611 | 0.369558275 | 0.685616352 |  |  |  |  |  |  |  |  |
| Gpam | 0.137646458 | 0.107357745 | 0.346052663 |  |  |  |  | FMRP |  |  |  |
| Gpatch1 | 0.029409234 | 0.79708032 | 0.933720314 |  |  |  |  |  |  |  |  |
| Gpatch11 | 0.353056836 | 0.009807221 | 0.058331389 |  |  |  |  |  |  |  |  |
| Gpatch2 | 0.031716893 | 0.987879558 | 0.99738321 |  |  |  |  |  |  |  |  |
| Gpatch8 | -0.104635445 | 0.153456181 | 0.430065531 |  |  |  |  |  |  |  |  |
| Gpc1 | 0.519919962 | 3.07709E-07 | 5.91394E-06 | SYN |  |  |  |  |  |  |  |
| Gpc5 | -0.2256879 | 0.024745458 | 0.119908732 |  |  |  |  |  |  |  |  |
| Gpc6 | -0.376864164 | 0.007236877 | 0.045665608 |  |  | ASD | ASD_sc |  |  |  |  |
| Gpcpd1 | 0.046947526 | 0.802720507 | 0.935764216 |  |  |  |  |  |  |  |  |
| Gpd1 | -0.14693805 | 0.297492862 | 0.613920587 |  |  |  |  |  |  |  |  |
| Gpd1l | -0.151276199 | 0.142546543 | 0.412986278 |  |  |  |  |  |  |  |  |
| Gpd2 | 0.004696986 | 0.807261951 | 0.93712146 | SYN |  | ASD |  |  |  |  |  |
| Gphn | 0.183603909 | 0.109896259 | 0.351174905 | SYN |  | ASD |  |  |  |  |  |
| Gpi1 | 0.106042552 | 0.286841746 | 0.604771283 | SYN | ID |  |  |  |  |  |  |
| Gpkow | 0.112533277 | 0.316561552 | 0.63375877 |  |  |  |  |  |  |  |  |
| Gpld1 | -0.003119549 | 0.951824078 | 0.988251608 |  |  |  |  |  |  |  |  |
| Gpm6a | 0.137272439 | 0.067881834 | 0.253276776 | SYN |  |  |  | FMRP |  | SZ_108 | SZ_full |
| Gpm6b | 0.160599966 | 0.111248538 | 0.353936314 |  |  |  |  |  |  |  |  |
| Gpr107 | -0.029617564 | 0.684492311 | 0.881561549 |  |  |  |  |  |  |  |  |
| Gpr108 | -0.053341301 | 0.657239912 | 0.869611321 |  |  |  |  |  |  |  |  |
| Gpr116 | -0.178932787 | 0.165993528 | 0.451095189 |  |  |  |  |  |  |  |  |
| Gpr12 | -1.032151286 | 2.14435E-13 | 9.71779E-12 |  |  |  |  |  |  |  |  |
| Gpr123 | -0.038969604 | 0.914333191 | 0.974441679 |  |  |  |  |  |  |  |  |
| Gpr124 | 0.155915685 | 0.183423775 | 0.477788383 |  |  |  |  |  |  |  |  |
| Gpr125 | -0.181210069 | 0.148974995 | 0.423921291 |  |  |  |  |  |  |  |  |
| Gpr137 | 0.248397653 | 0.009493985 | 0.056892579 |  |  |  |  |  |  |  |  |
| Gpr137c | -0.461959416 | 0.000169608 | 0.001835538 |  |  |  |  |  |  |  |  |
| Gpr146 | -0.028228913 | 0.606757539 | 0.840665356 |  |  |  |  |  |  |  |  |
| Gpr153 | -1.198781284 | 5.10445E-17 | 3.60293E-15 |  |  |  |  |  |  |  |  |
| Gpr155 | 0.24041821 | 0.01785006 | 0.093175445 |  |  |  |  |  |  |  |  |
| Gpr157 | -0.111435037 | 0.289450522 | 0.607535998 |  |  |  |  |  |  |  |  |
| Gpr158 | -0.525984223 | 4.04408E-09 | 1.06454E-07 | SYN |  |  |  | FMRP |  |  |  |
| Gpr162 | 0.056840025 | 0.438696912 | 0.738351249 |  |  |  |  | FMRP |  |  |  |
| Gpr17 | 0.215255022 | 0.055277531 | 0.219677921 |  |  |  |  |  |  |  |  |
| Gpr19 | 0.013510202 | 0.962276748 | 0.990072781 |  |  |  |  |  |  |  |  |
| Gpr26 | -0.299382473 | 0.00288719 | 0.021683831 |  |  |  |  |  |  |  |  |
| Gpr27 | 0.250904919 | 0.127574702 | 0.386601758 |  |  |  |  |  |  |  |  |
| Gpr37 | 0.38017455 | 0.058449413 | 0.22785558 |  |  | ASD |  |  |  |  |  |
| Gpr37l1 | 0.014763076 | 0.916773768 | 0.97516429 |  |  |  |  |  |  |  |  |
| Gpr68 | 0.319334091 | 0.084722078 | 0.295213323 |  |  |  |  |  |  |  |  |
| Gpr85 | 0.283856536 | 0.001258578 | 0.010899473 | SYN |  |  |  |  |  |  |  |
| Gpr88 | -0.61581032 | 0.469048233 | 0.762246238 |  |  |  |  |  |  |  |  |
| Gprasp1 | -0.095813782 | 0.386436163 | 0.698810424 |  |  |  |  |  |  |  |  |
| Gprc5b | 0.188676374 | 0.023746469 | 0.116055048 | SYN |  |  |  |  |  |  |  |
| Gprin1 | 0.025639945 | 0.552389949 | 0.807970335 | SYN |  |  |  | FMRP |  |  |  |
| Gprin3 | -0.052203358 | 0.911830864 | 0.973075056 |  |  |  |  |  |  |  |  |
| Gps1 | -0.034006997 | 0.783931287 | 0.928282627 |  |  |  |  |  |  |  |  |
| Gpsm1 | -0.254468854 | 0.012600005 | 0.070723178 | SYN |  |  |  |  |  |  |  |
| Gpt2 | 0.15899763 | 0.13935033 | 0.407276742 |  |  |  |  |  |  |  |  |
| Gpx1 | 0.198236449 | 0.360572399 | 0.677685543 | SYN |  | ASD | ASD_sc |  |  |  |  |
| Gpx3 | -0.327027571 | 0.387134597 | 0.699153451 |  |  |  |  |  |  |  |  |
| Gpx4 | 0.020340397 | 0.857419287 | 0.951609828 | SYN |  |  |  |  |  |  |  |
| Gramd1a | 0.27482275 | 0.051051286 | 0.208278804 |  |  |  |  |  |  |  |  |
| Gramd1b | -0.025203775 | 0.89810767 | 0.967230188 |  |  |  |  | FMRP |  | SZ_108 | SZ_full |
| Gramd3 | 0.162736401 | 0.335083561 | 0.655698351 |  |  |  |  |  |  |  |  |
| Gramd4 | -0.10310223 | 0.412549737 | 0.721283801 |  |  |  |  |  |  |  |  |
| Grasp | 0.057095045 | 0.676989541 | 0.878273493 |  |  |  |  |  |  |  |  |
| Grb10 | 0.458243616 | 1.8963E-06 | 3.13144E-05 | SYN |  |  |  |  |  |  |  |
| Grb2 | 0.104141145 | 0.114728333 | 0.360553164 | SYN |  |  |  |  |  |  |  |
| Grem2 | 0.175274766 | 0.078240805 | 0.279216401 |  |  |  |  |  |  |  |  |
| Gria1 | 0.635495622 | 2.65757E-09 | 7.18534E-08 | SYN |  |  |  |  | SZdb | SZ_108 | SZ_full |
| Gria2 | 0.223135247 | 0.00708136 | 0.044897398 | SYN |  |  |  |  |  |  |  |
| Gria3 | 0.337951277 | 0.0001107 | 0.001255964 | SYN | ID |  |  |  | SZdb |  | SZ_full |
| Gria4 | -0.316538284 | 0.000113896 | 0.001283103 | SYN |  |  |  |  | SZdb |  | SZ_full |
| Grid1 | -0.056997452 | 0.538288126 | 0.801453443 |  |  | ASD |  |  | SZdb |  | SZ_full |
| Grid2 | 0.2867119 | 0.125221468 | 0.38164556 | SYN |  | ASD | ASD_sc |  |  |  |  |
| Grik1 | -0.325971034 | 0.000916117 | 0.008275144 |  |  |  |  |  |  |  |  |
| Grik2 | -0.208285738 | 0.043987249 | 0.188119196 | SYN |  | ASD | ASD_sc |  | SZdb |  | SZ_full |
| Grik3 | 0.239454911 | 0.007160697 | 0.045256516 |  |  |  |  | FMRP | SZdb |  | SZ_full |
| Grik5 | 0.219076667 | 0.012057937 | 0.068354021 |  |  |  |  | FMRP |  |  |  |
| Grin1 | -0.126419828 | 0.043770516 | 0.187292723 | SYN |  | ASD |  | FMRP | SZdb |  | SZ_full |
| Grin2a | -0.474157529 | 0.008558868 | 0.052270699 | SYN | ID | ASD | ASD_sc | FMRP | SZdb | SZ_108 | SZ_full |
| Grin2b | -0.051505391 | 0.585411439 | 0.827439595 | SYN | ID | ASD | ASD_sc | FMRP | SZdb |  | SZ_full |
| Grin2c | 0.016279015 | 0.958171339 | 0.989551899 |  |  |  |  |  |  |  |  |
| Grin3a | 0.581622353 | 5.16982E-08 | 1.1518E-06 |  |  |  |  |  |  |  |  |
| Grina | 0.172365734 | 0.05150636 | 0.209492467 |  |  |  |  |  |  |  |  |
| Grip1 | 0.075098642 | 0.955490405 | 0.989355248 | SYN |  | ASD | ASD_sc |  |  |  |  |
| Grip2 | -0.269979138 | 0.022069954 | 0.110005586 |  |  |  |  |  |  |  |  |
| Gripap1 | 0.035293222 | 0.982358036 | 0.994693692 |  |  |  |  |  |  |  |  |
| Grk1 | -0.165725509 | 0.428852893 | 0.73153195 |  |  |  |  |  |  |  |  |
| Grk6 | 0.094949223 | 0.339804883 | 0.660719834 |  |  |  |  |  |  |  |  |
| Grlf1 | -0.35896664 | 0.006053963 | 0.039546606 |  |  |  |  | FMRP |  |  |  |
| Grm1 | 0.756389045 | 5.49691E-15 | 3.04468E-13 | SYN |  | ASD |  |  |  |  |  |
| Grm2 | -0.414961634 | 0.00047186 | 0.004612199 | SYN |  |  |  |  |  |  |  |
| Grm3 | -0.259778917 | 0.007964329 | 0.049550303 | SYN |  |  |  |  | SZdb | SZ_108 | SZ_full |
| Grm4 | -0.602888532 | 7.48888E-10 | 2.18796E-08 |  |  | ASD |  | FMRP | SZdb |  | SZ_full |
| Grm5 | 0.228141775 | 0.007578585 | 0.047483734 | SYN |  | ASD |  | FMRP | SZdb |  | SZ_full |
| Grm7 | -0.309270652 | 8.7589E-05 | 0.001024354 | SYN |  |  |  |  | SZdb |  | SZ_full |
| Grn | 0.195154203 | 0.05173016 | 0.210295494 |  |  |  |  |  |  |  |  |
| Grpel1 | 0.010171507 | 0.960989932 | 0.989984854 |  |  |  |  |  |  |  |  |
| Grpel2 | -0.013833291 | 0.915874167 | 0.97483035 |  |  |  |  |  |  |  |  |
| Grsf1 | -0.11913662 | 0.291372411 | 0.608692078 |  |  |  |  |  |  |  |  |
| Gse1 | 0.614748961 | 9.44808E-09 | 2.37722E-07 |  |  |  |  |  |  |  |  |
| Gsg1l | -0.526003634 | 0.000408245 | 0.004039907 |  |  |  |  |  |  |  |  |
| Gsk3a | -0.042360761 | 0.687713988 | 0.8825621 |  |  |  |  |  |  |  |  |
| Gsk3b | -0.027379421 | 0.93939835 | 0.98530815 | SYN |  | ASD |  | FMRP | SZdb |  | SZ_full |
| Gskip | -0.006761526 | 0.813985168 | 0.938538051 |  |  |  |  |  |  |  |  |
| Gsn | 0.302208405 | 0.131686886 | 0.393236467 | SYN |  | ASD |  |  |  |  |  |
| Gspt1 | 0.107498737 | 0.131529921 | 0.393062066 |  |  |  |  |  |  |  |  |
| Gspt2 | 0.090916178 | 0.338747687 | 0.659792809 |  |  |  |  |  |  |  |  |
| Gsr | 0.15857294 | 0.208244362 | 0.515220745 |  |  |  |  |  |  |  |  |
| Gsta4 | 0.324029863 | 0.004282104 | 0.029802844 |  |  |  |  |  |  |  |  |
| Gstcd | -0.181260854 | 0.214561562 | 0.520956779 |  |  |  |  |  |  |  |  |
| Gstm1 | -0.021425343 | 0.945023455 | 0.985962641 |  |  |  | ASD_sc |  | SZdb |  | SZ_full |
| Gstm3 | -0.092520436 | 0.582174514 | 0.825938086 |  |  |  |  |  |  |  |  |
| Gstm5 | 0.205462825 | 0.020955806 | 0.105653292 | SYN |  |  |  |  |  |  |  |
| Gstp1 | 0.479053013 | 0.093209225 | 0.31488216 | SYN |  |  |  |  |  |  |  |
| Gstp2 | 0.553632494 | 0.06800871 | 0.253593955 | SYN |  |  |  |  |  |  |  |
| Gtdc1 | -0.448521725 | 1.1589E-05 | 0.000165061 |  |  |  |  |  |  |  |  |
| Gtdc2 | -0.063370917 | 0.760414985 | 0.918671603 |  |  |  |  |  |  |  |  |
| Gtf2a1 | -0.086742307 | 0.306701453 | 0.624043555 |  |  |  |  |  |  |  |  |
| Gtf2b | -0.004869814 | 0.837989292 | 0.944306667 |  |  |  |  |  |  |  |  |
| Gtf2e1 | 0.069843578 | 0.671479971 | 0.876694099 |  |  |  |  |  |  |  |  |
| Gtf2f1 | 0.245227593 | 0.02789452 | 0.130951554 |  |  |  |  |  |  |  |  |
| Gtf2f2 | -0.061266228 | 0.867703062 | 0.955251846 |  |  |  |  |  |  |  |  |
| Gtf2h1 | 0.072642641 | 0.437581815 | 0.737692921 |  |  |  |  |  |  |  |  |
| Gtf2h5 | 0.056354928 | 0.765890038 | 0.920286747 |  |  |  |  |  |  |  |  |
| Gtf2i | -0.02189089 | 0.797632748 | 0.93391765 |  |  | ASD | ASD_sc |  |  |  |  |
| Gtf2ird1 | -0.414278049 | 0.000111255 | 0.001260471 |  |  |  |  |  |  |  |  |
| Gtf2ird2 | -0.14023741 | 0.228050861 | 0.540872893 |  |  |  |  |  |  |  |  |
| Gtf3a | 0.115661461 | 0.526969878 | 0.794238803 |  |  |  |  |  |  |  |  |
| Gtf3c1 | 0.190396623 | 0.020037583 | 0.101731231 |  |  |  |  | FMRP |  |  |  |
| Gtf3c2 | 0.018151307 | 0.990550752 | 0.998292355 |  |  |  |  | FMRP |  |  |  |
| Gtf3c4 | 0.034986344 | 0.613243843 | 0.844961985 |  |  |  |  |  |  |  |  |
| Gtf3c5 | 0.031008888 | 0.841745943 | 0.945289559 |  |  |  |  |  |  |  |  |
| Gtf3c6 | 0.044934784 | 0.774252859 | 0.923453342 |  |  |  |  |  |  |  |  |
| Gtl3 | -0.050168661 | 0.610515158 | 0.842510679 |  |  |  |  |  |  |  |  |
| Gtpbp1 | 0.065584169 | 0.319999742 | 0.637688241 |  |  |  |  |  |  |  |  |
| Gtpbp2 | 0.005677617 | 0.851323927 | 0.949009034 |  |  |  |  |  |  |  |  |
| Gtpbp3 | 0.01256503 | 0.950391347 | 0.987921463 |  |  |  |  |  |  |  |  |
| Gtpbp4 | 0.009810729 | 0.766595084 | 0.920286747 |  |  |  |  |  |  |  |  |
| Gtpbp5 | 0.03576234 | 0.797125208 | 0.933720314 |  |  |  |  |  |  |  |  |
| Gtpbp6 | 0.241588633 | 0.18593785 | 0.481595848 |  |  |  |  |  |  |  |  |
| Gucy1a2 | 0.035638715 | 0.922972033 | 0.978235671 | SYN |  | ASD |  |  |  |  |  |
| Gucy1a3 | -0.283547538 | 0.084033078 | 0.293582053 |  |  |  |  |  |  |  |  |
| Gucy1b3 | 0.036699629 | 0.399474972 | 0.709407276 |  |  |  |  |  |  |  |  |
| Guf1 | -0.144749178 | 0.076808654 | 0.27583333 |  |  |  |  |  |  |  |  |
| Guk1 | -0.046841044 | 0.606248889 | 0.840653884 | SYN |  |  |  |  |  |  |  |
| Gulp1 | -0.067867692 | 0.969838251 | 0.991454046 |  |  |  |  |  |  |  |  |
| Gxylt1 | 0.084937522 | 0.679626046 | 0.878841981 |  |  |  |  |  |  |  |  |
| Gyk | -0.033934784 | 0.615246528 | 0.845924204 | SYN | ID |  |  |  |  |  |  |
| Gys1 | -0.256086105 | 0.013261144 | 0.073554164 |  |  |  |  |  |  |  |  |
| Gzf1 | -0.150014736 | 0.237430107 | 0.552433645 |  |  |  |  |  |  |  |  |
| H13 | 0.217829038 | 0.052454266 | 0.212481071 |  |  |  |  |  |  |  |  |
| H1f0 | 0.006343937 | 0.699574804 | 0.887191673 |  |  |  |  |  |  |  |  |
| H1fx | 0.39734075 | 0.013256586 | 0.073554164 |  |  |  |  |  |  |  |  |
| H2-T23 | 0.312432764 | 0.028253384 | 0.132092024 |  |  |  |  |  |  |  |  |
| H2-T24 | -0.308633253 | 0.075519532 | 0.271570687 |  |  |  |  |  |  |  |  |
| H2afj | 0.423877975 | 0.003590284 | 0.025775073 |  |  |  |  |  |  |  |  |
| H2afv | -0.037605185 | 0.572391846 | 0.820827732 |  |  |  |  |  |  |  |  |
| H2afx | 0.143596086 | 0.401948083 | 0.711185063 |  |  |  |  |  |  |  |  |
| H2afy | 0.129339418 | 0.202730218 | 0.506730247 |  |  |  |  |  |  |  |  |
| H2afz | -0.092519624 | 0.212586321 | 0.519800275 |  |  |  |  |  |  |  |  |
| H3f3a | 0.2366586 | 0.003529171 | 0.025404937 |  |  |  |  |  |  |  |  |
| H3f3b | 0.340982545 | 0.001760184 | 0.014566415 |  |  |  |  |  |  |  |  |
| H6pd | -0.398993078 | 0.00579021 | 0.038010465 |  |  |  |  |  |  |  |  |
| Habp4 | -0.053378115 | 0.552196725 | 0.807970335 |  |  |  |  |  |  |  |  |
| Hace1 | -0.048144557 | 0.481707975 | 0.767193053 |  |  |  |  |  |  |  |  |
| Hadh | -0.027424578 | 0.877990857 | 0.959555967 | SYN |  |  |  |  |  |  |  |
| Hadha | -0.028963819 | 0.730627105 | 0.903885646 | SYN |  |  |  |  |  |  |  |
| Hadhb | 0.020707751 | 0.973788902 | 0.991836537 | SYN |  |  |  |  |  |  |  |
| Hagh | 0.165403336 | 0.141465547 | 0.411045522 |  |  |  |  |  |  |  |  |
| Haghl | 0.105624714 | 0.222568451 | 0.534706804 |  |  |  |  |  |  |  |  |
| Hap1 | -0.382473257 | 0.10442101 | 0.339527915 |  |  |  |  |  |  |  |  |
| Hapln2 | 0.486483274 | 4.58907E-05 | 0.000567479 | SYN |  |  |  |  |  |  |  |
| Hapln4 | -0.102305056 | 0.369765451 | 0.685616352 |  |  |  |  |  |  | SZ_108 | SZ_full |
| Harbi1 | -0.318105606 | 0.052778857 | 0.213306491 |  |  |  |  |  |  | SZ_108 | SZ_full |
| Hars2 | -0.006885043 | 0.521358639 | 0.791490992 | SYN |  |  |  |  |  | SZ_108 | SZ_full |
| Haus2 | -0.135773852 | 0.381209936 | 0.695268093 |  |  |  |  |  |  |  |  |
| Hax1 | 0.230342828 | 0.054248951 | 0.217104685 |  |  |  |  |  |  |  |  |
| Hba-a2 | -0.104634813 | 0.475619036 | 0.764637921 | SYN |  |  |  |  |  |  |  |
| Hbb-b1 | -0.203466505 | 0.139463649 | 0.407359045 |  |  |  |  |  |  |  |  |
| Hbp1 | 0.083800831 | 0.467823109 | 0.761418327 |  |  |  |  |  |  |  |  |
| Hccs | -0.171417552 | 0.44558584 | 0.743822819 |  | ID |  |  |  |  |  |  |
| Hcfc1 | 0.108251387 | 0.139254747 | 0.407146577 |  | ID | ASD |  | FMRP |  |  |  |
| Hcfc2 | 0.027998972 | 0.890711074 | 0.963688487 |  |  |  |  |  |  |  |  |
| Hcn2 | -0.182603946 | 0.149075252 | 0.423921291 | SYN |  |  |  | FMRP |  |  |  |
| Hdac1 | 0.169842019 | 0.250749543 | 0.568337129 |  |  |  |  |  |  |  |  |
| Hdac11 | 0.197806722 | 0.01815171 | 0.094317942 |  |  |  |  |  |  |  |  |
| Hdac2 | 0.126396874 | 0.109700138 | 0.350688697 |  |  |  |  |  |  |  |  |
| Hdac3 | 0.112475004 | 0.284489231 | 0.602965919 |  |  |  |  |  |  |  |  |
| Hdac4 | -0.396378189 | 0.009437654 | 0.056597539 |  | ID | ASD | ASD_sc | FMRP |  |  |  |
| Hdac5 | 0.053294075 | 0.505295885 | 0.783483666 |  |  |  |  | FMRP |  |  |  |
| Hdac6 | -0.08178916 | 0.296206113 | 0.612617868 |  |  | ASD |  |  |  |  |  |
| Hdac7 | -0.788234173 | 1.58276E-06 | 2.66894E-05 |  |  |  |  |  |  |  |  |
| Hdac9 | -0.77735293 | 9.67489E-10 | 2.7959E-08 |  |  |  |  |  |  |  |  |
| Hdgf | -0.011132524 | 0.979265971 | 0.993945956 |  |  |  |  |  |  |  |  |
| Hdgfrp2 | 0.073084376 | 0.6973927 | 0.886510487 |  |  |  |  |  |  |  |  |
| Hdgfrp3 | -0.072543958 | 0.476293597 | 0.764677481 |  |  |  |  |  |  |  |  |
| Hdhd2 | -0.077440215 | 0.533765653 | 0.79784761 |  |  |  |  |  |  |  |  |
| Hdlbp | -0.009967188 | 0.833826798 | 0.943106438 | SYN |  |  |  | FMRP |  |  |  |
| Hdx | 0.249046923 | 0.360213946 | 0.677462343 |  |  |  |  |  |  |  |  |
| Heatr1 | -0.004703422 | 0.970018597 | 0.991454046 |  |  |  |  |  |  |  |  |
| Heatr3 | 0.102390169 | 0.3174555 | 0.634433743 |  |  |  |  |  |  |  |  |
| Heatr5a | -0.106125703 | 0.482605534 | 0.767979423 |  |  |  |  |  |  |  |  |
| Heatr5b | 0.097187039 | 0.22685448 | 0.53947267 |  |  |  |  | FMRP |  |  |  |
| Heatr6 | 0.100830434 | 0.69744901 | 0.886510487 |  |  |  |  |  |  |  |  |
| Heca | -0.137186446 | 0.635035273 | 0.856243739 |  |  |  |  |  |  |  |  |
| Hectd1 | 0.093394476 | 0.219142988 | 0.529020725 |  |  |  |  |  |  |  |  |
| Hectd2 | -0.234510906 | 0.069143002 | 0.256982566 |  |  |  |  |  |  |  |  |
| Hectd3 | 0.134000636 | 0.160775782 | 0.442917039 |  |  |  |  |  |  |  |  |
| Hecw1 | -0.436689793 | 9.98832E-08 | 2.10203E-06 | SYN |  |  |  |  |  |  |  |
| Hecw2 | -0.41539462 | 0.015498094 | 0.083297034 | SYN |  |  |  |  |  |  |  |
| Helq | 0.009519511 | 0.620653247 | 0.848967638 |  |  |  |  |  |  |  |  |
| Helz | -0.121785624 | 0.199115605 | 0.501490037 |  |  |  |  |  |  |  |  |
| Hepacam | 0.075021389 | 0.469049301 | 0.762246238 |  |  | ASD | ASD_sc |  |  |  |  |
| Herc1 | 0.054305749 | 0.508828893 | 0.785601869 |  |  |  |  | FMRP |  |  |  |
| Herc2 | 0.032039159 | 0.674298397 | 0.878031572 |  |  | ASD |  | FMRP |  |  |  |
| Herc3 | -0.412221149 | 2.80953E-06 | 4.47372E-05 |  |  |  |  |  |  |  |  |
| Herc4 | -0.18558293 | 0.054531051 | 0.217578619 |  |  |  |  |  |  |  |  |
| Herc6 | -0.772573943 | 1.02502E-13 | 4.83763E-12 |  |  |  |  |  |  |  |  |
| Hes6 | 0.126879685 | 0.43070356 | 0.732047375 |  |  |  |  |  |  |  |  |
| Hexa | 0.170614531 | 0.085584834 | 0.297180947 |  | ID |  |  |  |  |  |  |
| Hexb | -0.510905323 | 0.179163343 | 0.471152926 |  | ID |  |  |  |  |  |  |
| Hexdc | 0.052976596 | 0.839709483 | 0.944846439 |  |  |  |  |  |  |  |  |
| Hexim1 | 0.246870474 | 0.006740556 | 0.043287178 |  |  |  |  |  |  |  |  |
| Hey1 | 0.064143529 | 0.293687712 | 0.610791196 |  |  |  |  |  |  |  |  |
| Hgs | 0.035912195 | 0.641341051 | 0.860142008 | SYN |  |  |  |  |  |  |  |
| Hgsnat | -0.048686775 | 0.887582106 | 0.962995126 |  |  |  |  |  |  |  |  |
| Hhatl | -0.250141846 | 0.019155696 | 0.098571506 |  |  |  |  |  |  |  |  |
| Hiat1 | -0.029627089 | 0.893911548 | 0.965225747 |  |  |  |  |  |  |  |  |
| Hiatl1 | 0.241838407 | 0.024061211 | 0.117234098 |  |  |  |  |  |  |  |  |
| Hibadh | -0.03574713 | 0.818726596 | 0.941021502 |  |  |  |  |  |  |  |  |
| Hid1 | -0.001766399 | 0.984846058 | 0.995848833 |  |  |  |  |  |  |  |  |
| Hif1a | -0.127645516 | 0.378529088 | 0.693356305 |  |  |  |  |  |  |  |  |
| Hif1an | -0.010217066 | 0.946786294 | 0.986189928 |  |  |  |  |  |  |  |  |
| Higd1a | -0.08919698 | 0.385681431 | 0.698492545 | SYN |  |  |  |  |  |  |  |
| Higd2a | 0.069641665 | 0.826107118 | 0.941950287 |  |  |  |  |  |  |  |  |
| Hint1 | 0.129098913 | 0.328664005 | 0.646873794 |  |  |  |  |  |  |  |  |
| Hip1 | 0.009596056 | 0.732324776 | 0.905497313 | SYN |  |  |  |  |  |  |  |
| Hip1r | -0.371136203 | 0.000987376 | 0.008848666 | SYN |  |  |  |  |  |  |  |
| Hipk1 | -0.209303829 | 0.150987361 | 0.426634282 |  |  |  |  | FMRP |  |  |  |
| Hipk3 | -0.291816511 | 0.034190964 | 0.153811129 |  |  |  |  | FMRP |  |  |  |
| Hira | -0.153955729 | 0.090210913 | 0.307094426 |  |  |  |  |  |  |  |  |
| Hist1h2be | 0.135525837 | 0.568229099 | 0.818824805 |  |  |  |  |  |  |  |  |
| Hist2h2aa1 | 0.624813107 | 0.044673539 | 0.190174377 |  |  |  |  |  |  |  |  |
| Hivep1 | -0.063595842 | 0.970446557 | 0.991454046 |  |  |  |  | FMRP |  |  |  |
| Hivep2 | -0.171093478 | 0.064020903 | 0.243729228 |  |  |  |  | FMRP |  |  |  |
| Hk1 | 0.041396099 | 0.529475495 | 0.79560975 | SYN |  |  |  | FMRP |  |  |  |
| Hlcs | -0.342315059 | 0.010354085 | 0.060857909 |  | ID |  |  |  |  |  |  |
| Hlf | 0.341272494 | 4.13601E-05 | 0.00052115 |  |  |  |  |  |  |  |  |
| Hltf | 0.03001874 | 0.760947701 | 0.918758533 |  |  |  |  |  |  |  |  |
| Hmbox1 | -0.113435933 | 0.196646167 | 0.498445165 |  |  |  |  |  |  |  |  |
| Hmbs | 0.191957939 | 0.461950196 | 0.756884708 |  |  |  |  |  | SZdb |  | SZ_full |
| Hmg20a | -0.078160232 | 0.370183168 | 0.686008584 |  |  |  |  |  |  |  |  |
| Hmg20b | 0.063402633 | 0.821280818 | 0.941239661 |  |  |  |  |  |  |  |  |
| Hmgb1 | 0.184901936 | 0.054160027 | 0.216857617 |  |  |  |  |  |  |  |  |
| Hmgb1-rs17 | 0.413576963 | 0.067891894 | 0.253276776 |  |  |  |  |  |  |  |  |
| Hmgcl | 0.106802852 | 0.686670651 | 0.882339565 |  | ID |  |  |  |  |  |  |
| Hmgcll1 | -0.237962487 | 0.034702693 | 0.155411949 |  |  |  |  |  |  |  |  |
| Hmgcr | -0.138718049 | 0.428972465 | 0.73153195 |  |  |  |  |  |  |  |  |
| Hmgn1 | 0.10281621 | 0.231923837 | 0.545953202 |  |  | ASD | ASD_sc |  |  |  |  |
| Hmgn2 | 0.228714323 | 0.065688121 | 0.247637225 |  |  |  |  |  |  |  |  |
| Hmgxb3 | -0.09660483 | 0.604745494 | 0.839508853 |  |  |  |  |  |  |  |  |
| Hmgxb4 | -0.054051756 | 0.495928779 | 0.77686598 |  |  |  |  |  |  |  |  |
| Hmox2 | 0.118206094 | 0.340567072 | 0.661237333 | SYN |  |  |  |  |  |  |  |
| Hn1 | -0.212854841 | 0.012307718 | 0.069424582 |  |  |  |  |  |  |  |  |
| Hnmt | 0.060135637 | 0.414995801 | 0.723182545 |  |  |  |  |  |  |  |  |
| Hnrnpa0 | 0.078926538 | 0.293834431 | 0.610791196 |  |  |  |  |  |  |  |  |
| Hnrnpa2b1 | 0.22444176 | 0.003856759 | 0.027222573 |  |  |  |  |  |  |  |  |
| Hnrnpa3 | -0.036172674 | 0.852259339 | 0.9494683 |  |  |  |  |  |  |  |  |
| Hnrnpc | 0.082093744 | 0.314004182 | 0.63143328 |  |  |  |  |  |  |  |  |
| Hnrnpd | 0.007389482 | 0.73743075 | 0.908090941 |  |  |  |  |  |  |  |  |
| Hnrnpf | 0.189256192 | 0.033742965 | 0.152483791 |  |  |  |  |  |  |  |  |
| Hnrnph1 | 0.075175384 | 0.231279888 | 0.545281816 |  |  |  |  |  |  |  |  |
| Hnrnph2 | 0.024969794 | 0.627839354 | 0.852766489 |  |  | ASD |  |  |  |  |  |
| Hnrnph3 | 0.092880001 | 0.786155421 | 0.929112906 |  |  |  |  |  |  |  |  |
| Hnrnpk | 0.001653692 | 0.616600263 | 0.847083641 |  |  |  |  |  |  |  |  |
| Hnrnpl | 0.019841892 | 0.763330194 | 0.919979858 |  |  |  |  |  |  |  |  |
| Hnrnpm | 0.110574851 | 0.228343287 | 0.541238055 | SYN |  |  |  |  |  |  |  |
| Hnrnpr | -0.082292041 | 0.658922986 | 0.87012744 |  |  |  |  |  |  |  |  |
| Hnrnpu | -0.046273476 | 0.641327762 | 0.860142008 |  |  |  |  |  |  |  |  |
| Hnrnpul1 | 0.080589101 | 0.448795913 | 0.746215594 |  |  |  |  | FMRP |  |  |  |
| Hnrnpul2 | 0.028404427 | 0.511694257 | 0.786675674 |  |  |  |  |  |  |  |  |
| Hnrpdl | -0.036942791 | 0.754064862 | 0.915220708 |  |  |  |  |  |  |  |  |
| Hnrpll | 0.247601858 | 0.002438336 | 0.018966688 |  |  |  |  |  |  |  |  |
| Homer1 | -0.19133947 | 0.676566532 | 0.878273493 | SYN |  | ASD |  |  |  |  |  |
| Homer2 | -0.099357157 | 0.371120722 | 0.686947988 | SYN |  |  |  |  |  |  |  |
| Hook1 | 0.033613069 | 0.839555841 | 0.944846439 |  |  |  |  |  |  |  |  |
| Hook2 | -0.200767518 | 0.086188107 | 0.298737274 |  |  |  |  |  |  |  |  |
| Hook3 | 0.017631461 | 0.888739492 | 0.962995126 | SYN |  |  |  |  |  |  |  |
| Hp1bp3 | 0.063338478 | 0.28618155 | 0.604177883 |  |  |  |  |  |  |  |  |
| Hpca | 0.102038871 | 0.227524828 | 0.540261396 | SYN |  |  |  |  |  |  |  |
| Hpcal1 | -0.772435862 | 0.000182889 | 0.001963292 | SYN |  |  |  |  |  |  |  |
| Hpcal4 | 0.521303048 | 4.49462E-05 | 0.000557528 |  |  |  |  |  |  |  |  |
| Hprt | -0.024067111 | 0.816237182 | 0.939945551 |  | ID |  |  |  |  |  |  |
| Hps4 | 0.047169061 | 0.905967875 | 0.970739161 |  |  |  |  |  |  |  |  |
| Hps5 | -0.035007973 | 0.86648589 | 0.954438815 |  |  |  |  |  |  |  |  |
| Hr | -1.076853521 | 2.95122E-26 | 3.46161E-24 |  |  |  |  |  |  |  |  |
| Hras1 | -0.140504661 | 0.174993189 | 0.464227433 |  |  |  |  |  |  |  |  |
| Hrh3 | -1.217713405 | 5.76186E-30 | 8.51048E-28 |  |  |  |  |  |  |  |  |
| Hs2st1 | -0.821400371 | 4.22132E-15 | 2.3545E-13 |  |  |  |  |  |  |  |  |
| Hs3st1 | -0.614264862 | 8.60849E-07 | 1.53605E-05 |  |  |  |  |  |  |  |  |
| Hs3st2 | 0.410167935 | 0.001501945 | 0.01274416 |  |  |  |  |  |  |  |  |
| Hs3st5 | -0.355737632 | 0.021054684 | 0.105947723 |  |  | ASD | ASD_sc |  |  |  |  |
| Hs6st1 | 0.22360993 | 0.011547777 | 0.066025139 |  |  |  |  |  |  |  |  |
| Hs6st2 | 1.106349095 | 1.04842E-34 | 2.20059E-32 |  |  |  |  |  |  |  |  |
| Hs6st3 | -1.342499759 | 0.000220983 | 0.002337615 |  |  |  |  |  |  |  |  |
| Hsbp1 | 0.135301528 | 0.312737118 | 0.630022965 |  |  |  |  |  |  |  |  |
| Hsd11b1 | -0.582771415 | 0.003398863 | 0.024622462 |  |  | ASD | ASD_sc |  |  |  |  |
| Hsd17b11 | 0.06979841 | 0.390750407 | 0.702258955 |  |  |  |  |  |  |  |  |
| Hsd17b12 | -0.116591119 | 0.363056668 | 0.679860924 | SYN |  |  |  |  |  |  |  |
| Hsd17b4 | -0.039382417 | 0.67633435 | 0.878273493 | SYN |  |  |  |  |  |  |  |
| Hsdl1 | -0.021828673 | 0.76644986 | 0.920286747 | SYN |  |  |  |  |  |  |  |
| Hsdl2 | -0.17889743 | 0.085678554 | 0.297376914 | SYN |  |  |  |  |  |  |  |
| Hsf1 | 0.12288541 | 0.371011412 | 0.686905066 |  |  |  |  |  |  |  |  |
| Hsf2 | 0.171673967 | 0.084169031 | 0.293799647 |  |  |  |  |  |  |  |  |
| Hsp90aa1 | -0.025780508 | 0.771366042 | 0.921473506 | SYN |  |  |  |  |  |  |  |
| Hsp90ab1 | 0.211674264 | 0.008213758 | 0.05078522 | SYN |  |  |  | FMRP |  |  |  |
| Hsp90b1 | 0.041736474 | 0.653535541 | 0.868766579 | SYN |  |  |  |  |  |  |  |
| Hspa12a | -0.270229069 | 0.001354186 | 0.011626471 | SYN |  |  |  |  |  |  |  |
| Hspa12b | -0.087405362 | 0.625675466 | 0.850835025 |  |  |  |  |  |  |  |  |
| Hspa13 | 0.009896407 | 0.510218293 | 0.786093307 |  |  |  |  |  |  |  |  |
| Hspa14 | -0.000212878 | 0.848952856 | 0.947758203 |  |  |  |  |  |  |  |  |
| Hspa1a | -0.645990805 | 0.017205905 | 0.090285724 | SYN |  |  |  |  |  |  |  |
| Hspa2 | 0.716720334 | 4.72831E-10 | 1.42852E-08 | SYN |  |  |  |  |  |  |  |
| Hspa4 | 0.122811672 | 0.106547505 | 0.34475574 | SYN |  |  |  |  |  |  |  |
| Hspa4l | 0.034309785 | 0.673469142 | 0.877823808 | SYN |  |  |  |  |  |  |  |
| Hspa5 | 0.371791274 | 0.179416305 | 0.471297716 | SYN |  |  |  |  |  |  |  |
| Hspa8 | 0.072356149 | 0.469699997 | 0.762409271 | SYN |  |  |  |  |  |  |  |
| Hspa9 | 0.118594208 | 0.108675224 | 0.348562975 | SYN |  |  |  |  |  | SZ_108 | SZ_full |
| Hspbp1 | -0.001762976 | 0.788651264 | 0.931066087 |  |  |  |  |  |  |  |  |
| Hspd1 | 0.10567214 | 0.197105087 | 0.498607729 | SYN |  |  |  |  |  | SZ_108 | SZ_full |
| Hspg2 | 0.19073403 | 0.211351235 | 0.518502158 |  |  |  |  |  |  |  |  |
| Hsph1 | -0.122150385 | 0.240510879 | 0.556194483 | SYN |  |  |  |  |  |  |  |
| Htatip2 | -0.003427777 | 0.757382207 | 0.916833457 |  |  |  |  |  |  |  |  |
| Htatsf1 | 0.011831852 | 0.97408877 | 0.991871828 |  |  |  |  |  |  |  |  |
| Htr1b | 2.575663221 | 5.92819E-27 | 7.388E-25 |  |  | ASD | ASD_sc |  |  |  |  |
| Htr2a | 0.406257184 | 0.002701971 | 0.020662439 |  |  | ASD |  |  | SZdb |  | SZ_full |
| Htr2c | 0.156609678 | 0.291203898 | 0.608499422 |  |  |  |  |  | SZdb |  | SZ_full |
| Htr3a | 0.424913605 | 0.013357593 | 0.073883608 |  |  | ASD | ASD_sc |  | SZdb |  | SZ_full |
| Htr5a | 0.061238857 | 0.508276521 | 0.785357135 |  |  |  |  |  | SZdb |  | SZ_full |
| Htr7 | -1.770054275 | 6.61164E-27 | 8.11299E-25 |  |  | ASD |  |  | SZdb |  | SZ_full |
| Htra1 | -0.095257371 | 0.187127408 | 0.483018836 |  |  |  |  |  |  |  |  |
| Htra2 | 0.135894314 | 0.186103389 | 0.481595848 |  |  |  |  |  |  |  |  |
| Htt | -0.156989278 | 0.081252641 | 0.28663028 | SYN |  |  |  | FMRP |  |  |  |
| Hunk | -0.664518706 | 4.06248E-07 | 7.69653E-06 |  |  |  |  |  |  |  |  |
| Huwe1 | 0.013819143 | 0.792706641 | 0.932266023 |  | ID | ASD |  | FMRP |  |  |  |
| Hyls1 | 1.510161606 | 5.85983E-07 | 1.0794E-05 |  |  |  |  |  |  |  |  |
| Hyou1 | 0.126978041 | 0.253495358 | 0.57082975 |  |  |  |  |  |  |  |  |
| Hypk | 0.105044718 | 0.571894212 | 0.820827732 |  |  |  |  |  |  |  |  |
| Iars | 0.03839098 | 0.579887016 | 0.824305622 | SYN |  |  |  |  |  |  |  |
| Iars2 | -0.167951519 | 0.138171387 | 0.405333454 | SYN |  |  |  |  |  |  |  |
| Ica1 | 0.265901831 | 0.074219432 | 0.268477727 | SYN |  | ASD | ASD_sc |  |  |  |  |
| Icam5 | 0.440731268 | 0.019546914 | 0.10019678 | SYN |  |  |  |  |  |  |  |
| Ict1 | 0.130660343 | 0.401335349 | 0.711185063 |  |  |  |  |  |  |  |  |
| Id2 | -0.204035097 | 0.096024296 | 0.320625427 |  |  |  |  |  |  |  |  |
| Id3 | 1.0587262 | 0.000112602 | 0.001272115 |  |  |  |  |  |  |  |  |
| Ide | 0.044293682 | 0.574961679 | 0.821430865 |  |  |  |  |  |  |  |  |
| Idh1 | 0.156013113 | 0.087682174 | 0.301965898 |  |  |  |  |  |  |  |  |
| Idh3a | 0.001370756 | 0.667432732 | 0.875092018 | SYN |  |  |  |  |  |  |  |
| Idh3b | -0.03011739 | 0.688758718 | 0.882941855 | SYN |  |  |  |  |  |  |  |
| Idh3g | -0.023468767 | 0.888871504 | 0.963004498 | SYN |  |  |  |  |  |  |  |
| Idi1 | 0.08101932 | 0.52927511 | 0.795468317 |  |  |  |  |  |  |  |  |
| Idnk | 0.017481576 | 0.768822294 | 0.920494741 |  |  |  |  |  |  |  |  |
| Ids | -0.042620671 | 0.718015649 | 0.897634174 |  | ID |  |  | FMRP |  |  |  |
| Idua | -0.267286952 | 0.051933438 | 0.210906874 |  | ID |  |  |  |  |  |  |
| Ier3 | -0.182452178 | 0.237587196 | 0.552637935 |  |  |  |  |  |  |  |  |
| Ier3ip1 | -0.125818168 | 0.132447961 | 0.394917733 |  |  |  |  |  |  |  |  |
| Ier5 | 0.201502262 | 0.024850659 | 0.120272365 |  |  |  |  |  |  |  |  |
| Iffo1 | 0.048850694 | 0.907287714 | 0.971215515 |  |  |  |  |  |  |  |  |
| Ifit3 | -0.313655639 | 0.18664601 | 0.482557076 |  |  |  |  |  |  |  |  |
| Ifitm10 | 0.402912851 | 0.000698099 | 0.006511004 |  |  |  |  |  |  |  |  |
| Ifltd1 | 0.040961409 | 0.986829827 | 0.996717973 |  |  |  |  |  |  |  |  |
| Ifrd2 | 0.054825347 | 0.879528979 | 0.959752724 |  |  |  |  |  |  |  |  |
| Ift122 | -0.142636531 | 0.223598633 | 0.536045294 |  |  |  |  |  |  |  |  |
| Ift140 | -0.112925274 | 0.175946895 | 0.465765826 |  |  |  |  |  |  |  |  |
| Ift172 | -0.17396374 | 0.068799602 | 0.255944786 |  |  |  |  |  |  |  |  |
| Ift46 | 0.083602214 | 0.432589124 | 0.733800692 |  |  |  |  |  |  |  |  |
| Ift52 | -0.066484717 | 0.613950841 | 0.845371525 |  |  |  |  |  |  |  |  |
| Ift57 | 0.606256261 | 2.77877E-10 | 8.59049E-09 |  |  |  |  |  |  |  |  |
| Ift80 | 0.274205043 | 0.011565725 | 0.066080389 |  |  |  |  |  |  |  |  |
| Ift81 | -0.326478326 | 0.003845848 | 0.027169603 |  |  |  |  |  |  |  |  |
| Ift88 | 0.00718516 | 0.709007115 | 0.892243729 |  |  |  |  |  |  |  |  |
| Igbp1 | -0.096404402 | 0.507081236 | 0.784399099 |  | ID |  |  |  |  |  |  |
| Igdcc4 | 0.112638363 | 0.351714075 | 0.670315762 |  |  |  |  |  |  |  |  |
| Igf1r | -0.05611456 | 0.497535181 | 0.77764856 |  |  |  |  |  |  |  |  |
| Igf2r | -0.042212077 | 0.686624273 | 0.882339565 |  |  |  |  |  |  |  |  |
| Igfbp2 | -0.453096995 | 3.06056E-05 | 0.00039564 |  |  |  |  |  |  |  |  |
| Igfbp4 | -0.8309782 | 2.7334E-18 | 2.00015E-16 |  |  |  |  |  |  |  |  |
| Igfbp5 | -0.10793489 | 0.862180835 | 0.953383383 |  |  |  |  |  |  |  |  |
| Igfbp6 | 0.271693681 | 0.014540995 | 0.079058609 |  |  |  |  |  |  |  |  |
| Igfn1 | -4.359309234 | 1.56258E-28 | 2.07718E-26 |  |  |  |  |  |  |  |  |
| Igsf11 | -0.045689822 | 0.959422378 | 0.989888316 |  |  |  |  |  |  |  |  |
| Igsf21 | -0.02132118 | 0.859912516 | 0.952644879 | SYN |  |  |  |  |  |  |  |
| Igsf3 | -0.574095637 | 2.75782E-05 | 0.000360006 |  |  |  |  |  |  |  |  |
| Igsf8 | 0.234631903 | 0.149950801 | 0.425625478 | SYN |  |  |  |  |  |  |  |
| Igsf9b | 0.036157035 | 0.839754453 | 0.944846439 |  |  |  |  | FMRP |  | SZ_108 | SZ_full |
| Ik | 0.143157995 | 0.159613709 | 0.441275196 |  |  |  |  |  |  | SZ_108 | SZ_full |
| Ikbip | -0.032695656 | 0.579304291 | 0.824099372 |  |  |  |  |  |  |  |  |
| Ikbkap | 0.021992595 | 0.678396241 | 0.878273493 |  |  |  |  |  |  |  |  |
| Ikbkb | 0.002455613 | 0.767245386 | 0.920286747 |  |  |  |  |  |  |  |  |
| Ikbkg | -0.12805111 | 0.354789219 | 0.674076378 |  | ID |  |  |  |  |  |  |
| Il11ra1 | -0.170497481 | 0.086302187 | 0.298761391 |  |  |  |  |  |  |  |  |
| Il17ra | -0.413495876 | 0.000733906 | 0.006790758 |  |  |  |  |  |  |  |  |
| Il1rap | 0.278823703 | 0.03401245 | 0.153290388 |  |  |  |  |  |  |  |  |
| Il31ra | -0.809491935 | 0.166669711 | 0.452008709 |  |  |  |  |  |  |  |  |
| Il34 | -0.171515869 | 0.095867728 | 0.320335566 |  |  |  |  |  |  |  |  |
| Il6st | 0.173350454 | 0.04790063 | 0.200191963 |  |  |  |  |  |  |  |  |
| Ildr2 | -0.628243166 | 1.91242E-09 | 5.29635E-08 |  |  |  |  |  |  |  |  |
| Ilf2 | 0.012968225 | 0.937027749 | 0.98530815 |  |  |  |  |  |  |  |  |
| Ilf3 | 0.060930375 | 0.486922241 | 0.771519173 |  |  |  |  |  |  |  |  |
| Ilk | 0.092039005 | 0.359532223 | 0.677389822 |  |  |  |  |  |  |  |  |
| Ilkap | 0.112255209 | 0.52174865 | 0.791604952 |  |  |  |  |  |  |  |  |
| Immp2l | -0.071714024 | 0.305525423 | 0.62282745 |  |  | ASD |  |  |  | SZ_108 | SZ_full |
| Immt | 0.106481851 | 0.161364209 | 0.44367579 | SYN |  |  |  |  |  |  |  |
| Impa1 | -0.174932902 | 0.144562379 | 0.417023707 | SYN |  |  |  |  |  |  |  |
| Impact | 0.267985082 | 0.000941105 | 0.008462518 |  |  |  |  |  |  |  |  |
| Impad1 | -0.012397697 | 0.97961499 | 0.993945956 |  |  |  |  |  |  |  |  |
| Impdh1 | -0.080403895 | 0.234492123 | 0.548638653 |  |  |  |  |  |  |  |  |
| Impdh2 | 0.226694186 | 0.040579622 | 0.17619572 |  |  |  |  |  |  |  |  |
| Ina | 0.146657638 | 0.079603574 | 0.283055207 | SYN |  |  |  |  |  | SZ_108 | SZ_full |
| Incenp | -0.422784728 | 0.000741017 | 0.006832777 |  |  |  |  |  |  |  |  |
| Inf2 | -0.586654189 | 3.42726E-10 | 1.04735E-08 | SYN |  |  |  |  |  |  |  |
| Ing1 | 0.153109881 | 0.09472461 | 0.318115152 |  |  |  |  |  |  |  |  |
| Ing2 | -0.262937605 | 0.037048428 | 0.163801698 |  |  |  |  |  |  |  |  |
| Ing3 | 0.210671665 | 0.08859535 | 0.303116267 |  |  |  |  |  |  |  |  |
| Ing4 | -0.044216328 | 0.859166487 | 0.952559341 |  |  |  |  |  |  |  |  |
| Inip | -0.007287238 | 0.857334306 | 0.951609828 |  |  |  |  |  |  |  |  |
| Ino80 | -0.150060081 | 0.128131553 | 0.387553002 |  |  |  |  |  |  |  |  |
| Ino80c | -0.113667219 | 0.378029139 | 0.693078766 |  |  |  |  |  |  |  |  |
| Ino80d | -0.119885702 | 0.242217972 | 0.55787772 |  |  |  |  |  |  |  |  |
| Ino80e | 0.121155739 | 0.398951677 | 0.709155315 |  |  |  |  |  |  | SZ_108 | SZ_full |
| Inpp4a | 0.061199848 | 0.571857917 | 0.820827732 |  |  |  |  | FMRP |  |  |  |
| Inpp5a | -0.335479598 | 0.001089544 | 0.009602431 |  |  |  |  |  |  |  |  |
| Inpp5b | 0.009940957 | 0.827747054 | 0.94210462 |  |  |  |  |  |  |  |  |
| Inpp5f | 0.306899646 | 0.010764636 | 0.062580899 |  |  |  |  |  |  |  |  |
| Inpp5j | -0.27379376 | 0.006343375 | 0.041033869 |  |  |  |  |  |  |  |  |
| Inpp5k | 0.037555924 | 0.821966293 | 0.941239661 |  |  |  |  |  |  |  |  |
| Insig1 | 0.013328673 | 0.801929686 | 0.935393732 |  |  |  |  |  |  |  |  |
| Insig2 | -0.112721102 | 0.273268622 | 0.591112429 |  |  |  |  |  |  |  |  |
| Insr | 0.010667193 | 0.907744911 | 0.97125661 |  |  |  |  |  |  |  |  |
| Ints1 | 0.059619904 | 0.378169357 | 0.693078766 |  |  |  |  | FMRP |  |  |  |
| Ints10 | 0.030510511 | 0.983336315 | 0.994693692 |  |  |  |  |  |  |  |  |
| Ints12 | -0.126260106 | 0.604896863 | 0.839508853 |  |  |  |  |  |  |  |  |
| Ints3 | 0.115023251 | 0.297380362 | 0.613847249 |  |  |  |  |  |  |  |  |
| Ints6 | 0.308120908 | 0.001983597 | 0.016097067 |  |  |  |  |  |  |  |  |
| Ints7 | -0.102343412 | 0.240224726 | 0.555855067 |  |  |  |  |  |  |  |  |
| Intu | 0.261576805 | 0.04612812 | 0.194562604 |  |  |  |  |  |  |  |  |
| Invs | 0.269958541 | 0.414558547 | 0.723036433 |  |  |  |  |  |  |  |  |
| Ip6k1 | 0.028255778 | 0.704995034 | 0.889721581 |  |  |  |  |  |  |  |  |
| Ip6k2 | 0.423426022 | 3.53404E-06 | 5.51615E-05 |  |  |  |  |  |  |  |  |
| Ipcef1 | -0.606617798 | 1.0494E-06 | 1.84769E-05 |  |  |  |  |  |  |  |  |
| Ipmk | -0.038740244 | 0.963148747 | 0.990416798 |  |  |  |  |  |  |  |  |
| Ipo11 | 0.125302467 | 0.125753418 | 0.382974136 |  |  |  |  |  |  |  |  |
| Ipo13 | 0.100352325 | 0.236838473 | 0.551700836 |  |  |  |  | FMRP |  |  |  |
| Ipo4 | 0.132441509 | 0.141999635 | 0.411601258 |  |  |  |  | FMRP |  |  |  |
| Ipo5 | 0.32771013 | 2.1535E-05 | 0.00028675 | SYN |  |  |  | FMRP | SZdb |  | SZ_full |
| Ipo7 | 0.077539895 | 0.17163635 | 0.460313224 | SYN |  |  |  |  |  |  |  |
| Ipo9 | -0.191721236 | 0.014586233 | 0.079155314 |  |  |  |  |  |  |  |  |
| Ipp | 0.265891087 | 0.026336252 | 0.125005428 |  |  |  |  |  |  |  |  |
| Iqcb1 | -0.084868695 | 0.496261202 | 0.77686598 |  |  |  |  |  |  |  |  |
| Iqce | -0.030802136 | 0.467867505 | 0.761418327 |  |  |  |  |  |  |  |  |
| Iqgap2 | 0.529514014 | 0.015078094 | 0.081203834 |  |  |  |  |  |  |  |  |
| Iqsec1 | -0.036295423 | 0.770527017 | 0.921167131 | SYN |  |  |  |  |  |  |  |
| Iqsec2 | 0.043575713 | 0.694536874 | 0.885065682 | SYN | ID | ASD |  | FMRP |  |  |  |
| Iqsec3 | -0.032493447 | 0.588752566 | 0.829437611 | SYN |  |  |  | FMRP |  |  |  |
| Irak1 | 0.081363845 | 0.624488333 | 0.850480636 |  |  |  |  |  |  |  |  |
| Irak2 | -0.105252882 | 0.303281616 | 0.621045999 |  |  |  |  |  |  |  |  |
| Ireb2 | 0.019828803 | 0.551471639 | 0.807908309 |  |  |  |  |  |  | SZ_108 | SZ_full |
| Irf2bp1 | 0.20099827 | 0.062201323 | 0.239208173 |  |  |  |  |  |  |  |  |
| Irf2bp2 | -0.013905069 | 0.632713845 | 0.855343327 |  |  |  |  |  |  |  |  |
| Irf2bpl | 0.197774198 | 0.012110109 | 0.068522283 |  |  |  |  |  |  |  |  |
| Irgm1 | -0.140981153 | 0.467294547 | 0.760951676 |  |  |  |  |  |  |  |  |
| Irgq | -0.093336319 | 0.333733805 | 0.65393084 | SYN |  |  |  |  |  |  |  |
| Irs2 | 0.031449946 | 0.650755454 | 0.866383611 |  |  |  |  | FMRP |  |  |  |
| Isca1 | 0.024190052 | 0.595570445 | 0.833964185 |  |  |  |  |  |  |  |  |
| Iscu | 0.19619923 | 0.141397069 | 0.411045522 |  |  |  |  |  |  |  |  |
| Islr2 | 1.423501628 | 1.81685E-34 | 3.71569E-32 |  |  |  |  |  |  |  |  |
| Ispd | -0.008654902 | 0.865549203 | 0.954329616 |  |  |  |  |  |  |  |  |
| Ist1 | 0.154040418 | 0.065092939 | 0.246216022 |  |  |  |  |  |  |  |  |
| Isy1 | 0.130434306 | 0.369525331 | 0.685616352 |  |  |  |  |  |  |  |  |
| Itch | 0.081116735 | 0.503409831 | 0.782106171 |  |  |  |  |  |  |  |  |
| Itfg1 | -0.238136875 | 0.014000865 | 0.076822679 | SYN |  |  |  |  |  |  |  |
| Itga1 | -0.073007279 | 0.99787916 | 1 |  |  |  |  |  |  |  |  |
| Itga3 | -0.339942184 | 0.003508002 | 0.025298213 |  |  |  |  |  |  |  |  |
| Itga6 | -0.042664146 | 0.942871859 | 0.985900058 |  |  |  |  |  |  |  |  |
| Itga7 | 0.637524147 | 3.49889E-07 | 6.67636E-06 |  | ID |  |  |  |  |  |  |
| Itgav | -1.013048241 | 9.63043E-29 | 1.3019E-26 |  |  |  |  |  |  |  |  |
| Itgb1 | -0.013770892 | 0.97432523 | 0.991895958 |  |  |  |  |  |  |  |  |
| Itgb5 | -0.02038149 | 0.933067904 | 0.983116349 |  |  |  |  |  |  |  |  |
| Itih5 | -0.014787375 | 0.998767317 | 1 |  |  |  |  |  |  |  |  |
| Itm2a | -0.047280071 | 0.834831941 | 0.943188599 |  |  |  |  |  |  |  |  |
| Itm2b | 0.090689423 | 0.233479638 | 0.548048187 |  |  |  |  |  |  |  |  |
| Itm2c | 0.429380465 | 1.71425E-06 | 2.8785E-05 |  |  |  |  |  |  |  |  |
| Itpk1 | 0.394602602 | 0.00015902 | 0.00173271 |  |  |  |  |  |  |  |  |
| Itpka | 0.10290238 | 0.261577674 | 0.578754638 |  |  |  |  |  |  |  |  |
| Itpr1 | -0.16066156 | 0.107655509 | 0.346653345 |  |  |  |  | FMRP |  |  |  |
| Itpr2 | -0.007257356 | 0.661740419 | 0.871583757 |  |  |  |  |  |  |  |  |
| Itpr3 | -0.303326563 | 0.097978152 | 0.32534294 |  |  |  |  |  |  |  |  |
| Itsn1 | -0.079690697 | 0.279349546 | 0.597118181 | SYN |  |  |  | FMRP |  |  |  |
| Itsn2 | 0.178986945 | 0.055942776 | 0.221438997 | SYN |  |  |  |  |  |  |  |
| Ivd | 0.122838574 | 0.27865737 | 0.59602338 |  |  |  |  |  |  |  |  |
| Ivns1abp | -0.100480843 | 0.524284025 | 0.7932511 |  |  |  |  |  |  |  |  |
| Iws1 | 0.068901925 | 0.569372202 | 0.819583591 |  |  |  |  |  |  |  |  |
| Izumo4 | 0.151816097 | 0.290132269 | 0.607535998 |  |  |  |  |  |  |  |  |
| Jag2 | -0.4976413 | 4.38619E-06 | 6.77989E-05 |  |  |  |  |  |  |  |  |
| Jagn1 | 0.070561625 | 0.659178099 | 0.870176186 |  |  |  |  |  |  |  |  |
| Jak1 | 0.028897879 | 0.588798476 | 0.829437611 |  |  |  |  | FMRP |  |  |  |
| Jak2 | 0.011717393 | 0.79654256 | 0.933720314 |  |  |  |  |  |  |  |  |
| Jakmip1 | -0.178605962 | 0.042810427 | 0.18447108 |  |  |  |  |  |  |  |  |
| Jakmip2 | -0.085750421 | 0.318480256 | 0.636003635 | SYN |  |  |  |  |  |  |  |
| Jakmip3 | -0.100239375 | 0.306209781 | 0.623679575 |  |  |  |  |  |  |  |  |
| Jam3 | 1.128267578 | 4.37823E-14 | 2.19627E-12 |  |  |  |  |  |  |  |  |
| Jarid2 | 0.183661319 | 0.089722217 | 0.305822395 |  |  | ASD |  |  | SZdb |  | SZ_full |
| Jazf1 | -0.158150443 | 0.11009189 | 0.35151838 |  |  |  |  |  |  |  |  |
| Jdp2 | 0.305738578 | 0.00438698 | 0.030426565 |  |  |  |  |  |  |  |  |
| Jhdm1d | -0.176595804 | 0.069346599 | 0.257619225 |  |  |  |  |  |  |  |  |
| Jkamp | 0.088553939 | 0.467207497 | 0.760951676 |  |  |  |  |  |  |  |  |
| Jmjd1c | -0.031958747 | 0.60654886 | 0.840665356 |  |  | ASD |  |  |  |  |  |
| Jmjd4 | -0.208707032 | 0.15331238 | 0.429813548 |  |  |  |  |  |  |  |  |
| Jmjd6 | 0.197806599 | 0.147554128 | 0.421522823 |  |  |  |  |  |  |  |  |
| Jmjd8 | 0.325945987 | 0.006091012 | 0.039723561 |  |  |  |  |  |  |  |  |
| Josd1 | -0.17240103 | 0.073016787 | 0.265455483 |  |  |  |  |  |  |  |  |
| Josd2 | 0.300417487 | 0.456346939 | 0.75237881 |  |  |  |  |  |  |  |  |
| Jph3 | -0.20637612 | 0.025402389 | 0.121980408 |  |  |  |  | FMRP |  |  |  |
| Jph4 | 0.307255568 | 0.000263621 | 0.002737812 |  |  |  |  | FMRP |  |  |  |
| Jtb | 0.043013337 | 0.69107797 | 0.884120561 |  |  |  |  |  |  |  |  |
| Jun | 0.292089142 | 0.050626513 | 0.207265309 |  |  |  |  |  |  |  |  |
| Junb | 0.768302699 | 0.086223303 | 0.298737274 |  |  |  |  |  |  |  |  |
| Jund | 0.094661897 | 0.774329984 | 0.923453342 |  |  |  |  |  |  |  |  |
| Jup | 0.194412286 | 0.084719654 | 0.295213323 | SYN |  |  |  |  |  |  |  |
| Kalrn | -0.328080995 | 7.90885E-06 | 0.000115958 | SYN |  |  |  | FMRP |  |  |  |
| Kank1 | 0.449758158 | 0.000203125 | 0.00216017 |  |  | ASD |  |  |  |  |  |
| Kank4 | -0.82050948 | 0.004916126 | 0.033342706 |  |  |  |  |  |  |  |  |
| Kansl1 | -0.181411196 | 0.053689178 | 0.215593817 |  |  |  |  |  |  |  |  |
| Kansl1l | 0.231359907 | 0.03034141 | 0.140345915 |  |  |  |  |  |  |  |  |
| Kansl2 | 0.03130303 | 0.801358484 | 0.935393732 |  |  |  |  |  |  |  |  |
| Kansl3 | 0.022304259 | 0.825538001 | 0.941950287 |  |  |  |  |  |  |  |  |
| Kars | 0.169226964 | 0.046032069 | 0.194405615 | SYN |  |  |  |  |  |  |  |
| Kat2a | -0.086530424 | 0.475062115 | 0.76408458 |  |  |  |  |  |  |  |  |
| Kat2b | -0.146737527 | 0.397925922 | 0.708292157 |  |  |  |  |  |  |  |  |
| Kat5 | 0.048694628 | 0.692063978 | 0.884316291 |  |  |  |  |  |  |  |  |
| Kat6a | 0.014147134 | 0.630758917 | 0.854148239 |  |  |  |  |  |  |  |  |
| Kat6b | -0.198945844 | 0.036361788 | 0.161481971 |  | ID |  |  |  |  |  |  |
| Kat7 | 0.020398204 | 0.547783319 | 0.806991235 |  |  |  |  |  |  |  |  |
| Katnal1 | -0.126252339 | 0.171102695 | 0.459500032 |  |  |  |  |  |  |  |  |
| Katnal2 | 0.257729483 | 0.259334863 | 0.576917826 |  |  | ASD | ASD_sc |  |  |  |  |
| Katnb1 | -0.0045565 | 0.996785526 | 1 |  |  |  |  |  |  |  |  |
| Kazn | -0.142262455 | 0.164760435 | 0.449428601 |  |  |  |  |  |  |  |  |
| Kbtbd11 | -0.1139675 | 0.206078102 | 0.511571409 | SYN |  |  |  |  |  |  |  |
| Kbtbd2 | 0.023165439 | 0.569125875 | 0.819376892 |  |  |  |  |  |  |  |  |
| Kbtbd7 | 0.052822027 | 0.328060402 | 0.645916999 |  |  |  |  |  |  |  |  |
| Kcmf1 | -0.188850952 | 0.032961433 | 0.149545157 |  |  |  |  |  |  |  |  |
| Kcna1 | 0.160398586 | 0.164093313 | 0.448091133 | SYN |  |  |  |  |  |  |  |
| Kcna2 | -0.411505201 | 0.001138709 | 0.009987669 | SYN |  |  |  | FMRP |  |  |  |
| Kcnab1 | -0.475420271 | 1.65544E-05 | 0.000226212 | SYN |  |  |  |  |  |  |  |
| Kcnab2 | 0.270238139 | 0.001081782 | 0.009555141 | SYN |  |  |  |  |  |  |  |
| Kcnab3 | -0.947831954 | 2.04833E-09 | 5.6531E-08 |  |  |  |  |  |  |  |  |
| Kcnb1 | -0.375624287 | 2.60263E-05 | 0.000342551 |  |  |  |  | FMRP |  | SZ_108 | SZ_full |
| Kcnb2 | -0.226504309 | 0.154555203 | 0.431810113 |  |  |  |  |  |  |  |  |
| Kcnc1 | -0.019307211 | 0.722692246 | 0.899390444 |  |  |  |  |  |  |  |  |
| Kcnc2 | 0.206485168 | 0.041522437 | 0.1796003 |  |  |  |  |  |  |  |  |
| Kcnc3 | -0.02472704 | 0.657163701 | 0.869611321 |  |  |  |  | FMRP |  |  |  |
| Kcnc4 | -0.415044571 | 3.09336E-05 | 0.000399234 |  |  |  |  |  |  |  |  |
| Kcnd2 | 0.092428845 | 0.455896342 | 0.752289793 |  |  | ASD |  | FMRP |  |  |  |
| Kcnd3 | 0.237590742 | 0.007005621 | 0.044558876 |  |  |  |  |  |  |  |  |
| Kcnf1 | -0.230665773 | 0.08781068 | 0.302147535 |  |  |  |  |  |  |  |  |
| Kcng4 | -0.912046785 | 7.02799E-08 | 1.52638E-06 |  |  |  |  |  |  |  |  |
| Kcnh1 | -1.021419165 | 2.3743E-21 | 1.95231E-19 |  |  |  |  | FMRP |  |  |  |
| Kcnh2 | -0.503397021 | 3.97874E-05 | 0.000503721 |  |  |  |  |  |  |  |  |
| Kcnh3 | -0.38656928 | 4.86746E-06 | 7.46594E-05 |  |  |  |  | FMRP |  |  |  |
| Kcnh4 | -1.56047469 | 4.14028E-12 | 1.61877E-10 |  |  |  |  |  |  |  |  |
| Kcnh5 | -2.47340133 | 5.11162E-71 | 1.01926E-67 |  |  |  |  |  |  |  |  |
| Kcnh7 | -0.497435248 | 7.1232E-06 | 0.000105603 |  |  |  |  | FMRP |  |  |  |
| Kcnip1 | -0.608955502 | 0.185907797 | 0.481595848 |  |  |  |  |  |  |  |  |
| Kcnip2 | -0.601437433 | 1.02152E-06 | 1.80257E-05 |  |  |  |  |  |  |  |  |
| Kcnip3 | -0.492494411 | 0.003001398 | 0.022352145 |  |  |  |  |  |  |  |  |
| Kcnip4 | -0.04526473 | 0.490929966 | 0.773853814 |  |  |  |  |  |  |  |  |
| Kcnj10 | 0.157761011 | 0.618521159 | 0.847941692 |  |  | ASD | ASD_sc |  |  |  |  |
| Kcnj11 | -0.146661585 | 0.459983142 | 0.755524205 |  | ID |  |  |  |  |  |  |
| Kcnj16 | -0.539705734 | 0.002876386 | 0.021643444 |  |  |  |  |  |  |  |  |
| Kcnj2 | 0.001554627 | 0.677417491 | 0.878273493 |  |  | ASD |  |  |  |  |  |
| Kcnj3 | 0.006299555 | 0.909945952 | 0.972262947 |  |  |  |  |  |  |  |  |
| Kcnj4 | -0.34525644 | 0.001612034 | 0.013591526 | SYN |  |  |  |  |  |  |  |
| Kcnj6 | 0.393085763 | 0.006103714 | 0.039758552 |  |  |  |  |  |  |  |  |
| Kcnj9 | -0.317030429 | 0.000798469 | 0.007295066 |  |  |  |  |  |  |  |  |
| Kcnk1 | -0.222755708 | 0.010099371 | 0.059801473 |  |  |  |  |  |  |  |  |
| Kcnk12 | 0.082025036 | 0.563906757 | 0.816283175 |  |  |  |  |  |  |  |  |
| Kcnk2 | -0.041308475 | 0.891727065 | 0.964133804 |  |  |  |  |  |  |  |  |
| Kcnk9 | -0.052310783 | 0.615177207 | 0.845924204 |  |  |  |  |  |  |  |  |
| Kcnma1 | -0.16261409 | 0.124378826 | 0.380472824 | SYN |  | ASD | ASD_sc | FMRP |  |  |  |
| Kcnmb4 | -0.056639659 | 0.814278249 | 0.938538051 |  |  |  |  |  |  |  |  |
| Kcnn1 | -0.057417162 | 0.55760499 | 0.810839999 |  |  |  |  |  |  |  |  |
| Kcnn2 | -0.154077523 | 0.049820799 | 0.20557201 |  |  |  |  |  |  |  |  |
| Kcnq1 | -0.083796766 | 0.235632102 | 0.549854197 |  |  |  |  |  |  |  |  |
| Kcnq1ot1 | -0.106285267 | 0.188608509 | 0.48542803 |  |  |  |  |  |  |  |  |
| Kcnq2 | 0.117886879 | 0.182478299 | 0.475791732 | SYN |  | ASD |  | FMRP |  |  |  |
| Kcnq3 | -0.974441598 | 8.18692E-06 | 0.000119595 |  |  | ASD |  | FMRP |  |  |  |
| Kcnq4 | -0.406844705 | 0.001359403 | 0.011658708 |  |  |  |  |  |  |  |  |
| Kcnq5 | 0.029446873 | 0.874817682 | 0.958600683 |  |  |  |  |  |  |  |  |
| Kcns1 | -0.216062197 | 0.115395106 | 0.362073707 |  |  |  |  |  |  |  |  |
| Kcns2 | -0.056538189 | 0.984861534 | 0.995848833 |  |  |  |  |  |  |  |  |
| Kcnt1 | 0.284584363 | 0.001890558 | 0.015481609 |  |  | ASD |  | FMRP |  |  |  |
| Kcnt2 | -0.599994203 | 5.01437E-10 | 1.50356E-08 |  |  |  |  |  |  |  |  |
| Kcnv1 | -0.284153489 | 0.138336845 | 0.405652454 |  |  |  |  |  |  | SZ_108 | SZ_full |
| Kctd1 | 0.353681371 | 6.90357E-05 | 0.000821834 |  |  |  |  |  |  |  |  |
| Kctd12 | 0.167271433 | 0.047362707 | 0.198510221 | SYN |  |  |  |  |  |  |  |
| Kctd13 | -0.016113191 | 0.879853551 | 0.959752724 |  |  | ASD | ASD_sc |  |  | SZ_108 | SZ_full |
| Kctd15 | -1.263192372 | 7.53729E-19 | 5.56643E-17 |  |  |  |  |  |  |  |  |
| Kctd17 | -0.150805035 | 0.044791851 | 0.190538563 |  |  |  |  |  |  |  |  |
| Kctd2 | -0.305092282 | 0.003787793 | 0.026931532 |  |  |  |  |  |  |  |  |
| Kctd20 | -0.00211644 | 0.977695153 | 0.993086842 |  |  |  |  |  |  |  |  |
| Kctd21 | -0.144767177 | 0.382454226 | 0.69629192 |  |  |  |  |  |  |  |  |
| Kctd3 | -0.02087612 | 0.948012226 | 0.986605625 |  |  |  |  |  |  |  |  |
| Kctd6 | -0.164223128 | 0.134051116 | 0.397988249 |  |  |  |  |  |  |  |  |
| Kctd9 | -0.348064552 | 0.003791327 | 0.026931532 |  |  |  |  |  |  |  |  |
| Kdelc1 | -0.042354148 | 0.590950894 | 0.830403111 |  |  |  |  |  |  |  |  |
| Kdelr1 | 0.157707833 | 0.134044603 | 0.397988249 |  |  |  |  |  |  |  |  |
| Kdelr2 | 0.164320358 | 0.143973458 | 0.416254139 |  |  |  |  |  |  |  |  |
| Kdm1a | -0.102702699 | 0.194558661 | 0.495712359 |  |  |  |  |  |  |  |  |
| Kdm1b | -0.063822519 | 0.618689258 | 0.848007618 |  |  |  |  |  |  |  |  |
| Kdm2a | 0.000378754 | 0.814161884 | 0.938538051 |  |  |  |  |  |  |  |  |
| Kdm2b | 0.155030079 | 0.119606837 | 0.369761293 |  |  |  |  |  |  |  |  |
| Kdm3a | 0.21553342 | 0.015617031 | 0.0837956 |  |  |  |  |  |  |  |  |
| Kdm3b | -0.052021665 | 0.507829402 | 0.785078912 |  |  |  |  |  |  | SZ_108 | SZ_full |
| Kdm4a | -0.021445714 | 0.866402491 | 0.954438815 |  |  |  |  |  |  | SZ_108 | SZ_full |
| Kdm4b | 0.135904286 | 0.149492739 | 0.424627524 |  |  |  |  |  |  |  |  |
| Kdm4c | -0.023459956 | 0.857904495 | 0.951689326 |  |  |  |  |  |  |  |  |
| Kdm5a | -0.007621672 | 0.921317931 | 0.977575072 |  |  |  |  |  |  |  |  |
| Kdm5b | 0.050889881 | 0.570084004 | 0.820016233 |  |  | ASD | ASD_sc |  |  |  |  |
| Kdm5c | 0.03377559 | 0.791657385 | 0.931893103 |  | ID | ASD |  |  |  |  |  |
| Kdm6a | -0.108588073 | 0.374950073 | 0.690191964 |  |  |  |  |  |  |  |  |
| Kdm6b | 0.836957509 | 7.7374E-14 | 3.78611E-12 |  |  | ASD | ASD_sc |  |  |  |  |
| Kdm8 | 0.009216467 | 0.781120616 | 0.92656842 |  |  |  |  |  |  |  |  |
| Kdr | 0.246795657 | 0.138138268 | 0.405333454 |  |  |  |  |  |  |  |  |
| Keap1 | -0.018416812 | 0.966488542 | 0.990416798 |  |  |  |  |  |  |  |  |
| Khdrbs1 | 0.026959655 | 0.654584535 | 0.86959601 |  |  |  |  |  |  |  |  |
| Khdrbs2 | 0.580624853 | 0.038358509 | 0.168473275 |  |  | ASD | ASD_sc |  |  |  |  |
| Khdrbs3 | 0.067517627 | 0.576574126 | 0.822118754 |  |  |  |  |  |  |  |  |
| Khnyn | -0.169572805 | 0.209904441 | 0.517206618 |  |  |  |  |  |  |  |  |
| Khsrp | 0.293271346 | 0.003932175 | 0.027681398 |  |  |  |  |  |  |  |  |
| Kidins220 | -0.154972208 | 0.122718468 | 0.377715609 |  |  |  |  |  |  |  |  |
| Kif13a | 0.039696176 | 0.871711889 | 0.956962054 |  |  |  |  |  |  |  |  |
| Kif13b | 0.054421654 | 0.657915868 | 0.869611321 |  |  |  |  |  |  |  |  |
| Kif16b | -0.326656948 | 0.002357484 | 0.018470816 |  |  |  |  |  |  |  |  |
| Kif1a | -0.066821602 | 0.491323434 | 0.773853814 | SYN |  |  |  | FMRP |  |  |  |
| Kif1b | 0.036318868 | 0.717626179 | 0.897567257 |  |  |  |  | FMRP |  |  |  |
| Kif1c | 0.057636504 | 0.631960514 | 0.854900636 |  |  |  |  |  |  |  |  |
| Kif21a | 0.051179317 | 0.543294214 | 0.804103664 | SYN |  |  |  | FMRP |  |  |  |
| Kif21b | -0.096867413 | 0.215925478 | 0.523471614 |  |  |  |  | FMRP |  |  |  |
| Kif26a | -0.000214397 | 0.818207533 | 0.941021502 |  |  |  |  |  |  |  |  |
| Kif2a | -0.011222087 | 0.784601567 | 0.928282627 | SYN |  |  |  |  | SZdb |  | SZ_full |
| Kif3a | -0.049081843 | 0.550627204 | 0.807666914 | SYN |  |  |  |  |  |  |  |
| Kif3b | -0.099890506 | 0.316012412 | 0.633613625 |  |  |  |  |  |  |  |  |
| Kif3c | -0.090810181 | 0.337164992 | 0.658317742 |  |  |  |  | FMRP |  |  |  |
| Kif5a | 0.218473155 | 0.062650939 | 0.240473481 | SYN |  |  |  | FMRP |  |  |  |
| Kif5b | -0.135442108 | 0.259670887 | 0.576917826 | SYN |  |  |  |  |  |  |  |
| Kif5c | -0.157889362 | 0.056631084 | 0.223277077 | SYN |  | ASD |  | FMRP |  |  |  |
| Kifap3 | 0.058898575 | 0.358438883 | 0.676344578 | SYN |  |  |  |  |  |  |  |
| Kifc2 | 0.083916111 | 0.456978539 | 0.75307042 |  |  |  |  | FMRP |  |  |  |
| Kifc3 | 0.158659512 | 0.100782542 | 0.331946496 |  |  |  |  |  |  |  |  |
| Kifc5b | -0.082894772 | 0.77249138 | 0.922225902 |  |  |  |  |  |  |  |  |
| Kin | 0.17402299 | 0.3383021 | 0.659569187 |  |  |  |  |  |  |  |  |
| Kirrel3 | -0.79712616 | 1.43192E-05 | 0.000198972 |  |  |  |  |  |  |  |  |
| Kit | 0.637184777 | 7.07551E-06 | 0.000105092 |  |  | ASD |  |  |  |  |  |
| Kitl | -0.830501554 | 1.82287E-10 | 5.74672E-09 |  |  |  |  |  |  |  |  |
| Klc1 | 0.152362957 | 0.044629387 | 0.190151704 | SYN |  |  |  | FMRP |  | SZ_108 | SZ_full |
| Klc2 | 0.05446832 | 0.86082145 | 0.952894784 | SYN |  | ASD |  |  |  |  |  |
| Klf13 | 0.503634265 | 7.17377E-10 | 2.11918E-08 |  |  |  |  |  |  |  |  |
| Klf15 | 0.07814097 | 0.703868608 | 0.888802931 |  |  |  |  |  |  |  |  |
| Klf9 | 0.071125233 | 0.291536183 | 0.60871534 |  |  |  |  |  |  |  |  |
| Klhdc10 | 0.160673311 | 0.072451841 | 0.264232227 |  |  |  |  |  |  |  |  |
| Klhdc2 | -0.11241589 | 0.295932466 | 0.612443525 |  |  |  |  |  |  |  |  |
| Klhdc3 | -0.146555291 | 0.088375418 | 0.302697643 |  |  |  |  |  |  |  |  |
| Klhdc5 | -0.038153445 | 0.485552188 | 0.77082338 |  |  |  |  |  |  |  |  |
| Klhdc8b | 0.021265558 | 0.862882953 | 0.953793902 |  |  |  |  |  |  |  |  |
| Klhl11 | -0.106328352 | 0.522673799 | 0.792104544 |  |  |  |  |  |  |  |  |
| Klhl13 | 0.077200934 | 0.461765436 | 0.756884708 |  |  |  |  |  |  |  |  |
| Klhl17 | 0.037700705 | 0.830152849 | 0.942131349 |  |  |  |  |  |  |  |  |
| Klhl2 | 0.014979953 | 0.546614229 | 0.806312971 |  |  |  |  |  |  |  |  |
| Klhl20 | 0.044692193 | 0.763982012 | 0.919979858 |  |  |  |  |  |  |  |  |
| Klhl21 | 0.267578488 | 0.046956707 | 0.197327026 |  |  |  |  |  |  |  |  |
| Klhl22 | -0.012003114 | 0.918286952 | 0.976215315 |  |  |  |  | FMRP |  |  |  |
| Klhl23 | -0.694720202 | 2.76905E-13 | 1.227E-11 |  |  |  |  |  |  |  |  |
| Klhl24 | 0.023146768 | 0.818996778 | 0.941021502 |  |  |  |  |  |  |  |  |
| Klhl26 | 0.110370618 | 0.177300623 | 0.46795161 |  |  |  |  |  |  |  |  |
| Klhl29 | 0.003878088 | 0.79173837 | 0.931893103 |  |  |  |  |  |  |  |  |
| Klhl5 | -0.240528511 | 0.023336601 | 0.114684369 |  |  |  |  |  |  |  |  |
| Klhl7 | 0.278959045 | 0.023005501 | 0.113495842 |  |  |  |  |  |  |  |  |
| Klhl9 | 0.012362891 | 0.640265606 | 0.860142008 |  |  |  |  |  |  |  |  |
| Kndc1 | 0.020656222 | 0.973777974 | 0.991836537 |  |  |  |  | FMRP |  |  |  |
| Kpna1 | -0.061458334 | 0.786813013 | 0.929583853 | SYN |  |  |  |  |  |  |  |
| Kpna2 | 0.035508882 | 0.495318441 | 0.77686598 |  |  |  |  |  |  |  |  |
| Kpna3 | -0.060901374 | 0.777672364 | 0.924797943 |  |  |  |  |  | SZdb |  | SZ_full |
| Kpna4 | 0.035889134 | 0.453558635 | 0.750292953 |  |  |  |  |  |  |  |  |
| Kpna6 | -0.044996896 | 0.851691199 | 0.949153137 |  |  |  |  |  |  |  |  |
| Kpnb1 | -0.030366647 | 0.904894099 | 0.970378259 | SYN |  |  |  |  |  |  |  |
| Kptn | -0.058043609 | 0.478331653 | 0.765984483 |  |  | ASD |  |  |  |  |  |
| Kras | 0.118745728 | 0.147102258 | 0.421136975 | SYN | ID |  |  |  |  |  |  |
| Kremen1 | -0.001320693 | 0.73819445 | 0.908476922 |  |  |  |  |  |  |  |  |
| Krit1 | 0.071122463 | 0.457909364 | 0.754136916 |  |  |  |  |  |  |  |  |
| Krr1 | -0.002943694 | 0.9315099 | 0.982118039 |  |  |  |  |  |  |  |  |
| Krt222 | -0.089786791 | 0.584770426 | 0.827352368 |  |  |  |  |  |  |  |  |
| Krtcap2 | 0.068492065 | 0.830390894 | 0.94226743 |  |  |  |  |  |  |  |  |
| Ksr1 | 0.94640184 | 4.59916E-24 | 4.70294E-22 |  |  |  |  |  |  |  |  |
| Ksr2 | -0.176542064 | 0.289958439 | 0.607535998 |  |  |  |  |  |  |  |  |
| Ktn1 | -0.109052608 | 0.1241398 | 0.380155845 | SYN |  |  |  |  |  |  |  |
| L1cam | -0.164865917 | 0.062486409 | 0.240073024 | SYN | ID |  |  |  | SZdb |  | SZ_full |
| L3mbtl1 | -0.867895527 | 0.005519134 | 0.03653163 |  |  |  |  |  |  |  |  |
| L3mbtl2 | 0.052395048 | 0.519978269 | 0.790843055 |  |  |  |  |  |  | SZ_108 | SZ_full |
| L3mbtl3 | -0.435343956 | 0.000582486 | 0.005537436 |  |  |  |  |  |  |  |  |
| l7Rn6 | 0.165679887 | 0.226390102 | 0.53915805 |  |  |  |  |  |  |  |  |
| Lace1 | -0.239344183 | 0.025660994 | 0.122852394 |  |  |  |  |  |  |  |  |
| Lactb | -0.177073858 | 0.092859011 | 0.313965016 |  |  |  |  |  |  |  |  |
| Lama2 | 0.468824966 | 6.75081E-05 | 0.000804849 |  | ID |  |  |  |  |  |  |
| Lama4 | -1.644672026 | 0.155483127 | 0.433158722 |  |  |  |  |  |  |  |  |
| Lamb1 | 1.220508084 | 2.02497E-06 | 3.29616E-05 |  |  | ASD | ASD_sc |  |  |  |  |
| Lamc1 | 0.302057771 | 0.008475109 | 0.051878334 |  |  |  |  |  |  |  |  |
| Lamp1 | -0.069809948 | 0.511415806 | 0.786598895 |  |  |  |  |  |  |  |  |
| Lamp2 | -0.019208421 | 0.957593764 | 0.989355248 |  | ID |  |  |  |  |  |  |
| Lamp5 | -0.944071583 | 4.46349E-11 | 1.53452E-09 |  |  |  |  |  |  |  |  |
| Lamtor2 | 0.015071468 | 0.98071429 | 0.994381525 |  |  |  |  |  |  |  |  |
| Lamtor3 | 0.059656612 | 0.612194815 | 0.844056326 |  |  |  |  |  |  |  |  |
| Lamtor5 | -0.07141754 | 0.410622157 | 0.719965338 |  |  |  |  |  |  |  |  |
| Lancl1 | -0.034606406 | 0.966214749 | 0.990416798 | SYN |  |  |  |  |  |  |  |
| Lancl2 | -0.249730486 | 0.016336471 | 0.086635433 | SYN |  |  |  |  |  |  |  |
| Laptm4a | 0.070376423 | 0.306519712 | 0.624043555 |  |  |  |  |  |  |  |  |
| Laptm4b | 0.065461533 | 0.531557186 | 0.79708594 |  |  |  |  |  |  |  |  |
| Laptm5 | -0.008319465 | 0.842512456 | 0.94530975 |  |  |  |  |  |  |  |  |
| Large | 0.073257866 | 0.502972203 | 0.782106171 |  | ID |  |  | FMRP |  |  |  |
| Larp1 | -0.035958088 | 0.677465251 | 0.878273493 |  |  |  |  |  |  |  |  |
| Larp4 | -0.020670392 | 0.84095827 | 0.945012317 |  |  |  |  |  |  |  |  |
| Larp4b | 0.020635217 | 0.527179066 | 0.794403973 |  |  |  |  |  |  |  |  |
| Larp6 | 0.120838273 | 0.214443386 | 0.520956779 |  |  |  |  |  |  |  |  |
| Larp7 | -0.027182129 | 0.983345005 | 0.994693692 |  |  |  |  |  |  |  |  |
| Lars | -0.113143821 | 0.163308953 | 0.446572777 |  |  |  |  |  |  |  |  |
| Lars2 | -0.636528667 | 0.173550421 | 0.462387242 |  |  |  |  | FMRP |  |  |  |
| Lasp1 | -0.177267922 | 0.022116259 | 0.110043219 | SYN |  |  |  |  |  |  |  |
| Lats1 | 0.155858888 | 0.151368552 | 0.426634282 |  |  |  |  |  |  |  |  |
| Lats2 | 0.089495135 | 0.269348332 | 0.587615508 |  |  |  |  |  |  |  |  |
| Lbh | -0.002574423 | 0.842481389 | 0.94530975 |  |  |  |  |  |  |  |  |
| Lbr | -0.123064229 | 0.293382659 | 0.610652424 |  |  |  |  |  |  |  |  |
| Lcat | 0.360469955 | 0.009098807 | 0.055038506 | SYN |  |  |  |  |  | SZ_108 | SZ_full |
| Lclat1 | -0.279383117 | 0.009101779 | 0.055038506 |  |  |  |  |  |  |  |  |
| Lcmt2 | -0.055956299 | 0.426097989 | 0.73059413 |  |  |  |  |  |  |  |  |
| Lcorl | -0.565554659 | 2.09667E-05 | 0.000280588 |  |  |  |  |  |  |  |  |
| Lcp1 | -0.155227173 | 0.381768987 | 0.695520658 | SYN |  |  |  |  |  |  |  |
| Ldb1 | 0.160027001 | 0.057878983 | 0.226628753 |  |  |  |  |  |  |  |  |
| Ldb2 | 0.955616334 | 7.01033E-22 | 6.21271E-20 |  |  |  |  |  |  |  |  |
| Ldha | 0.113236128 | 0.172682546 | 0.461777959 | SYN |  |  |  |  |  |  |  |
| Ldhb | -0.43931328 | 4.37427E-07 | 8.24803E-06 | SYN |  |  |  |  |  |  |  |
| Ldlr | 0.167821868 | 0.468198143 | 0.761645602 |  |  |  |  |  |  |  |  |
| Ldlrad3 | 0.045692132 | 0.836524091 | 0.943188599 |  |  |  |  |  |  |  |  |
| Ldlrad4 | 0.02864778 | 0.941285957 | 0.985779516 |  |  |  |  |  |  |  |  |
| Lemd1 | -1.402995826 | 4.46951E-22 | 4.051E-20 |  |  |  |  |  |  |  |  |
| Lemd3 | 0.116692964 | 0.269321172 | 0.587615508 |  |  |  |  |  |  |  |  |
| Leng8 | 0.034669631 | 1 | 1 |  |  |  |  |  |  |  |  |
| Leo1 | -0.093805455 | 0.721831159 | 0.898879832 |  |  |  |  |  |  |  |  |
| Leprel2 | 0.37306614 | 0.015020724 | 0.081004255 |  |  |  |  |  |  |  |  |
| Leprel4 | -0.005528696 | 0.856448238 | 0.951412026 |  |  |  |  |  |  |  |  |
| Leprot | 0.174704737 | 0.182369097 | 0.475791732 |  |  |  |  |  |  |  |  |
| Leprotl1 | -0.217864496 | 0.025102243 | 0.121195819 |  |  |  |  |  |  |  |  |
| Letm1 | 0.085958235 | 0.322728737 | 0.639897456 | SYN |  |  |  |  |  |  |  |
| Letm2 | -0.124786181 | 0.167577667 | 0.453699754 |  |  |  |  |  |  |  |  |
| Letmd1 | 0.013150857 | 0.669041342 | 0.875803996 |  |  |  |  |  |  |  |  |
| Lfng | 0.241701765 | 0.108030484 | 0.347160009 |  |  |  |  |  |  |  |  |
| Lgalsl | -0.134595389 | 0.296827584 | 0.613072033 |  |  |  |  |  |  |  |  |
| Lgi1 | -0.39356219 | 9.95421E-06 | 0.000143831 | SYN |  |  |  |  |  |  |  |
| Lgi2 | 0.032398922 | 0.939420944 | 0.98530815 |  |  |  |  |  |  |  |  |
| Lgi3 | -0.198355102 | 0.184983913 | 0.480596642 |  |  |  |  |  |  |  |  |
| Lgi4 | 0.093906131 | 0.320331945 | 0.637688241 |  |  |  |  |  |  |  |  |
| Lgmn | -0.102804874 | 0.351074215 | 0.669600324 |  |  |  |  |  |  |  |  |
| Lgr4 | -0.537712952 | 2.6974E-06 | 4.31152E-05 |  |  |  |  |  |  |  |  |
| Lhfp | -0.601039166 | 6.51933E-06 | 9.7741E-05 |  |  |  |  |  |  |  |  |
| Lhfpl2 | 0.178324639 | 0.137823055 | 0.405078683 |  |  |  |  |  |  |  |  |
| Lhfpl4 | 0.042191421 | 0.534871516 | 0.798215669 |  |  |  |  | FMRP |  |  |  |
| Lias | -0.147040557 | 0.164993044 | 0.449755476 |  |  |  |  |  |  |  |  |
| Lifr | -0.609132817 | 2.23076E-09 | 6.09335E-08 |  |  |  |  |  |  |  |  |
| Lig3 | -0.110978578 | 0.194868582 | 0.495712359 |  |  |  |  |  |  |  |  |
| Lig4 | -0.118164174 | 0.410407034 | 0.719965338 |  |  |  |  |  |  |  |  |
| Lima1 | -0.125717518 | 0.383434155 | 0.697439184 | SYN |  |  |  |  |  |  |  |
| Limch1 | -0.056586176 | 0.539622164 | 0.802092133 | SYN |  |  |  |  |  |  |  |
| Limd2 | 0.767067985 | 1.69955E-07 | 3.42313E-06 |  |  |  |  |  |  |  |  |
| Lime1 | -0.07679013 | 0.436403805 | 0.737296585 | SYN |  |  |  |  |  |  |  |
| Limk1 | -0.040856606 | 0.729223278 | 0.902532534 |  | ID |  |  |  |  |  |  |
| Limk2 | -0.083570138 | 0.314620706 | 0.631791222 |  |  |  |  |  |  |  |  |
| Lims1 | 0.00789618 | 0.852569203 | 0.9494683 |  |  |  |  |  |  |  |  |
| Lin52 | 0.028286919 | 0.691389733 | 0.884120561 |  |  |  |  |  |  |  |  |
| Lin7a | -0.270704731 | 0.010382436 | 0.060979608 | SYN |  |  |  |  |  |  |  |
| Lin7b | -0.082898644 | 0.355237244 | 0.674451858 | SYN |  | ASD |  |  |  |  |  |
| Lin7c | -0.159902418 | 0.198900488 | 0.501241799 | SYN |  |  |  |  |  |  |  |
| Lingo1 | 0.02644066 | 0.649313224 | 0.865461611 | SYN |  |  |  | FMRP |  |  |  |
| Lingo2 | -0.197171788 | 0.113066468 | 0.357601697 |  |  |  |  |  |  |  |  |
| Lingo3 | 0.139739873 | 0.284574009 | 0.602965919 |  |  |  |  |  |  |  |  |
| Litaf | 0.472864614 | 0.004822263 | 0.03284224 |  |  |  |  |  |  |  |  |
| Lix1 | -0.023712917 | 0.877389866 | 0.959427141 |  |  |  |  |  |  |  |  |
| Llgl1 | 0.105489252 | 0.404227937 | 0.713459178 | SYN |  |  |  | FMRP |  |  |  |
| Lman2 | 0.016664346 | 0.743172736 | 0.911229169 |  |  |  |  |  |  |  |  |
| Lman2l | 0.283547583 | 0.004502887 | 0.031095259 |  |  |  |  |  |  |  |  |
| Lmbr1 | -0.2305678 | 0.014420873 | 0.078512548 |  |  |  |  |  |  |  |  |
| Lmbrd1 | -0.230882062 | 0.008390519 | 0.051399981 |  |  |  |  |  |  |  |  |
| Lmbrd2 | -0.059939143 | 0.764702531 | 0.919979858 |  |  |  |  |  |  |  |  |
| Lmf1 | 0.193744287 | 0.162511792 | 0.445185011 |  |  |  |  |  |  |  |  |
| Lmf2 | 0.133382747 | 0.368884806 | 0.685281419 |  |  |  |  |  |  |  |  |
| Lmnb2 | 0.112506882 | 0.320898072 | 0.637688241 | SYN |  |  |  |  |  |  |  |
| Lmo3 | -0.698346427 | 0.000936311 | 0.008447985 |  |  |  |  |  |  |  |  |
| Lmo4 | 0.028774552 | 0.823971183 | 0.941950287 |  |  |  |  |  |  |  |  |
| Lmtk2 | -0.220519153 | 0.037139597 | 0.16402294 | SYN |  |  |  | FMRP |  |  |  |
| Lmtk3 | 0.221836696 | 0.048042078 | 0.200409841 | SYN |  |  |  | FMRP |  |  |  |
| Lnp | -0.346004123 | 0.000302938 | 0.003093774 |  |  |  |  |  |  |  |  |
| Lnpep | -0.010393897 | 0.939085128 | 0.98530815 |  |  |  |  |  |  |  |  |
| Lnx1 | -0.017375486 | 0.730724003 | 0.903885646 |  |  |  |  |  |  |  |  |
| Loh12cr1 | -0.004958907 | 0.806001759 | 0.936945007 |  |  |  |  |  |  |  |  |
| Lonp1 | 0.095801783 | 0.343077422 | 0.6637042 | SYN |  |  |  |  |  |  |  |
| Lonp2 | -0.080004056 | 0.463049724 | 0.757441468 |  |  |  |  |  |  |  |  |
| Lonrf1 | 0.269114053 | 0.003300701 | 0.024075827 |  |  |  |  |  |  |  |  |
| Lonrf2 | 0.109057641 | 0.097807123 | 0.325045671 |  |  |  |  |  |  |  |  |
| Lonrf3 | -0.025467029 | 0.856192298 | 0.951377789 |  |  |  |  |  |  |  |  |
| Lpar1 | 0.740613279 | 0.004436114 | 0.030713928 |  |  |  |  |  |  |  |  |
| Lpar6 | -0.208382579 | 0.461821982 | 0.756884708 |  |  |  |  |  |  |  |  |
| Lpcat2 | -0.196384609 | 0.132306229 | 0.394642663 |  |  |  |  |  |  |  |  |
| Lpcat4 | -0.328886734 | 0.000763132 | 0.007004306 |  |  |  |  |  |  |  |  |
| Lpgat1 | -0.058364488 | 0.541225912 | 0.802680899 |  |  |  |  |  |  |  |  |
| Lphn1 | -0.150841689 | 0.067468904 | 0.252644122 | SYN |  |  |  | FMRP |  |  |  |
| Lphn2 | -0.121155863 | 0.442157883 | 0.741213325 |  |  |  |  |  |  |  |  |
| Lphn3 | 0.102004805 | 0.261827979 | 0.578808193 | SYN |  |  |  | FMRP |  |  |  |
| Lpin2 | 0.045810094 | 0.4902355 | 0.773684278 |  |  |  |  | FMRP |  |  |  |
| Lpp | -0.069277696 | 0.776075481 | 0.924077297 |  |  |  |  |  |  |  |  |
| Lrba | -0.079311833 | 0.530362926 | 0.796096095 |  |  |  |  |  |  |  |  |
| Lrch1 | -0.344306846 | 0.001153292 | 0.010075199 |  |  |  |  |  |  |  |  |
| Lrch2 | 0.01291874 | 0.84061054 | 0.94499151 |  |  |  |  |  |  |  |  |
| Lrfn1 | -0.039652451 | 0.582526366 | 0.825996496 |  |  |  |  |  |  |  |  |
| Lrfn2 | 0.270759569 | 0.039710239 | 0.173360078 |  |  |  |  |  |  |  |  |
| Lrfn5 | -0.222806727 | 0.009172094 | 0.055379731 |  |  | ASD | ASD_sc |  |  |  |  |
| Lrig1 | 0.074346666 | 0.482618167 | 0.767979423 |  |  |  |  |  |  |  |  |
| Lrig2 | 0.084011596 | 0.413785935 | 0.722640371 |  |  |  |  |  |  |  |  |
| Lrp1 | -0.094672863 | 0.37300314 | 0.688992138 | SYN |  |  |  | FMRP |  | SZ_108 | SZ_full |
| Lrp10 | 0.3197004 | 0.007132179 | 0.045181583 |  |  |  |  |  |  |  |  |
| Lrp11 | -0.083595124 | 0.248996107 | 0.56613254 |  |  |  |  |  |  |  |  |
| Lrp12 | -0.545925646 | 5.83283E-08 | 1.2923E-06 |  |  |  |  |  |  |  |  |
| Lrp1b | 0.390701883 | 0.000260381 | 0.002711227 |  |  |  |  |  |  |  |  |
| Lrp3 | -0.127700247 | 0.197550767 | 0.499157953 |  |  |  |  | FMRP |  |  |  |
| Lrp4 | 0.078123236 | 0.335976407 | 0.656961957 |  |  |  |  |  |  |  |  |
| Lrp6 | -0.014696165 | 0.871485402 | 0.956962054 |  |  |  |  |  |  |  |  |
| Lrp8 | 0.084331653 | 0.277785361 | 0.594636618 |  |  |  |  | FMRP |  |  |  |
| Lrpap1 | 0.303663345 | 0.000171885 | 0.001855147 |  |  |  |  |  |  |  |  |
| Lrpprc | 0.048786636 | 0.601204474 | 0.836949801 | SYN | ID | ASD |  |  |  |  |  |
| Lrrc1 | 0.020705767 | 0.700837411 | 0.887191673 |  |  | ASD | ASD_sc |  |  |  |  |
| Lrrc14 | -0.161636409 | 0.25656843 | 0.574108159 |  |  |  |  |  |  |  |  |
| Lrrc16a | -0.073430097 | 0.374546757 | 0.689927236 |  |  |  |  |  |  |  |  |
| Lrrc16b | 0.541754697 | 2.46625E-06 | 3.96589E-05 |  |  |  |  |  |  |  |  |
| Lrrc17 | -2.728574578 | 0.289606136 | 0.607535998 |  |  |  |  |  |  |  |  |
| Lrrc20 | -0.069698137 | 0.687424425 | 0.882484918 |  |  |  |  |  |  |  |  |
| Lrrc28 | 0.334402646 | 0.005289854 | 0.035425589 |  |  |  |  |  |  |  |  |
| Lrrc3 | -0.559681414 | 0.018044248 | 0.093909404 |  |  |  |  |  |  |  |  |
| Lrrc4 | -0.526674176 | 5.40257E-07 | 9.98984E-06 |  |  |  |  |  |  |  |  |
| Lrrc40 | -0.142705893 | 0.247194028 | 0.564174707 | SYN |  |  |  |  |  |  |  |
| Lrrc47 | 0.121766269 | 0.19414603 | 0.495364279 | SYN |  |  |  |  |  |  |  |
| Lrrc49 | -0.365236301 | 0.00164691 | 0.013801681 |  |  |  |  |  |  |  |  |
| Lrrc4b | 0.143044165 | 0.073845707 | 0.267602616 |  |  |  |  | FMRP |  |  |  |
| Lrrc4c | -0.21103462 | 0.013617556 | 0.075009408 |  |  |  |  |  |  |  |  |
| Lrrc55 | -0.073546219 | 0.42110597 | 0.72810345 |  |  |  |  |  |  |  |  |
| Lrrc57 | 0.075837194 | 0.567675233 | 0.818193721 | SYN |  |  |  |  |  |  |  |
| Lrrc58 | 0.038238818 | 0.3884542 | 0.699866884 |  |  |  |  |  |  |  |  |
| Lrrc59 | 0.156299537 | 0.066747286 | 0.250648 | SYN |  |  |  |  |  |  |  |
| Lrrc7 | -0.084657386 | 0.225481025 | 0.538695632 | SYN |  | ASD |  | FMRP |  |  |  |
| Lrrc73 | 0.112376294 | 0.380225312 | 0.694930589 |  |  |  |  |  |  |  |  |
| Lrrc8a | 0.006911083 | 0.993122987 | 0.999619397 |  |  |  |  |  |  |  |  |
| Lrrc8b | -0.560574976 | 6.41134E-09 | 1.639E-07 |  |  |  |  | FMRP |  |  |  |
| Lrrc8d | -0.152501603 | 0.211080097 | 0.518502158 |  |  |  |  |  |  |  |  |
| Lrrcc1 | -0.274226203 | 0.100237363 | 0.330779152 |  |  |  |  |  |  |  |  |
| Lrrfip1 | 0.087331424 | 0.518160818 | 0.790287905 |  |  |  |  |  |  |  |  |
| Lrrfip2 | -0.256093956 | 0.00199633 | 0.016181636 |  |  |  |  |  |  |  |  |
| Lrriq1 | -0.000157093 | 1 | 1 |  |  |  |  |  |  |  |  |
| Lrrk2 | -0.344331473 | 0.044891106 | 0.19085899 |  |  |  |  |  |  |  |  |
| Lrrn1 | 0.539952072 | 1.96763E-08 | 4.71286E-07 |  |  |  |  |  |  |  |  |
| Lrrn2 | 0.518245725 | 5.25656E-10 | 1.57028E-08 |  |  |  |  | FMRP |  |  |  |
| Lrrn3 | 0.353581953 | 3.56087E-05 | 0.000455152 |  |  |  |  |  |  |  |  |
| Lrrtm2 | 0.228271819 | 0.03156672 | 0.144698944 |  |  |  |  |  |  |  |  |
| Lrrtm4 | -1.067560525 | 1.49472E-09 | 4.15397E-08 |  |  |  |  |  |  |  |  |
| Lrsam1 | -0.151329084 | 0.057430228 | 0.225647043 | SYN |  |  |  |  |  |  |  |
| Lrtm2 | -0.447277873 | 7.43921E-06 | 0.00010988 |  |  |  |  |  |  |  |  |
| Lsamp | 0.087475962 | 0.843681257 | 0.945776768 | SYN |  |  |  |  |  |  |  |
| Lsg1 | 0.150188028 | 0.218237187 | 0.527792543 |  |  |  |  |  |  |  |  |
| Lsm11 | -0.041797176 | 0.751671829 | 0.91454844 |  |  |  |  |  |  |  |  |
| Lsm12 | 0.001442064 | 0.825291129 | 0.941950287 |  |  |  |  |  |  |  |  |
| Lsm14a | -0.018939779 | 0.774583686 | 0.923617803 |  |  |  |  |  |  |  |  |
| Lsm14b | 0.029794038 | 0.5516941 | 0.807970335 |  |  |  |  |  |  |  |  |
| Lsm2 | 0.080521847 | 0.831975464 | 0.943106438 |  |  |  |  |  |  |  |  |
| Lsm7 | 0.01989097 | 0.872238174 | 0.957337509 |  |  |  |  |  |  |  |  |
| Lss | 0.100662499 | 0.466397336 | 0.760422149 |  |  |  |  |  |  |  |  |
| Lta4h | 0.156078478 | 0.114676064 | 0.360553164 |  |  |  |  |  |  |  |  |
| Ltbp3 | 0.0807375 | 0.412847903 | 0.721580052 |  |  |  |  |  |  |  |  |
| Ltbp4 | 0.153933782 | 0.08909514 | 0.304074813 |  |  |  |  |  |  |  |  |
| Ltbr | -0.161126053 | 0.62142315 | 0.849292502 |  |  |  |  |  |  |  |  |
| Ltn1 | 0.03831392 | 0.489942775 | 0.77362416 |  |  |  |  |  |  |  |  |
| Luc7l | 0.10911955 | 0.22223744 | 0.534227191 |  |  |  |  |  |  |  |  |
| Luc7l2 | 0.014504476 | 0.952777118 | 0.988597671 |  |  |  |  |  |  |  |  |
| Luc7l3 | -0.034299005 | 0.513110817 | 0.787790544 |  |  |  |  |  |  |  |  |
| Luzp1 | 0.172803266 | 0.065658273 | 0.247637225 |  |  |  |  |  |  |  |  |
| Luzp2 | -0.732765312 | 5.97402E-17 | 4.17972E-15 |  |  |  |  |  |  | SZ_108 | SZ_full |
| Lxn | 0.691211578 | 3.86135E-09 | 1.02319E-07 |  |  |  |  |  |  |  |  |
| Ly6c1 | -0.153590598 | 0.418294017 | 0.725853593 |  |  |  |  |  |  |  |  |
| Ly6e | -0.379769866 | 0.00037723 | 0.003770414 |  |  |  |  |  |  |  |  |
| Ly6h | 0.401827778 | 0.047405278 | 0.198584297 | SYN |  |  |  |  |  |  |  |
| Ly86 | -0.083913739 | 0.784317025 | 0.928282627 |  |  |  |  |  |  |  |  |
| Lynx1 | -0.071767748 | 0.354442421 | 0.673785884 |  |  |  |  | FMRP |  |  |  |
| Lypd1 | -0.884247407 | 1.20376E-07 | 2.47454E-06 |  |  |  |  |  |  |  |  |
| Lypd6 | -0.161431117 | 0.193319912 | 0.493888411 |  |  |  |  |  |  |  |  |
| Lypla1 | -0.199178429 | 0.145756263 | 0.418786728 |  |  |  |  |  |  |  |  |
| Lypla2 | 0.12887683 | 0.162770907 | 0.445678253 |  |  |  |  |  |  |  |  |
| Lyrm5 | -0.266663957 | 0.053763303 | 0.215593817 |  |  |  |  |  |  |  |  |
| Lyrm9 | -0.170586542 | 0.152451709 | 0.428338929 |  |  |  |  |  |  |  |  |
| Lysmd2 | -0.042096737 | 0.755902813 | 0.916153715 |  |  |  |  |  |  |  |  |
| Lysmd3 | 0.093230357 | 0.44449387 | 0.743066334 |  |  |  |  |  |  |  |  |
| Lysmd4 | -0.110509693 | 0.457260636 | 0.753379638 |  |  |  |  |  |  |  |  |
| Lyst | 0.176936848 | 0.076890157 | 0.275859153 |  |  |  |  |  |  |  |  |
| Lyz2 | -0.044453482 | 0.68579901 | 0.882247243 |  |  |  |  |  |  |  |  |
| Lztfl1 | 0.136993849 | 0.121872427 | 0.375745837 |  |  |  |  |  |  |  |  |
| Lztr1 | 0.356158955 | 1.16103E-05 | 0.000165069 |  |  |  |  |  |  |  |  |
| Lzts1 | 0.426779157 | 0.000287051 | 0.002944201 | SYN |  |  |  |  |  |  |  |
| Lzts2 | -0.027839943 | 0.895511647 | 0.965840329 |  |  | ASD | ASD_sc |  |  |  |  |
| M6pr | -0.011250498 | 0.940825448 | 0.985779516 |  |  |  |  |  |  |  |  |
| Macf1 | 0.024249824 | 0.836094258 | 0.943188599 | SYN |  |  |  | FMRP |  |  |  |
| Macrod2 | -0.342033969 | 0.00921576 | 0.055559262 |  |  | ASD | ASD_sc |  |  |  |  |
| Mad2l1bp | -0.104706915 | 0.514904932 | 0.788117778 |  |  |  |  |  |  |  |  |
| Madd | -0.008157375 | 0.920115309 | 0.977078911 | SYN |  |  |  | FMRP |  |  |  |
| Maea | -0.057142032 | 0.699393533 | 0.887191673 |  |  |  |  |  |  |  |  |
| Maf | -0.425530137 | 0.00014729 | 0.001615931 |  |  |  |  |  |  |  |  |
| Maf1 | 0.052090405 | 0.565131631 | 0.816870222 |  |  |  |  |  |  |  |  |
| Mafg | 0.087597214 | 0.409674748 | 0.71925287 |  |  |  |  |  |  |  |  |
| Mafk | -0.019443336 | 0.976842092 | 0.992822463 |  |  |  |  |  |  |  |  |
| Mag | 0.394930325 | 0.093029404 | 0.314407851 | SYN |  |  |  |  | SZdb |  | SZ_full |
| Maged1 | 0.364655076 | 3.08432E-06 | 4.86177E-05 |  |  | ASD |  | FMRP |  |  |  |
| Maged2 | 0.105926129 | 0.343863903 | 0.664564694 |  |  |  |  |  |  |  |  |
| Magee1 | 0.063410649 | 0.31963119 | 0.637503969 |  |  |  |  |  |  |  |  |
| Magi1 | -0.799198711 | 1.72297E-19 | 1.33422E-17 |  |  |  |  |  | SZdb |  | SZ_full |
| Magi2 | -0.139320696 | 0.08714674 | 0.301041568 | SYN |  |  |  | FMRP | SZdb |  | SZ_full |
| Magi3 | -0.433079146 | 1.12454E-05 | 0.000160453 |  |  |  |  |  | SZdb |  | SZ_full |
| Magt1 | -0.014266823 | 1 | 1 |  | ID |  |  |  |  |  |  |
| Mak16 | 0.061026397 | 0.796976892 | 0.933720314 |  |  |  |  |  |  |  |  |
| Mal | 0.598845556 | 0.033233859 | 0.15052428 |  |  |  |  |  |  |  |  |
| Mal2 | 0.110283914 | 0.105567793 | 0.342419161 |  |  |  |  |  |  |  |  |
| Malat1 | -0.150295192 | 0.381379264 | 0.695268093 |  |  |  |  |  |  |  |  |
| Malsu1 | -0.16896608 | 0.108949699 | 0.348848976 |  |  |  |  |  |  |  |  |
| Mamld1 | 0.291786542 | 0.01167547 | 0.066582117 |  |  |  |  |  |  |  |  |
| Man1a2 | -0.760912135 | 2.01854E-16 | 1.36439E-14 |  |  |  |  |  |  |  |  |
| Man1b1 | -0.020528817 | 0.780339501 | 0.926216702 |  |  |  |  |  |  |  |  |
| Man1c1 | -0.376676404 | 0.009803698 | 0.058331389 |  |  |  |  |  |  |  |  |
| Man2a1 | 0.267630066 | 0.172692962 | 0.461777959 |  |  |  |  |  |  | SZ_108 | SZ_full |
| Man2a2 | 0.105223133 | 0.274627696 | 0.592033284 |  |  |  |  | FMRP |  | SZ_108 | SZ_full |
| Man2b1 | -0.020438949 | 0.813310074 | 0.938538051 |  | ID |  |  |  |  |  |  |
| Man2b2 | 0.070854772 | 0.405462105 | 0.715162705 |  |  |  |  |  |  |  |  |
| Man2c1 | 0.116557175 | 0.314197497 | 0.63154306 |  |  |  |  |  |  |  |  |
| Manba | 0.040836326 | 0.888742116 | 0.962995126 |  | ID |  |  |  |  |  |  |
| Manea | 0.050459677 | 0.511333064 | 0.786598895 |  |  |  |  |  |  |  |  |
| Maneal | -0.077724592 | 0.506428944 | 0.784371068 |  |  |  |  |  |  |  |  |
| Manf | 0.43926196 | 0.325033156 | 0.642805468 |  |  |  |  |  |  |  |  |
| Maoa | 0.036325368 | 0.487126085 | 0.771519173 | SYN | ID | ASD | ASD_sc |  | SZdb |  | SZ_full |
| Map1a | 0.024827053 | 0.674276421 | 0.878031572 | SYN |  |  |  |  |  |  |  |
| Map1b | -0.059953551 | 0.585828533 | 0.827452483 | SYN |  |  |  |  |  |  |  |
| Map1lc3a | -0.04791789 | 0.356979584 | 0.675290951 | SYN |  |  |  |  |  |  |  |
| Map1lc3b | -0.026159286 | 0.704577374 | 0.889475963 |  |  |  |  |  |  |  |  |
| Map1s | 0.161710021 | 0.090751104 | 0.308520056 |  |  |  |  |  |  |  |  |
| Map2 | 0.172340885 | 0.027133602 | 0.128057755 | SYN |  | ASD |  |  |  |  |  |
| Map2k1 | 0.242699239 | 0.000693318 | 0.006475294 | SYN | ID |  |  |  |  |  |  |
| Map2k2 | 0.069582644 | 0.597197661 | 0.834737271 | SYN | ID |  |  |  |  |  |  |
| Map2k4 | 0.107809312 | 0.193621241 | 0.494183366 |  |  |  |  |  |  |  |  |
| Map2k5 | -0.080577025 | 0.29795183 | 0.614708689 |  |  |  |  |  |  |  |  |
| Map2k6 | 0.08812859 | 0.692782204 | 0.884375762 |  |  |  |  |  |  |  |  |
| Map2k7 | 0.04692772 | 0.91036799 | 0.972294468 | SYN |  |  |  |  |  |  |  |
| Map3k1 | -0.155345549 | 0.329724023 | 0.648232389 |  |  |  |  |  |  |  |  |
| Map3k10 | -0.131339726 | 0.067440544 | 0.252644122 |  |  |  |  |  |  |  |  |
| Map3k11 | 0.064681279 | 0.814239433 | 0.938538051 |  |  |  |  |  |  |  |  |
| Map3k12 | 0.036916333 | 0.73765311 | 0.908090941 | SYN |  |  |  | FMRP |  |  |  |
| Map3k2 | -0.020658225 | 0.817895493 | 0.94093963 |  |  |  |  |  |  |  |  |
| Map3k5 | -1.185744347 | 4.53594E-35 | 1.00496E-32 |  |  |  |  |  |  |  |  |
| Map3k7 | 0.020487339 | 0.756728785 | 0.916833457 |  |  |  |  |  |  |  |  |
| Map3k9 | -0.525441471 | 3.43226E-09 | 9.12523E-08 |  |  |  |  |  |  |  |  |
| Map4 | -0.276167021 | 0.031888737 | 0.145589334 | SYN |  |  |  |  |  |  |  |
| Map4k2 | -0.231572818 | 0.157550225 | 0.437389695 |  |  |  |  |  |  |  |  |
| Map4k3 | -0.035528154 | 0.740041037 | 0.909610197 |  |  |  |  |  |  |  |  |
| Map4k4 | 0.16425961 | 0.138941213 | 0.406826399 |  |  |  |  | FMRP |  |  |  |
| Map4k5 | 0.114124791 | 0.242412546 | 0.558164684 |  |  |  |  |  |  |  |  |
| Map6 | 0.050746971 | 0.678593838 | 0.878273493 | SYN |  |  |  |  | SZdb |  | SZ_full |
| Map6d1 | -0.334295031 | 0.000249932 | 0.002616085 | SYN |  |  |  |  |  |  |  |
| Map7 | -0.078864727 | 0.292996185 | 0.610185151 |  |  |  |  |  |  |  |  |
| Map7d1 | 0.102405111 | 0.245712119 | 0.563224472 | SYN |  |  |  |  |  |  |  |
| Map7d2 | -0.120693043 | 0.132923178 | 0.395788702 |  |  |  |  |  |  |  |  |
| Map9 | -0.100189572 | 0.296956883 | 0.613131788 |  |  |  |  |  |  |  |  |
| Mapk1 | 0.190240972 | 0.005677862 | 0.037396059 | SYN |  | ASD |  | FMRP |  |  |  |
| Mapk10 | -0.000453994 | 0.859960272 | 0.952644879 | SYN |  |  |  |  |  |  |  |
| Mapk11 | 0.054880363 | 0.834322847 | 0.943106438 |  |  |  |  |  |  |  |  |
| Mapk14 | -0.319866516 | 0.000104799 | 0.001200971 |  |  |  |  |  |  |  |  |
| Mapk1ip1 | -0.095642084 | 0.245302191 | 0.562706436 |  |  |  |  |  |  |  |  |
| Mapk1ip1l | 0.101447694 | 0.327068958 | 0.644601436 |  |  |  |  |  |  |  |  |
| Mapk3 | 0.412234124 | 0.016207501 | 0.086180687 | SYN |  | ASD | ASD_sc |  |  | SZ_108 | SZ_full |
| Mapk4 | -0.110965672 | 0.262822494 | 0.579626142 |  |  |  |  | FMRP |  |  |  |
| Mapk6 | -0.314533981 | 0.003524112 | 0.025391431 |  |  |  |  |  |  |  |  |
| Mapk7 | -0.004936825 | 0.852543571 | 0.9494683 |  |  |  |  |  |  |  |  |
| Mapk8 | -0.066782883 | 0.68516999 | 0.881864748 |  |  |  |  |  |  |  |  |
| Mapk8ip1 | 0.052443987 | 0.457513523 | 0.753640615 | SYN |  |  |  | FMRP |  |  |  |
| Mapk8ip2 | 0.095438628 | 0.226586984 | 0.53915805 |  |  | ASD |  |  | SZdb |  | SZ_full |
| Mapk8ip3 | 0.100270602 | 0.193476718 | 0.493972569 | SYN |  |  |  | FMRP |  |  |  |
| Mapk9 | -0.301151363 | 0.000486972 | 0.004730927 |  |  |  |  |  |  |  |  |
| Mapkapk2 | 0.038668123 | 0.643476523 | 0.861470791 |  |  |  |  |  |  |  |  |
| Mapkapk5 | 0.087712954 | 0.69500499 | 0.885237911 |  |  |  |  |  |  |  |  |
| Mapkbp1 | 0.064127922 | 0.633095928 | 0.85542489 |  |  |  |  | FMRP |  |  |  |
| Mapre2 | -0.08307349 | 0.446875722 | 0.744686785 | SYN |  |  |  |  |  |  |  |
| Mapre3 | 0.144249796 | 0.099267862 | 0.32866769 | SYN |  |  |  |  |  |  |  |
| Mapt | 0.016193924 | 0.906228957 | 0.970739161 | SYN |  |  |  |  |  |  |  |
| March1 | 0.328705472 | 0.003647537 | 0.02609216 |  |  |  |  |  |  |  |  |
| March2 | -0.692124402 | 1.1292E-10 | 3.6912E-09 |  |  |  |  |  |  |  |  |
| March5 | -0.133978112 | 0.205673855 | 0.511204322 |  |  |  |  |  |  |  |  |
| March6 | 0.045423143 | 0.394363125 | 0.705533207 |  |  |  |  |  |  |  |  |
| March7 | -0.005059648 | 0.966717567 | 0.990416798 |  |  |  |  |  |  |  |  |
| March8 | 0.006139973 | 0.975436456 | 0.99211875 |  |  |  |  |  |  |  |  |
| March9 | 0.161259065 | 0.122651912 | 0.377711061 |  |  |  |  |  |  |  |  |
| Marcks | -0.184266618 | 0.305303794 | 0.62282745 |  |  |  |  |  |  |  |  |
| Marcksl1 | -0.347696571 | 0.089892869 | 0.306273184 | SYN |  |  |  |  |  |  |  |
| Marf1 | -0.196396974 | 0.023713884 | 0.115966855 |  |  |  |  |  |  |  |  |
| Mark1 | -0.727187654 | 5.0606E-16 | 3.25511E-14 | SYN |  | ASD | ASD_sc |  |  |  |  |
| Mark2 | -0.083202134 | 0.231975351 | 0.545953202 | SYN |  |  |  |  |  |  |  |
| Mark3 | 0.044675408 | 0.719861302 | 0.897994027 |  |  |  |  |  |  |  |  |
| Mark4 | 0.124800425 | 0.218498247 | 0.527873129 |  |  |  |  |  |  |  |  |
| Mars | -0.137662379 | 0.224947926 | 0.537905582 | SYN |  |  |  |  |  |  |  |
| Masp1 | 0.098608319 | 0.521874257 | 0.791644937 |  |  |  |  |  |  |  |  |
| Mast1 | -0.045367758 | 0.419120373 | 0.726086902 |  |  |  |  | FMRP |  |  |  |
| Mast2 | -0.163951134 | 0.051868872 | 0.210751971 |  |  |  |  | FMRP |  |  |  |
| Mast3 | -0.388495599 | 7.45414E-06 | 0.000109897 |  |  |  |  |  |  |  |  |
| Mast4 | -0.37184678 | 0.000527615 | 0.005058002 |  |  |  |  | FMRP |  |  |  |
| Mat2a | 0.18490465 | 0.016978107 | 0.089502565 |  |  |  |  |  |  |  |  |
| Mat2b | -0.596085903 | 2.36378E-11 | 8.72849E-10 |  |  |  |  |  |  |  |  |
| Matk | 0.320023364 | 0.00538693 | 0.035745553 |  |  |  |  |  |  |  |  |
| Matn2 | 2.234258486 | 4.8032E-111 | 3.831E-107 |  |  |  |  |  |  |  |  |
| Matn4 | -0.285643239 | 0.058387076 | 0.227825652 |  |  |  |  |  |  |  |  |
| Matr3 | -0.036425449 | 0.970297875 | 0.991454046 |  |  |  |  |  |  |  |  |
| Mau2 | 0.05670907 | 0.607547796 | 0.841251463 |  |  |  |  |  |  | SZ_108 | SZ_full |
| Max | -0.084874982 | 0.321729394 | 0.638972522 |  |  |  |  |  |  |  |  |
| Maz | 0.126380585 | 0.159271594 | 0.440940726 |  |  |  |  | FMRP |  |  |  |
| Mb21d2 | -1.019636647 | 2.96155E-21 | 2.41034E-19 |  |  |  |  |  |  |  |  |
| Mbd1 | 0.029875536 | 0.9487994 | 0.986912479 |  |  | ASD | ASD_sc |  |  |  |  |
| Mbd2 | -0.040956831 | 0.779154485 | 0.925608604 |  |  |  |  |  |  |  |  |
| Mbd3 | 0.092489967 | 0.251737931 | 0.569444623 |  |  | ASD | ASD_sc |  |  |  |  |
| Mbd4 | -0.121465919 | 0.287187665 | 0.605020818 |  |  | ASD | ASD_sc |  |  |  |  |
| Mbd5 | -0.088852706 | 0.320602633 | 0.637688241 |  |  | ASD | ASD_sc | FMRP |  |  |  |
| Mbd6 | 0.189843762 | 0.181209468 | 0.473877612 |  |  | ASD |  |  |  |  |  |
| Mbip | -0.09642891 | 0.276532655 | 0.59394256 |  |  |  |  |  |  |  |  |
| Mblac2 | 0.118288146 | 0.295212745 | 0.611588794 |  |  |  |  |  |  |  |  |
| Mbnl1 | -0.036120483 | 0.849013586 | 0.947758203 |  |  |  |  |  |  |  |  |
| Mbnl2 | 0.120757147 | 0.10963683 | 0.350626846 |  |  |  |  |  |  |  |  |
| Mboat2 | -0.194388956 | 0.040580684 | 0.17619572 |  |  |  |  |  |  |  |  |
| Mboat7 | -0.040168222 | 0.916845162 | 0.97516429 |  |  |  |  |  |  |  |  |
| Mbp | 0.430164871 | 0.050203467 | 0.206509979 | SYN |  |  |  | FMRP |  |  |  |
| Mbtd1 | -0.258875141 | 0.00822967 | 0.050844188 |  |  |  |  |  |  |  |  |
| Mbtps1 | -0.076342901 | 0.453666996 | 0.750292953 |  |  |  |  |  |  |  |  |
| Mbtps2 | -0.083785474 | 0.392595461 | 0.703988624 |  | ID |  |  |  |  |  |  |
| Mcat | -0.142534219 | 0.350213413 | 0.669369249 |  |  |  |  |  |  |  |  |
| Mcc | -0.313860795 | 0.001303445 | 0.011239219 |  |  | ASD |  |  |  |  |  |
| Mccc1 | -0.048737078 | 0.320840384 | 0.637688241 | SYN | ID |  |  |  |  |  |  |
| Mccc2 | -0.220545378 | 0.083109023 | 0.291631132 | SYN | ID |  |  |  |  |  |  |
| Mcee | 0.078997949 | 0.969165493 | 0.991454046 |  |  |  |  |  |  |  |  |
| Mcf2l | 0.005843368 | 0.957062696 | 0.989355248 |  |  |  |  |  |  |  |  |
| Mcfd2 | -0.054327792 | 0.710314719 | 0.893043852 |  |  |  |  |  |  |  |  |
| Mcl1 | 0.047359735 | 0.349012857 | 0.668361715 |  |  |  |  |  |  |  |  |
| Mcm3ap | -0.016320758 | 0.678698985 | 0.878273493 |  |  |  |  |  |  |  |  |
| Mcm7 | 0.126321973 | 0.360192012 | 0.677462343 |  |  |  |  |  |  |  |  |
| Mcmbp | 0.057143053 | 0.7535126 | 0.915220708 |  |  |  |  |  |  |  |  |
| Mcoln1 | -0.045314526 | 0.612091904 | 0.844056326 |  | ID |  |  |  |  |  |  |
| Mcrs1 | -0.064633486 | 0.538243912 | 0.801453443 |  |  |  |  |  |  |  |  |
| Mctp1 | 0.55253961 | 4.25719E-09 | 1.11329E-07 |  |  |  |  |  |  |  |  |
| Mcts1 | 0.020410262 | 0.960579677 | 0.989984854 |  |  |  |  |  |  |  |  |
| Mcu | -0.164292652 | 0.141515923 | 0.411045522 |  |  |  |  |  |  |  |  |
| Mdc1 | -0.152685063 | 0.220031034 | 0.530361901 |  |  |  |  |  |  |  |  |
| Mdga1 | 2.751607619 | 2.28277E-38 | 6.06912E-36 |  |  |  |  |  |  |  |  |
| Mdga2 | -0.161091736 | 0.080631306 | 0.285701568 |  |  | ASD | ASD_sc |  |  |  |  |
| Mdh1 | -0.198464396 | 0.018287684 | 0.094777496 | SYN |  |  |  |  |  |  |  |
| Mdh2 | 0.087089981 | 0.288298311 | 0.606559569 | SYN |  |  |  |  |  |  |  |
| Mdm1 | 0.293042407 | 0.050242308 | 0.20651797 |  |  |  |  |  |  |  |  |
| Mdm2 | 0.144446595 | 0.26268992 | 0.579626142 |  |  |  |  |  |  |  |  |
| Mdn1 | 0.026608368 | 1 | 1 |  |  |  |  |  |  |  |  |
| Mdp1 | 0.13610456 | 0.300090853 | 0.617046828 |  |  |  |  |  |  |  |  |
| Me1 | 0.660436739 | 7.5921E-13 | 3.20394E-11 |  |  |  |  |  |  |  |  |
| Me3 | 0.534515861 | 5.57381E-09 | 1.43409E-07 | SYN |  |  |  |  |  |  |  |
| Meaf6 | 0.086050213 | 0.392285982 | 0.703908435 |  |  |  |  |  |  |  |  |
| Mecp2 | 0.087407797 | 0.278783959 | 0.596134277 |  | ID | ASD | ASD_sc |  |  |  |  |
| Med1 | 0.147714757 | 0.115962854 | 0.363140842 |  |  |  |  |  |  |  |  |
| Med12 | -0.011584883 | 0.8087235 | 0.93712146 |  | ID | ASD |  |  | SZdb |  | SZ_full |
| Med12l | -0.043794281 | 0.587432624 | 0.828352242 |  |  |  |  |  |  |  |  |
| Med13 | 0.023831398 | 0.526064732 | 0.793773756 |  |  |  |  | FMRP |  |  |  |
| Med13l | -0.291618865 | 0.001912976 | 0.015601119 |  |  | ASD | ASD_sc | FMRP |  |  |  |
| Med14 | 0.059827992 | 0.279393813 | 0.597118181 |  |  |  |  | FMRP |  |  |  |
| Med15 | 0.080923221 | 0.458606477 | 0.754418091 |  |  |  |  |  | SZdb |  | SZ_full |
| Med17 | 0.063836745 | 0.470402326 | 0.762867484 |  | ID |  |  |  |  |  |  |
| Med22 | 0.052017378 | 0.499003151 | 0.779027036 |  |  |  |  |  |  |  |  |
| Med23 | 0.036234612 | 0.677834709 | 0.878273493 |  | ID |  |  |  |  |  |  |
| Med24 | 0.165409227 | 0.037820069 | 0.166567017 |  |  |  |  |  |  |  |  |
| Med25 | -0.047784573 | 0.692634427 | 0.884375762 |  |  |  |  |  |  |  |  |
| Med27 | 0.370647268 | 0.002996918 | 0.022340306 |  |  |  |  |  |  |  |  |
| Med28 | -0.197734175 | 0.083554222 | 0.292421446 |  |  |  |  |  |  |  |  |
| Med4 | -0.049209859 | 0.597541109 | 0.834762978 |  |  |  |  |  |  |  |  |
| Med6 | 0.003997836 | 0.888368389 | 0.962995126 |  |  |  |  |  |  |  |  |
| Med9 | -0.142041558 | 0.230922026 | 0.544855243 |  |  |  |  |  |  |  |  |
| Mef2a | 0.15247317 | 0.087611118 | 0.301965898 |  |  |  |  |  |  |  |  |
| Mef2c | 0.737601711 | 7.4295E-14 | 3.65788E-12 |  |  | ASD | ASD_sc |  |  | SZ_108 | SZ_full |
| Mef2d | 0.347439312 | 0.000132377 | 0.001476702 |  |  |  |  | FMRP |  |  |  |
| Meg3 | 0.153311245 | 0.580803809 | 0.825020691 |  |  |  |  |  |  |  |  |
| Megf11 | 0.135938859 | 0.387847205 | 0.699405224 |  |  |  |  |  |  |  |  |
| Megf8 | -0.092081766 | 0.298629016 | 0.615445356 |  |  |  |  |  |  |  |  |
| Megf9 | -0.345665107 | 0.000671242 | 0.006306035 |  |  |  |  |  |  |  |  |
| Meis2 | -0.16711784 | 0.658390571 | 0.869835247 |  |  |  |  |  |  |  |  |
| Meis3 | 0.327821259 | 0.002831043 | 0.021382954 |  |  |  |  |  |  |  |  |
| Memo1 | -0.129828197 | 0.130026251 | 0.39017659 |  |  |  |  |  |  |  |  |
| Men1 | 0.027787352 | 0.572251174 | 0.820827732 |  |  |  |  |  |  |  |  |
| Mepce | 0.018786988 | 0.966054419 | 0.990416798 |  |  |  |  |  |  |  |  |
| Mertk | -0.270635286 | 0.008571013 | 0.052304822 |  |  |  |  |  |  |  |  |
| Mesdc1 | -0.364947471 | 0.003089081 | 0.02283458 |  |  |  |  |  |  |  |  |
| Mesdc2 | -0.047735162 | 0.774793102 | 0.923729414 |  |  |  |  |  |  |  |  |
| Mest | -0.201292049 | 0.21741419 | 0.526121233 |  |  |  |  |  |  |  |  |
| Metap1 | 0.145074012 | 0.107927957 | 0.347110235 |  |  |  |  |  |  |  |  |
| Metap1d | 0.155259949 | 0.148043107 | 0.422465767 |  |  |  |  |  |  |  |  |
| Metap2 | -0.014220229 | 0.806290139 | 0.936945007 |  |  |  |  |  |  |  |  |
| Metrn | -0.057688579 | 0.560201674 | 0.813429555 |  |  |  |  |  |  |  |  |
| Mettl10 | -0.096584354 | 0.510231325 | 0.786093307 |  |  |  |  |  |  |  |  |
| Mettl17 | 0.079168682 | 0.733814384 | 0.905992378 |  |  |  |  |  |  |  |  |
| Mettl2 | -0.071343403 | 0.339349575 | 0.660457575 |  |  |  |  |  |  |  |  |
| Mettl23 | -0.059220075 | 0.57216624 | 0.820827732 |  |  |  |  |  |  |  |  |
| Mettl3 | 0.148290951 | 0.303606155 | 0.621431579 |  |  |  |  |  |  |  |  |
| Mettl7a1 | 0.013929796 | 0.793126344 | 0.932484628 |  |  |  |  |  |  |  |  |
| Mettl8 | -0.099673741 | 0.387742528 | 0.699405224 |  |  |  |  |  |  |  |  |
| Mettl9 | -0.138007296 | 0.243884912 | 0.560127769 |  |  |  |  |  |  |  |  |
| Mex3c | -0.04030473 | 0.766715238 | 0.920286747 |  |  |  |  |  |  |  |  |
| Mex3d | 0.00689803 | 0.9965369 | 1 |  |  |  |  |  |  |  |  |
| Mfap1a | 0.160042685 | 0.056710189 | 0.22347849 |  |  |  |  |  |  |  |  |
| Mfap3 | -0.441545402 | 0.001666729 | 0.01389755 |  |  |  |  |  |  |  |  |
| Mfap3l | -0.227820426 | 0.011521336 | 0.065968542 |  |  |  |  |  |  |  |  |
| Mff | -0.029005431 | 0.995003726 | 1 | SYN |  |  |  |  |  |  |  |
| Mfge8 | -0.392413304 | 1.4608E-05 | 0.00020193 |  |  |  |  |  |  |  |  |
| Mfhas1 | -0.000796598 | 0.848147003 | 0.947466244 |  |  |  |  | FMRP |  |  |  |
| Mfn1 | -0.175840186 | 0.014308936 | 0.078116409 |  |  |  |  |  |  |  |  |
| Mfn2 | -0.071428817 | 0.531873901 | 0.797261085 | SYN |  |  |  |  |  |  |  |
| Mfsd1 | 0.065371472 | 0.64312825 | 0.861247636 |  |  |  |  |  |  |  |  |
| Mfsd11 | 0.082120992 | 0.617269783 | 0.847391358 |  |  |  |  |  |  |  |  |
| Mfsd12 | 0.142289001 | 0.30768668 | 0.624820652 |  |  |  |  |  |  |  |  |
| Mfsd4 | -0.273606506 | 0.001176181 | 0.010251298 |  |  |  |  |  |  |  |  |
| Mfsd5 | 0.032347809 | 0.681708923 | 0.879966074 |  |  |  |  |  |  |  |  |
| Mfsd6 | -0.170490428 | 0.10694265 | 0.345613686 |  |  |  |  |  |  |  |  |
| Mfsd7b | -0.019500517 | 0.607848646 | 0.841251463 |  |  |  |  |  |  |  |  |
| Mfsd8 | 0.186082926 | 0.092503656 | 0.313028918 |  |  |  |  |  |  |  |  |
| Mga | 0.090641004 | 0.396974224 | 0.707387492 |  |  |  |  |  |  |  |  |
| Mgat1 | -0.179093414 | 0.058400667 | 0.227825652 |  |  |  |  |  |  |  |  |
| Mgat2 | 0.130667816 | 0.184396217 | 0.479382082 |  | ID |  |  |  |  |  |  |
| Mgat3 | -0.036250135 | 0.879191952 | 0.959555967 |  |  |  |  |  |  |  |  |
| Mgat4a | 0.016391476 | 0.736572695 | 0.907461202 |  |  |  |  |  |  |  |  |
| Mgat4c | 0.140954011 | 0.526695622 | 0.794238803 |  |  |  |  |  |  |  |  |
| Mgat5 | 0.183776813 | 0.032074967 | 0.146188537 |  |  |  |  |  |  |  |  |
| Mgat5b | -0.751293673 | 1.53296E-08 | 3.72772E-07 |  |  |  |  | FMRP |  |  |  |
| Mgea5 | -0.270203261 | 0.003077436 | 0.022769597 |  |  |  |  |  |  |  |  |
| Mgll | 0.002210288 | 0.83305742 | 0.943106438 |  |  |  |  |  |  |  |  |
| Mgrn1 | -0.022869064 | 0.767609971 | 0.920286747 |  |  |  |  |  |  |  |  |
| Mgst3 | -0.187465279 | 0.088886814 | 0.303783059 | SYN |  |  |  |  |  |  |  |
| Miat | -0.063827955 | 0.371235943 | 0.687001829 |  |  |  |  |  |  |  |  |
| Mib1 | -0.105658779 | 0.310403327 | 0.627438665 |  |  | ASD | ASD_sc | FMRP |  |  |  |
| Mib2 | 0.075324088 | 0.591031398 | 0.830403111 |  |  |  |  |  |  |  |  |
| Mical2 | -0.056613769 | 0.554116419 | 0.808611129 |  |  |  |  | FMRP |  |  |  |
| Mical3 | -0.730637264 | 1.16352E-06 | 2.04411E-05 |  |  |  |  |  |  |  |  |
| Micall1 | 0.126985958 | 0.379384547 | 0.693775541 |  |  |  |  |  |  |  |  |
| Micu1 | -0.012463527 | 0.83080664 | 0.942471023 |  |  |  |  |  |  |  |  |
| Mid1ip1 | -0.018488717 | 0.991936467 | 0.998950159 |  |  |  |  |  |  |  |  |
| Midn | 0.233367461 | 0.234231447 | 0.548217089 |  |  |  |  |  |  |  |  |
| Mien1 | -0.123148555 | 0.284618345 | 0.602965919 |  |  |  |  |  |  |  |  |
| Mier1 | -0.050164452 | 0.767998437 | 0.920305819 |  |  |  |  |  |  |  |  |
| Mif | 0.264400585 | 0.183685933 | 0.47831505 | SYN |  |  |  |  |  |  |  |
| Mif4gd | 0.007884979 | 0.782672213 | 0.927577054 |  |  |  |  |  |  |  |  |
| Miip | 0.091443349 | 0.577754731 | 0.823155759 |  |  |  |  |  |  |  |  |
| Mink1 | 0.010297546 | 0.719194944 | 0.897994027 | SYN |  |  |  | FMRP |  |  |  |
| Minos1 | -0.055236028 | 0.485630327 | 0.77082338 |  |  |  |  |  |  |  |  |
| Minpp1 | -0.039405235 | 0.895230691 | 0.965827133 |  |  |  |  |  |  |  |  |
| Mios | 0.075309268 | 0.223084844 | 0.535135253 |  |  |  |  |  |  |  |  |
| Mir101a | -0.045255889 | 0.545133303 | 0.805331215 |  |  |  |  |  |  |  |  |
| Mir125b-1 | -0.405309144 | 0.198167268 | 0.500025982 |  |  |  |  |  |  |  |  |
| Mir132 | 0.50673142 | 0.368931596 | 0.685281419 |  |  |  |  |  |  |  |  |
| Mir22hg | 0.064905871 | 0.542752252 | 0.803723472 |  |  |  |  |  |  |  |  |
| Mir3078 | 0.109946098 | 0.548100839 | 0.806991235 |  |  |  |  |  |  |  |  |
| Mir3093 | -0.112051069 | 0.169448489 | 0.457522392 |  |  |  |  |  |  |  |  |
| Mir9-3 | -0.26480787 | 0.002664635 | 0.020396476 |  |  |  |  |  |  |  |  |
| Mirg | 0.227989515 | 0.304765403 | 0.622326896 |  |  |  |  |  |  |  |  |
| Mki67ip | 0.087013584 | 0.483393397 | 0.76869627 |  |  |  |  |  |  |  |  |
| Mkl1 | 0.210775939 | 0.036891168 | 0.16319687 |  |  |  |  |  |  |  |  |
| Mkl2 | -0.096683111 | 0.275664873 | 0.593281983 | SYN |  | ASD | ASD_sc | FMRP |  |  |  |
| Mkln1 | 0.16761651 | 0.052286774 | 0.21191022 |  |  |  |  |  |  |  |  |
| Mknk2 | 0.154536106 | 0.20546671 | 0.511039097 |  |  |  |  |  |  |  |  |
| Mkrn1 | -0.007218972 | 0.979470895 | 0.993945956 |  |  |  |  |  |  |  |  |
| Mkrn2 | -0.071213163 | 0.667135147 | 0.875092018 |  |  |  |  |  |  |  |  |
| Mkx | 0.034398788 | 0.800741099 | 0.935166555 |  |  |  |  |  |  |  |  |
| Mlc1 | -0.011982865 | 0.80849752 | 0.93712146 |  |  |  |  |  | SZdb |  | SZ_full |
| Mlec | -0.101144893 | 0.27100192 | 0.589288799 |  |  |  |  |  |  |  |  |
| Mlf2 | 0.122703996 | 0.149155181 | 0.423970677 | SYN |  |  |  |  |  |  |  |
| Mlh1 | -0.160724998 | 0.103312947 | 0.337024158 |  |  |  |  |  |  |  |  |
| Mlip | -0.458950512 | 0.000139949 | 0.001555862 |  |  |  |  |  |  |  |  |
| Mll1 | -0.001167257 | 0.995284581 | 1 |  |  |  |  | FMRP |  |  |  |
| Mll2 | -0.017202813 | 0.7500727 | 0.914068732 |  |  |  |  | FMRP |  |  |  |
| Mll3 | 0.122312541 | 0.179386778 | 0.471297716 |  |  |  |  | FMRP |  |  |  |
| Mll5 | 0.013541634 | 0.906174519 | 0.970739161 |  |  |  |  | FMRP |  |  |  |
| Mllt10 | -0.107462135 | 0.247556386 | 0.564495266 |  |  |  |  |  |  |  |  |
| Mllt11 | 0.303940759 | 0.000512114 | 0.004939081 |  |  |  |  |  |  |  |  |
| Mllt3 | -0.015350627 | 0.877912313 | 0.959555967 |  |  |  |  |  |  |  |  |
| Mllt4 | 0.061800939 | 0.516617254 | 0.789224137 | SYN |  |  |  |  |  |  |  |
| Mllt6 | -0.014453988 | 0.805700441 | 0.936945007 |  |  |  |  |  |  |  |  |
| Mlst8 | -0.054934696 | 0.572870147 | 0.820827732 |  |  |  |  |  |  |  |  |
| Mlx | 0.155140123 | 0.233427662 | 0.548048187 |  |  |  |  |  |  |  |  |
| Mlycd | 0.032672788 | 0.775827073 | 0.924077297 |  | ID |  |  |  |  |  |  |
| Mmaa | -0.167628343 | 0.024357823 | 0.118462191 |  |  |  |  |  |  |  |  |
| Mmab | 0.056278265 | 0.394518318 | 0.705533207 |  |  |  |  |  |  |  |  |
| Mmachc | -0.131132365 | 0.505500274 | 0.783648238 |  |  |  |  |  |  |  |  |
| Mmadhc | 0.042288591 | 0.556496345 | 0.810114044 |  |  |  |  |  |  |  |  |
| Mmd | 0.344177939 | 5.04608E-06 | 7.69552E-05 |  |  |  |  |  |  |  |  |
| Mmd2 | 0.155012633 | 0.105556037 | 0.342419161 |  |  |  |  |  |  |  |  |
| Mmgt2 | 0.071933656 | 0.678508933 | 0.878273493 |  |  |  |  |  |  |  |  |
| Mmp16 | 0.275193213 | 0.011503004 | 0.065910891 |  |  |  |  |  |  | SZ_108 | SZ_full |
| Mmp17 | 0.477719409 | 3.02647E-08 | 7.03765E-07 |  |  |  |  |  |  |  |  |
| Mmp24 | 0.20876949 | 0.095676772 | 0.32023413 |  |  |  |  | FMRP |  |  |  |
| Mms19 | 0.154275 | 0.170148415 | 0.458736914 |  |  |  |  |  |  |  |  |
| Mn1 | 0.526386147 | 4.10711E-09 | 1.07758E-07 |  |  |  |  |  |  |  |  |
| Mnat1 | -0.092642752 | 0.3483038 | 0.667863914 |  |  |  |  |  |  |  |  |
| Mnf1 | -0.045203861 | 0.667839156 | 0.875092018 |  |  |  |  |  |  |  |  |
| Mnt | 0.314458174 | 0.001780079 | 0.014697629 |  |  |  |  |  |  |  |  |
| Mob1b | -0.069786516 | 0.690419178 | 0.884076979 |  |  |  |  |  |  |  |  |
| Mob3a | 0.053941503 | 0.699864701 | 0.887191673 |  |  |  |  |  |  |  |  |
| Mobp | 0.615791747 | 0.011686931 | 0.066582117 |  |  |  |  |  |  |  |  |
| Mocs2 | -0.057448515 | 0.512737321 | 0.787509131 |  | ID |  |  |  |  |  |  |
| Mog | 0.537531819 | 0.077372941 | 0.276986792 | SYN |  |  |  |  | SZdb |  | SZ_full |
| Mon1a | 0.024186008 | 0.891553262 | 0.964133804 |  |  |  |  |  |  |  |  |
| Mon2 | 0.088861599 | 0.243511231 | 0.55972495 |  |  |  |  | FMRP |  |  |  |
| Morc2a | -0.127651964 | 0.174960067 | 0.464227433 |  |  |  |  |  |  |  |  |
| Morc3 | 0.050828046 | 0.560649649 | 0.813834731 |  |  |  |  |  |  |  |  |
| Morc4 | 0.091210674 | 0.703313542 | 0.888798685 |  |  |  |  |  |  |  |  |
| Morf4l1 | -0.046696532 | 0.97239188 | 0.991571804 |  |  |  |  |  |  |  |  |
| Morf4l2 | 0.117683128 | 0.147162793 | 0.421153516 |  |  |  |  |  |  |  |  |
| Morn1 | -0.150370954 | 0.123814541 | 0.379824915 |  |  |  |  |  |  |  |  |
| Morn4 | 0.081229343 | 0.373261664 | 0.688992138 |  |  |  |  |  |  |  |  |
| Mospd2 | 0.142614554 | 0.153721398 | 0.430204164 |  |  |  |  |  |  |  |  |
| Mospd3 | 0.199010208 | 0.245907534 | 0.563224472 |  |  |  |  |  |  |  |  |
| Mov10 | 0.16066259 | 0.691587181 | 0.884120561 |  |  |  |  |  |  |  |  |
| Mpc1 | -0.006435312 | 0.563305563 | 0.815709 |  |  |  |  |  |  |  |  |
| Mpc2 | -0.03216284 | 0.58285257 | 0.826018497 |  |  |  |  |  |  |  |  |
| Mpdu1 | 0.17289174 | 0.301509895 | 0.619485554 |  | ID |  |  |  |  |  |  |
| Mpdz | -0.268633504 | 0.049440384 | 0.204743771 |  |  |  |  |  |  |  |  |
| Mphosph10 | 0.266038834 | 0.021067338 | 0.105947723 |  |  |  |  |  |  |  |  |
| Mphosph8 | 0.037103689 | 0.869945839 | 0.956485207 |  |  |  |  |  |  |  |  |
| Mphosph9 | -0.057978853 | 0.277575653 | 0.594347225 |  |  |  |  |  |  | SZ_108 | SZ_full |
| Mpi | -0.003885385 | 0.846838797 | 0.947043961 |  |  |  |  |  |  |  |  |
| Mpnd | 0.190022006 | 0.107366545 | 0.346052663 |  |  |  |  |  |  |  |  |
| Mpp1 | 0.088672308 | 0.350546058 | 0.669369249 | SYN |  |  |  |  |  |  |  |
| Mpp2 | 0.047976895 | 0.492666108 | 0.775509152 | SYN |  |  |  |  |  |  |  |
| Mpp3 | -0.892295677 | 3.95881E-15 | 2.22363E-13 | SYN | ID |  |  |  |  |  |  |
| Mpp5 | -0.073347502 | 0.60357486 | 0.83854957 |  |  |  |  |  |  |  |  |
| Mpp6 | 0.015503763 | 0.645375753 | 0.862572203 | SYN |  |  |  |  |  | SZ_108 | SZ_full |
| Mpp7 | -0.098533952 | 0.437433831 | 0.737692921 | SYN |  |  |  |  |  |  |  |
| Mppe1 | -0.05915069 | 0.55199276 | 0.807970335 |  |  |  |  |  |  |  |  |
| Mpped1 | 0.29529596 | 8.80368E-05 | 0.001028084 |  |  |  |  |  |  |  |  |
| Mpped2 | -0.08364053 | 0.547231303 | 0.806637752 |  |  |  |  |  |  |  |  |
| Mprip | -0.120583172 | 0.137468873 | 0.404858426 | SYN |  |  |  | FMRP |  |  |  |
| Mpst | 0.077188907 | 0.886115926 | 0.962577588 |  |  |  |  |  |  |  |  |
| Mpv17 | 0.031413861 | 0.682434809 | 0.880618029 |  |  |  |  |  |  |  |  |
| Mpv17l | -0.021305294 | 0.83099356 | 0.942549009 |  |  |  |  |  |  |  |  |
| Mpv17l2 | 0.134613663 | 0.3720281 | 0.687670017 |  |  |  |  |  |  |  |  |
| Mpzl1 | 0.20621513 | 0.035471394 | 0.15849851 |  |  |  |  |  | SZdb |  | SZ_full |
| Mras | 0.116135698 | 0.11866468 | 0.367846671 | SYN |  |  |  |  |  |  |  |
| Mre11a | -0.184644172 | 0.292422328 | 0.609448781 |  |  |  |  |  |  |  |  |
| Mrfap1 | 0.123724255 | 0.112225614 | 0.356334194 |  |  |  |  |  |  |  |  |
| Mrgprx2 | 0.253116961 | 0.132775475 | 0.395598502 |  |  |  |  |  |  |  |  |
| Mro | 0.161705976 | 0.276915123 | 0.59394256 |  |  |  |  |  |  |  |  |
| Mroh1 | -0.056537125 | 0.396124641 | 0.706474453 |  |  |  |  |  |  |  |  |
| Mrpl1 | -0.087672513 | 0.578669107 | 0.823895893 |  |  |  |  |  |  |  |  |
| Mrpl10 | 0.157535831 | 0.153785492 | 0.43023258 |  |  |  |  |  |  |  |  |
| Mrpl12 | 0.181469993 | 0.147753798 | 0.421942103 |  |  |  |  |  |  |  |  |
| Mrpl13 | 0.21848588 | 0.156574513 | 0.435286969 |  |  |  |  |  |  |  |  |
| Mrpl14 | -0.00515619 | 0.520287131 | 0.791042729 |  |  |  |  |  |  |  |  |
| Mrpl15 | -0.0244594 | 0.856461678 | 0.951412026 |  |  |  |  |  |  |  |  |
| Mrpl17 | 0.179300552 | 0.273370116 | 0.591112429 |  |  |  |  |  |  |  |  |
| Mrpl19 | 0.0919233 | 0.483519206 | 0.76869627 |  |  |  |  |  |  |  |  |
| Mrpl20 | 0.017638265 | 0.797266762 | 0.933720314 |  |  |  |  |  |  |  |  |
| Mrpl22 | -0.132887016 | 0.315274887 | 0.632452841 |  |  |  |  |  |  |  |  |
| Mrpl28 | 0.058194173 | 0.832188361 | 0.943106438 |  |  |  |  |  |  |  |  |
| Mrpl3 | 0.090155348 | 0.267791512 | 0.5855003 |  |  |  |  |  |  |  |  |
| Mrpl32 | 0.236108194 | 0.170185874 | 0.458736914 |  |  |  |  |  |  |  |  |
| Mrpl35 | -0.0192474 | 0.972374869 | 0.991571804 |  |  |  |  |  |  |  |  |
| Mrpl37 | -0.005912053 | 0.800352453 | 0.935166555 |  |  |  |  |  |  |  |  |
| Mrpl38 | 0.343863908 | 0.00693961 | 0.044355774 |  |  |  |  |  |  |  |  |
| Mrpl4 | 0.230381323 | 0.029584278 | 0.137508274 |  |  |  |  |  |  |  |  |
| Mrpl41 | 0.177466934 | 0.623197356 | 0.850114949 |  |  |  |  |  |  |  |  |
| Mrpl43 | -0.086428852 | 0.41290012 | 0.721580052 |  |  |  |  |  |  |  |  |
| Mrpl9 | 0.024113795 | 0.886203772 | 0.962577588 |  |  |  |  |  |  |  |  |
| Mrps10 | 0.002008625 | 0.936229465 | 0.98530815 |  |  |  |  |  |  |  |  |
| Mrps11 | 0.220794511 | 0.161292446 | 0.44367579 |  |  |  |  |  |  |  |  |
| Mrps14 | 0.048839408 | 0.878910513 | 0.959555967 |  |  |  |  |  |  |  |  |
| Mrps15 | 0.009733689 | 0.840985133 | 0.945012317 |  |  |  |  |  |  |  |  |
| Mrps18a | 0.07555881 | 0.871075041 | 0.956962054 |  |  |  |  |  |  |  |  |
| Mrps2 | 0.072476668 | 0.618373244 | 0.847941692 |  |  |  |  |  |  |  |  |
| Mrps24 | 0.09055282 | 0.844606054 | 0.945963967 |  |  |  |  |  |  |  |  |
| Mrps25 | 0.120321688 | 0.205149836 | 0.51053825 |  |  |  |  |  |  |  |  |
| Mrps27 | 0.20748757 | 0.308260775 | 0.625257997 |  |  |  |  |  |  |  |  |
| Mrps30 | 0.125327591 | 0.285663397 | 0.603881429 |  |  |  |  |  |  |  |  |
| Mrps31 | 0.053214285 | 0.812590442 | 0.938355489 |  |  |  |  |  |  |  |  |
| Mrps34 | 0.0431992 | 0.853575843 | 0.950119195 |  |  |  |  |  |  |  |  |
| Mrps5 | 0.049464115 | 0.770380825 | 0.921167131 |  |  |  |  |  |  |  |  |
| Mrps6 | 0.28549404 | 0.070747059 | 0.260276079 |  |  |  |  |  |  |  |  |
| Mrps9 | 0.032300241 | 0.963920214 | 0.990416798 |  |  |  |  |  |  |  |  |
| Mrs2 | -0.099218037 | 0.38369344 | 0.697684359 |  |  |  |  |  |  |  |  |
| Mrto4 | 0.148503 | 0.314036381 | 0.63143328 |  |  |  |  |  |  |  |  |
| Msh2 | -0.106484471 | 0.304401991 | 0.622062588 |  |  |  |  |  |  |  |  |
| Msh3 | -0.138063354 | 0.236976232 | 0.551724415 |  |  |  |  |  |  |  |  |
| Msh6 | -0.019491568 | 0.929531591 | 0.981434219 |  |  |  |  |  |  |  |  |
| Msi2 | -0.190368702 | 0.022210023 | 0.110281768 |  |  |  |  |  |  |  |  |
| Msl1 | -0.046364932 | 0.557183263 | 0.810374491 |  |  |  |  |  |  |  |  |
| Msl2 | -0.056729243 | 0.762277866 | 0.91966847 |  |  |  |  |  |  | SZ_108 | SZ_full |
| Msra | 0.411103106 | 0.001443304 | 0.012312077 |  |  |  |  |  |  |  |  |
| Msrb2 | 0.29015805 | 0.021914035 | 0.109378189 |  |  |  |  |  |  |  |  |
| Msrb3 | -0.319122746 | 0.001648297 | 0.013801681 |  |  |  |  |  |  |  |  |
| Mss51 | -0.047541375 | 0.62384035 | 0.85035801 |  |  |  |  |  |  |  |  |
| Mt1 | 0.206452944 | 0.579328633 | 0.824099372 |  |  |  |  |  |  |  |  |
| Mt2 | 0.23837179 | 0.552304606 | 0.807970335 |  |  |  |  |  |  |  |  |
| Mt3 | 0.075080421 | 0.951289091 | 0.988210704 |  |  |  |  |  |  |  |  |
| Mta1 | -0.085840152 | 0.322545031 | 0.639795864 |  |  |  |  |  |  |  |  |
| Mta2 | 0.16117429 | 0.204135005 | 0.509283954 |  |  |  |  |  |  |  |  |
| Mta3 | -0.095835608 | 0.271985617 | 0.590140718 |  |  |  |  |  |  |  |  |
| Mtch1 | -0.199057671 | 0.0172996 | 0.090717692 | SYN | ID |  |  |  |  |  |  |
| Mtch2 | -0.091898476 | 0.295118408 | 0.611588794 | SYN |  |  |  |  |  |  |  |
| Mtcp1 | 0.124454517 | 0.658416858 | 0.869835247 |  |  |  |  |  |  |  |  |
| Mtdh | 0.103896411 | 0.265288393 | 0.581621832 | SYN |  |  |  |  |  |  |  |
| Mterfd1 | -0.043435455 | 0.839886813 | 0.944846439 |  |  |  |  |  |  |  |  |
| Mtf1 | 0.160027024 | 0.09967381 | 0.329737995 |  |  | ASD | ASD_sc |  |  |  |  |
| Mtf2 | 0.061397522 | 0.655886884 | 0.86959601 |  |  |  |  |  |  |  |  |
| Mtfp1 | 0.357952416 | 0.011904967 | 0.067631065 |  |  |  |  |  |  |  |  |
| Mtfr1l | -0.018559075 | 0.742438372 | 0.910888856 |  |  |  |  |  |  |  |  |
| Mthfd1 | 0.023347332 | 0.690435745 | 0.884076979 | SYN |  |  |  |  | SZdb |  | SZ_full |
| Mthfr | 0.103471121 | 0.423512019 | 0.729913577 |  | ID | ASD | ASD_sc |  | SZdb |  | SZ_full |
| Mtif2 | 0.030638121 | 0.966094677 | 0.990416798 |  |  |  |  |  |  |  |  |
| Mtif3 | -0.006710314 | 0.982004407 | 0.99459269 |  |  |  |  |  |  |  |  |
| Mtm1 | -0.008062753 | 0.767751757 | 0.920286747 |  |  |  |  |  |  |  |  |
| Mtmr12 | 0.181288323 | 0.025868123 | 0.123399609 |  |  |  |  |  |  |  |  |
| Mtmr2 | 0.000346986 | 0.879835197 | 0.959752724 |  |  |  |  |  |  |  |  |
| Mtmr3 | -0.106501509 | 0.39008837 | 0.701385221 |  |  |  |  |  |  |  |  |
| Mtmr4 | 0.06496542 | 0.349803222 | 0.669217079 |  |  |  |  | FMRP |  |  |  |
| Mtmr6 | -0.040902867 | 0.87366758 | 0.957983588 |  |  |  |  |  |  |  |  |
| Mtmr7 | -0.041732127 | 0.646093839 | 0.863334639 |  |  |  |  |  |  |  |  |
| Mto1 | 0.016854025 | 0.87483129 | 0.958600683 |  |  |  |  |  |  |  |  |
| Mtor | 0.115691562 | 0.15592103 | 0.434075441 |  |  |  |  |  |  |  |  |
| Mtpn | -0.040568542 | 0.66763767 | 0.875092018 |  |  |  |  |  |  |  |  |
| Mtrr | 0.368578979 | 0.007409931 | 0.046573372 |  | ID |  |  |  |  |  |  |
| Mtss1l | 0.037643716 | 0.481498629 | 0.767012795 |  |  |  |  |  |  |  |  |
| Mtus1 | -0.329653468 | 0.025384831 | 0.121969527 |  |  |  |  |  |  |  |  |
| Mtus2 | 0.007560058 | 0.822168595 | 0.941239661 |  |  |  |  |  |  |  |  |
| Mtx2 | 0.007835222 | 0.912786819 | 0.973857742 | SYN |  | ASD |  |  |  |  |  |
| Mtx3 | -0.044044736 | 0.655088959 | 0.86959601 |  |  |  |  |  |  |  |  |
| Mul1 | 0.026227279 | 0.711244684 | 0.893500957 |  |  |  |  |  |  |  |  |
| Mum1 | 0.067454219 | 0.573838445 | 0.820827732 |  |  |  |  |  |  |  |  |
| Mut | 0.048111911 | 0.510068316 | 0.786093307 |  |  |  |  |  |  |  |  |
| Mvb12b | 0.192747302 | 0.0226522 | 0.112080612 |  |  |  |  |  |  |  |  |
| Mvk | 0.279796104 | 0.027385795 | 0.128942798 |  |  |  |  |  |  |  |  |
| Mxd1 | -0.050944482 | 0.47923572 | 0.765984483 |  |  |  |  |  |  |  |  |
| Mxd4 | 0.278970869 | 0.005972387 | 0.039075243 |  |  |  |  |  |  |  |  |
| Mxi1 | -0.174734483 | 0.067550515 | 0.252712433 |  |  |  |  |  |  |  |  |
| Mxra7 | -1.270274651 | 3.22695E-11 | 1.14903E-09 |  |  |  |  |  |  |  |  |
| Myadm | 0.445042791 | 6.42245E-06 | 9.64697E-05 |  |  |  |  |  |  |  |  |
| Mybbp1a | -0.030943258 | 0.891045291 | 0.963919333 |  |  |  |  |  |  |  |  |
| Mycbp2 | -0.049477785 | 0.437019047 | 0.737692921 | SYN |  |  |  | FMRP |  |  |  |
| Myef2 | 0.187047293 | 0.054029112 | 0.216515785 |  |  |  |  |  |  |  |  |
| Myeov2 | 0.109076822 | 0.616983128 | 0.847289502 |  |  |  |  |  |  |  |  |
| Myg1 | 0.212259948 | 0.042948799 | 0.184686665 |  |  |  |  |  |  |  |  |
| Myh10 | -0.094522705 | 0.23370324 | 0.548048187 | SYN |  |  |  | FMRP |  |  |  |
| Myh14 | -0.142397301 | 0.241876991 | 0.557723052 | SYN |  |  |  |  |  |  |  |
| Myh9 | 0.096217056 | 0.188811797 | 0.485468048 | SYN |  |  |  |  |  |  |  |
| Myl12a | 0.127047513 | 0.429520011 | 0.73153195 |  |  |  |  |  |  |  |  |
| Myl12b | -0.011548055 | 0.88597554 | 0.962577588 |  |  |  |  |  |  |  |  |
| Myl6 | 0.185769748 | 0.108537761 | 0.348369088 | SYN |  |  |  |  |  |  |  |
| Mylip | 0.517758031 | 5.6034E-06 | 8.49672E-05 |  |  |  |  |  |  |  |  |
| Mylk | -0.495951951 | 0.024798018 | 0.12009046 |  |  |  |  |  |  |  |  |
| Myo10 | 0.267591925 | 0.005171361 | 0.034807405 |  |  |  |  | FMRP |  |  |  |
| Myo16 | -1.615265003 | 4.74781E-31 | 7.72828E-29 |  |  | ASD | ASD_sc | FMRP |  |  |  |
| Myo18a | -0.093518082 | 0.234021555 | 0.548217089 | SYN |  |  |  | FMRP |  |  |  |
| Myo1b | -1.265132256 | 2.77503E-29 | 4.02429E-27 | SYN |  |  |  |  |  |  |  |
| Myo1c | 0.269206852 | 0.036787557 | 0.162919242 | SYN |  |  |  |  |  |  |  |
| Myo1d | 0.044627404 | 0.781126572 | 0.92656842 | SYN |  |  |  |  |  |  |  |
| Myo5a | -0.209028084 | 0.014068883 | 0.077016752 | SYN | ID |  |  | FMRP |  |  |  |
| Myo5b | -0.455460409 | 0.000309737 | 0.003155126 |  |  |  |  |  |  |  |  |
| Myo5c | 0.461547513 | 0.134998033 | 0.399534068 | SYN |  |  |  |  |  |  |  |
| Myo6 | 0.100937132 | 0.316158194 | 0.633711949 | SYN |  |  |  |  |  |  |  |
| Myo7a | 0.029470528 | 0.833125118 | 0.943106438 |  |  |  |  |  |  |  |  |
| Myo9a | -0.07294222 | 0.603160391 | 0.838119735 |  |  |  |  |  |  |  |  |
| Myo9b | 0.022966483 | 0.946100986 | 0.986031813 |  |  | ASD | ASD_sc |  | SZdb |  | SZ_full |
| Myof | 0.283013152 | 0.361496399 | 0.678582085 |  |  |  |  |  |  |  |  |
| Mypop | 0.027471094 | 0.641439902 | 0.860142008 |  |  |  |  |  |  |  |  |
| Myrf | 0.390213869 | 0.139107389 | 0.406864883 |  |  |  |  |  |  |  |  |
| Myrip | 0.055948865 | 0.75207263 | 0.91454844 |  |  |  |  |  |  |  |  |
| Myt1 | -0.349001287 | 0.059212372 | 0.230604435 |  |  |  |  |  |  |  |  |
| Myt1l | 0.206353471 | 0.00509899 | 0.034383137 |  |  | ASD | ASD_sc | FMRP |  |  |  |
| Mzt1 | -0.066285265 | 0.776229627 | 0.924077297 |  |  |  |  |  |  |  |  |
| N28178 | -0.290518947 | 0.001016085 | 0.009055074 | SYN |  |  |  | FMRP |  |  |  |
| N4bp1 | -0.022540576 | 0.918322907 | 0.976215315 |  |  |  |  |  |  |  |  |
| N4bp2l1 | -0.031346732 | 0.519400793 | 0.790843055 |  |  |  |  |  |  |  |  |
| N4bp2l2 | 0.091601958 | 0.234078927 | 0.548217089 |  |  |  |  |  |  |  |  |
| N4bp3 | 0.710852899 | 1.95596E-08 | 4.71286E-07 |  |  |  |  |  |  |  |  |
| Naa10 | 0.343794423 | 0.144081351 | 0.416343008 |  | ID |  |  |  |  |  |  |
| Naa15 | 0.01269309 | 0.734588781 | 0.90627493 |  |  | ASD | ASD_sc |  |  |  |  |
| Naa16 | 0.14876844 | 0.598151449 | 0.835219314 |  |  |  |  |  |  |  |  |
| Naa25 | -0.034381911 | 0.876036643 | 0.958877817 |  |  |  |  |  |  |  |  |
| Naa30 | -0.13719691 | 0.418066377 | 0.725719582 |  |  |  |  |  |  |  |  |
| Naa35 | 0.03538975 | 0.677924976 | 0.878273493 |  |  |  |  |  |  |  |  |
| Naa40 | -0.048683475 | 0.432529895 | 0.733800692 |  |  |  |  |  |  |  |  |
| Naa50 | -0.140465063 | 0.319760037 | 0.637601513 |  |  |  |  |  |  |  |  |
| Naa60 | -0.10220874 | 0.250668143 | 0.568337129 |  |  |  |  |  |  |  |  |
| Naaa | -0.248680684 | 0.051389904 | 0.209232199 |  |  |  |  |  |  |  |  |
| Nab1 | 0.094508334 | 0.310851355 | 0.627487766 |  |  |  |  |  |  |  |  |
| Naca | 0.140111993 | 0.396091452 | 0.706474453 |  |  |  |  |  |  |  |  |
| Nacad | 0.108494535 | 0.211566125 | 0.518502158 |  |  |  |  |  |  |  |  |
| Nacc1 | -0.029063258 | 0.861022155 | 0.952894784 |  |  |  |  |  |  |  |  |
| Nacc2 | -0.248211027 | 0.006923176 | 0.044317217 |  |  |  |  |  |  |  |  |
| Nadk | -0.038222429 | 0.577010991 | 0.822415952 |  |  |  |  |  |  |  |  |
| Nadkd1 | 0.209722733 | 0.022195376 | 0.110281768 |  |  |  |  |  |  |  |  |
| Nae1 | -0.038637 | 0.599270428 | 0.835219314 |  |  |  |  |  |  |  |  |
| Nagk | -0.046857614 | 0.610545603 | 0.842510679 |  |  |  |  |  |  |  |  |
| Nalcn | 0.249465284 | 0.002259181 | 0.017893968 |  |  |  |  |  |  |  |  |
| Nanp | 0.015135693 | 0.956158129 | 0.989355248 |  |  |  |  |  |  |  |  |
| Nap1l1 | -0.403314672 | 7.24412E-07 | 1.31316E-05 | SYN |  |  |  |  |  |  |  |
| Nap1l3 | 0.146108822 | 0.12032516 | 0.371693833 |  |  |  |  |  |  |  |  |
| Nap1l4 | 0.012630993 | 0.947715608 | 0.986554384 | SYN |  |  |  |  |  |  |  |
| Nap1l5 | -0.122200091 | 0.155856075 | 0.434046107 |  |  |  |  |  |  |  |  |
| Napa | -0.187605285 | 0.022081111 | 0.110005586 | SYN |  |  |  |  |  |  |  |
| Napb | -0.04465033 | 0.834246065 | 0.943106438 | SYN |  |  |  |  |  |  |  |
| Napepld | -0.112276965 | 0.418503345 | 0.725964045 |  |  |  |  |  |  |  |  |
| Napg | -0.1142401 | 0.24671407 | 0.563675572 | SYN |  |  |  |  |  |  |  |
| Narf | 0.182740227 | 0.120106993 | 0.371163648 |  |  |  |  |  |  |  |  |
| Narfl | -0.044940882 | 0.930354925 | 0.981434219 |  |  |  |  |  |  |  |  |
| Nars | 0.023799034 | 0.694420294 | 0.885065682 |  |  |  |  |  |  |  |  |
| Nat10 | 0.100905486 | 0.546650659 | 0.806312971 |  |  |  |  |  |  |  |  |
| Nat14 | 0.267911144 | 0.046638317 | 0.196610579 |  |  |  |  |  |  |  |  |
| Nat8l | -0.095811987 | 0.201047627 | 0.504464218 |  |  |  |  | FMRP |  |  |  |
| Nav1 | -0.035474249 | 0.805853643 | 0.936945007 |  |  |  |  | FMRP |  |  |  |
| Nav2 | -0.012926633 | 0.745315395 | 0.911928524 |  |  |  |  | FMRP |  |  |  |
| Nav3 | -0.246840284 | 0.00266318 | 0.020396476 |  |  |  |  | FMRP |  |  |  |
| Nbas | 0.030048234 | 0.902367843 | 0.969220665 |  |  |  |  |  |  |  |  |
| Nbea | -0.00243421 | 0.794457433 | 0.933219384 | SYN |  | ASD | ASD_sc | FMRP |  |  |  |
| Nbeal1 | -0.16028963 | 0.186797377 | 0.482680181 |  |  |  |  |  |  |  |  |
| Nbl1 | -0.431995366 | 0.234898167 | 0.549105446 |  |  |  |  |  |  |  |  |
| Nbr1 | -0.079797672 | 0.446008486 | 0.744112298 |  |  |  |  |  |  |  |  |
| Ncald | 0.36573433 | 6.9191E-06 | 0.000103153 | SYN |  |  |  |  |  |  |  |
| Ncam1 | 0.048294101 | 0.624389311 | 0.850480636 | SYN |  |  |  | FMRP |  |  |  |
| Ncam2 | -0.2348998 | 0.018777678 | 0.096939004 | SYN |  |  |  |  |  |  |  |
| Ncan | 0.036317136 | 0.710269471 | 0.893043852 | SYN |  |  |  | FMRP |  | SZ_108 | SZ_full |
| Ncapd2 | 0.091153555 | 0.822092182 | 0.941239661 |  |  |  |  |  |  |  |  |
| Ncapd3 | -0.161018354 | 0.080703353 | 0.285703483 |  |  |  |  |  |  |  |  |
| Ncaph2 | 0.001742553 | 0.948757737 | 0.986912479 |  |  |  |  |  |  |  |  |
| Ncbp1 | 0.075463779 | 0.31911492 | 0.636792745 |  |  |  |  |  |  |  |  |
| Ncbp2 | 0.063372323 | 0.67296414 | 0.877823808 |  |  |  |  |  |  |  |  |
| Ncdn | 0.161881176 | 0.077458035 | 0.277042731 | SYN |  |  |  | FMRP |  |  |  |
| Nceh1 | -0.032832338 | 0.963737048 | 0.990416798 |  |  |  |  |  |  |  |  |
| Nck2 | 0.015662972 | 0.691605937 | 0.884120561 |  |  |  |  |  |  |  |  |
| Nckap1 | 0.149705344 | 0.03350194 | 0.151566347 | SYN |  | ASD | ASD_sc | FMRP |  |  |  |
| Nckipsd | -0.113301781 | 0.355786595 | 0.674719677 | SYN |  |  |  |  |  |  |  |
| Ncl | 0.029648558 | 0.602757001 | 0.837705147 |  |  |  |  |  |  |  |  |
| Ncln | 0.033567045 | 0.78434547 | 0.928282627 |  |  |  |  |  |  |  |  |
| Ncoa1 | 0.126246564 | 0.073478879 | 0.266879572 |  |  |  |  | FMRP |  |  |  |
| Ncoa2 | -0.116843247 | 0.257394194 | 0.575063331 |  |  |  |  | FMRP |  |  |  |
| Ncoa3 | -0.166636931 | 0.056573604 | 0.223207782 |  |  |  |  |  |  |  |  |
| Ncoa4 | 0.227930661 | 0.005346301 | 0.035629216 |  |  |  |  |  |  |  |  |
| Ncoa5 | 0.150268793 | 0.148233526 | 0.422857871 |  |  |  |  |  |  |  |  |
| Ncoa6 | -0.183667793 | 0.146174576 | 0.419208794 |  |  |  |  | FMRP |  |  |  |
| Ncoa7 | -0.088263957 | 0.366354748 | 0.683679333 |  |  |  |  |  |  |  |  |
| Ncor1 | -0.051821659 | 0.476084959 | 0.764668463 |  |  |  |  | FMRP |  |  |  |
| Ncor2 | 0.235502399 | 0.020975281 | 0.105684676 |  |  |  |  | FMRP |  |  |  |
| Ncs1 | 0.007873915 | 0.935244354 | 0.984753659 |  |  |  |  |  |  |  |  |
| Ncstn | 0.022429585 | 0.69683025 | 0.886206371 |  |  |  |  |  |  |  |  |
| Nde1 | 0.233909591 | 0.050130849 | 0.206317673 |  |  |  |  |  |  |  |  |
| Ndel1 | 0.003626666 | 0.997659069 | 1 | SYN |  |  |  |  |  |  |  |
| Ndfip1 | -0.34553465 | 0.000249183 | 0.002611671 |  |  |  |  |  |  |  |  |
| Ndn | 0.092160869 | 0.550878424 | 0.807666914 |  |  |  |  |  |  |  |  |
| Ndor1 | 0.078950556 | 0.668327045 | 0.875497086 |  |  |  |  |  |  |  |  |
| Ndrg1 | 0.326601949 | 0.028235127 | 0.132084089 | SYN |  |  |  |  |  |  |  |
| Ndrg2 | -0.080320863 | 0.446038227 | 0.744112298 | SYN |  |  |  | FMRP |  |  |  |
| Ndrg3 | -0.013039614 | 0.886944636 | 0.962888134 |  |  |  |  |  |  |  |  |
| Ndrg4 | -0.013864825 | 0.819794675 | 0.941021502 |  |  |  |  | FMRP |  |  |  |
| Ndst2 | 0.069337034 | 0.541009285 | 0.802680899 |  |  |  |  |  |  |  |  |
| Ndst3 | 0.013707751 | 0.821333598 | 0.941239661 |  |  |  |  |  |  |  |  |
| Ndufa1 | -0.058088747 | 0.441717257 | 0.741088944 |  | ID |  |  |  |  |  |  |
| Ndufa10 | 0.113534145 | 0.228053603 | 0.540872893 | SYN |  |  |  |  |  |  |  |
| Ndufa11 | -0.125367733 | 0.597088226 | 0.834737271 |  |  |  |  |  |  |  |  |
| Ndufa12 | 0.195262823 | 0.512663619 | 0.787509131 | SYN |  |  |  |  |  |  |  |
| Ndufa13 | 0.106760547 | 0.942469116 | 0.985900058 | SYN |  |  |  |  |  | SZ_108 | SZ_full |
| Ndufa2 | 0.1027043 | 0.869841256 | 0.956485207 | SYN |  |  |  |  |  | SZ_108 | SZ_full |
| Ndufa3 | -0.245231159 | 0.266760041 | 0.583885316 |  |  |  |  |  |  |  |  |
| Ndufa4 | 0.077564023 | 0.47956772 | 0.765984483 | SYN |  |  |  |  |  |  |  |
| Ndufa5 | 0.048750941 | 0.867068022 | 0.954948156 | SYN |  | ASD | ASD_sc |  |  |  |  |
| Ndufa6 | -0.03384298 | 0.573585368 | 0.820827732 | SYN |  |  |  |  |  | SZ_108 | SZ_full |
| Ndufa8 | 0.056421264 | 0.770408163 | 0.921167131 | SYN |  |  |  |  |  |  |  |
| Ndufa9 | 0.044353515 | 0.536240667 | 0.799449638 | SYN |  |  |  |  |  |  |  |
| Ndufab1 | 0.080163498 | 0.669304788 | 0.87586136 |  |  |  |  |  |  |  |  |
| Ndufaf3 | 0.115454435 | 0.700472563 | 0.887191673 |  |  |  |  |  |  |  |  |
| Ndufaf5 | 0.050095079 | 0.711225664 | 0.893500957 |  |  |  |  |  |  |  |  |
| Ndufb10 | 0.20620907 | 0.644847583 | 0.862391737 | SYN |  |  |  |  |  |  |  |
| Ndufb11 | 0.151071489 | 0.740141698 | 0.909610197 |  |  |  |  |  |  |  |  |
| Ndufb2 | 0.053697562 | 0.48607317 | 0.771274148 |  |  |  |  |  |  |  |  |
| Ndufb3 | 0.080296847 | 0.79029636 | 0.931851876 |  |  |  |  |  |  |  |  |
| Ndufb5 | -0.049091748 | 0.533290329 | 0.797585535 |  |  |  |  |  |  |  |  |
| Ndufb6 | 0.119563685 | 0.348207767 | 0.667863914 | SYN |  |  |  |  |  |  |  |
| Ndufb8 | 0.257438545 | 0.021702594 | 0.10859466 | SYN |  |  |  |  |  |  |  |
| Ndufb9 | 0.054724404 | 0.841730725 | 0.945289559 | SYN |  |  |  |  |  |  |  |
| Ndufc1 | -0.174630862 | 0.242540842 | 0.558239142 |  |  |  |  |  |  |  |  |
| Ndufc2 | -0.000116757 | 0.767009928 | 0.920286747 | SYN |  |  |  |  |  |  |  |
| Ndufs1 | -0.05129983 | 0.701840061 | 0.887565614 | SYN | ID |  |  |  |  |  |  |
| Ndufs2 | -0.017536898 | 0.835704788 | 0.943188599 | SYN | ID |  |  |  |  |  |  |
| Ndufs3 | -0.089252496 | 0.282826175 | 0.601392047 | SYN |  |  |  |  |  |  |  |
| Ndufs4 | -0.084009662 | 0.274580955 | 0.592033284 |  | ID |  |  |  |  |  |  |
| Ndufs5 | -0.196123384 | 0.322756703 | 0.639897456 |  |  |  |  |  |  |  |  |
| Ndufs6 | -0.015463686 | 0.716785136 | 0.897078024 | SYN |  |  |  |  |  |  |  |
| Ndufs7 | 0.23372455 | 0.291976503 | 0.608837802 | SYN | ID |  |  |  |  |  |  |
| Ndufs8 | 0.062528776 | 0.58152218 | 0.825342533 |  | ID |  |  |  |  |  |  |
| Ndufv1 | 0.153396065 | 0.093708097 | 0.316032044 | SYN | ID |  |  |  |  |  |  |
| Ndufv2 | 0.143583017 | 0.160732928 | 0.442917039 | SYN |  |  |  |  | SZdb |  | SZ_full |
| Ndufv3 | -0.069174076 | 0.393806282 | 0.705533207 | SYN |  |  |  |  |  |  |  |
| Neat1 | 0.298886859 | 0.007465328 | 0.046847726 |  |  |  |  |  |  |  |  |
| Nebl | 0.056672556 | 0.806719596 | 0.937002402 |  |  |  |  |  |  |  |  |
| Necab1 | -0.135756646 | 0.765423221 | 0.920261623 |  |  |  |  |  |  |  |  |
| Necab2 | -0.182815347 | 0.733916463 | 0.905992378 |  |  |  |  |  |  |  |  |
| Necab3 | 0.419334299 | 0.001620456 | 0.013648105 |  |  |  |  |  |  |  |  |
| Necap1 | 0.103135688 | 0.167063337 | 0.452922221 |  |  |  |  |  |  |  |  |
| Nedd4 | 0.156349468 | 0.031346493 | 0.144103531 | SYN |  |  |  | FMRP |  |  |  |
| Nedd4l | -0.211305063 | 0.107379722 | 0.346052663 |  |  |  |  |  |  |  |  |
| Nedd9 | 1.133424785 | 1.18827E-13 | 5.54247E-12 |  |  |  |  |  |  |  |  |
| Nefh | 0.20372752 | 0.391106555 | 0.702663556 | SYN |  |  |  |  |  |  |  |
| Nefl | 0.322680397 | 0.000207822 | 0.002204243 | SYN |  | ASD |  |  |  |  |  |
| Nefm | -0.094983014 | 0.203003651 | 0.507095872 | SYN |  |  |  |  |  |  |  |
| Negr1 | -1.13406146 | 1.54535E-31 | 2.6795E-29 | SYN |  |  |  |  |  |  |  |
| Nek1 | -0.204856974 | 0.023392352 | 0.11488756 |  |  |  |  |  |  | SZ_108 | SZ_full |
| Nek6 | -0.136966826 | 0.473581091 | 0.76408458 |  |  |  |  |  |  |  |  |
| Nek9 | -0.007751057 | 0.819457434 | 0.941021502 |  |  |  |  |  |  |  |  |
| Nelfb | -0.005693385 | 0.936063584 | 0.98530815 |  |  |  |  |  |  |  |  |
| Nelfcd | 0.100982173 | 0.261947626 | 0.578912237 |  |  |  |  |  |  |  |  |
| Nell1 | -1.341579498 | 2.18026E-26 | 2.59549E-24 |  |  | ASD |  |  |  |  |  |
| Nell2 | 0.507014461 | 1.02253E-10 | 3.37353E-09 |  |  |  |  |  |  |  |  |
| Nemf | 0.096460532 | 0.519874965 | 0.790843055 |  |  |  |  |  |  |  |  |
| Nenf | -0.065997762 | 0.409222153 | 0.719062719 |  |  |  |  |  |  |  |  |
| Neo1 | -0.036174929 | 0.720212051 | 0.897994027 | SYN |  |  |  |  |  |  |  |
| Nes | -0.188065014 | 0.362050435 | 0.679302345 |  |  |  |  |  |  |  |  |
| Net1 | -1.026930348 | 3.88304E-19 | 2.9218E-17 |  |  |  |  |  |  |  |  |
| Neto1 | 0.190612209 | 0.039336148 | 0.172103738 |  |  |  |  |  |  |  |  |
| Neto2 | -0.430122801 | 0.004814728 | 0.032822456 |  |  |  |  |  |  |  |  |
| Neu1 | -0.095858464 | 0.240374678 | 0.556040728 |  | ID |  |  |  |  |  |  |
| Neurl1a | -0.571467233 | 7.78341E-08 | 1.66883E-06 |  |  |  |  |  |  |  |  |
| Neurl1b | -0.169779153 | 0.025431619 | 0.122047289 |  |  |  |  |  |  |  |  |
| Neurl4 | 0.036949029 | 0.599000876 | 0.835219314 |  |  |  |  |  |  |  |  |
| Neurod2 | 0.671120155 | 4.81841E-08 | 1.07652E-06 |  |  |  |  |  |  |  |  |
| Neurod6 | 1.319584923 | 7.40764E-21 | 5.90833E-19 |  |  |  |  |  |  |  |  |
| Nexn | -0.369512388 | 0.378935338 | 0.693655256 |  |  |  |  |  |  |  |  |
| Nf1 | -0.082709597 | 0.416115381 | 0.724350718 | SYN | ID | ASD | ASD_sc | FMRP |  |  |  |
| Nf2 | 0.318732201 | 0.000517263 | 0.004982719 |  |  |  |  |  |  |  |  |
| Nfasc | 0.15876482 | 0.149908267 | 0.425625478 | SYN |  |  |  |  |  |  |  |
| Nfat5 | 0.117118662 | 0.160196533 | 0.441814506 |  |  |  |  |  |  |  |  |
| Nfatc3 | -0.172799845 | 0.186109147 | 0.481595848 |  |  |  |  |  |  | SZ_108 | SZ_full |
| Nfe2l1 | 0.103952942 | 0.157282945 | 0.437024788 |  |  |  |  |  |  |  |  |
| Nfia | -0.083967196 | 0.463564384 | 0.75812785 |  |  | ASD |  |  |  |  |  |
| Nfib | -0.500516866 | 1.04838E-07 | 2.18327E-06 |  |  |  |  |  |  |  |  |
| Nfic | -0.019056071 | 0.540386636 | 0.802535845 |  |  |  |  | FMRP |  |  |  |
| Nfix | -0.151877797 | 0.169155242 | 0.457040044 |  |  |  |  | FMRP |  |  |  |
| Nfkb1 | -0.032131742 | 0.545312979 | 0.805395969 |  |  |  |  |  |  |  |  |
| Nfkbib | 0.098547266 | 0.842766604 | 0.94530975 |  |  |  |  |  |  |  |  |
| Nfkbiz | 0.579299776 | 0.000912544 | 0.008252211 |  |  |  |  |  |  |  |  |
| Nfrkb | -0.027452677 | 0.655594168 | 0.86959601 |  |  |  |  |  |  |  |  |
| Nfu1 | -0.137252352 | 0.14848039 | 0.423108106 |  |  |  |  |  |  |  |  |
| Nfx1 | -0.126112951 | 0.23958321 | 0.554531538 |  |  |  |  |  |  |  |  |
| Nfyb | 0.087927489 | 0.453126723 | 0.750288301 |  |  |  |  |  |  |  |  |
| Nfyc | 0.076784513 | 0.425473806 | 0.730430279 |  |  |  |  |  |  |  |  |
| Ngdn | 0.213688133 | 0.213397757 | 0.520155963 |  |  |  |  |  |  |  |  |
| Ngef | -0.292206525 | 0.000593809 | 0.005624963 | SYN |  |  |  | FMRP |  | SZ_108 | SZ_full |
| Ngfrap1 | 0.223974473 | 0.150639851 | 0.426518798 |  |  |  |  |  |  |  |  |
| Ngly1 | -0.06428494 | 0.416440743 | 0.724350718 |  |  |  |  |  |  |  |  |
| Nhlrc1 | 0.270287699 | 0.015622374 | 0.0837956 |  |  |  |  |  |  |  |  |
| Nhp2 | 0.302252315 | 0.057977796 | 0.22690427 |  |  |  |  |  |  |  |  |
| Nhp2l1 | -0.122697592 | 0.370569976 | 0.686565884 |  |  |  |  |  |  |  |  |
| Nhsl2 | 0.429411177 | 8.02757E-07 | 1.44533E-05 |  |  |  |  |  |  |  |  |
| Nicn1 | 0.119948113 | 0.174194164 | 0.463433173 |  |  |  |  |  |  |  |  |
| Nin | 0.19774006 | 0.031817978 | 0.145516165 |  |  |  |  |  |  |  |  |
| Ninl | -0.680147996 | 0.004788898 | 0.032730295 |  |  |  |  |  |  |  |  |
| Nip7 | 0.151289625 | 0.128556292 | 0.388101812 |  |  |  |  |  |  |  |  |
| Nipa1 | 0.025727496 | 0.81656002 | 0.939945551 |  |  | ASD |  |  |  |  |  |
| Nipa2 | -0.023920705 | 0.775222912 | 0.924077297 |  |  | ASD |  |  |  |  |  |
| Nipal2 | -0.243425137 | 0.063105041 | 0.241635047 |  |  |  |  |  |  |  |  |
| Nipal3 | -0.071415583 | 0.403210471 | 0.712451642 |  |  |  |  |  |  |  |  |
| Nipsnap1 | 0.025250359 | 0.710231557 | 0.893043852 | SYN |  |  |  |  |  |  |  |
| Nisch | 0.056587377 | 0.31704618 | 0.634185332 |  |  |  |  | FMRP |  | SZ_108 | SZ_full |
| Nit1 | -0.240110604 | 0.050580701 | 0.207265309 |  |  |  |  |  |  |  |  |
| Nkain1 | 0.179941726 | 0.131085553 | 0.392174934 |  |  |  |  |  |  |  |  |
| Nkain2 | -0.027954754 | 0.562118265 | 0.814652203 |  |  |  |  |  |  |  |  |
| Nkap | 0.055430707 | 0.514147211 | 0.787995408 |  |  |  |  |  |  |  |  |
| Nkiras1 | -0.043023925 | 0.59926055 | 0.835219314 | SYN |  |  |  |  |  |  |  |
| Nkrf | 0.391679229 | 0.000678017 | 0.006352987 |  |  |  |  |  |  |  |  |
| Nktr | 0.110002775 | 0.355393466 | 0.674472305 |  |  |  |  |  |  |  |  |
| Nle1 | 0.202481241 | 0.286063655 | 0.604177883 |  |  |  |  |  |  |  |  |
| Nlgn1 | -0.758618803 | 6.23445E-13 | 2.67344E-11 | SYN |  | ASD | ASD_sc |  |  |  |  |
| Nlgn2 | -0.093283756 | 0.398986842 | 0.709155315 | SYN |  | ASD |  | FMRP |  |  |  |
| Nlgn3 | 0.167250925 | 0.031879646 | 0.145589334 | SYN | ID | ASD | ASD_sc | FMRP |  |  |  |
| Nlk | 0.016785545 | 0.905167283 | 0.970378259 |  |  |  |  |  |  |  |  |
| Nmd3 | -0.103434548 | 0.437103698 | 0.737692921 |  |  |  |  |  |  |  |  |
| Nme1 | -0.232349803 | 0.018828767 | 0.097139876 | SYN |  |  |  |  |  |  |  |
| Nme2 | -0.030436811 | 0.553256975 | 0.808072808 |  |  |  |  |  |  |  |  |
| Nme7 | 0.061370061 | 0.564638899 | 0.816750065 |  |  |  |  |  |  |  |  |
| Nmnat2 | -0.227648504 | 0.019143102 | 0.098571506 |  |  |  |  |  |  |  |  |
| Nmral1 | 0.384250365 | 0.004809285 | 0.032820759 |  |  |  |  |  |  |  |  |
| Nmt1 | 0.227516632 | 0.00660134 | 0.042495794 |  |  |  |  |  |  |  |  |
| Nmt2 | -0.050980676 | 0.496103457 | 0.77686598 |  |  |  |  |  |  |  |  |
| Nnt | -0.001592271 | 0.663163012 | 0.872692325 | SYN |  |  |  |  |  |  |  |
| Nob1 | 0.094425615 | 0.356299063 | 0.675182068 |  |  |  |  |  |  |  |  |
| Nod1 | 0.185530543 | 0.272517943 | 0.590748776 |  |  |  |  |  |  |  |  |
| Nol10 | -0.042090984 | 0.564260943 | 0.816385912 |  |  |  |  |  |  |  |  |
| Nol11 | 0.040882318 | 0.642765919 | 0.861051556 |  |  |  |  |  |  |  |  |
| Nol4 | -0.999397382 | 1.77605E-25 | 1.96747E-23 |  |  |  |  |  |  |  |  |
| Nol6 | 0.135153811 | 0.098539015 | 0.326933106 |  |  |  |  |  |  |  |  |
| Nol7 | -0.066577584 | 0.50244833 | 0.782106171 |  |  |  |  |  |  |  |  |
| Nol8 | 0.034181443 | 0.745268512 | 0.911928524 |  |  |  |  |  |  |  |  |
| Nol9 | 0.095301665 | 0.519610769 | 0.790843055 |  |  |  |  |  |  |  |  |
| Nolc1 | 0.147856153 | 0.279917464 | 0.597277071 |  |  |  |  |  |  |  |  |
| Nom1 | -0.075520801 | 0.551537205 | 0.807908309 |  |  |  |  |  |  |  |  |
| Nomo1 | -0.036088597 | 0.790651869 | 0.931851876 | SYN |  |  |  | FMRP |  |  |  |
| Nono | 0.135892849 | 0.088264896 | 0.302697643 |  |  |  |  |  |  |  |  |
| Nop14 | 0.042675815 | 0.660566507 | 0.870999911 |  |  |  |  |  |  |  |  |
| Nop2 | 0.088400414 | 0.510596395 | 0.786200163 |  |  |  |  |  |  |  |  |
| Nop56 | 0.209363325 | 0.060328671 | 0.233469906 |  |  |  |  |  |  |  |  |
| Nop9 | -0.020770193 | 0.945533564 | 0.985962641 |  |  |  |  |  |  |  |  |
| Nos1 | 1.544417302 | 3.15386E-05 | 0.000406384 | SYN |  |  |  |  | SZdb |  | SZ_full |
| Nos1ap | -0.155389557 | 0.249868539 | 0.567244294 |  |  | ASD |  |  | SZdb |  | SZ_full |
| Nosip | 0.117406821 | 0.293005157 | 0.610185151 |  |  |  |  |  |  | SZ_108 | SZ_full |
| Notch1 | 0.336098452 | 0.000397596 | 0.003949223 |  |  |  |  |  |  |  |  |
| Notch2 | 0.283537526 | 0.047869785 | 0.200191963 |  |  |  |  |  |  |  |  |
| Notch3 | 0.062395077 | 0.471707396 | 0.76369839 |  |  |  |  |  |  |  |  |
| Nov | 0.831176413 | 0.000175455 | 0.001888564 |  |  |  |  |  |  |  |  |
| Nova1 | 0.257537179 | 0.137074184 | 0.404178813 |  |  |  |  |  |  |  |  |
| Nova2 | 0.057667601 | 0.447222881 | 0.744686785 |  |  |  |  |  |  |  |  |
| Noxred1 | 0.547742032 | 0.255296725 | 0.572540822 |  |  |  |  |  |  |  |  |
| Npas1 | 0.551291822 | 0.000496489 | 0.004805819 |  |  |  |  |  |  |  |  |
| Npas2 | 0.647470376 | 3.04076E-11 | 1.08758E-09 |  |  | ASD | ASD_sc | FMRP |  |  |  |
| Npas3 | 0.02824145 | 0.902959403 | 0.969220665 |  |  |  |  |  |  |  |  |
| Npas4 | -0.082369793 | 0.780479245 | 0.926216702 |  |  |  |  |  |  |  |  |
| Npc1 | 0.25869447 | 0.009236647 | 0.055601128 |  | ID |  |  |  |  |  |  |
| Npc1l1 | 0.874977846 | 0.001440518 | 0.012301469 |  |  |  |  |  |  |  |  |
| Npdc1 | 0.264440375 | 0.007013045 | 0.044570553 |  |  |  |  |  |  |  |  |
| Npepl1 | 0.050759664 | 0.654635314 | 0.86959601 |  |  |  |  |  |  |  |  |
| Npepps | -0.009640147 | 0.792531743 | 0.932197785 | SYN |  |  |  |  |  |  |  |
| Nphp1 | -0.051536115 | 0.995796868 | 1 |  |  |  |  |  |  |  |  |
| Nphp4 | -0.009059798 | 0.740915128 | 0.910061682 |  |  |  |  |  |  |  |  |
| Npm1 | 0.111219938 | 0.21297193 | 0.520155963 | SYN |  |  |  |  |  |  |  |
| Npr2 | -0.636710196 | 6.51183E-10 | 1.938E-08 |  |  |  |  |  |  |  |  |
| Nptn | -0.278945744 | 0.000771263 | 0.007062682 | SYN |  |  |  |  |  |  |  |
| Nptx1 | 0.551879847 | 2.49983E-12 | 9.87061E-11 | SYN |  |  |  |  |  |  |  |
| Npy | 0.550445987 | 0.032023749 | 0.14603855 |  |  |  |  |  | SZdb |  | SZ_full |
| Npy1r | -0.200460925 | 0.065529265 | 0.247589493 |  |  |  |  |  |  |  |  |
| Nr1d1 | -0.226683722 | 0.074928824 | 0.27017735 |  |  |  |  |  |  |  |  |
| Nr1d2 | -0.032328745 | 0.770865331 | 0.921167131 |  |  |  |  |  |  |  |  |
| Nr1h2 | -0.134263379 | 0.261719471 | 0.578754638 |  |  |  |  |  |  |  |  |
| Nr2c1 | 0.017120563 | 0.957600616 | 0.989355248 |  |  |  |  |  |  |  |  |
| Nr2c2 | -0.130510406 | 0.234106775 | 0.548217089 |  |  |  |  |  |  |  |  |
| Nr2c2ap | 0.281940364 | 0.063931253 | 0.243603331 |  |  |  |  |  |  |  |  |
| Nr2f1 | -0.678320657 | 1.35724E-08 | 3.3515E-07 |  |  |  |  | FMRP |  |  |  |
| Nr2f6 | 0.073588996 | 0.426293397 | 0.73059413 |  |  |  |  |  |  |  |  |
| Nr3c1 | 0.164014715 | 0.04963616 | 0.20521492 |  |  |  |  |  |  |  |  |
| Nr3c2 | 1.066837391 | 2.13828E-24 | 2.21493E-22 |  |  | ASD | ASD_sc |  |  |  |  |
| Nr4a1 | -0.043671577 | 0.763855649 | 0.919979858 |  |  |  |  |  |  |  |  |
| Nr4a3 | 2.122551203 | 6.68723E-12 | 2.53987E-10 |  |  |  |  |  |  |  |  |
| Nrarp | -0.460314529 | 0.00073955 | 0.006832777 |  |  |  |  |  |  |  |  |
| Nras | 0.135853441 | 0.203128491 | 0.507129388 | SYN |  |  |  |  |  |  |  |
| Nrbf2 | 0.040304105 | 0.824590277 | 0.941950287 |  |  |  |  |  |  |  |  |
| Nrbp1 | -0.102229377 | 0.310064354 | 0.627296021 |  |  |  |  |  |  |  |  |
| Nrbp2 | 0.066235411 | 0.444572787 | 0.743066334 |  |  |  |  |  |  |  |  |
| Nrcam | -0.064404713 | 0.366585192 | 0.683745407 | SYN |  | ASD | ASD_sc |  |  |  |  |
| Nrd1 | -0.099819435 | 0.268809534 | 0.587243179 |  |  |  |  |  |  |  |  |
| Nrep | 0.051892018 | 0.773793646 | 0.923227841 |  |  |  |  |  |  |  |  |
| Nrg3 | -0.364551458 | 0.045081951 | 0.191438681 |  |  |  |  |  | SZdb |  | SZ_full |
| Nrgn | -0.300132549 | 0.048879995 | 0.203042489 |  |  |  |  | FMRP | SZdb |  | SZ_full |
| Nrip3 | -0.40067376 | 0.000698774 | 0.006511004 |  |  |  |  |  |  |  |  |
| Nrn1 | -0.436606232 | 4.13219E-07 | 7.81004E-06 | SYN |  |  |  |  |  |  |  |
| Nrp1 | 0.535617749 | 2.33931E-05 | 0.000309426 |  |  |  |  |  |  |  |  |
| Nrsn1 | -1.234171685 | 7.91588E-49 | 3.50761E-46 |  |  |  |  |  |  |  |  |
| Nrsn2 | 0.53831313 | 0.002348114 | 0.018432281 |  |  |  |  |  |  |  |  |
| Nrxn1 | -0.318767369 | 5.14432E-05 | 0.000632221 | SYN | ID | ASD | ASD_sc | FMRP |  |  |  |
| Nrxn2 | -0.177005071 | 0.074221839 | 0.268477727 | SYN |  | ASD | ASD_sc | FMRP |  |  |  |
| Nrxn3 | -0.53491501 | 2.09388E-08 | 4.98531E-07 | SYN |  | ASD | ASD_sc | FMRP |  |  |  |
| Nsa2 | 0.079108365 | 0.581191635 | 0.825130737 |  |  |  |  |  |  |  |  |
| Nsd1 | 0.001654972 | 0.820346862 | 0.941239661 |  | ID | ASD | ASD_sc | FMRP |  |  |  |
| Nsdhl | 0.116908927 | 0.248337212 | 0.56544732 |  | ID |  |  |  |  |  |  |
| Nsf | 0.263209454 | 0.00032485 | 0.003292256 | SYN |  |  |  | FMRP |  |  |  |
| Nsg1 | 0.012474462 | 0.757731163 | 0.916884789 |  |  |  |  |  |  |  |  |
| Nsg2 | 0.02226796 | 0.463915738 | 0.758236051 |  |  |  |  |  |  |  |  |
| Nsl1 | -0.006144739 | 0.96400655 | 0.990416798 |  |  |  |  |  |  |  |  |
| Nsmaf | -0.206269573 | 0.023241381 | 0.114359413 |  |  |  |  |  |  |  |  |
| Nsmce1 | 0.067648684 | 0.476102393 | 0.764668463 |  |  |  |  |  |  |  |  |
| Nsmf | 0.251712552 | 0.002789246 | 0.021147366 |  |  |  |  |  |  |  |  |
| Nt5c | 0.295390098 | 0.320011129 | 0.637688241 |  |  |  |  |  |  |  |  |
| Nt5c1a | 1.010282784 | 0.007826717 | 0.04877023 |  |  |  |  |  |  |  |  |
| Nt5c2 | -0.297047832 | 0.002580029 | 0.019920924 |  |  |  |  |  |  | SZ_108 | SZ_full |
| Nt5c3 | 0.597422445 | 2.92919E-08 | 6.85138E-07 |  |  |  |  |  |  |  |  |
| Nt5c3l | 0.052761159 | 0.787782374 | 0.930315697 |  |  |  |  |  |  |  |  |
| Nt5dc2 | 0.411871244 | 0.195393407 | 0.496552674 |  |  |  |  |  |  | SZ_108 | SZ_full |
| Nt5dc3 | -0.150662325 | 0.255219949 | 0.572540822 |  |  |  |  |  |  |  |  |
| Nt5m | 0.068005474 | 0.534608183 | 0.798060053 |  |  |  |  |  |  |  |  |
| Ntan1 | -0.021003803 | 0.642385386 | 0.860830954 |  |  |  |  |  |  |  |  |
| Ntm | -0.376388918 | 0.001027159 | 0.009123187 | SYN |  |  |  |  |  |  |  |
| Ntn3 | -0.016198386 | 0.96571075 | 0.990416798 |  |  |  |  |  |  |  |  |
| Ntng1 | -1.470902984 | 7.92405E-32 | 1.43641E-29 |  |  | ASD | ASD_sc |  | SZdb |  | SZ_full |
| Ntng2 | 0.825859075 | 2.61939E-06 | 4.19523E-05 |  |  |  |  |  | SZdb |  | SZ_full |
| Ntrk2 | -0.074783474 | 0.620900761 | 0.849066642 | SYN |  |  |  | FMRP |  |  |  |
| Ntrk3 | 0.115885419 | 0.51230138 | 0.787153883 |  |  | ASD | ASD_sc | FMRP |  |  |  |
| Ntsr2 | 0.668675273 | 1.70961E-13 | 7.79193E-12 |  |  |  |  |  |  |  |  |
| Nuak1 | -0.645701045 | 3.34195E-05 | 0.000428543 |  |  |  |  |  |  |  |  |
| Nub1 | -0.005660731 | 0.938201908 | 0.98530815 |  |  |  |  |  |  |  |  |
| Nubp1 | -0.103217902 | 0.439350064 | 0.738982731 |  |  |  |  |  |  |  |  |
| Nucb1 | 0.094575955 | 0.300572855 | 0.617878633 |  |  |  |  |  |  |  |  |
| Nucks1 | -0.029315304 | 0.881243243 | 0.960480474 |  |  |  |  |  |  |  |  |
| Nudc | 0.025877692 | 0.746780445 | 0.912020725 |  |  |  |  |  |  |  |  |
| Nudcd3 | 0.137678969 | 0.11430826 | 0.35979585 |  |  |  |  |  |  |  |  |
| Nudt13 | 0.04966871 | 0.840729659 | 0.94499151 |  |  |  |  |  |  |  |  |
| Nudt16 | -0.014699947 | 0.812997709 | 0.938538051 |  |  |  |  |  |  |  |  |
| Nudt16l1 | 0.29266393 | 0.006106347 | 0.039758552 |  |  |  |  |  |  |  |  |
| Nudt19 | 0.125594444 | 0.099187728 | 0.328538753 |  |  |  |  |  |  |  |  |
| Nudt3 | 0.049314346 | 0.914886313 | 0.974640474 |  |  |  |  |  |  |  |  |
| Nudt4 | -0.467527553 | 2.67316E-05 | 0.000351254 |  |  |  |  |  |  |  |  |
| Nudt9 | 0.059044673 | 0.763831632 | 0.919979858 |  |  |  |  |  |  |  |  |
| Nufip1 | -0.036283702 | 0.781356469 | 0.92656842 |  |  |  |  |  |  |  |  |
| Numa1 | 0.015239849 | 0.893225679 | 0.965099975 |  |  |  |  |  |  |  |  |
| Numb | -0.572814097 | 5.44736E-09 | 1.40609E-07 |  |  |  |  |  |  |  |  |
| Numbl | 0.199338088 | 0.137930224 | 0.405078683 | SYN |  |  |  |  | SZdb |  | SZ_full |
| Nup107 | 0.181465912 | 0.283057546 | 0.601723611 |  |  |  |  |  |  |  |  |
| Nup133 | 0.029528996 | 0.69099124 | 0.884120561 |  |  |  |  |  |  |  |  |
| Nup153 | -0.005431949 | 0.901228236 | 0.969020815 |  |  |  |  |  |  |  |  |
| Nup155 | 0.003858697 | 0.940334936 | 0.985559981 |  |  |  |  |  |  |  |  |
| Nup188 | -0.061492186 | 0.595381652 | 0.833964185 |  |  |  |  |  |  |  |  |
| Nup205 | -0.131613871 | 0.296459229 | 0.612828608 |  |  |  |  |  |  |  |  |
| Nup210 | -0.124114174 | 0.198782097 | 0.501101771 |  |  |  |  |  |  |  |  |
| Nup35 | 0.154516729 | 0.239330034 | 0.554531538 |  |  |  |  |  |  |  |  |
| Nup50 | 0.033643863 | 0.828309921 | 0.94210462 |  |  |  |  |  |  |  |  |
| Nup54 | 0.143222059 | 0.285738279 | 0.603881429 |  |  |  |  |  |  |  |  |
| Nup88 | -0.157898381 | 0.167522554 | 0.453699754 |  |  |  |  |  |  |  |  |
| Nup93 | 0.029044537 | 0.807735015 | 0.93712146 |  |  |  |  |  |  |  |  |
| Nupl2 | -0.155907971 | 0.230013497 | 0.543259595 |  |  |  |  |  |  |  |  |
| Nus1 | -0.035258907 | 0.868719484 | 0.955711255 |  |  |  |  |  |  |  |  |
| Nutf2 | 0.043490072 | 0.550466807 | 0.807666914 |  |  |  |  |  |  | SZ_108 | SZ_full |
| Nvl | -0.082355571 | 0.246445999 | 0.563448687 |  |  |  |  |  |  |  |  |
| Nwd1 | -0.101899878 | 0.252766024 | 0.570531033 |  |  |  |  | FMRP |  |  |  |
| Nxf1 | 0.162132221 | 0.091827682 | 0.310873339 |  |  |  |  |  |  |  |  |
| Nxpe3 | -0.113812299 | 0.542416634 | 0.803401127 |  |  |  |  |  |  |  |  |
| Nxpe4 | -0.158341416 | 0.099045837 | 0.328341479 |  |  |  |  |  |  |  |  |
| Nxph1 | -0.06169515 | 0.49507317 | 0.776692291 |  |  | ASD | ASD_sc |  |  |  |  |
| Nxph3 | 0.669577893 | 2.27419E-06 | 3.67929E-05 |  |  |  |  |  |  |  |  |
| Nxt2 | 0.048946254 | 0.553029818 | 0.808060922 |  |  |  |  |  |  |  |  |
| Nyap1 | -0.00750248 | 0.929748546 | 0.981434219 |  |  |  |  |  |  |  |  |
| Nyap2 | -1.015151551 | 6.21032E-21 | 5.00338E-19 |  |  |  |  |  |  |  |  |
| Oat | -0.014388041 | 0.743411126 | 0.911381362 |  | ID |  |  |  |  |  |  |
| Oaz1 | 0.058385167 | 0.514148026 | 0.787995408 |  |  |  |  |  |  |  |  |
| Oaz2 | -0.083377339 | 0.701828415 | 0.887565614 |  |  |  |  |  |  |  |  |
| Obsl1 | 0.021622804 | 0.961764196 | 0.989984854 |  |  |  |  |  |  |  |  |
| Ociad1 | 0.014912236 | 0.859604052 | 0.952644879 |  |  |  |  |  |  |  |  |
| Ociad2 | 0.780434475 | 3.78341E-13 | 1.66721E-11 |  |  |  |  |  |  |  |  |
| Ocrl | 0.041933256 | 0.686459456 | 0.882339565 |  | ID |  |  |  |  |  |  |
| Odc1 | 0.054980219 | 0.3462783 | 0.666324661 |  |  |  |  |  |  |  |  |
| Odf2 | -0.017149669 | 0.634165038 | 0.855565011 |  |  |  |  |  |  |  |  |
| Odf2l | 0.056019295 | 0.941233046 | 0.985779516 |  |  |  |  |  |  |  |  |
| Ogdh | -0.044988168 | 0.479318794 | 0.765984483 | SYN |  |  |  | FMRP |  |  |  |
| Ogdhl | -0.097712561 | 0.243967386 | 0.560127769 | SYN |  |  |  |  |  |  |  |
| Ogfod1 | 0.161978366 | 0.04907128 | 0.203426471 |  |  |  |  |  |  |  |  |
| Ogfod2 | 0.183447829 | 0.230192715 | 0.543521934 |  |  |  |  |  |  | SZ_108 | SZ_full |
| Ogfr | 0.051061018 | 0.611810329 | 0.843963886 |  |  |  |  |  |  |  |  |
| Ogfrl1 | 0.505061717 | 0.000100445 | 0.0011544 |  |  |  |  |  |  |  |  |
| Ogt | -0.214191406 | 0.011177082 | 0.064506807 | SYN |  | ASD |  |  |  |  |  |
| Ola1 | 0.111735838 | 0.280830473 | 0.598744681 | SYN |  |  |  |  |  |  |  |
| Olfm1 | 0.029544209 | 0.827912607 | 0.94210462 | SYN |  |  |  | FMRP |  |  |  |
| Olfm2 | -0.701662674 | 8.61967E-15 | 4.70894E-13 | SYN |  |  |  |  |  |  |  |
| Olfm3 | -0.218787351 | 0.138662593 | 0.40645823 |  |  |  |  |  |  |  |  |
| Olfml3 | 0.056047547 | 0.36251816 | 0.679700246 |  |  |  |  |  |  |  |  |
| Olig1 | 0.391529492 | 0.000278025 | 0.002865018 |  |  |  |  |  |  |  |  |
| Oma1 | -0.143452824 | 0.307245177 | 0.624472496 |  |  |  |  |  |  |  |  |
| Omg | -0.022132254 | 0.763355041 | 0.919979858 | SYN |  |  |  |  |  |  |  |
| Opa1 | -0.059955175 | 0.554113844 | 0.808611129 | SYN |  |  |  |  |  |  |  |
| Opalin | 0.381104034 | 0.275083104 | 0.592509543 |  |  |  |  |  |  |  |  |
| Opcml | 0.14612828 | 0.630103379 | 0.853812312 | SYN |  |  |  |  |  |  |  |
| Oplah | -0.033646619 | 0.753315363 | 0.915220708 |  |  |  |  |  |  |  |  |
| Optn | 0.402346493 | 0.004895579 | 0.033259912 |  |  |  |  |  |  |  |  |
| Orai2 | 0.774995641 | 2.37816E-16 | 1.59397E-14 |  |  |  |  |  |  |  |  |
| Orc2 | -0.372791999 | 0.000310887 | 0.003162798 |  |  |  |  |  |  |  |  |
| Orc3 | 0.091650199 | 0.225784792 | 0.538695632 |  |  |  |  |  |  |  |  |
| Orc4 | -0.060708041 | 0.493347222 | 0.776428066 |  |  |  |  |  |  |  |  |
| Orc6 | 0.034087347 | 0.851525393 | 0.949100969 |  |  |  |  |  |  |  |  |
| Os9 | 0.072219802 | 0.447209203 | 0.744686785 |  |  |  |  |  |  |  |  |
| Osbp | -0.004698064 | 0.915989704 | 0.97483035 |  |  |  |  |  |  |  |  |
| Osbp2 | -0.714264173 | 8.42369E-16 | 5.24901E-14 |  |  |  |  |  |  |  |  |
| Osbpl1a | -0.195109963 | 0.029780827 | 0.138260695 |  |  |  |  |  |  |  |  |
| Osbpl2 | -0.296799965 | 0.004717297 | 0.032351818 |  |  |  |  |  |  |  |  |
| Osbpl3 | 0.058795301 | 0.966424238 | 0.990416798 |  |  |  |  |  |  | SZ_108 | SZ_full |
| Osbpl5 | -0.838019065 | 1.8047E-15 | 1.06624E-13 |  |  |  |  |  |  |  |  |
| Osbpl6 | 0.032778329 | 0.945005905 | 0.985962641 |  |  |  |  |  |  |  |  |
| Osbpl8 | -0.391192984 | 0.055268205 | 0.219677921 |  |  |  |  |  |  |  |  |
| Osbpl9 | -0.193570919 | 0.017657749 | 0.092292404 |  |  |  |  |  |  |  |  |
| Osgepl1 | -0.13223841 | 0.275654806 | 0.593281983 |  |  |  |  |  |  |  |  |
| Ostm1 | 0.003287625 | 0.762980609 | 0.919979858 |  |  |  |  |  |  |  |  |
| Otub1 | 0.129613453 | 0.144122749 | 0.416343008 |  |  |  |  |  |  |  |  |
| Otub2 | 0.325016393 | 0.000634613 | 0.005976002 |  |  |  |  |  |  |  |  |
| Otud4 | -0.024285274 | 0.875670389 | 0.95886148 |  |  |  |  |  |  |  |  |
| Otud5 | -0.022716626 | 0.942789628 | 0.985900058 |  |  |  |  |  |  |  |  |
| Otud7a | -0.068455854 | 0.20857574 | 0.515341041 |  |  |  |  |  |  |  |  |
| Otud7b | 0.026552495 | 0.979752755 | 0.993959295 |  |  |  |  |  |  | SZ_108 | SZ_full |
| Otx1 | -0.402672921 | 0.031450202 | 0.144413825 |  |  | ASD |  |  |  |  |  |
| Ovca2 | -0.064336288 | 0.44946306 | 0.746235921 |  |  |  |  |  |  |  |  |
| Oxa1l | 0.003466571 | 0.871659068 | 0.956962054 |  |  |  |  |  |  |  |  |
| Oxct1 | -0.099652156 | 0.239458201 | 0.554531538 | SYN |  |  |  |  |  |  |  |
| Oxr1 | 0.084364661 | 0.27595674 | 0.593589794 | SYN |  |  |  | FMRP |  |  |  |
| P4ha1 | 0.411312958 | 6.89084E-06 | 0.000102924 |  |  |  |  |  |  |  |  |
| P4hb | 0.24989865 | 0.004449945 | 0.030782964 |  |  |  |  |  |  |  |  |
| P4htm | 0.130648074 | 0.173476937 | 0.462387242 |  |  |  |  |  |  |  |  |
| Pa2g4 | 0.15274287 | 0.109541925 | 0.350463857 |  |  |  |  |  |  |  |  |
| Pabpc1 | 0.159145792 | 0.090784425 | 0.308520056 |  |  |  |  |  |  |  |  |
| Pabpc4 | -0.040311868 | 0.541356898 | 0.8027259 | SYN |  |  |  |  |  |  |  |
| Pabpn1 | 0.096870276 | 0.348900246 | 0.668361715 |  |  |  |  |  |  |  |  |
| Pacrg | 0.345118246 | 0.020392042 | 0.103171124 |  |  |  |  |  |  |  |  |
| Pacrgl | 0.011822375 | 0.862910832 | 0.953793902 |  |  |  |  |  |  |  |  |
| Pacs1 | 0.079241435 | 0.474633331 | 0.76408458 | SYN |  |  |  | FMRP |  |  |  |
| Pacs2 | 0.192752114 | 0.174620786 | 0.463949163 |  |  |  |  | FMRP |  |  |  |
| Pacsin1 | -0.141574546 | 0.067659759 | 0.252883897 | SYN |  |  |  |  |  |  |  |
| Pacsin2 | -0.885005972 | 3.21829E-19 | 2.46818E-17 | SYN |  |  |  |  |  |  |  |
| Pacsin3 | -0.890276718 | 3.63603E-15 | 2.05681E-13 |  |  |  |  |  |  |  |  |
| Paf1 | 0.210483481 | 0.068112411 | 0.253861958 |  |  |  |  |  |  |  |  |
| Pafah1b1 | -0.083923964 | 0.499338651 | 0.77924576 | SYN | ID | ASD |  |  |  |  |  |
| Pafah1b2 | -0.063414906 | 0.837034234 | 0.943363721 |  |  |  |  |  |  |  |  |
| Pafah1b3 | 0.151571714 | 0.589292267 | 0.829543792 |  |  |  |  |  |  |  |  |
| Pagr1a | 0.138488725 | 0.26202621 | 0.5789255 |  |  |  |  |  |  |  |  |
| Paics | 0.041003988 | 0.470479583 | 0.762867484 | SYN |  |  |  |  |  |  |  |
| Paip1 | 0.016382744 | 0.705582602 | 0.889759184 |  |  |  |  |  |  |  |  |
| Paip2 | -0.102334671 | 0.212124955 | 0.51899038 |  |  |  |  |  |  |  |  |
| Paip2b | -0.092107397 | 0.589270037 | 0.829543792 |  |  |  |  |  |  |  |  |
| Pak1 | -0.688163292 | 8.39646E-16 | 5.24901E-14 | SYN |  |  |  |  |  |  |  |
| Pak1ip1 | 0.139904312 | 0.089001755 | 0.304016273 |  |  |  |  |  |  |  |  |
| Pak2 | 0.127942284 | 0.163986389 | 0.448083399 |  |  |  |  |  |  |  |  |
| Pak3 | -0.142311906 | 0.165760247 | 0.451061536 |  | ID |  |  |  |  |  |  |
| Pak6 | -0.995831994 | 7.13388E-22 | 6.25273E-20 |  |  |  |  | FMRP |  | SZ_108 | SZ_full |
| Pak7 | -0.878194761 | 1.84214E-12 | 7.42068E-11 |  |  |  |  |  |  |  |  |
| Palm | 0.050976203 | 0.853858804 | 0.950119195 | SYN |  |  |  |  |  |  |  |
| Palmd | 1.15383287 | 5.12708E-22 | 4.59479E-20 |  |  |  |  |  |  |  |  |
| Pam | 0.379420723 | 6.29956E-05 | 0.000760141 |  |  |  |  |  |  |  |  |
| Pan3 | -0.099094798 | 0.251542328 | 0.569325087 |  |  |  |  |  |  |  |  |
| Pank1 | -0.111662323 | 0.261633412 | 0.578754638 |  |  |  |  |  |  |  |  |
| Pank2 | -0.036787041 | 0.637122532 | 0.858538489 |  | ID |  |  |  |  |  |  |
| Pank3 | -0.013873872 | 0.951688946 | 0.988251608 |  |  |  |  |  |  |  |  |
| Pank4 | -0.023580786 | 0.64075466 | 0.860142008 |  |  |  |  |  |  |  |  |
| Panx1 | 0.128517255 | 0.305675063 | 0.62282745 |  |  |  |  |  |  |  |  |
| Panx2 | 0.534934389 | 7.87275E-05 | 0.000927519 |  |  |  |  |  |  |  |  |
| Papd5 | 0.003438739 | 0.851183322 | 0.949009034 |  |  |  |  |  |  |  |  |
| Papd7 | 0.086170249 | 0.284156893 | 0.602965919 |  |  |  |  |  |  |  |  |
| Papola | -0.035723287 | 0.72698735 | 0.901500482 |  |  |  |  |  |  |  |  |
| Papolg | 0.038517563 | 0.809555406 | 0.93712146 |  |  |  |  |  |  |  |  |
| Papss1 | 0.056996074 | 0.511545696 | 0.786598895 |  |  |  |  |  |  |  |  |
| Papss2 | 0.823547065 | 0.001018586 | 0.00906723 |  |  |  |  |  |  |  |  |
| Paqr3 | 3.60088E-05 | 0.985312304 | 0.995925857 |  |  |  |  |  |  |  |  |
| Paqr4 | 0.05030196 | 0.784766573 | 0.928282627 |  |  |  |  |  |  |  |  |
| Paqr7 | 0.288521141 | 0.005772433 | 0.037924983 |  |  |  |  |  |  |  |  |
| Paqr8 | 0.020258985 | 0.79686645 | 0.933720314 |  |  |  |  |  |  |  |  |
| Pard3 | -0.114161563 | 0.59760489 | 0.834762978 |  |  |  |  |  |  |  |  |
| Park7 | 0.09910623 | 0.400324435 | 0.71034209 | SYN |  |  |  |  |  |  |  |
| Parl | 0.02175907 | 0.946870921 | 0.986189928 |  |  |  |  |  |  |  |  |
| Parm1 | -0.2319885 | 0.25721264 | 0.574847386 |  |  |  |  |  |  |  |  |
| Parp1 | 0.175156896 | 0.054559898 | 0.217584872 | SYN |  |  |  |  |  |  |  |
| Parp11 | 0.069926317 | 0.609348213 | 0.84187794 |  |  |  |  |  |  |  |  |
| Parp16 | -0.214327268 | 0.251007801 | 0.568599324 |  |  |  |  |  |  |  |  |
| Parp2 | -0.062006699 | 0.456033243 | 0.752289793 |  |  |  |  |  |  |  |  |
| Parp6 | -0.267009926 | 0.002265591 | 0.017926938 |  |  |  |  |  |  |  |  |
| Parp8 | -1.1201931 | 6.81675E-17 | 4.72786E-15 |  |  |  |  |  |  |  |  |
| Parva | -0.41220628 | 0.001649073 | 0.013801681 |  |  |  |  |  |  |  |  |
| Patl1 | 0.111275796 | 0.523219064 | 0.79247916 |  |  |  |  |  |  |  |  |
| Patz1 | 0.105733241 | 0.236986941 | 0.551724415 |  |  |  |  |  |  |  |  |
| Paxbp1 | 0.037284453 | 0.877027834 | 0.959294295 |  |  |  |  |  |  |  |  |
| Paxip1 | -0.036524745 | 0.768628576 | 0.920494741 |  |  |  |  |  |  |  |  |
| Pbdc1 | -0.005672153 | 0.855941739 | 0.951377789 |  |  |  |  |  |  |  |  |
| Pbld1 | 0.097795962 | 0.87405929 | 0.958281361 |  |  |  |  |  |  |  |  |
| Pbrm1 | -0.081758779 | 0.624531731 | 0.850480636 |  |  |  |  |  |  | SZ_108 | SZ_full |
| Pbx1 | 0.58616997 | 6.27735E-12 | 2.40712E-10 |  |  |  |  |  |  |  |  |
| Pbx2 | 0.345687233 | 0.000782525 | 0.007157589 |  |  |  |  |  |  |  |  |
| Pbxip1 | -0.023905958 | 1 | 1 | SYN |  |  |  |  |  |  |  |
| Pcbp1 | 0.194223736 | 0.070293478 | 0.259805737 | SYN |  |  |  |  |  |  |  |
| Pcbp2 | -0.13942048 | 0.170467881 | 0.4591867 | SYN |  |  |  |  |  |  |  |
| Pcbp3 | -0.016981236 | 0.907979986 | 0.97125661 |  |  |  |  |  |  |  |  |
| Pcbp4 | 0.116884719 | 0.411233849 | 0.720088075 |  |  |  |  |  |  |  |  |
| Pcca | 0.061104554 | 0.711855459 | 0.893853769 | SYN | ID |  |  |  |  |  |  |
| Pccb | 0.047791637 | 0.629126723 | 0.85355374 |  | ID |  |  |  |  | SZ_108 | SZ_full |
| Pcdh1 | 0.360922938 | 0.000585251 | 0.005550487 | SYN |  |  |  | FMRP |  |  |  |
| Pcdh10 | 0.402917992 | 1.6858E-07 | 3.40404E-06 |  |  | ASD | ASD_sc | FMRP |  |  |  |
| Pcdh15 | -0.284875229 | 0.013190968 | 0.073369008 |  |  | ASD |  |  |  |  |  |
| Pcdh17 | 1.052551299 | 1.6312E-08 | 3.94256E-07 |  |  |  |  |  |  |  |  |
| Pcdh18 | 0.005748522 | 0.848320404 | 0.947466244 |  |  |  |  |  |  |  |  |
| Pcdh19 | -0.658813788 | 2.08674E-07 | 4.15058E-06 |  | ID | ASD | ASD_sc |  |  |  |  |
| Pcdh20 | -1.301068906 | 1.07835E-20 | 8.5158E-19 |  |  |  |  |  |  |  |  |
| Pcdh7 | 0.414905295 | 2.42561E-07 | 4.71869E-06 |  |  |  |  | FMRP |  |  |  |
| Pcdh8 | -0.319296609 | 0.286940815 | 0.604793246 |  |  | ASD |  |  | SZdb |  | SZ_full |
| Pcdh9 | -0.089784143 | 0.319421105 | 0.637244305 |  |  | ASD | ASD_sc | FMRP |  |  |  |
| Pcdha4-g | -0.071825609 | 0.261555387 | 0.578754638 |  |  |  |  |  |  |  |  |
| Pced1a | -0.030284101 | 0.538617085 | 0.801497271 |  |  |  |  |  |  |  |  |
| Pcf11 | 0.042996539 | 0.420832362 | 0.727945981 |  |  |  |  |  |  |  |  |
| Pcgf3 | 0.15556706 | 0.115211106 | 0.361923504 |  |  |  |  |  |  |  |  |
| Pcgf6 | -0.118863179 | 0.224058822 | 0.536517357 |  |  |  |  |  |  | SZ_108 | SZ_full |
| Pcid2 | 0.005073324 | 0.979505745 | 0.993945956 |  |  |  |  |  |  |  |  |
| Pcif1 | 0.01646229 | 0.734967171 | 0.906323154 |  |  |  |  |  |  |  |  |
| Pck2 | -0.165732102 | 0.224981573 | 0.537905582 |  |  |  |  |  |  |  |  |
| Pclo | 0.137697947 | 0.167301186 | 0.45325892 | SYN |  |  |  | FMRP |  |  |  |
| Pcm1 | 0.015503206 | 0.791499457 | 0.931893103 |  |  |  |  |  | SZdb |  | SZ_full |
| Pcmt1 | -0.211102715 | 0.013167789 | 0.073291198 | SYN |  |  |  |  |  |  |  |
| Pcmtd1 | -0.048963479 | 0.66536131 | 0.87416169 |  |  |  |  |  |  |  |  |
| Pcmtd2 | -0.055839727 | 0.602108426 | 0.837095487 |  |  |  |  |  |  |  |  |
| Pcna | 0.038386192 | 0.438371089 | 0.73827023 |  |  |  |  |  |  |  |  |
| Pcnp | -0.03763498 | 0.914696983 | 0.974568947 |  |  |  |  |  |  |  |  |
| Pcnt | -0.240292048 | 0.014027132 | 0.076893751 |  |  |  |  |  |  |  |  |
| Pcnx | -0.239684214 | 0.014563051 | 0.079124589 |  |  |  |  | FMRP |  |  |  |
| Pcnxl2 | -0.078159082 | 0.270900376 | 0.589228634 |  |  |  |  | FMRP |  |  |  |
| Pcnxl3 | -0.093036444 | 0.273358709 | 0.591112429 |  |  |  |  | FMRP |  |  |  |
| Pcnxl4 | 0.107086036 | 0.232898045 | 0.547317268 |  |  |  |  |  |  |  |  |
| Pcp4l1 | -0.124249038 | 0.585153698 | 0.827439595 |  |  |  |  |  |  |  |  |
| Pcsk1 | 0.187649103 | 0.274639312 | 0.592033284 |  |  |  |  |  |  |  |  |
| Pcsk1n | 0.271530124 | 0.284736111 | 0.602965919 |  |  |  |  |  |  |  |  |
| Pcsk2 | -0.224711092 | 0.006153757 | 0.040001931 |  |  |  |  |  |  |  |  |
| Pcsk5 | -0.402219558 | 0.011308352 | 0.064982286 |  |  |  |  |  |  |  |  |
| Pcx | 0.078273894 | 0.26795307 | 0.585532517 | SYN | ID |  |  |  |  |  |  |
| Pcyox1 | -0.089939176 | 0.444255755 | 0.742847778 |  |  |  |  |  |  |  |  |
| Pcyox1l | 0.211439343 | 0.072008762 | 0.262977055 |  |  |  |  |  |  |  |  |
| Pcyt1a | -0.006817442 | 0.976139075 | 0.992312677 |  |  |  |  |  |  |  |  |
| Pcyt1b | -0.017019161 | 0.826447674 | 0.941950287 |  |  |  |  |  |  |  |  |
| Pcyt2 | 0.141270077 | 0.251879169 | 0.569602566 |  |  |  |  |  |  |  |  |
| Pdap1 | 0.157541928 | 0.534003803 | 0.79794988 |  |  |  |  |  |  |  |  |
| Pdcd10 | -0.015358996 | 0.943357468 | 0.985962641 |  |  |  |  |  |  |  |  |
| Pdcd4 | 0.127617398 | 0.233124625 | 0.547688369 |  |  |  |  |  |  |  |  |
| Pdcd6 | -0.07017559 | 0.439925476 | 0.739573524 |  |  |  |  |  |  |  |  |
| Pdcd6ip | 0.068818934 | 0.409275331 | 0.719062719 | SYN |  |  |  |  |  |  |  |
| Pdcd7 | -0.012413017 | 0.80340198 | 0.936421772 |  |  |  |  |  |  |  |  |
| Pddc1 | 0.045526647 | 0.673425886 | 0.877823808 |  |  |  |  |  |  |  |  |
| Pde10a | 0.293895191 | 0.312273529 | 0.629439895 | SYN |  |  |  |  |  |  |  |
| Pde1a | -0.288452975 | 0.111669749 | 0.35499319 | SYN |  |  |  |  |  |  |  |
| Pde1b | -0.306247876 | 0.158789144 | 0.439757711 |  |  |  |  |  |  |  |  |
| Pde2a | 0.047756287 | 0.426922602 | 0.731029342 | SYN |  |  |  | FMRP |  |  |  |
| Pde4a | 0.359752627 | 2.92194E-05 | 0.000379567 |  |  | ASD |  |  |  |  |  |
| Pde4b | -0.169237953 | 0.071143905 | 0.261254045 | SYN |  | ASD |  | FMRP | SZdb |  | SZ_full |
| Pde4d | -0.561242885 | 3.42113E-11 | 1.20327E-09 | SYN |  |  |  |  |  |  |  |
| Pde4dip | -0.011940671 | 0.88217958 | 0.960863288 |  |  |  |  | FMRP |  |  |  |
| Pde5a | -0.381093633 | 0.118130128 | 0.366617081 |  |  |  |  |  |  |  |  |
| Pde7a | -0.375799866 | 0.000256438 | 0.002677156 |  |  |  |  |  |  |  |  |
| Pde7b | -1.069266487 | 1.4832E-09 | 4.13636E-08 |  |  |  |  |  |  |  |  |
| Pde8b | -0.595447636 | 3.67077E-11 | 1.28412E-09 |  |  |  |  | FMRP |  |  |  |
| Pdgfb | 0.475194619 | 1.21505E-06 | 2.12063E-05 |  |  |  |  |  |  |  |  |
| Pdgfra | -0.042521132 | 0.859090345 | 0.952559341 |  |  |  |  |  |  |  |  |
| Pdha1 | 0.086400772 | 0.202055478 | 0.505677595 | SYN | ID |  |  |  |  |  |  |
| Pdhb | -0.018052259 | 0.757180286 | 0.916833457 | SYN |  |  |  |  |  |  |  |
| Pdhx | -0.059555269 | 0.381231419 | 0.695268093 | SYN |  |  |  |  |  |  |  |
| Pdia3 | 0.033860201 | 0.620259211 | 0.84868447 | SYN |  |  |  |  |  |  |  |
| Pdia4 | 0.33011259 | 0.072558214 | 0.264345131 |  |  |  |  |  |  |  |  |
| Pdia6 | 0.240286291 | 0.057676772 | 0.226250076 | SYN |  |  |  |  |  |  |  |
| Pdk1 | -0.268520973 | 0.004772361 | 0.032645245 | SYN |  |  |  |  |  |  |  |
| Pdk2 | 0.057174867 | 0.401271 | 0.711185063 |  |  |  |  |  |  |  |  |
| Pdlim7 | -0.145201898 | 0.213302769 | 0.520155963 | SYN |  |  |  |  |  |  |  |
| Pdp1 | -0.978480003 | 1.49136E-06 | 2.53626E-05 | SYN |  |  |  |  |  |  |  |
| Pdpk1 | -0.1027093 | 0.381503237 | 0.695268093 | SYN |  |  |  |  |  |  |  |
| Pdpn | 0.15805951 | 0.325109987 | 0.642805468 |  |  |  |  |  |  |  |  |
| Pdpr | -0.018804118 | 0.640263893 | 0.860142008 |  |  |  |  |  |  |  |  |
| Pdrg1 | 0.415250408 | 3.82429E-05 | 0.000486484 |  |  |  |  |  |  |  |  |
| Pds5a | -0.011066323 | 0.993361394 | 0.999627867 |  |  |  |  |  |  |  |  |
| Pds5b | 0.030385116 | 0.427881147 | 0.73153195 |  |  |  |  | FMRP |  |  |  |
| Pdss1 | -0.042785632 | 0.514119206 | 0.787995408 |  |  |  |  |  |  |  |  |
| Pdxdc1 | 0.092737513 | 0.415319481 | 0.723588505 |  |  |  |  |  |  |  |  |
| Pdxk | -0.042357296 | 0.703483714 | 0.888798685 | SYN |  |  |  |  |  |  |  |
| Pdxp | 0.130026625 | 0.130437596 | 0.390822789 |  |  |  |  |  |  |  |  |
| Pdzd11 | -0.266453403 | 0.046110074 | 0.194562604 |  |  |  |  |  |  |  |  |
| Pdzd2 | -0.919293309 | 4.65677E-06 | 7.17035E-05 |  |  |  |  | FMRP |  |  |  |
| Pdzd4 | -0.046904178 | 0.64059169 | 0.860142008 |  |  | ASD |  |  |  |  |  |
| Pdzd8 | -0.108503318 | 0.728475309 | 0.902465385 |  |  |  |  | FMRP |  |  |  |
| Pdzrn3 | -0.767393343 | 3.60896E-16 | 2.35943E-14 |  |  |  |  |  |  |  |  |
| Pdzrn4 | -0.440302144 | 0.007067316 | 0.044844001 |  |  |  |  |  |  |  |  |
| Pea15a | 0.385230554 | 3.24523E-07 | 6.22211E-06 | SYN |  |  |  |  |  |  |  |
| Pebp1 | -0.03117311 | 0.638803957 | 0.859677519 | SYN |  |  |  |  |  |  |  |
| Pef1 | -0.122487232 | 0.212315255 | 0.519296681 |  |  |  |  |  |  |  |  |
| Peg10 | -0.691346915 | 2.05882E-09 | 5.66248E-08 |  |  |  |  |  |  |  |  |
| Peg3 | 0.109202912 | 0.446438475 | 0.744468592 |  |  |  |  | FMRP |  |  |  |
| Peli1 | -0.008454038 | 0.980877514 | 0.994381525 |  |  |  |  |  |  |  |  |
| Peli2 | 0.047907191 | 0.64817518 | 0.864441038 |  |  |  |  |  |  |  |  |
| Peli3 | 0.044182803 | 0.726551523 | 0.901240272 |  |  |  |  |  |  |  |  |
| Penk | 1.291487138 | 2.02807E-21 | 1.70273E-19 |  |  |  |  |  |  |  |  |
| Peo1 | 0.138413598 | 0.334350586 | 0.654906747 |  |  |  |  |  |  |  |  |
| Pepd | -0.100236663 | 0.30773465 | 0.624820652 |  | ID |  |  |  |  |  |  |
| Per1 | 0.155888501 | 0.196663209 | 0.498445165 |  |  | ASD | ASD_sc | FMRP |  |  |  |
| Per2 | 0.390256084 | 0.035650338 | 0.158941918 |  |  |  |  |  |  |  |  |
| Per3 | 0.78659479 | 1.121E-11 | 4.2375E-10 |  |  |  |  |  | SZdb |  | SZ_full |
| Pes1 | 0.140470665 | 0.172703567 | 0.461777959 | SYN |  |  |  |  |  |  |  |
| Pex1 | 0.088312519 | 0.573673504 | 0.820827732 |  | ID |  |  |  |  |  |  |
| Pex11a | -0.094461025 | 0.812116509 | 0.938012489 |  |  |  |  |  |  |  |  |
| Pex11b | -0.109370889 | 0.386303756 | 0.698810424 | SYN |  |  |  |  |  |  |  |
| Pex14 | -0.110827618 | 0.304536848 | 0.622162555 |  |  |  |  |  |  |  |  |
| Pex16 | -0.011167926 | 0.814166497 | 0.938538051 |  | ID |  |  |  |  |  |  |
| Pex19 | 0.077784961 | 0.379419372 | 0.693775541 |  | ID |  |  |  |  |  |  |
| Pex5 | -0.079435686 | 0.237979782 | 0.55290613 |  | ID |  |  |  |  |  |  |
| Pex5l | -0.429374875 | 2.02147E-05 | 0.000271435 | SYN |  |  |  |  |  |  |  |
| Pex6 | -0.090550962 | 0.365751127 | 0.683032308 |  | ID |  |  |  |  |  |  |
| Pex7 | -0.006695113 | 0.951531055 | 0.988251608 |  | ID | ASD |  |  |  |  |  |
| Pfas | 0.118787872 | 0.242092429 | 0.557749628 |  |  |  |  |  |  |  |  |
| Pfdn1 | 0.028038919 | 0.770546312 | 0.921167131 |  |  |  |  |  |  |  |  |
| Pfkfb2 | -0.006410078 | 0.68992612 | 0.883708164 |  |  |  |  |  |  |  |  |
| Pfkfb3 | 0.040266338 | 0.504431606 | 0.782850979 |  |  |  |  |  |  |  |  |
| Pfkl | 0.357494022 | 4.56995E-05 | 0.000565992 | SYN |  |  |  |  |  |  |  |
| Pfkm | -0.150555066 | 0.059805953 | 0.231784392 | SYN |  |  |  | FMRP |  |  |  |
| Pfkp | -0.041429758 | 0.613287907 | 0.844961985 | SYN |  |  |  |  |  |  |  |
| Pfn1 | 0.272951331 | 0.07443924 | 0.269142057 | SYN |  |  |  |  |  |  |  |
| Pfn2 | 0.049030015 | 0.374070836 | 0.689528308 | SYN |  |  |  |  |  |  |  |
| Pgam1 | 0.073817618 | 0.186814659 | 0.482680181 |  |  |  |  |  |  |  |  |
| Pgap1 | 0.196864085 | 0.214708582 | 0.520996547 |  |  |  |  |  |  |  |  |
| Pgbd5 | -0.124406592 | 0.126898654 | 0.384991884 |  |  |  |  |  |  |  |  |
| Pgk1 | -0.06983596 | 0.548355376 | 0.806991235 | SYN | ID |  |  |  |  |  |  |
| Pgls | 0.432954668 | 0.018333534 | 0.094891801 |  |  |  |  |  |  |  |  |
| Pgm2 | 0.239119488 | 0.033741919 | 0.152483791 |  |  |  |  |  |  |  |  |
| Pgm2l1 | 0.066276914 | 0.212956279 | 0.520155963 | SYN |  |  |  | FMRP |  |  |  |
| Pgm3 | 0.075851601 | 0.466338753 | 0.760422149 |  |  |  |  |  |  |  |  |
| Pgp | 0.084519111 | 0.471704488 | 0.76369839 |  |  |  |  |  |  |  |  |
| Pgrmc1 | 0.227565306 | 0.013167302 | 0.073291198 | SYN |  |  |  |  |  |  |  |
| Pgrmc2 | -0.136577223 | 0.350726372 | 0.669393047 |  |  |  |  |  |  |  |  |
| Phactr1 | -0.020134762 | 0.630321351 | 0.853845635 | SYN |  |  |  | FMRP |  |  |  |
| Phactr2 | -0.747266927 | 1.12022E-07 | 2.32076E-06 |  |  |  |  |  |  |  |  |
| Phactr3 | -0.152559167 | 0.05351164 | 0.215341962 |  |  |  |  |  |  |  |  |
| Phb2 | 0.184372678 | 0.085785964 | 0.297620203 | SYN |  |  |  |  |  |  |  |
| Phc1 | -0.081193671 | 0.331682585 | 0.651119936 |  |  |  |  |  |  |  |  |
| Phc2 | -0.009537518 | 0.749140243 | 0.913909847 |  |  |  |  |  |  |  |  |
| Phc3 | -0.048010731 | 0.574495187 | 0.821220039 |  |  |  |  |  |  |  |  |
| Phex | -0.098785765 | 0.734438181 | 0.90623127 |  |  |  |  |  |  |  |  |
| Phf10 | -0.115422498 | 0.40714003 | 0.717487601 |  |  |  |  |  |  |  |  |
| Phf14 | -0.052912964 | 0.638462826 | 0.859677519 |  |  |  |  |  |  |  |  |
| Phf15 | 0.027215817 | 0.573560862 | 0.820827732 |  |  |  |  |  |  |  |  |
| Phf20 | 0.114982991 | 0.208398102 | 0.515220745 |  |  |  |  | FMRP |  |  |  |
| Phf20l1 | -0.146750187 | 0.061085016 | 0.235615351 |  |  |  |  |  |  |  |  |
| Phf21a | 0.001386875 | 0.974488188 | 0.991898646 |  |  |  |  |  |  |  |  |
| Phf3 | 0.035240077 | 0.561383127 | 0.814107604 |  |  |  |  |  |  |  |  |
| Phf5a | -0.017476883 | 0.749726421 | 0.914065719 |  |  |  |  |  |  |  |  |
| Phgdh | 0.440747366 | 5.68879E-05 | 0.000694851 | SYN |  |  |  |  |  |  |  |
| Phip | -0.175772182 | 0.124160729 | 0.380155845 |  |  |  |  |  |  |  |  |
| Phka1 | -0.113868642 | 0.171044543 | 0.459498577 |  |  |  |  |  |  |  |  |
| Phka2 | 0.307095197 | 0.003293342 | 0.02405467 |  |  |  |  |  |  |  |  |
| Phkb | -0.350959223 | 0.001747097 | 0.014506261 |  |  |  |  |  |  |  |  |
| Phldb1 | 0.204676376 | 0.333770367 | 0.65393084 | SYN |  |  |  | FMRP |  |  |  |
| Phlpp1 | -0.014835446 | 0.902718609 | 0.969220665 |  |  |  |  |  |  |  |  |
| Phlpp2 | -0.097702595 | 0.205646965 | 0.511204322 |  |  |  |  |  |  |  |  |
| Phospho1 | -0.040578377 | 0.601397698 | 0.836949801 |  |  |  |  |  |  |  |  |
| Phpt1 | 0.041155484 | 0.810861071 | 0.93712146 |  |  |  |  |  |  |  |  |
| Phrf1 | 0.059523219 | 0.494358258 | 0.77668892 |  |  |  |  |  |  |  |  |
| Phtf1 | 0.03289319 | 0.968977692 | 0.991454046 |  |  |  |  |  |  |  |  |
| Phtf2 | -0.269226618 | 0.016228121 | 0.086232838 |  |  |  |  |  |  |  |  |
| Phyh | -0.090898515 | 0.832109458 | 0.943106438 |  |  |  |  |  |  |  |  |
| Phyhd1 | 0.126754488 | 0.501051962 | 0.781352415 |  |  |  |  |  |  |  |  |
| Phyhip | 0.055186329 | 0.524360203 | 0.7932511 | SYN |  |  |  | FMRP |  |  |  |
| Phyhipl | 0.238669497 | 0.002039862 | 0.016422737 |  |  |  |  |  |  |  |  |
| Pi4k2a | -0.077559107 | 0.443625136 | 0.742415879 |  |  |  |  |  |  |  |  |
| Pi4ka | -0.0785688 | 0.414978393 | 0.723182545 | SYN |  |  |  | FMRP | SZdb |  | SZ_full |
| Pi4kb | -0.10868346 | 0.279600228 | 0.597182756 |  |  |  |  |  |  |  |  |
| Pianp | 0.082840283 | 0.377118983 | 0.692586003 |  |  |  |  |  |  |  |  |
| Pias1 | -0.145866279 | 0.130944047 | 0.39189858 |  |  |  |  |  |  |  |  |
| Pias2 | -0.055986079 | 0.625140501 | 0.850614729 |  |  |  |  |  |  |  |  |
| Pias3 | 0.066551495 | 0.460938939 | 0.756470983 |  |  |  |  |  |  |  |  |
| Picalm | 0.074141845 | 0.232450904 | 0.546749752 | SYN |  |  |  |  |  |  |  |
| Pick1 | 0.041740351 | 0.964484837 | 0.990416798 | SYN |  |  |  |  | SZdb |  | SZ_full |
| Pid1 | -0.111973462 | 0.474201695 | 0.76408458 |  |  |  |  |  |  |  |  |
| Piezo1 | -0.343620299 | 0.047214236 | 0.198011372 |  |  |  |  |  |  |  |  |
| Pigf | 0.177481718 | 0.257225466 | 0.574847386 |  |  |  |  |  |  |  |  |
| Pigg | -0.050698061 | 0.691848346 | 0.884182408 |  |  |  |  |  |  |  |  |
| Pigh | -0.034382027 | 0.809236629 | 0.93712146 |  |  |  |  |  |  |  |  |
| Pigk | -0.258139602 | 0.006515977 | 0.041983856 | SYN |  |  |  |  |  |  |  |
| Pign | -0.154118894 | 0.341148055 | 0.6621047 |  |  |  |  |  |  |  |  |
| Pigo | 0.065100537 | 0.395986573 | 0.706474453 |  |  |  |  |  |  |  |  |
| Pigp | -0.051987714 | 0.846948919 | 0.947043961 |  |  |  |  |  |  |  |  |
| Pigq | 0.045781289 | 0.739819841 | 0.909494921 |  |  |  |  | FMRP |  |  |  |
| Pigr | 0.356934617 | 0.589705468 | 0.829832535 |  |  |  |  |  |  |  |  |
| Pigs | 0.056448547 | 0.52452436 | 0.7932511 |  |  |  |  |  |  |  |  |
| Pigt | -0.014653894 | 0.978497939 | 0.99346008 |  |  |  |  |  |  |  |  |
| Pigz | 0.05864067 | 0.825752771 | 0.941950287 |  |  |  |  |  |  |  |  |
| Pih1d1 | -0.104511692 | 0.299451756 | 0.616368311 |  |  |  |  |  |  |  |  |
| Pik3c2a | 0.050777916 | 0.494822645 | 0.77668892 |  |  |  |  |  |  |  |  |
| Pik3c2b | 0.254835738 | 0.024618989 | 0.119368424 |  |  |  |  |  |  |  |  |
| Pik3c3 | -0.016142129 | 0.957751554 | 0.989383033 |  |  |  |  |  | SZdb |  | SZ_full |
| Pik3ca | -0.186485873 | 0.067081735 | 0.251643028 | SYN |  |  |  |  |  |  |  |
| Pik3cd | 0.013342209 | 0.963474046 | 0.990416798 |  |  |  |  |  |  |  |  |
| Pik3ip1 | 0.287768269 | 0.011616728 | 0.066324283 |  |  |  |  |  |  |  |  |
| Pik3r1 | -0.267545261 | 0.004369881 | 0.030334351 |  |  |  |  |  |  |  |  |
| Pik3r2 | 0.070456863 | 0.486107716 | 0.771274148 |  |  | ASD |  |  |  |  |  |
| Pik3r3 | 0.262625025 | 0.012161454 | 0.068745398 |  |  |  |  |  |  |  |  |
| Pikfyve | -0.082124831 | 0.566789514 | 0.817784581 |  |  |  |  |  |  |  |  |
| Pin1 | -0.193714542 | 0.025720338 | 0.12298886 | SYN |  |  |  |  |  |  |  |
| Pink1 | -0.116161861 | 0.293908803 | 0.610791196 | SYN |  |  |  | FMRP |  |  |  |
| Pion | -0.008276633 | 0.689910416 | 0.883708164 |  |  |  |  |  |  |  |  |
| Pip4k2a | -0.182141733 | 0.071833138 | 0.262576126 | SYN |  |  |  |  | SZdb |  | SZ_full |
| Pip4k2b | -0.074498028 | 0.618463399 | 0.847941692 | SYN |  |  |  |  |  |  |  |
| Pip4k2c | 0.484804275 | 1.2963E-08 | 3.21096E-07 |  |  |  |  |  |  |  |  |
| Pip5k1a | 0.136554755 | 0.159410799 | 0.441129079 |  |  |  |  |  |  |  |  |
| Pip5k1c | -0.014381566 | 0.66735704 | 0.875092018 | SYN |  |  |  | FMRP |  |  |  |
| Pisd | 0.138887725 | 0.43866197 | 0.738351249 |  |  |  |  |  |  |  |  |
| Pisd-ps2 | 0.204238237 | 0.473975709 | 0.76408458 |  |  |  |  |  |  |  |  |
| Pisd-ps3 | 0.468316662 | 0.111322197 | 0.354029442 |  |  |  |  |  |  |  |  |
| Pithd1 | -0.095865536 | 0.287730108 | 0.606003523 |  |  |  |  |  |  |  |  |
| Pitpna | -0.079583194 | 0.385677181 | 0.698492545 | SYN |  |  |  |  |  |  |  |
| Pitpnb | -0.042049352 | 0.649435751 | 0.865480293 |  |  |  |  |  |  |  |  |
| Pitpnc1 | -0.476960735 | 1.81274E-07 | 3.63277E-06 |  |  |  |  |  |  |  |  |
| Pitpnm1 | -0.219401266 | 0.017711998 | 0.092515323 |  |  |  |  | FMRP |  |  |  |
| Pitpnm2 | 0.176192751 | 0.046042152 | 0.194405615 |  |  |  |  | FMRP |  | SZ_108 | SZ_full |
| Pitpnm3 | -0.152658753 | 0.134963964 | 0.399534068 |  |  |  |  |  |  |  |  |
| Pja1 | 0.239570063 | 0.002741424 | 0.020904012 |  |  |  |  |  |  | SZ_108 | SZ_full |
| Pja2 | 0.015603021 | 0.667473243 | 0.875092018 |  |  |  |  | FMRP |  |  |  |
| Pkd1 | 0.070833369 | 0.784679347 | 0.928282627 |  |  |  |  | FMRP |  |  |  |
| Pkd2 | 0.118351821 | 0.262439244 | 0.579517002 |  |  |  |  |  |  |  |  |
| Pkia | -0.135839366 | 0.482683155 | 0.767979423 |  |  |  |  |  |  |  |  |
| Pkm | 0.098124589 | 0.119171122 | 0.369016169 |  |  |  |  |  |  |  |  |
| Pkn1 | -0.489125775 | 8.84528E-07 | 1.57478E-05 | SYN |  |  |  |  |  |  |  |
| Pkn2 | -0.094942064 | 0.346002446 | 0.666324661 |  |  |  |  |  |  |  |  |
| Pknox2 | -0.523995472 | 0.00010812 | 0.00122844 |  |  |  |  |  |  |  |  |
| Pkp4 | 0.150514074 | 0.12332628 | 0.378618325 | SYN |  |  |  | FMRP |  |  |  |
| Pla2g15 | 0.128675529 | 0.33689548 | 0.657952583 |  |  |  |  |  |  | SZ_108 | SZ_full |
| Pla2g16 | 0.326776426 | 0.00173165 | 0.01440213 |  |  |  |  |  |  |  |  |
| Pla2g4e | -0.437418233 | 1.49834E-06 | 2.54271E-05 |  |  |  |  |  |  |  |  |
| Pla2g6 | 0.069793298 | 0.576370178 | 0.822118754 |  |  |  |  |  | SZdb |  | SZ_full |
| Pla2g7 | 0.036567392 | 0.519110819 | 0.790842978 |  |  |  |  |  |  |  |  |
| Plbd2 | -0.230685407 | 0.007005468 | 0.044558876 |  |  |  |  |  |  |  |  |
| Plcb1 | -0.285265944 | 0.000577296 | 0.00550779 | SYN |  | ASD |  | FMRP |  |  |  |
| Plcb3 | 0.058424296 | 0.392084456 | 0.703705135 |  |  |  |  |  |  |  |  |
| Plcb4 | -0.490385532 | 5.80058E-08 | 1.28873E-06 | SYN |  |  |  |  |  |  |  |
| Plcd3 | -0.529194558 | 8.17132E-07 | 1.46789E-05 | SYN |  |  |  |  |  |  |  |
| Plcg1 | -0.157788033 | 0.076543692 | 0.275005625 | SYN |  |  |  |  |  |  |  |
| Plch2 | -0.679060397 | 6.63775E-12 | 2.53314E-10 |  |  |  |  | FMRP |  | SZ_108 | SZ_full |
| Plcl1 | -0.059581497 | 0.592228932 | 0.831183874 |  |  |  |  |  |  | SZ_108 | SZ_full |
| Plcl2 | -0.192294615 | 0.206324794 | 0.511941136 |  |  |  |  |  |  |  |  |
| Plcxd1 | -0.10416158 | 0.396450632 | 0.706770282 |  |  |  |  |  |  |  |  |
| Plcxd2 | -1.409716008 | 5.52851E-31 | 8.64616E-29 |  |  |  |  |  |  |  |  |
| Plcxd3 | -0.141748053 | 0.591349146 | 0.830533684 |  |  |  |  |  |  |  |  |
| Pld2 | 0.283021863 | 0.011246873 | 0.064768998 |  |  |  |  |  |  |  |  |
| Pld3 | -0.144328086 | 0.03048054 | 0.14072494 | SYN |  |  |  | FMRP |  |  |  |
| Plec | 0.00838268 | 0.93978903 | 0.985376272 | SYN |  |  |  |  |  |  |  |
| Plekha1 | -0.025471371 | 0.755529599 | 0.915960493 | SYN |  |  |  |  |  |  |  |
| Plekha2 | -0.700725007 | 0.000146195 | 0.001608345 |  |  |  |  |  |  |  |  |
| Plekha5 | -0.183528819 | 0.021050833 | 0.105947723 | SYN |  |  |  |  |  |  |  |
| Plekha6 | -0.332104443 | 5.27231E-05 | 0.000646953 | SYN |  |  |  |  |  |  |  |
| Plekha7 | 0.315560504 | 0.001849752 | 0.015241348 |  |  |  |  |  |  |  |  |
| Plekhb1 | 0.305721441 | 0.064198626 | 0.243886832 |  |  |  |  |  |  |  |  |
| Plekhb2 | -0.146407976 | 0.130165949 | 0.390249192 |  |  |  |  |  |  |  |  |
| Plekhg5 | 0.167271886 | 0.063777658 | 0.24327623 |  |  |  |  |  |  |  |  |
| Plekhh1 | 0.247067269 | 0.255833005 | 0.573341963 |  |  |  |  |  |  |  |  |
| Plekhh2 | 0.058164848 | 0.684756327 | 0.881616863 |  |  |  |  |  |  |  |  |
| Plekhj1 | 0.096895852 | 0.459345061 | 0.754875159 |  |  |  |  |  |  |  |  |
| Plekhm2 | -0.105740343 | 0.150635998 | 0.426518798 |  |  |  |  |  |  |  |  |
| Plekhm3 | -0.260222994 | 0.012009455 | 0.068127606 |  |  |  |  |  |  |  |  |
| Plekhn1 | -0.059887993 | 0.7870503 | 0.92972648 |  |  |  |  |  |  |  |  |
| Plekho1 | -0.219135899 | 0.037895658 | 0.166807818 |  |  |  |  |  |  | SZ_108 | SZ_full |
| Plekho2 | -0.151738974 | 0.211414759 | 0.518502158 |  |  |  |  |  |  |  |  |
| Plgrkt | 0.022246725 | 0.647048193 | 0.86359712 |  |  |  |  |  |  |  |  |
| Plk1s1 | 0.084493247 | 0.516184125 | 0.789167066 |  |  |  |  |  |  |  |  |
| Plk2 | 0.143865482 | 0.053704923 | 0.215593817 |  |  |  |  |  |  |  |  |
| Pllp | 0.379038701 | 0.042976235 | 0.184686665 | SYN |  |  |  |  |  |  |  |
| Plod1 | 0.350628336 | 0.004965444 | 0.0335915 |  |  |  |  |  |  |  |  |
| Plp1 | 0.554043644 | 0.010948624 | 0.063485466 | SYN | ID |  |  | FMRP | SZdb |  | SZ_full |
| Plp2 | 0.587566989 | 0.005966882 | 0.039073772 |  |  |  |  |  |  |  |  |
| Pls3 | 0.615959493 | 0.000131835 | 0.001472711 |  |  |  |  |  |  |  |  |
| Plscr3 | -0.12859208 | 0.272653481 | 0.590748776 |  |  |  |  |  |  |  |  |
| Pltp | 0.169095644 | 0.209314243 | 0.516221607 |  |  |  |  |  |  |  |  |
| Plxdc1 | -0.425136904 | 0.009626662 | 0.0575148 |  |  |  |  |  |  |  |  |
| Plxdc2 | -0.13783441 | 0.128313312 | 0.38780863 |  |  |  |  |  |  |  |  |
| Plxna1 | 0.404362094 | 1.35657E-06 | 2.34199E-05 | SYN |  |  |  | FMRP |  |  |  |
| Plxna2 | 0.034567866 | 0.509969621 | 0.786093307 | SYN |  |  |  | FMRP | SZdb |  | SZ_full |
| Plxna3 | -0.049013469 | 0.63172267 | 0.854727738 | SYN |  |  |  |  |  |  |  |
| Plxna4 | 0.404517479 | 0.00241863 | 0.018857276 | SYN |  | ASD |  | FMRP |  |  |  |
| Plxnb1 | 0.041415221 | 0.518523614 | 0.790473159 |  |  |  |  | FMRP |  |  |  |
| Plxnb2 | 0.117862177 | 0.116940527 | 0.364627696 |  |  |  |  |  |  |  |  |
| Plxnb3 | 0.530612977 | 0.000996462 | 0.008903352 |  |  |  |  |  |  |  |  |
| Plxnd1 | -0.675355423 | 2.28737E-05 | 0.000303058 |  |  |  |  | FMRP |  |  |  |
| Pmepa1 | -0.182266979 | 0.080839851 | 0.285932882 |  |  |  |  |  |  |  |  |
[truncated: 225,779 more chars]
